# A Modular Synthesis of Teraryl‐Based α‐Helix Mimetics, Part 4: Core Fragments with Two Halide Leaving Groups Featuring Side Chains of Proteinogenic Amino Acids

**DOI:** 10.1002/ejoc.202101279

**Published:** 2022-02-24

**Authors:** Melanie Trobe, Julia Blesl, Martin Vareka, Till Schreiner, Rolf Breinbauer

**Affiliations:** ^1^ Institute of Organic Chemistry Graz University of Technology Stremayrgasse 9 8010 Graz Austria

**Keywords:** Imidazole, Inhibitors, Peptide mimetics, Protein-protein interaction, Suzuki coupling

## Abstract

Teraryl‐based α‐helix mimetics have proven to be useful compounds for the inhibition of protein‐protein interactions (PPI). We have developed a modular and flexible approach for the synthesis of teraryl‐based α‐helix mimetics using a benzene core unit featuring two halide leaving groups of differentiated reactivity in the Pd‐catalyzed cross‐coupling used for teraryl assembly. The use of para‐bromo iodoarene core fragments resolved the issue of hydrolysis during cross‐coupling that was observed when using triflate as a leaving group. We report a complete set of para‐bromoiodoarene core fragments decorated with side chains of all proteinogenic amino acids relevant for PPI (Ala, Arg, Asn, Asp, Cys, Gln, Glu, His, Ile, Leu, Lys, Met, Phe, Ser, Thr, Trp, Tyr and Val). In order to be compatible with general cross‐coupling conditions, some of the nucleophilic side chains had to be provided in a protected form to serve as stable building blocks.

## Graphical abstract

A series of para‐bromoiodoarene compounds decorated with the side chains of all proteinogenic amino acids is reported. These fragments serve as building blocks for the modular assembly of teraryl‐based α‐helix mimetics via sequential Pd cross‐coupling. Compared to our established iodotriflate approach, the new building blocks showed higher stability and better reactivity under cross‐coupling conditions.

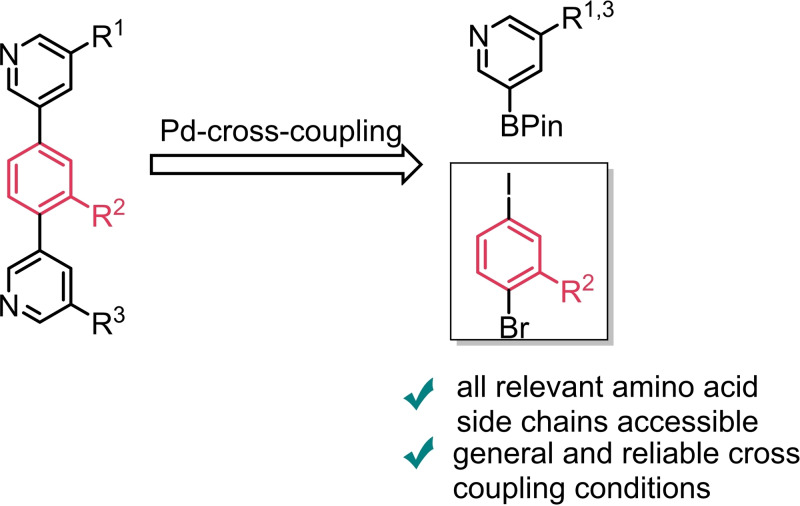

## Introduction

In the highly connected human proteome, protein‐protein interactions (PPIs) play a critical role in various biological processes.^[1]^ The number of different PPIs in the human proteome is estimated to be beyond 110.000,[^2^, ^3^] which offers a huge opportunity for Chemical Biology.^[3]^ Achieving control of these interactions with small molecule inhibitors is recognized as a new and important goal in drug design,^[4]^ but also implies considerable challenges. The flat and rather large interfaces of PPIs lack defined binding‐cavities.^[5]^ As a consequence it is difficult to find small molecules that are able to bind strongly enough to the protein to either inhibit the interaction or induce further downstream effects by activating the protein upon binding.^[6]^ Gratifyingly, structural analysis of many different PPIs has revealed that only a small number of amino acid residues within a binding site, which are called “hot spots”, is responsible for the vast majority of the binding energy.^[7]^ Therefore, a small molecule which can interact with these hot spots should result in an efficient inhibitor. It has been shown that in ∼60 % of all PPI sites these hot spots are arranged in an α‐helical fold.^[8]^ With this structural analysis in mind, Hamilton and co‐workers have found that the substituents of a terarylic structure overlap almost perfectly with *i*, *i*+3 (or *i*+4) and *i*+7 amino acid side chain residues of a folded α‐helix.^[9]^ However, limited solubility and synthetic accessibility is a major drawback of the terphenyl compound type.^[10]^

**Figure 1:**
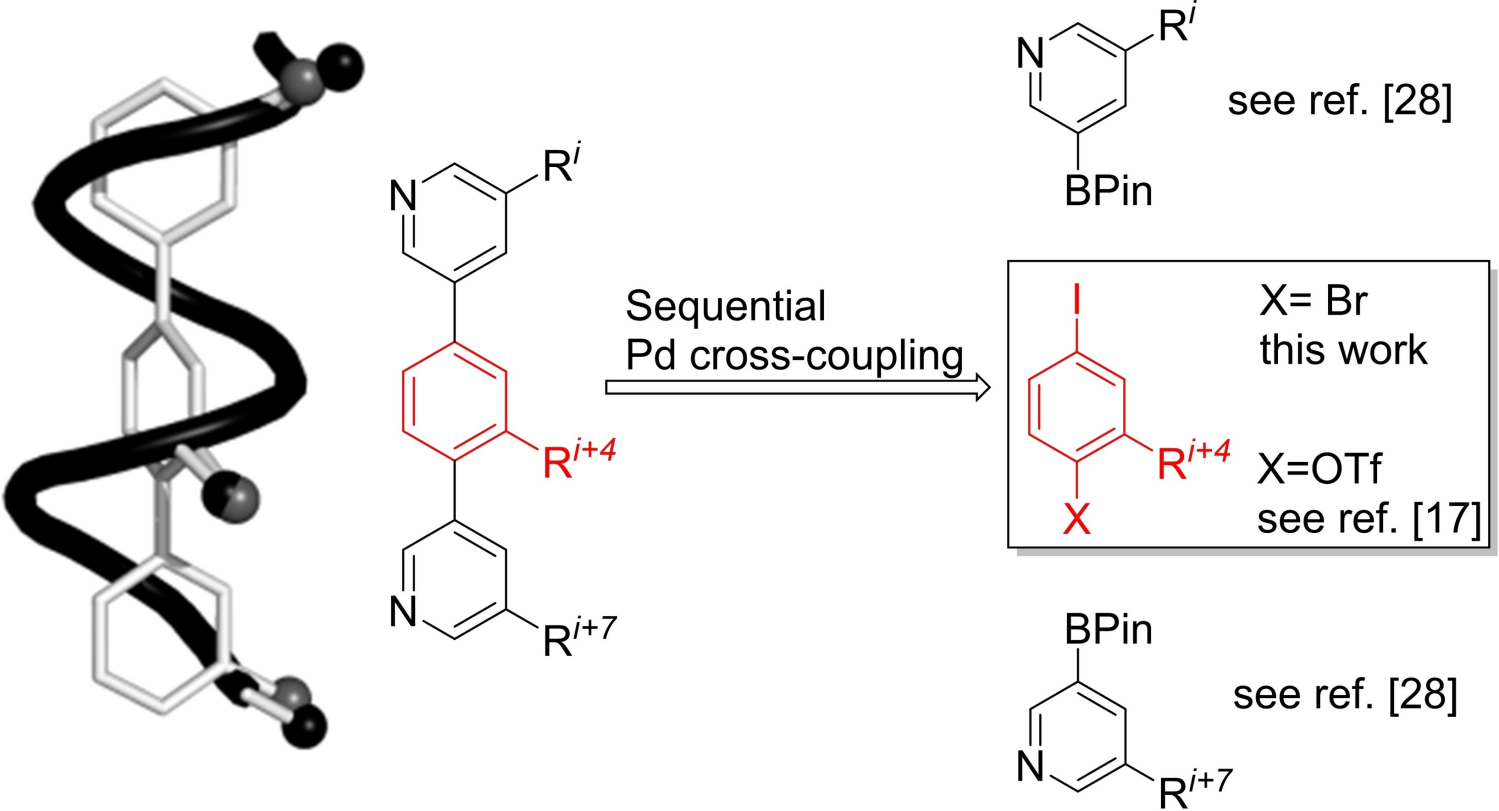
Design principle of teraryl‐based alpha‐helix mimetics (BPin: boronic acid pinacol ester).

Therefore, our group introduced a more versatile approach for synthesizing teraryls,[^11^, ^12^, ^13^, ^14^, ^15^] based on the modular assembly of a limited set of building blocks via sequential Suzuki‐couplings.^[16]^ By defining each ring of the terarylic structure as building block, the side chains responsible for the interaction with proteins can be easily exchanged.^[14]^ Furthermore, by replacing the top and bottom phenyl rings with two pyridine moieties we improved the aqueous solubility of the resulting teraryls. The nitrogen of the added heterocycles is placed at the water‐exposed face distal to the protein binding site, increasing the polar character of the molecule and reducing the entropic cost of binding.^[11]^ With bench stable building blocks in hand we were then able to readily prepare teraryls in a two‐step cross‐coupling procedure.[^11^, ^12^, ^13^] In our earlier approach we focused on using building blocks containing iodine and triflate as electronically differentiated leaving groups. However, in some cases hydrolysis of the triflate group during Pd‐catalyzed cross coupling led to undesired by‐products and low yields.^[17]^ We reasoned that an iterative Pd‐catalyzed cross coupling of building blocks with electronically differentiated halogen leaving groups could avoid this problem (Figure 1).

Herein, we disclose the synthesis of a full set of such aromatic building blocks featuring iodide and bromide as differentiated leaving groups, which contain the side chains of all 18 proteinogenic amino acids relevant for α‐mimetics.^[18]^

## Results and Discussion

In our synthetic efforts we set the goal to provide synthetic routes which are scalable to provide the building blocks in gram quantities as needed for a library synthesis effort. Furthermore, general intermediates were used as often as possible to ensure access to all building blocks, while minimizing the synthetic effort.

**Scheme 1:**
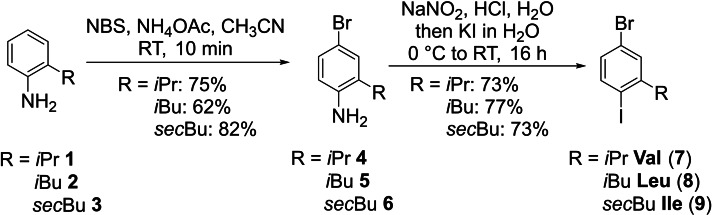
Synthesis of the Val, Leu and Ile building blocks (NBS: *N*‐bromo succinimide).

In general, four different strategies were used to synthesize the targeted building blocks. The first one utilized electrophilic aromatic halogenation followed by diazonium salt formation to introduce a second halide via a Sandmeyer type reaction. It turned out to be instrumental to introduce the more stable bromide first followed by conversion of the amine to the more reactive and labile iodide.^[19]^ Commercially available 2‐alkyl substituted anilines **1**–**3** were treated with a slight excess of NBS to ensure full conversion as the corresponding dibrominated side products were easily separated by column chromatography whereas unreacted starting material could not be separated utilizing this purification method. The subsequent diazotation with NaNO_2_/HCl and reaction with KI proceeded nicely and gave access to building blocks **7**–**9**, mimicking the Val, Leu and Ile side chains, in 50–60 % yield over two steps (Scheme 1).

**Scheme 2:**
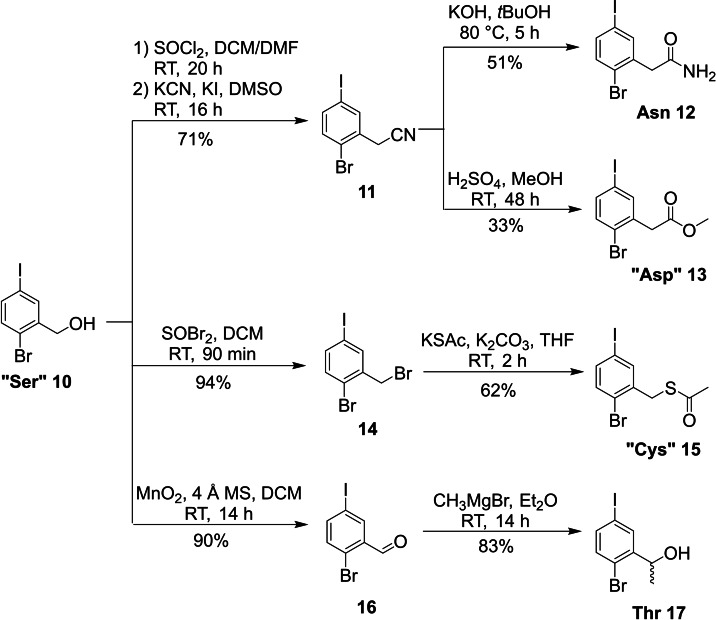
Synthesis of the Asn, “Asp”, “Cys” and Thr building blocks.

Conveniently, the Ser core unit fragment **10** is already commercially available but served also as the common starting material for the synthesis of the next series of core unit fragments including Asn, “Asp” (Please note that core fragments marked with “ “ were synthesized in a protected form.), “Cys” and Thr (Scheme 2). To access the first three, the alcohol was converted into the corresponding benzyl chloride or bromide with SOCl_2_ and SOBr_2_, respectively. The chloride was then substituted with KCN to form benzonitrile **11**, which served as precursor for the synthesis of Asn or “Asp” depending on the conditions used for further conversion. Basic hydrolysis of **11** with KOH yielded the free amide **12**, which represents the Asn core unit fragment, in 36 % overall yield, whereas acidic hydrolysis of **11** with H_2_SO_4_ in MeOH led to methyl ester **13** as a protected form of the Asp (“Asp”) core unit fragment in 23 % overall yield. Benzylic bromide **14** served as intermediate for the “Cys” core unit fragment **15**. In this case it was substituted with KSAc, furnishing the desired thioester building block **15** in 58 % total yield over two steps.[^12^, ^14^, ^20^] The acetyl group conveniently serves as protecting group of the thiol during cross‐coupling to avoid catalyst poisoning.^[21]^ For the synthesis of the Thr core unit fragment alcohol **10** was first oxidized to the corresponding aldehyde with MnO_2_. 4 Å molecular sieves were added to avoid over‐oxidation of the aldehyde to the carboxylic acid via an aldehyde hydrate. After reaction with MeMgBr a racemic mixture of the Thr core fragment **17** containing the secondary alcohol motif could be isolated in 75 % yield over two steps. We are aware that the racemic side chains of the Ile and Thr building blocks **9** and **17** do not strictly represent the chiral nature of the corresponding (enantiomerically pure) proteinogenic amino acids. However, at the early stage of screening compounds for biological activity against a new target we believe that the incorporation of these racemic building blocks with their better synthetic accessibility could even offer additional advantages as they allow direct exploration of both stereoisomers and might show better solubility. Upon detection of interesting biological activity, the enantiomers could be separated or synthesized using well established methods, such as asymmetric hydrogenation of alkenes^[22]^ or asymmetric reduction of ketones.^[23]^

**Scheme 3:**
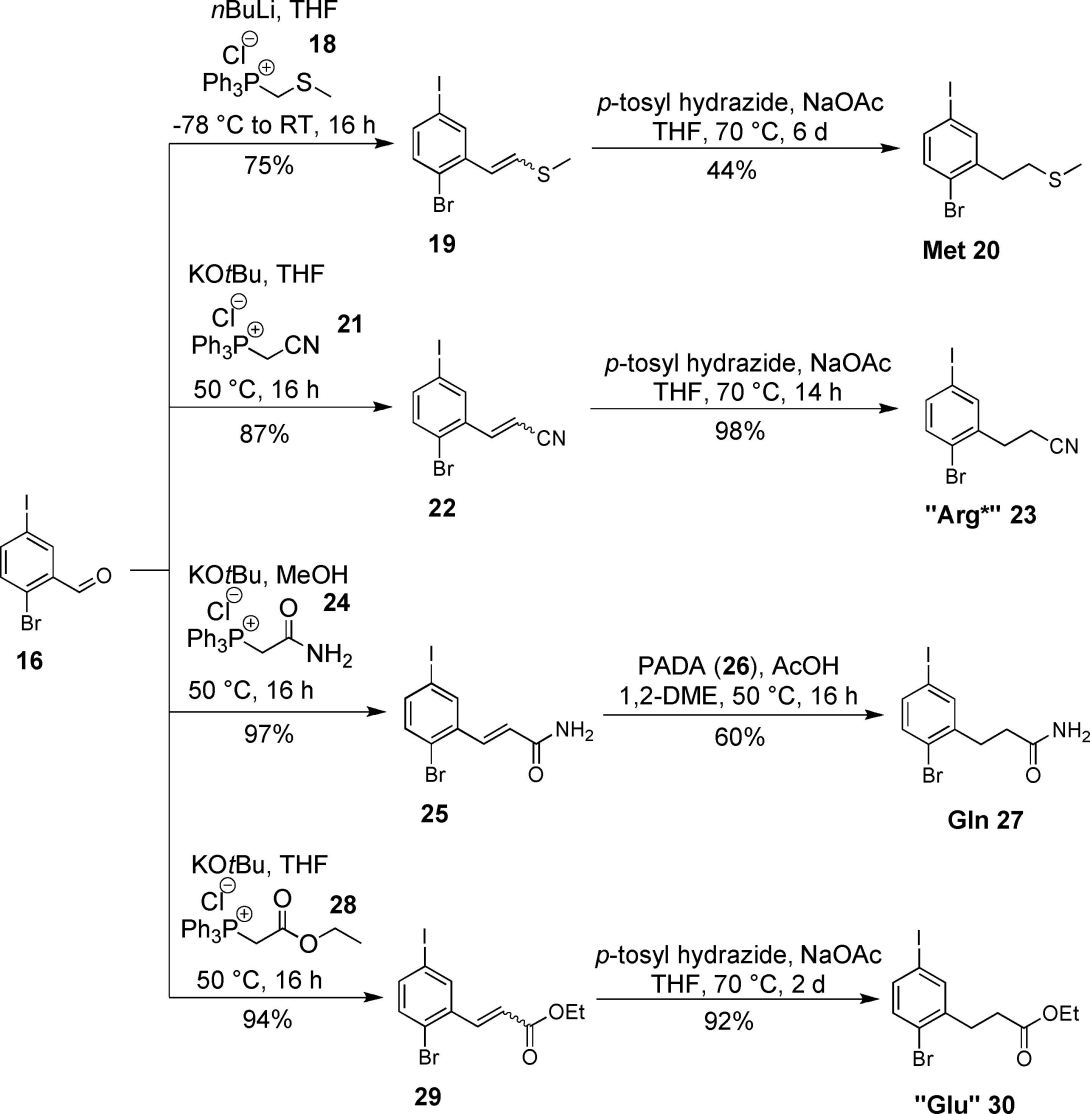
Synthesis of the Met, “Arg*”, Gln and “Glu” building blocks (1,2‐DME: 1,2‐dimethoxyethane; PADA: dipotassium azodicarboxylate).

A third set of building blocks was synthesized via the modification of benzaldehyde **16** utilizing a Wittig reaction/diimide reduction sequence (Scheme 3). This synthetic strategy could be applied for the synthesis of the Met, Gln and “Glu” core unit fragments as well as the Arg core unit precursor (“Arg*”). (Please note that core fragments marked with “*” were synthesized in a latent form and have to be converted into the desired functional group after cross coupling.) In previous studies we observed that having the guanidyl residue already attached to a building block before teraryl assembly renders this building block less reactive during cross‐coupling even when the basic nitrogen atoms are presented in Boc‐protected form.^[15]^ Therefore, we decided to prepare “Arg*” in masked form as a nitrile, which after cross‐coupling will be reduced to the corresponding amine and transformed to the guanidyl residue.

The conditions of the Wittig reaction were slightly varied for the introduction of each side chain to ensure formation and isolation of each building block in maximum yield. In the last step the double bond was reduced to produce the actual core fragment. Since all building blocks contain iodine as well as bromine classical hydrogenation with Pd/C was not considered as it would lead to dehalogenation of the carefully installed aryl halides. Therefore, we decided to use diimide reduction, which has already served us well in the synthesis of the I/OTf core fragment series.^[13]^ In general, *p*‐tosyl hydrazide/NaOAc was used for the in situ generation of diimide. Met **20** (30 % overall yield), “Glu” **30** (78 % overall yield) and “Arg*” **23** (77 % overall yield) were smoothly formed in this reaction. However, in the case of the Gln fragment the desired product could not be separated from excess *p*‐tosyl hydrazide. Therefore the protonation of potassium azodicarboxylate^[24]^ (PADA) (**26**) with AcOH was found as a more suitable way for diimide generation and the Gln building block **27** was isolated in 52 % overall yield.

**Scheme 4:**
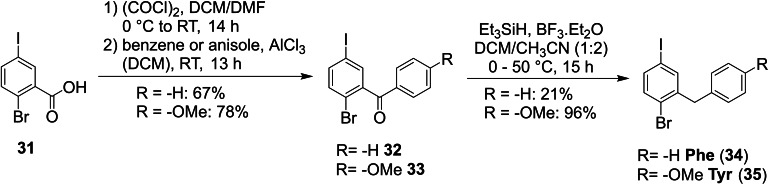
Synthesis of the Phe and “Tyr” core fragments.

The set of Phe and “Tyr” core unit fragments (Scheme 4) could be synthesized via Friedel‐Crafts acylation of arenes with 2‐bromo‐5‐iodobenzoic acid chloride.^[25]^ Benzene was used as both reagent as well as solvent to deliver ketone **32** in 67 % yield. Similarly, anisole was used for the introduction of the “Tyr” side chain protected as a methyl ether, to avoid side reactions with the free phenol. In the second step the formed ketones were reduced with Et_3_SiH in the presence of BF_3_ ⋅ Et_2_O. For the production of Phe building block **34**, only poor conversion was achieved at RT and the corresponding secondary alcohol was identified as main product. This unsatisfactory reaction outcome reflects the less pronounced +M‐effect of a phenyl vs. *p*‐MeO‐phenyl group, which is key in the formation of the benzhydryl‐carbocation intermediate. Ultimately, after heating to 50 °C for 4 d full conversion was achieved and the Phe core fragment **34** could be isolated in 14 % yield over three steps. In contrast, the reduction of the anisole‐derived ketone **33** proceeded smoothly at RT and methyl protected “Tyr” (**35**) could be isolated in 75 % over three steps.

**Scheme 5:**
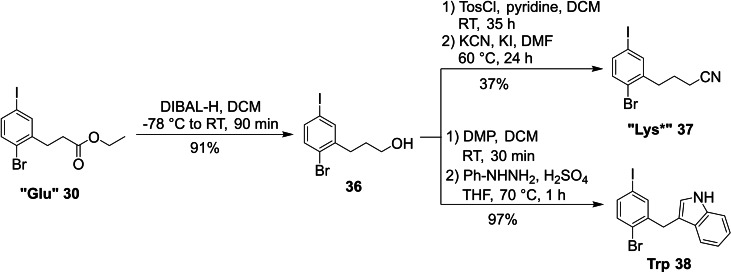
Synthesis of the “Lys*” and Trp building blocks (Tos: tosyl; DMP: Dess‐Martin periodinane).

For the three remaining core fragments “Lys*”, Trp and His building block precursors could be reused, for which the synthesis has been described above (Scheme 5). For the “Lys*” and Trp core unit fragments “Glu” (**30**) could be used as intermediate. Reduction of the ester **30** with DIBAL‐H smoothly yielded alcohol **36** (91 % yield), which was further converted to a tosylate and subsequently substituted with KCN, furnishing “Lys*” building block **37** in 26 % overall yield after six steps. The nitrile was used as Lys precursor and can be reduced to the free amine after cross‐coupling. To access the Trp building block **38**, previously synthesized alcohol **36** was oxidized to the corresponding aldehyde using Dess‐Martin periodinane. This set the stage for a Fischer indole synthesis with phenylhydrazine and H_2_SO_4_ as a catalyst, furnishing **38** in an excellent 97 % yield over two steps (Scheme 5).

**Scheme 6:**
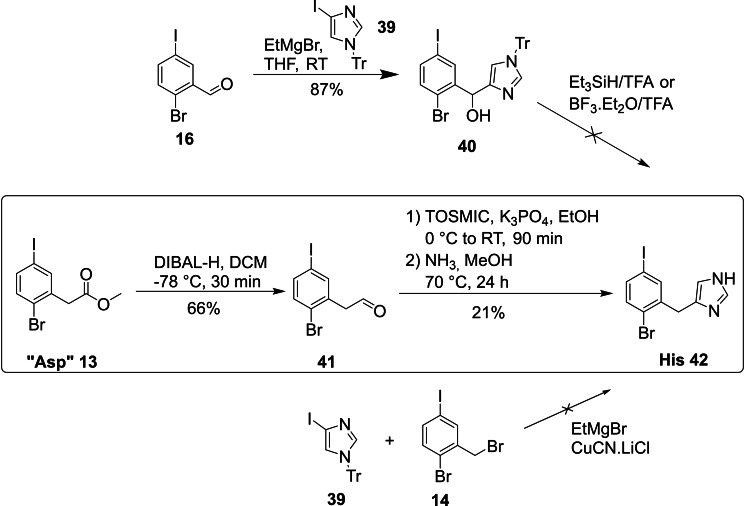
Synthesis of the His building block (TFA: trifluoroacetic acid; TOSMIC: tosylmethyl isocyanide; Tr: trityl).

Many unsuccessful attempts at synthesizing the His core unit led us to explore several routes for this challenging building block (Scheme 6). In our first strategy we performed a metal‐halogen exchange with EtMgBr on trityl (Tr) protected 4‐iodo imidazole **39**,^[26]^ followed by addition to aldehyde **16**. While the secondary benzylic alcohol **40** was isolated in 87 % yield, removal of the hydroxyl group to the desired His core fragment **42** could not be accomplished. Under acidic, reducing conditions (such as Et_3_SiH/TFA) which are necessary for deoxygenation of hydroxyl groups only decomposition of the compound was observed. Since attempts to assemble the building block via benzylation of cuprated imidazole substrate **39** ^[26]^ with benzyl bromide **14** had also failed, we decided to pursue an alternative approach. Therefore we first constructed the carbon framework of the building block and then synthesized the heterocycle from scratch – a strategy which had served us well in the synthesis of the Trp building block. The Asp core unit fragment **13** was reduced directly with DIBAL‐H to the corresponding aldehyde **41** (66 % yield), which was then further converted to the imidazole moiety via a van Leusen synthesis.^[27]^ First, an oxazoline was formed under base catalysis with K_3_PO_4_ by the addition of tosylmethyl isocyanide (TosMIC) to **41** followed by treatment with 7 M NH_3_ solution in MeOH to generate the imidazole heterocycle. The His fragment **42** was isolated in 14 % over the last three steps.

**Scheme 7:**
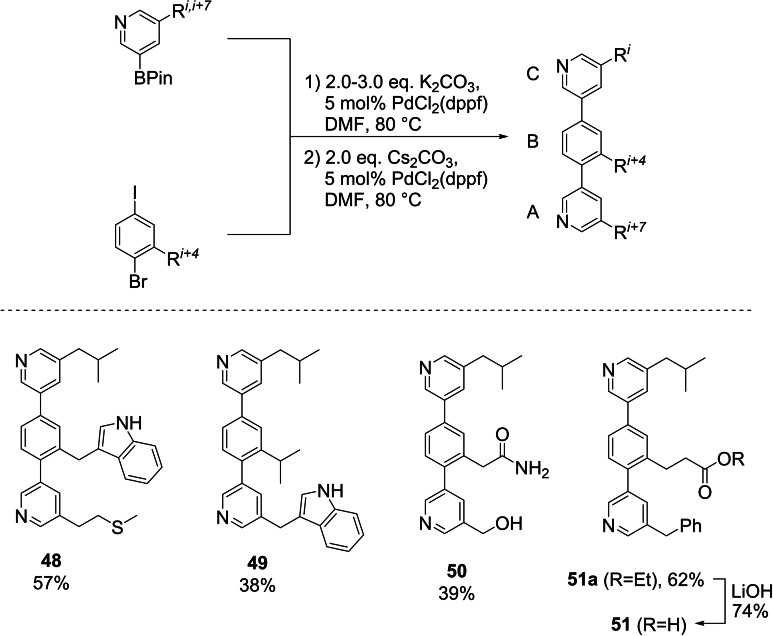
Teraryl assembly (dppf: 1,1’‐bis(diphenylphosphino)ferrocene). Please note that the order of addition of the pyridine building blocks was reversed in case of **49** due to the different I/Br substitution pattern on the Val core building block.

With the complete iodo bromo building block set in hand we synthesized a set of teraryls featuring different side chains to highlight the differentiated reactivity between the two halide leaving groups and the functional group tolerance of this approach (Scheme 7). Similarly to our coupling reactions with the already established I/OTf core building blocks,^[14]^ PdCl_2_(dppf) served as an efficient catalyst. K_2_CO_3_ was found to be the best base for the first coupling step with the iodide leaving group, while for the less reactive bromide leaving group a switch to the stronger base Cs_2_CO_3_ was necessary.

With these general coupling conditions in hand several different side chains with varying polarity could be incorporated into the teraryl scaffold. In the first step, the core fragments Trp (**38**), Val (**7**), Asn (**12**) and “Glu” (**30**) were connected to either the Leu or Trp pyridine boronic acid building block^[28]^ via the more reactive iodide leaving group. The reaction occurred chemoselectively and no cross‐coupling with the bromide was detected. The corresponding diaryl bromides were isolated in 54–80 % yield (see SI for details). In the second step, coupling of the bromide leaving group with the Met, Leu, “Ser” or Phe pyridine boronic acid building block^[28]^ also proceeded very smoothly, furnishing teraryls **48**–**51 a** in 38–62 % total yield. TBDPS deprotection of the pyridine “Ser” building block in **50** already occurred during cross coupling, while teraryl **51 a** was saponified to reveal the free Glu side chain, producing Leu‐Glu‐Phe teraryl **51** in 74 % yield.

## Conclusion

**Figure 2:**
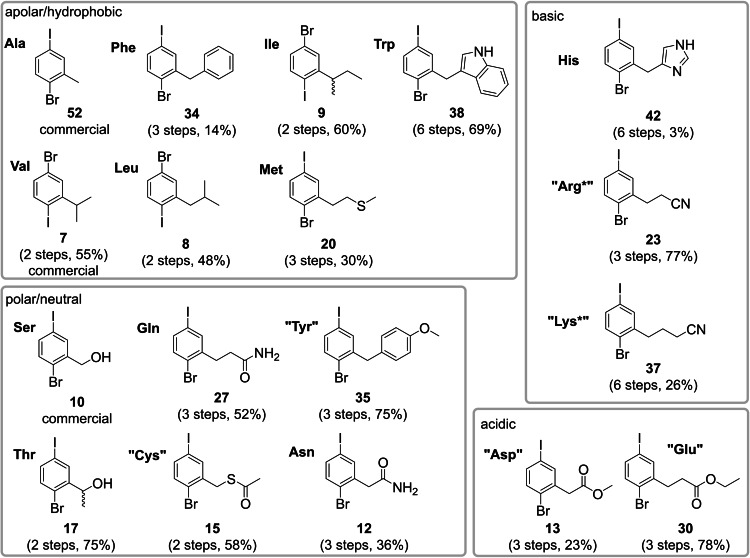
Comprehensive set of I/Br core fragments.

In conclusion, we have established the synthesis of a comprehensive set of iodo‐bromo core unit fragments featuring all relevant proteinogenic amino acids to be used as building blocks for the modular synthesis of teraryl‐based α‐helix mimetics (Figure 2). By replacing the −OTf leaving group with a −Br we could avoid the issue of hydrolysis of the triflate to the unreactive phenol during cross coupling. This alternative strategy also gives access to the His core unit fragment, which could not be synthesized as the iodine triflate equivalent. We notice that some of the apolar, hydrophobic building blocks such as Leu or Phe are easier accessible via the iodine triflate approach[^12^, ^13^] and could be conveniently used for the assembly of teraryls featuring non‐functionalized side chains. For teraryls with polar side chains we recommend the Br/I approach described here. With these two approaches we can now provide a practical solution for the synthesis of any core fragment building block featuring the side chains of all proteinogenic amino acid side chains. Together with our similarly comprehensive approach to 5‐substituted 3‐pyridine boronic acids,[^11^, ^28^] any motif of α‐helix‐hot spots has become accessible.

## Experimental Section

### Representative procedure for the synthesis of teraryls by consecutive double Suzuki‐Coupling (1^st^ step)

A flame dried Schlenk‐flask was charged with 1.0 eq. of the appropriate core fragment, 1.0–1.1 eq. boronic acid pinacol ester, 2.0–3.0 eq. K_2_CO_3_ and 0.05 eq. PdCl_2_(dppf)⋅DCM. The Schlenk‐flask was flushed with argon and absolute, degassed DMF (∼0.2 M) was added. The reaction mixture was stirred at 80 °C until full conversion was detected by TLC or GC‐MS (generally 3–4 h). The resulting dark brown suspension was concentrated under reduced pressure. The crude product was purified via flash column chromatography.

### Representative procedure for the synthesis of teraryls by consecutive double Suzuki‐Coupling (2^nd^ step)

A flame dried Schlenk‐flask was charged with 1.0 eq. of the corresponding bromo derivative form previous step, 1.0–1.1 eq. boronic acid pinacol ester, 2.0–3.0 eq. Cs_2_CO_3_ and 0.05 eq. PdCl_2_(dppf)⋅DCM. The Schlenk‐flask was flushed with argon and absolute, degassed DMF (∼0.2 M) was added. The reaction mixture was stirred at 80 °C for 18 h. The resulting dark brown suspension was concentrated under reduced pressure and the crude product was purified via flash column chromatography.

## Conflict of interest

The authors declare no conflict of interest.

## Supporting information

As a service to our authors and readers, this journal provides supporting information supplied by the authors. Such materials are peer reviewed and may be re‐organized for online delivery, but are not copy‐edited or typeset. Technical support issues arising from supporting information (other than missing files) should be addressed to the authors.

Supporting Information
