# Supplementary material for: A Modular Synthesis of Teraryl‐Based α‐Helix Mimetics, Part 4: Core Fragments with Two Halide Leaving Groups Featuring Side Chains of Proteinogenic Amino Acids
Source: European J Org Chem. 2022 Feb 24;2022(17):e202101279. doi: 10.1002/ejoc.202101279 (PMC9304293; doi:10.1002/ejoc.202101279)

European Journal of Organic Chemistry

Supporting Information

A Modular Synthesis of Teraryl-Based α-Helix Mimetics, Part 4: Core Fragments with Two Halide Leaving Groups Featuring Side Chains of Proteinogenic Amino Acids

Melanie Trobe, Julia Blesl, Martin Vareka, Till Schreiner, and Rolf Breinbauer*

1 General Experimental Aspects, Materials and Methods

NMR spectra were recorded on a Bruker Avance III 300 MHz FT NMR spectrometer (300.36 MHz (^{1}H), 75.53 MHz (^{13}C)), or on a Varian Unity Inova 500 MHz NB high resolution FT NMR spectrometer (499.76 MHz (^{1}H), 125.67 MHz (^{13}C)) at 27 °C. Chemical shifts δ [ppm] are referenced to residual protonated solvent signals as internal standard [D6]DMSO: δ = 2.50 ppm (^{1}H), 39.52 ppm (^{13}C) and $CDCl_3$: δ = 7.26 ppm (^{1}H), 77.16 ppm (^{13}C). Signal multiplicities are abbreviated as s (singlet), d (doublet), dd (doublet of doublet), t (triplet), dt (doublet of triplet), q (quadruplet), dq (doublet of quadruplet), sept (septet), m (multiplet) with the prefix b in case of broad signals. Superscript abbreviations are used as follows: H^{Ar} (phenyl); abbreviation C_q is used for quaternary carbon atoms. ^{13}C NMR resonances were assigned by APT or 2D-HSQC and HMBC experiments. GC-MS measurements were performed on an Agilent Technologies 7890A (G3440A) GC system equipped with an Agilent Technologies J&W GC-column HP-5MS ((5%-phenyl)-methylpolysiloxane; length: 30 m; inner-diameter: 0.250 mm; film: 0.25 µm) at a constant helium flow rate (He 5.0; Air Liquide; "Alphagaz"; 1.085 mL/min; average velocity 41.6 cm/s) in split mode 1/175 (inlet temperature: 250 °C; injection volume: 2.0 µL; sample concentration: ~0.5 mg/mL in ethyl acetate (EtOAc), methanol (MeOH), dichloromethane (DCM), or diethyl ether (Et_2O)). The GC was coupled to a 5975C inert mass sensitive detector with triple-axis detector (MSD, EI, 70 eV; transfer line: 300 °C; MS source: 240 °C; MS quad: 180 °C), with a solvent delay of 2.60 min. One general gradient "Method_1" (initial temperature: 50 °C, 1.0 min; linear ramp: 40 °C/min; final temperature: 300 °C; final time: 5.0 min; post run 1.0 min; detecting range: 50.0 to 550.0 amu) was applied. When reactions were monitored by GC-MS, the samples were prepared using a microscale workup. This means, an aliquot was taken from the reaction mixture, quenched with ~1 mL aqueous solution and ~1 mL DCM, EtOAc, or Et_2O. After proper mixing and phase separation, the organic layer was collected, dried over $MgSO_4$ and filtered through cotton in a Pasteur-pipette. Reaction mixtures containing transition metals were additionally filtered through a short pad of silica gel (~1 cm) over cotton in a Pasteur-pipette (eluted with EtOAc or MeOH). Analytical thin layer chromatography (TLC) was performed on Merck silica gel 60-F254 and spots were visualized by UV-light (λ = 254 and/or 366 nm), and by treatment with cerium ammonium molybdate solution (CAM) (CAM: 2.0 g Ce(IV)SO_4, 50 g $(NH_4)_2MoO_4$, 50 mL conc. H_2SO_4 in 400 mL water), vanillin solution (15 g vanillin in 250 mL ethanol and 2.5 mL concentrated H_2SO_4), ninhydrin solution (1.5 g ninhydrin in 100 mL n-butanol and 3.0 mL acetic acid) or $FeCl_3$ solution (5 g $FeCl_3$ in 100 mL 0.1M HCl), followed by warming with a heat gun.

Flash column chromatography was performed using silica gel 60 Å (35-70 µm particle size) from Acros Organics at an air pressure of ~1.5 bar. A 20 to 100-fold excess of silica gel was used with respect to the amount of dry raw material (exact values are given in experimental procedures). The stationary phase was filled in an appropriately sized column resulting in a pad of 15-25 cm silica gel. The column was equilibrated with the solvent or solvent mixture, and the sample was loaded onto the pad by diluting the crude product with the eluent. If the crude product was not soluble in the eluent, the sample was dissolved in a proper solvent (MeOH or EtOAc), and the double amount of silica gel (or Celite®545, particle size 0.02-0.1 mm) was added, followed by removing the solvent using a rotary evaporator and drying in vacuo. The mobile phase was forced through the column by means of a rubber bulb pump. Analytical high-performance liquid chromatography was performed on an "Agilent Technologies 1200 Series" HPLC system with 1260 HiP Degasser G4225A, binary pump SL G1312, autosampler HiP-ALS SL G1367C, thermostated column compartment TCC SL G1316B, multiple wavelength detector G1365C MWD SL with deuterium lamp (λ = 190 – 400 nm) and subsequent connected mass detector (Agilent Technologies 6120 Quadrupole LC/MS) with an electrospray ionization (ESI) source. The separation was performed with a reversed phase column ("Poroshell® 120 SB-C18EC, 3.0 x 100 mm, 2.7 µm" from Agilent Technologies with a Merck LiChroCART® 4-4 pre-column). Signals were detected at 210 nm or 254 nm. As mobile phase acetonitrile (VWR HiPerSolv, HPLC-MS grade) and water (deionized and filtered through a 0.2 µm cellulose nitrate membrane filter) with 0.05% TFA were used. One general gradient "MV_general" (0.0 – 0.1 min, isocratic, 2% MeCN (98% H_2O + 0.05% TFA); 0.1 – 8.0 min, linear, 2% to 100% MeCN; 8.0 – 11.1 min, isocratic, 100% MeCN; 11.1 – 11.3 min, linear, 100% to 2% MeCN; 11.3 – 12.0 min, isocratic, 2% MeCN) was applied. Reversed phase preparative HPLC purifications were performed on a Thermo Scientific UltiMate 3000 system. Detection was accomplished with a Dionex UltiMate Diode Array Detector. The separations were carried out on a Macherey Nagel 125/21 Nucleodur® 100-5 C18EC (125 x 21 mm, 5 µm) column. Acetonitrile and water with 0.05% HCOOH were used as eluents for the purification. The following method was applied: MV_NucleodurC18_001HCOOH_70to100 (0.0 – 13.0 min, linear, 70% CH_3CN to 100% CH_3CN, 13.0 – 15.0 min, isocratic, 100% CH_3CN, flow rate: 12 mL/min, 15.0 – 16.0 min, linear, 100% CH_3CN to 70% CH_3CN, 16 – 18 min, isocratic, 70% CH_3CN, T = 30 °C, flow rate: 12 mL/min). High Resolution Mass Spectrometry (HRMS) was performed on a Waters GCT Premier Micromass (Direct Inlet (DI-EI) or matrix-assisted laser desorption/ionization (MALDI)). Melting points were determined on a "Mel-Temp" melting-point apparatus

(Electrothermal) and are given uncorrected. Chemicals were purchased from Sigma-Aldrich, Fisher Scientific, Merck, or Alfa Aesar. All reagents were used without further purification unless otherwise noted. For determination of concentration of the alkyl-lithium solution in n-hexane a procedure according to Kofron and Baclawksi was used,[1] concentration of Grignard solutions were determined following a procedure of Watson and Eastham.[2] DCM was first tried over P_2O_5, distilled, then further dried over CaH_2 and distilled under an argon atmosphere before use. THF was dried by heating under reflux in an atmosphere of argon over Na, until benzophenone indicated dryness by a deep blue color and stored over 4Å molecular sieves in an amber glass Schlenk-flask under an argon atmosphere. Molecular sieves were activated by filling a 500 mL round-bottomed flask to one third of its volume with molecular sieves (Sigma-Aldrich, beads, 8-12 mesh) and heating the flask in a heating mantle (~150 °C) under oil pump vacuum for ~3 d, followed by cooling to RT under an atmosphere of argon. When working at a temperature of 0 °C, an ice-water bath served as the cooling agent, and -78 °C was achieved by a dry ice/acetone mixture.

2 Experimental Procedures and Analytical Data for Building Block Synthesis

2.1 Synthesis of Valine, Leucine and Isoleucine building blocks

2.1.1 4-Bromo-2-isopropylaniline

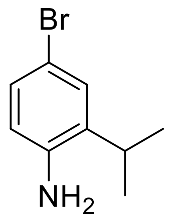

4

In a 100 mL one-neck round-bottom flask 29 mg (380 µmol, 0.10 eq) NH_4OAc were dissolved in 15 mL MeCN. 518 µL (3.70 mmol, 1.00 eq) 2-Isopropylaniline (**1**) and 692 mg (3.89 mmol, 1.05 eq) NBS were added. The red suspension was stirred at RT for 10 min. After full conversion was detected via GC-MS the solvent was removed under reduced pressure. The residue was diluted with 20 mL EtOAc and washed with $NaHSO_3$ solution (3 x 20 mL). The combined aqueous layers were reextracted with EtOAc (2 x 20 mL). The combined organic layers were dried over Na_2SO_4,

filtered and the solvent was removed under reduced pressure. The red brown oil was purified via flash column chromatography (110 g SiO_2, 4.0 x 13.0 cm, eluent: cyclohexane/EtOAc = 12/1, fraction size: 50 mL).

Yield: 587 mg (75%), brown oil, $C_9H_{12}BrN$ [213.02 g/mol]

TLC: R_f = 0.15 (cyclohexane/EtOAc = 12/1, UV and $KMnO_4$); **GC-MS** (Method_1): t_R = 5.59 min, *m/z* = 213 (59%), 198 (100%), 171 (4%), 119 (87%), 91 (19%); **^{1}H-NMR** (300.36 MHz, $CDCl_3$): δ = 7.21 (d, ^4J (H,H) = 1.7 Hz, 1H, H^{Ar}), 7.10 (dd, ^3J (H,H) = 8.3, ^4J (H,H) = 2.0 Hz, 1H, H^{Ar}), 6.55 (d, ^3J (H,H) = 8.4 Hz, 1H, H^{Ar}), 3.65 (bs, 2H, NH_2), 2.84 (hept, ^3J (H,H) = 6.7 Hz, 1H, CH), 1.24 (d, ^3J (H,H) = 6.8 Hz, 6H, CH_3) ppm; **^{13}C-NMR** (75.53 MHz, $CDCl_3$): δ = 142.5 (C_q, C^{Ar}), 134.9 (C_q, C^{Ar}), 129.3 (C^{Ar}), 128.5 (C^{Ar}), 117.4 (C^{Ar}), 111.1 (C_q, C^{Ar}), 28.0 (CH), 22.2 (CH_3) ppm.

Analytical data are in accordance with those reported.[3]

2.1.2 1-Bromo-4-iodo-2-isopropylbenzene

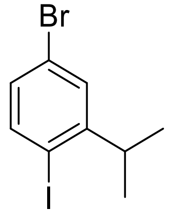

7

In a 100 mL one-neck round-bottom flask 570 mg (2.68 mmol, 1.00 eq) 4-bromo-2-isopropylaniline (**4**) were suspended in 3 mL dist. H_2O and cooled to 0 °C. 1.6 mL conc. HCl were added dropwise. Then a cooled solution of 314 mg $NaNO_2$ in 2.4 mL H_2O was added at 0 °C and the suspension was stirred at 0 °C. After 1 h a solution of 866 mg KI (5.21 mmol, 1.94 eq) in 3.5 mL H_2O was added dropwise at 0 °C. The brown suspension was stirred at RT for 16 h. After full conversion of the starting material was indicated by GC-MS, the reaction was diluted with 30 mL EtOAc and washed with half-satd. $NaHSO_3$ solution (3 x 20 mL). The combined aqueous layers were reextracted with EtOAc (2 x 30 mL). The combined organic layers were dried over Na_2SO_4, filtered and the solvent was removed under reduced pressure. The brown oil was purified via flash column chromatography (100 g SiO_2, 4.0 x 12.5 cm, eluent: cyclohexane, fraction size: 20 mL).

Yield: 637 mg (73%), brown oil, $C_9H_{10}BrI$ [323.90 g/mol]

TLC: R_f = 0.80 (cyclohexane, UV and $KMnO_4$); **GC-MS** (Method_1): t_R = 5.66 min, *m/z* = 323 (100%), 309 (100%), 182 (57%), 102 (49%); **^{1}H-NMR** (300.36 MHz, $CDCl_3$): δ = 7.66 (d, ^3J (H,H) = 8.4 Hz, 1H, H^{Ar}), 7.34 (d, ^3J (H,H) = 2.1 Hz, 1H, H^{Ar}), 7.01 (dd, ^3J (H,H) = 8.4, ^4J (H,H) = 2.3 Hz, 1H, H^{Ar}), 3.14 (hept, ^3J (H,H) = 6.8 Hz, 1H, CH), 1.22 (d, ^3J (H,H) = 6.8 Hz, 6H, CH_3) ppm; **^{13}C-NMR** (75.53 MHz, $CDCl_3$): δ = 152.8 (C_q, C^{Ar}), 140.9 (C^{Ar}), 130.9 (C^{Ar}), 129.4 (C^{Ar}), 123.2 (C_q, C^{Ar}), 99.0 (C_q, C^{Ar}), 38.3 (CH), 23.0 (CH_2) ppm.

Analytical data are in accordance with those reported.[3]

2.1.3 4-Bromo-2-isobutylaniline

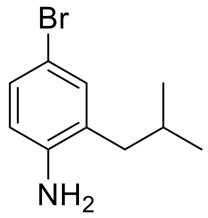

5

In a 10 mL one-neck round-bottom flask 9.0 mg (117 µmol, 0.11 eq) NH_4OAc were dissolved in 5 mL MeCN. 166 mg (1.11 mmol, 1.00 eq) 2-isobutylaniline (**2**) and 208 mg (1.17 mmol, 1.05 eq) NBS were added. The red suspension was stirred at RT for 10 min. After full conversion was detected via GC-MS the solvent was removed under reduced pressure. The residue was diluted with 20 mL EtOAc and washed with half-satd. $NaHSO_3$ solution (3 x 20 mL). The combined aqueous layers were reextracted with EtOAc (2 x 20 mL). The combined organic layers were dried over Na_2SO_4, filtered and the solvent was removed under reduced pressure. The brown oil was purified via flash column chromatography (40 g SiO_2, 3.0 x 10.5 cm, eluent: cyclohexane/EtOAc = 20/1, fraction size: 20 mL).

Yield: 156 mg (62%), red-brown oil, $C_{10}H_{14}BrN$ [227.03 g/mol]

TLC: R_f = 0.17 (cyclohexane/EtOAc = 20/1, UV and CAM); **GC-MS** (Method_1): t_R = 5.81 min, *m/z* = 227 (29%), 184 (100%), 104 (14%), 78 (12%); **^{1}H-NMR** (300.36 MHz, $CDCl_3$): δ = 7.11 (m, 2H, H^{Ar}), 6.55 (d, ^3J (H,H) = 9.0 Hz, 1H, H^{Ar}), 3.60 (bs, 2H, NH_2), 2.33 (d, ^3J (H,H) = 7.2 Hz, 2H, CH_2), 1.91 (hept, ^3J (H,H) = 6.7 Hz, 1H, CH), 0.95 (d, ^3J (H,H) = 6.6 Hz, 6H, CH_3) ppm; **^{13}C-**

NMR (75.53 MHz, $CDCl_3$): δ = 143.5 (C_q, C^{Ar}), 133.2 (C^{Ar}), 129.7 (C^{Ar}), 128.2 (C_q, C^{Ar}), 117.3 (C^{Ar}), 110.4 (C_q, C^{Ar}), 40.8 (CH_2), 27.9 (CH), 22.8 (CH_3) ppm; **HRMS** (DI-EI TOF): calcd. (*m/z*) for [M^+]: 227.0310; found: 226.9697.

2.1.4 4-Bromo-1-iodo-2-isobutylbenzene

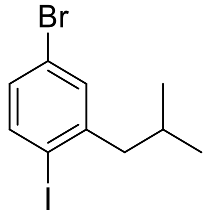

8

In a 50 mL one-neck round-bottom flask 97 mg (425 µmol, 1.00 eq) 4-bromo-2-isobutylaniline (**5**) were suspended in 550 µL dist. H_2O and cooled to 0 °C. 300 µL conc. HCl were added dropwise. Then a cooled solution of 50 mg (723 µmol, 1.70 eq) $NaNO_2$ in 330 µL H_2O was added at 0 °C. The suspension was stirred at 0 °C. After 1 h a solution of 138 mg KI (833 µmol, 2.00 eq) in 550 µL H_2O was added dropwise at 0 °C. The red-brown suspension was stirred at RT for 16 h. After full conversion of the starting material was indicated by GC-MS, the reaction was diluted with 10 mL EtOAc and washed with half-satd. $NaHSO_3$ solution (3 x 10 mL). The combined aqueous layers were reextracted with EtOAc (2 x 10 mL). The combined organic layers were dried over Na_2SO_4, filtered and the solvent was removed under reduced pressure. The brown oil was purified via flash column chromatography (10 g SiO_2, 2.0 x 6.5 cm, eluent: cyclohexane, fraction size: 3 mL).

Yield: 111 mg (77%), orange oil, $C_{10}H_{12}BrI$ [337.92 g/mol]

TLC: R_f = 0.80 (cyclohexane, UV and $KMnO_4$); **GC-MS** (Method_1): t_R = 5.91 min, m/z = 338 (100%), 296 (93%), 217 (19%), 171 (32%), 115 (23%), 89 (51%); **^{1}H-NMR** (300.36 MHz, $CDCl_3$): δ = 7.65 (d, ^3J (H,H) = 8.4 Hz, 1H, H^{Ar}), 7.28 (d, ^3J (H,H) = 2.1 Hz, 1H, H^{Ar}), 7.01 (dd, ^3J (H,H) = 8.3 Hz, ^4J (H,H) = 2.2 Hz, 1H, H^{Ar}), 2.55 (d, ^3J (H,H) = 7.2 Hz, 2H, CH_2), 1.96 (hept, ^3J (H,H) = 6.7 Hz, 1H, CH), 0.95 (d, ^3J (H,H) = 6.6 Hz, 6H, CH_3) ppm; **^{13}C-NMR** (75.53 MHz, $CDCl_3$): δ = 146.6 (C_q, C^{Ar}), 140.9 (C^{Ar}), 133.2 (C^{Ar}), 130.8 (C^{Ar}), 122.4 (C_q, C^{Ar}), 99.2 (C_q, C^{Ar}), 49.4 (CH_2), 29.1 (CH), 22.3 (CH_3) ppm; **HRMS** (DI-EI TOF): calcd. (*m/z*) for [M^+]: 337.9167; found: 337.9179.

2.1.5 4-Bromo-2-(*sec*-butyl)aniline

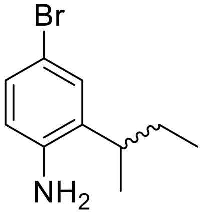

6

In a 50 mL one-neck round-bottom flask 26 mg (337 µmol, 0.10 eq) NH_4OAc were dissolved in 15 mL MeCN. 531 µL (3.35 mmol, 1.00 eq) 2-*sec*-butylaniline (**3**) and 626 mg (352 mmol, 1.05 eq) NBS were added. The red suspension was stirred at RT for 10 min. After full conversion was indicated by GC-MS the reaction was diluted with 20 mL EtOAc and washed with half-satd. $NaHSO_3$ solution (3 x 20 mL). The combined aqueous layers were reextracted with EtOAc (2 x 20 mL). The combined organic layers were dried over Na_2SO_4, filtered and the solvent was removed under reduced pressure. The red brown oil was purified via flash column chromatography (220 g SiO_2, 6.5 x 15.0 cm, eluent: cyclohexane/EtOAc = 10/1, fraction size: 70 mL).

Yield: 627 mg (82%), dark-red oil, $C_{10}H_{14}BrN$ [227.03 g/mol]

TLC: R_f = 0.27 (cyclohexane/EtOAc = 10/1, UV and $KMnO_4$); **GC-MS** (Method_1): t_R = 5.80 min, *m/z* = 227 (39%), 198 (100%), 184 (9%), 119 (32%), 91 (11%); **^{1}H-NMR** (300.36 MHz, $CDCl_3$): δ = 7.17 (d, ^3J (H,H) = 2.1 Hz, 1H, H^{Ar}), 7.09 (dd, ^3J (H,H) = 8.4, ^4J (H,H) = 2.1 Hz, 1H, H^{Ar}), 6.56 (d, ^3J (H,H) = 8.4 Hz, 1H, H^{Ar}), 3.63 (s, 2H, NH_2), 2.59 (hex, ^3J (H,H) = 6.8 Hz, 1H, CH), 1.61 (m, 2H, CH_2), 1.23 (d, ^3J (H,H) = 6.8 Hz, 3H, CH_3), 0.90 (t, ^3J (H,H) = 7.4 Hz, 3H, CH_3) ppm; **^{13}C-NMR** (75.53 MHz, $CDCl_3$): δ = 142.9 (C_q, C^{Ar}), 134.1 (C_q, C^{Ar}), 129.3 (C^{Ar}), 129.2 (C^{Ar}), 117.6 (C^{Ar}), 111.1 (C_q, C^{Ar}), 34.9 (CH), 29.5 (CH_2), 20.1 (CH_3), 12.3 (CH_3) ppm; **HRMS** (DI-EI TOF): calcd. (*m/z*) for [M^+]: 227.0310; found: 227.0311.

2.1.6 4-Bromo-2-(*sec*-butyl)-1-iodobenzene

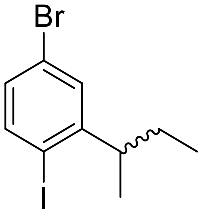

9

In a 100 mL one-neck round-bottom flask 750 mg (3.29 mmol, 1.00 eq) 4-bromo-2-(*sec*-butyl)aniline (**6**) were suspended in 3.5 mL H_2O and cooled to 0 °C. 2.0 mL conc. HCl were added dropwise. Then a cooled solution of 386 mg (5.59 mmol, 1.70 eq) $NaNO_2$ in 2.5 mL H_2O was added at 0 °C and the suspension was stirred at 0 °C. After 1 h a solution of 1.06 g (6.39 mmol, 1.94 eq) KI in 4.3 mL H_2O was added dropwise at 0 °C. The brown suspension was stirred at RT for 16 h. After full conversion of the starting material was indicated by GC-MS, the reaction was diluted with 30 mL EtOAc and washed with half-satd. $NaHSO_3$ solution (3 x 20 mL). The combined aqueous layers were reextracted with EtOAc (2 x 30 mL). The combined organic layers were dried over Na_2SO_4, filtered and the solvent was removed under reduced pressure. The brown oil was purified via flash column chromatography (100 g SiO_2, 4.0 x 12.5 cm, eluent: cyclohexane, fraction size: 60 mL).

Yield: 637 mg (73%), brown oil, $C_{10}H_{12}BrI$ [339.01 g/mol]

TLC: R_f = 0.79 (cyclohexane, UV and $KMnO_4$); **GC-MS** (Method_1): t_R = 5.88 min, *m/z* = 340 (60%), 311 (100%), 182 (40%), 102 (26%), 77 (22%); **^{1}H-NMR** (300.36 MHz, $CDCl_3$): δ = 7.46 (d, ^3J (H,H) = 8.4 Hz, 1H, H^{Ar}), 7.06 (d, ^4J (H,H) = 2.2 Hz, 1H, H^{Ar}), 6.80 (dd, ^3J (H,H) = 8.3 Hz, ^4J (H,H) = 2.2 Hz, 1H, H^{Ar}), 2.73 (hex, ^3J (H,H) = 6.9 Hz, 1H, CH), 1.37 (m, 2H, CH_2), 0.98 (d, ^3J (H,H) = 6.8 Hz, 3H, CH_3), 0.68 (t, ^3J (H,H) = 7.3 Hz, 3H, CH_3) ppm; **^{13}C-NMR** (75.53 MHz, $CDCl_3$): δ = 152.0 (C_q, C^{Ar}), 140.8 (C^{Ar}), 130.9 (C^{Ar}), 129.8 (C^{Ar}), 123.1 (C_q, C^{Ar}), 99.9 (C_q, C^{Ar}), 45.1 (CH), 30.5 (CH_2), 20.9 (CH_3), 12.1 (CH_3) ppm; **HRMS** (DI-EI TOF): calcd. (*m/z*) for [M^+]: 337.9167; found: 337.9183.

2.2 Synthesis of Asparagine and Aspartate building blocks

2.2.1 2-(2-Bromo-5-iodophenyl)acetonitrile

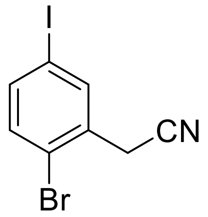

11

In a 50 mL one-neck round-bottom flask 1.00 g (3.21 mmol, 1.00 eq) 2-bromo-5-iodobenzyl alcohol (**10**) was dissolved in 10 mL DCM. 25 µL (325 µmol, 0.10 eq) DMF were added. Then 500 µL (6.95 mmol, 2.17 eq) thionylchloride were added slowly and the colorless suspension was stirred at RT. After full conversion was detected via TLC and GC-MS the solvent was removed under high vacuum using a cooling trap. The crude intermediate (2-bromo-5-iodobenzyl chloride) was used without further purification in the next reaction.
In a 25 mL one-neck round-bottom flask 500 mg (1.51 mmol, 1.00 eq) crude intermediate were dissolved in 4 mL DMSO. Then 197 mg (3.03 mmol, 2.01 eq) KCN and 26 mg (157 µmol, 0.01 eq) KI were added. The reaction was stirred at RT overnight. After full conversion (16 h) was detected via GC-MS the reaction was diluted with 15 mL H_2O and extracted with EtOAc. The phases were separated, and the organic phase was washed with H_2O (3 x 20 mL). The organic layers were dried over Na_2SO_4, filtered and the solvent was removed under reduced pressure. The slightly yellow solid was purified via flash column chromatography (40 g SiO_2, 4.0 x 9.5 cm, eluent: cyclohexane/EtOAc = 50/1 to 30/1, fraction size: 25 mL).

Yield: 345 mg (71% over 2 steps), yellowish solid, C_8H_5BrIN [320.87 g/mol]

TLC: R_f = 0.32 (cyclohexane/EtOAc = 20/1, UV and $KMnO_4$); **mp**$^{exp.}$ = 87-91 °C; **GC-MS** (Method_1): t_R = 6.37 min, *m/z* = 321 (100%), 242 (26%), 194 (30%), 167 (5%), 115 (54%); **^{1}H-NMR** (300.36 MHz, $CDCl_3$): δ = 7.84 (s, 1H, H^{Ar}), 7.54 (d, ^3J (H,H) = 8.4 Hz, 1H, H^{Ar}), 7.32 (d, ^3J (H,H) = 8.3 Hz, 1H, H^{Ar}), 3.79 (s, 2H, CH_2) ppm; **^{13}C-NMR** (75.53 MHz, $CDCl_3$, APT): δ = 139.2 (C^{Ar}), 138.5 (C^{Ar}), 134.7 (C^{Ar}), 132.2 (C_q, C^{Ar}), 123.5 (C_q, C^{Ar}), 116.4 (C_q, CN), 93.0 (C_q, C^{Ar}), 24.6 (CH_2) ppm; **HRMS** (DI-EI TOF): calcd. (*m/z*) for [M^+]: 320.8650; found: 320.8663.

2.2.2 2-(2-Bromo-5-iodophenyl)acetamide

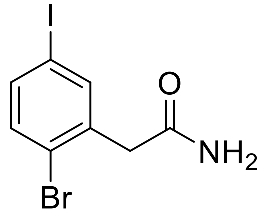

12

In a 50 mL one-neck round-bottom flask 739 mg (2.30 mmol, 1.00 eq) 2-(2-bromo-5-iodophenyl)acetonitrile (**11**) were suspended in 13 mL *tert*-butanol. Then 515 mg (9.18 mmol, 3.99

eq) finely powdered KOH were added. The yellow suspension was stirred at 80 °C. After full conversion of the starting material was indicated by TLC, the reaction was quenched by the addition of 50 mL dist. H_2O. The aqueous phase was extracted with $CHCl_3$ (3 x 50 mL). The combined organic layers were dried over Na_2SO_4, filtered and the solvent was removed under reduced pressure. The brown solid was purified via flash column chromatography (60 g SiO_2, 3.0 x 23.0 cm, eluent: cyclohexane/EtOAc = 1/1, fraction size: 70 mL).

Yield: 395 mg (51%), colorless solid, C_8H_7BrINO [339.96 g/mol]

TLC: R_f = 0.20 (cyclohexane/EtOAc = 1/1, UV and CAM); **mp**$^{exp.}$ = 176-182 °C; **^{1}H-NMR** (300.36 MHz, [D6]DMSO): δ = 7.71 (d, ^4J (H,H) = 1.7 Hz, 1H, H^{Ar}), 7.51 (dd, ^3J (H,H) = 8.1 Hz, ^4J (H,H) = 1.8 Hz, 1H, H^{Ar}), 7.36 (d, ^3J (H,H) = 8.3 Hz, 1H, H^{Ar}), 7.01 (s, 2H, NH_2), 3.54 (s, 2H, CH_2) ppm; **^{13}C-NMR** (75.53 MHz, [D6]DMSO, APT): δ = 170.4 (C_q, CO), 140.3 (C^{Ar}), 138.7 (C_q, C^{Ar}), 137.1 (C^{Ar}), 134.1 (C^{Ar}), 124.5 (C_q, C^{Ar}), 93.2 (C_q, C^{Ar}), 41.5 (CH_2) ppm; **HRMS** (DI-EI TOF): calcd. (*m/z*) for [M^+-H_2O]: 320.8650; found: 320.8563.

2.2.3 Methyl 2-(2-bromo-5-iodophenyl)acetate

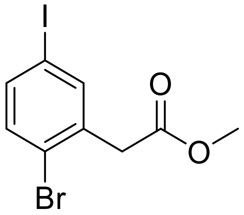

13

In a 50 mL one-neck round-bottom flask 200 mg (621 µmol, 1.00 eq) 2-(2-bromo-5-iodophenyl)acetonitrile (**11**) were suspended in 6 mL MeOH. The yellowish suspension was cooled to 0 °C and then 0.5 mL (9.38 mmol, 15.1 eq) conc. H_2SO_4 were added. The reaction was stirred at 65 °C. After full conversion (48 h) was detected via TLC the reaction was diluted with 20 mL dist. H_2O and extracted with EtOAc (3 x 20 mL). The combined organic layers were dried over Na_2SO_4, filtered and the solvent was removed under reduced pressure. The light-yellow solid was purified via flash column chromatography (25 g SiO_2, 2.5 x 9.0 cm, eluent: cyclohexane/EtOAc = 100/1, fraction size: 15 mL).

Yield: 73.0 mg (33%), colorless solid, $C_9H_8BrIO_2$ [354.97 g/mol]

TLC: R_f = 0.32 (cyclohexane/EtOAc = 20/1, UV and CAM); **mp**$^{exp.}$ = 42-46 °C; **GC-MS** (Method_1): t_R = 6.40 min, *m/z* = 356 (8%), 323 (4%), 297 (20%), 275 (100%), 207 (6%), 89 (24%); **^{1}H-NMR** (300.36 MHz, $CDCl_3$): δ = 7.61 (d, ^4J (H,H) = 1.6 Hz, 1H, H^{Ar}), 7.45 (dd, ^3J (H,H) = 8.3 Hz, ^4J (H,H) = 1.7 Hz, 1H, H^{Ar}), 7.29 (d, ^3J (H,H) = 8.4 Hz, 1H, H^{Ar}), 3.73 (s, 5H, CH_2 & CH_3) ppm; **^{13}C-NMR** (75.53 MHz, $CDCl_3$): δ = 170.4 (C_q, CO), 140.3 (C^{Ar}), 138.0 (C^{Ar}), 136.5 (C_q, C^{Ar}), 134.5 (C^{Ar}), 125.1 (C_q, C^{Ar}), 92.5 (C_q, C^{Ar}), 52.5 (CH_2), 41.2 (CH_3) ppm; **HRMS** (DI-EI TOF): calcd. (*m/*z) for [M^+]: 353.8752; found: 353.8766.

2.3 Synthesis of Cysteine building block

2.3.1 1-Bromo-2-(bromomethyl)-4-iodobenzene

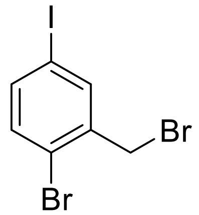

14

In a flame dried 10 mL Schlenk-flask 200 mg (639 µmol, 1.00 eq) 2-bromo-5-iodobenzyl alcohol (**10**) were suspended in 5 mL DCM. Then 10 µL DMF and 70 µL (906 µmol, 1.42 eq) thionylbromide were added and the yellow/orange solution was stirred at RT. After full conversion (90 min) was indicated by TLC and GC-MS, 20 mL DCM were added, and the organic phase was washed with satd. $NaHCO_3$ solution (2 x 30 mL). Then the organic phase was dried over Na_2SO_4, filtered and the solvent was removed under reduced pressure. The colorless crude product was purified via flash column chromatography (30 g SiO_2, 2.5 x 9.5 cm, eluent: cyclohexane, fraction size: 20 mL).

Yield: 227 mg (94%), colorless solid $C_7H_5Br_2I$ [375.83 g/mol]

TLC: R_f = 0.56 (cyclohexane, UV and $KMnO_4$); **mp**$^{exp.}$ = 105-108 °C (**mp**$^{lit.}$ = 112-114 °C)[4]; **GC-MS** (Method_1): t_R = 6.31 min, *m/z* = 376 (21%), 297 (100%), 168 (20%), 127 (12%), 89 (52%); **^{1}H-NMR** (300.36 MHz, $CDCl_3$): δ = 7.77 (d, ^4J (H,H) = 1.8 Hz, 1H, H^{Ar}), 7.47 (dd, ^3J (H,H) = 8.4 Hz, ^4J (H,H) = 1.8 Hz, 1H, H^{Ar}), 7.29 (d, ^3J (H,H) = 8.4 Hz, 1H, H^{Ar}), 4.50 (s, 2H,

CH_2) ppm; **^{13}C-NMR** (75.53 MHz, $CDCl_3$): δ = 140.0 (C^{Ar}), 139.3 (C_q, C^{Ar}), 139.1 (C^{Ar}), 135.0 (C^{Ar}), 124.4 (C_q, C^{Ar}), 92.6 (C_q, C^{Ar}), 32.1 (CH_2) ppm.

Analytical data are in accordance with those reported.[4]

2.3.2 (2-Bromo-5-iodobenzyl) ethanethioate

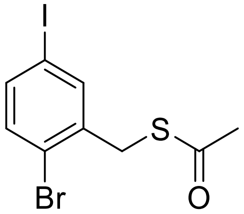

15

In an 8 mL Schlenk-flask 200 mg (532 µmol, 1.00 eq) 1-bromo-2-(bromomethyl)-4-iodobenzene (**14**) were dissolved in 2 mL abs THF. Then 162 mg (1.17 mmol, 2.20 eq) K_2CO_3 were added. Afterwards 50 µL (700 µmol, 1.31 eq) thioacetic acid were added via syringe over 1 min. The yellow suspension was stirred at RT. After full conversion of the starting material was indicated by GC-MS (2 h), the reaction was quenched by addition of 1 mL 1M HCl. Then 20 mL H_2O were added, and the aqueous phase was extracted with DCM (3 x 30 mL). The combined organic layers were dried over Na_2SO_4, filtered and the solvent was removed under reduced pressure. The yellow solid was purified via flash column chromatography (25 g SiO_2, 2.5 x 8.5 cm, eluent: cyclohexane/toluene = 4/1, fraction size: 8 mL).

Yield: 123 mg (62%), colorless solid, C_9H_8BrIOS [371.03 g/mol]

TLC: R_f = 0.72 (toluene, UV and KMnO4); **mp**$^{exp.}$ = 65-71 °C; **GC-MS** (Method_1): t_R = 6.89 min, *m/z* = 372 (2%), 330 (3%), 291 (100%), 248 (5%), 89 (7%); **^{1}H-NMR** (300.36 MHz, $CDCl_3$): δ = 7.75 (d, ^4J (H,H) = 1.8 Hz, 1H, H^{Ar}), 7.41 (dd, ^3J (H,H) = 8.3 Hz, ^4J (H,H) = 1.9 Hz, 1H, H^{Ar}), 7.24 (d, ^3J (H,H) = 6.8 Hz, 1H, H^{Ar}), 4.14 (s, 2H, CH_2), 2.35 (s, 3H, CH_3) ppm; **^{13}C-NMR** (75.53 MHz, $CDCl_3$): δ = 194.6 (C_q, CO), 139.9 (C^{Ar}), 139.6 (C_q, C^{Ar}), 138.1 (C^{Ar}), 134.5 (C^{Ar}), 124.5 (C_q, C^{Ar}), 92.6 (C_q, C^{Ar}), 33.5 (CH_2), 30.5 (CH_3) ppm; **HRMS** (DI-EI TOF): calcd. (*m/*z) for [M^+]: 369.8524; found: 369.8538.

2.4 Synthesis of Threonine building block

2.4.1 2-Bromo-5-iodobenzaldehyde

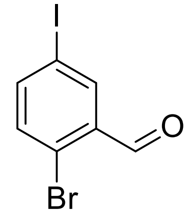

16

In a 250 mL flame dried round-bottom flask equipped with a Schlenk adapter 2.40 g (7.67 mmol, 1.00 eq) (2-bromo-5-iodophenyl)-methanol (**10**) were dissolved in 75 mL DCM. 1.5 g anhydrous 4 Å MS and 4.0 g (46.0 mmol, 5.99 eq) MnO_2 were added under inert atmosphere. The black reaction mixture was stirred overnight. After full conversion of the starting material was indicated by TLC and GC-MS (14 h) MnO_2 was removed via filtration through a pad of silica (20g SiO_2, eluent: EtOAc, fraction size: 50 mL). The filtrate was collected, and the solvent was removed under reduced pressure. The crude product was used without further purification.

Yield: 2.14 g (90%), colorless solid, C_7H_4BrIO [309.85 g/mol]

TLC: R_f = 0.67 (cyclohexane/EtOAc = 3/1, UV and CAM); **mp**$^{exp.}$ = 103-107 °C (**mp**$^{lit.}$ = 112-114 °C)[5]; **GC-MS** (Method_1): t_R = 5.83 min, *m/z* = 310 (100%), 283 (12%), 202 (6%), 157 (8%), 127 (10%), 75 (34%); **^{1}H-NMR** (300.36 MHz, $CDCl_3$): δ = 10.24 (s, 1H, COH), 8.19 (d, ^4J (H,H) = 2.1 Hz, 1H, H^{Ar}), 7.74 (dd, ^3J (H,H) = 8.3, ^4J (H,H) = 2.1 Hz, 1H, H^{Ar}), 7.38 (d, ^3J (H,H) = 8.4 Hz, 1H, H^{Ar}) ppm; **^{13}C-NMR** (75.53 MHz, $CDCl_3$): δ = 190.5 (CO), 144.0 (C^{Ar}), 138.8 (C_q, C^{Ar}), 135.6 (C^{Ar}), 134.9 (C^{Ar}), 126.7 (C_q, C^{Ar}), 93.0 (C_q, C^{Ar}) ppm.

Analytical data are in accordance with those reported.[5]

2.4.2 1-(2-Bromo-5-iodophenyl)ethan-1-ol

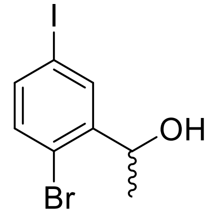

17

In a 50 mL Schlenk-flask 1.00 g (3.22 mmol, 1.00 eq) 2-bromo-5-iodobenzaldehyde (**16**) was dissolved in 13 mL Et_2O. Then 1.90 mL (3.70 mmol, 1.70 eq) MeMgBr solution (3M in Et_2O) were slowly added. The colorless suspension was stirred at RT. After full conversion of the starting material was indicated by TLC (14 h), the reaction was washed with 1M HCl (1 x 50 mL). Then the organic phase was extracted with satd. $NaHCO_3$ solution (1 x 50 mL). The organic layer was dried over Na_2SO_4, filtered and the solvent was removed under reduced pressure. The colorless solid was purified via flash column chromatography (100 g SiO_2, 5.0 x 14.0 cm, eluent: cyclohexane/EtOAc = 10/1, fraction size: 50 mL).

Yield: 873 mg (83%), colorless solid, C_8H_8BrIO [326.96 g/mol]

TLC: R_f = 0.32 (cyclohexane/EtOAc = 10/1, UV and CAM); **mp**$^{exp.}$ = 64-68 °C; **^{1}H-NMR** (300.36 MHz, $CDCl_3$): δ = 7.91 (d, ^4J (H,H) = 1.8 Hz, 1H, H^{Ar}), 7.43 (dd, ^3J (H,H) = 8.3, ^4J (H,H) = 1.9 Hz, 1H, H^{Ar}), 7.22 (d, ^3J (H,H) = 8.3 Hz, 1H, H^{Ar}), 5.14 (q, ^3J (H,H) = 6.3 Hz, 1H, CH), 2.04 (bs, 1H, OH), 1.46 (d, ^3J (H,H) = 6.3 Hz, 3H, CH_3) ppm; **^{13}C-NMR** (75.53 MHz, $CDCl_3$): δ = 147.0 (C_q, C^{Ar}), 137.82 (C^{Ar}), 135.99 (C^{Ar}), 134.38 (C^{Ar}), 121.47 (C_q, C^{Ar}), 93.28 (C_q, C^{Ar}), 69.01 (CH), 23.77 (CH_3) ppm; **HRMS** (DI-EI TOF): calcd. *(m/z)* for [M^+]: 325.8803; found: 325.8805.

2.5 Synthesis of Methionine, Arginine, Glutamine and Glutamate building blocks

2.5.1 ((Methylthio)methyl)triphenylphosphonium chloride

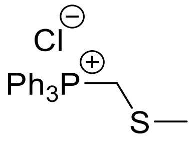

18

In a flame dried 250 mL Schlenk-flask 20.01 g (76.3 mmol, 1.00 eq) triphenylphosphine were dissolved in 40 mL toluene. Then 6.40 mL (77.5 mmol, 1.02 eq) chloromethyl-methylsulfide were added via a syringe. The colorless solution was stirred at 100 °C and after 2 h a colorless precipitate was formed. After 24 h the colorless suspension was cooled to 0 °C and the precipitate was collected by filtration and washed with cold toluene (3 x 15 mL). After drying the filter cake in vacuum, the product was isolated as colorless powder.

Yield: 17.04 g (92%), colorless powder, $C_{20}H_{20}ClPS$ [358.86 g/mol]

$mp^{exp.}$ = 209-217 °C (mp^{lit} = 220-222 °C)[6]; **^{1}H-NMR** (300.36 MHz, $CDCl_3$): δ = 7.93 – 7.87 (m, 6H, H^{Ar}), 7.74 – 7.62 (m, 9H, H^{Ar}), 5.28 (d, ^{2}J (H,P) = 8.3 Hz, 2H, CH_2), 2.12 (s, 3H, CH_3) ppm; **^{13}C-NMR** (75.53 MHz, $CDCl_3$): δ = 135.0 (d, ^{4}J (C,P) = 2.9 Hz, C^{Ar}), 134.2 (d, ^{3}J (C,P) = 9.9 Hz, C^{Ar}), 130.2 (d, ^{2}J (C,P) = 12.6 Hz, C^{Ar}), 118.2 (d, ^{1}J (C,P) = 86.9 Hz, C^{Ar}), 25.8 (d, ^{1}J (C,P) = 51.8 Hz, CH_2), 18.1 (d, ^{3}J (C,P) = 3.2 Hz, CH_3) ppm.

Analytical data are in accordance with those reported.[7]

2.5.2 (2-Bromo-5-iodostyryl)(methyl)sulfane

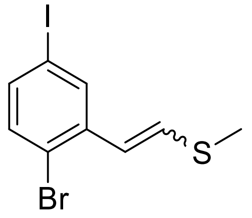

10

In a flame dried 100 mL Schlenk-tube 1.30 g (3.62 mmol, 1.50 eq) phosphonium-salt **18** were suspended in 15 mL abs. THF. Then the colorless suspension was cooled to -78 °C in a dry ice/acetone bath. Afterwards 1.5 mL (2.5M in hexane, 3.75 mmol, 1.50 eq) *n*-BuLi were added dropwise via a syringe. The dark red suspension was stirred at -78 °C for 1 h. In the meanwhile, in another flame dried Schlenk-flask 751 mg (2.42 mmol, 1.00 eq) 2-bromo-5-iodobenzaldehyde (**16**) were dissolved in 5 mL abs. THF. Then the aldehyde solution was added via syringe to the ylide at -78 °C. After 20 min the reaction mixture became a light orange suspension and was stirred overnight (16 h) at RT until complete conversion was indicated by TLC. The orange suspension was quenched by the addition of 20 mL satd. NH_4Cl-solution. The phases were separated, and the aqueous phase was extracted with DCM (3 x 20 mL). The combined organic layers were dried over Na_2SO_4, filtered, and concentrated to dryness under reduced pressure. The yellow crude product was purified via column chromatography (125 g SiO_2, 4.5 x 8.5 cm, eluent: cyclohexane, fraction size: 50 mL).

Yield: 646 mg (75%), *E/Z* mixture = 1/1.4, colorless solid, C_9H_8BrIS [355.03 g/mol]

TLC: R_f = 0.58 and 0.62 (cyclohexane, UV and $KMnO_4$); **$mp^{exp.}$** = 35-40 °C; **GC-MS** (Method_1): t_{R1} = 6.89 min, t_{R2} = 7.04 min, *m/z* = 354 (15%), 275 (100%), 212 (8%), 148 (65%), 89 (19%); **^{1}H-**

NMR (300.36 MHz, $CDCl_3$): δ = 7.98 (d, ^4J (H,H) = 1.9 Hz, 0.7H, H^{Ar}), 7.73 (d, ^4J (H,H) = 1.9 Hz, 1H, H^{Ar}), 7.40 – 7.22 (m, 4H, H^{Ar}), 6.82 (d, ^3J (H,H) = 15.3 Hz, 1H, CH), 6.54 – 6.40 (m, 2.5H, H^{Ar} & CH), 2.42 (s, 3H, CH_3), 2.40 (s, 3H, CH_3) ppm; **^{13}C-NMR** (75.53 MHz, $CDCl_3$): δ = 139.1 (C_q, C^{Ar}), 138.4 (C_q, C^{Ar}), 138.2 C^{Ar}), 137.1 C^{Ar}), 136.6 (C^{Ar}), 135.0 (C^{Ar}), 134.6 (C^{Ar}), 134.4 (C^{Ar}), 133.1 (CH), 131.0 (CH), 123.7 (C_q, C^{Ar}), 123.1 (CH), 122.3 (C_q, C^{Ar}), 121.5 (CH), 92.8 (C_q, C^{Ar}), 92.1 (C_q, C^{Ar}), 18.6 (CH_3), 14.9 (CH_3) ppm; **HRMS** (DI-EI TOF): calcd. *(m/z)* for [M^+]: 353.8575; found: 353.8580.

2.5.3 (2-Bromo-5-iodophenethyl)(methyl)sulfane

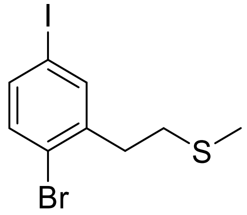

20

In a 10 mL one-neck round-bottom flask 131 mg (368 µmol, 1.00 eq) (2-bromo-5-iodostyryl)(methyl)sulfane (**19**) were dissolved in 3 mL THF. Then 685 mg (3.68 mmol, 10.0 eq) p-tosylhydrazide, 301 mg (2.21 mmol, 6.00 eq) $NaOAc \cdot 3H_2O$ were added. The yellowish suspension was stirred at 70 °C until 83% conversion was achieved (6 d). The orange suspension was distributed between satd. $NaHCO_3$-solution and DCM. The phases were separated and the aqueous phase was extracted with DCM (2 x 10 mL). The combined organic layers were dried over Na_2SO_4, filtered and concentrated to dryness under reduced pressure. The yellow crude product was purified via flash column chromatography (25 g SiO_2, 2.5 x 8.5 cm, eluent: cyclohexane/EtOAc = 100:1, fraction size: 8 mL). Another flash column chromatography for purification had to be performed (13 g SiO_2, 2.0 x 7.5 cm, eluent: cyclohexane/toluene = 100:1, fraction size: 5 mL) to produce pure product.

Yield: 57.2 mg (44%), colorless solid, $C_9H_{10}BrIS$ [357.05 g/mol]

TLC: R_f = 0.48 (cyclohexane/toluene = 100/1, UV and $KMnO_4$); **mp**$^{exp.}$ = 43-45 °C; **^{1}H-NMR** (300.36 MHz, $CDCl_3$): δ = 7.57 (d, ^4J (H,H) = 1.7 Hz, 1H, H^{Ar}), 7.39 (dd, ^3J (H,H) = 8.4 Hz, ^4J (H,H) = 1.7 Hz, 1H, H^{Ar}), 7.24 (d, ^3J (H,H) = 8.4 Hz, 1H, H^{Ar}), 3.00 – 2.91 (m, 2H, CH_2), 2.73 (m, 2H, CH_2), 2.16 (s, 3H, CH_3) ppm; **^{13}C-NMR** (75.53 MHz, $CDCl_3$): δ = 142.2 (C_q, C^{Ar}), 139.5 (C^{Ar}),

137.2 (C^{Ar}), 134.6 (C^{Ar}), 124.3 (C_q, C^{Ar}), 92.7 (C_q, C^{Ar}), 36.1 (CH_2), 33.8 (CH_2), 15.8 (CH_3) ppm, **HRMS** (DI-EI TOF): calcd. *(m/z)* for [M^+]: 355.8731; found: 355.8674.

2.5.4 (Cyanomethyl)triphenylphosphonium chloride

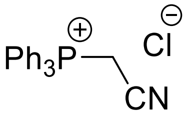

21

In a flame dried and argon flushed Schlenk-flask 9.00 g PPh_3 (34.4 mmol, 1.00 eq) were dissolved in 50 mL absolute, degassed toluene. 4.35 mL 2-Chloroacetonitrile (58.6 mmol, 2.0 eq) were added to the colourless solution. The reaction mixture was heated under reflux for 24 h during which a colourless precipitate was formed. The suspension was cooled to RT and the precipitate was collected by filtration and washed with Et_2O (2 x 10 mL).

Yield: 11.5 g (99%), colourless powder, $C_{20}H_{17}ClNP$ [337.78 g/mol].

mp$^{exp.}$ = 263 °C (decomposition); **^{1}H-NMR** (300 MHz, [D6]DMSO): δ = 8.01 – 7.81 (m, 15H, H_{Ar}), 6.32 (d, ^2J (H,P) = 15.9 Hz, 2H; CH_2) ppm; **^{13}C-NMR** (76 MHz, [D6]DMSO, APT): δ = 135.9 (d, ^4J (C,P) = 3 Hz, C_{Ar}), 133.8 (d, ^3J (C,P) = 11 Hz, C_{Ar}), 130.5 (d, ^2J (C,P) = 13 Hz, C_{Ar}), 116.3 (d, ^1J (C,P) = 89 Hz, C_{Ar}), 113.0 (d, ^2J (C,P) = 9 Hz, CN), 14.4 (d, ^1J (C,P) = 55 Hz, CH_2) ppm.

Analytical data are in accordance with those reported.[8]

2.5.5 3-(2-Bromo-5-iodophenyl)acrylonitrile

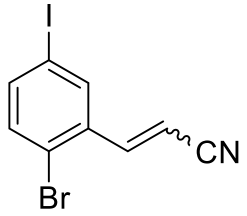

22

In a flame dried and N_2 flushed 250 mL Schlenk-flask 1.86 g (5.51 mmol, 1.10 eq) (cyanomethyl)triphenylphosphonium chloride **(21)** and 620 mg (5.51 mmol, 1.10 eq) KO*t*Bu were suspended in 30 mL abs. THF. The suspension was stirred for 10 min at RT and 1.57 g (5.04 mmol, 1.00 eq) 2-bromo-5-iodobenzaldehyde (**16**) were added. The reaction mixture was stirred at 50 °C

for 16 h. The solvent was removed under reduced pressure and the residue was dissolved in 50 mL DCM and washed with water (40 mL). The organic phase was dried over Na_2SO_4, filtered and the solvent was removed under reduced pressure. The crude product was purified via column chromatography (50 g SiO_2, 3.5 x 12 cm, eluent: cyclohexane/AcOEt = 10/1).

Yield: 1.47 g (87%), *E/Z* mixture = 1/1, colorless powder, C_9H_5BrIN [333.95 g/mol].

TLC: R_f = 0.22 (cyclohexane/EtOAc = 20/1, UV); **GC-MS** (Method_1): $t_{R(E)}$ = 6.56 min, $t_{R(Z)}$ = 6.77 min, *m/z* = 335 (97%), 333 (95%), 254 (66%), 127 (100%); **^{1}H-NMR** (300.36 MHz, $CDCl_3$): δ = 8.24 (d, ^4J (H,H) = 1.7 Hz, 1H, H^{Ar}), 7.81 (d, ^4J (H,H) = 1.7 Hz, 1H, H^{Ar}), 7.67 (d, ^3J (H,H) = 16.6 Hz, 1H, CH), 7.63 – 7.51 (m, 2H, H^{Ar}), 7.42 – 7.30 (m, 3H, H^{Ar} & CH), 5.86 (d, ^3J (H,H) = 16.6 Hz, 1H, CH), 5.64 (d, ^3J (H,H) = 11.9 Hz, 1H, CH) ppm; **^{13}C-NMR** (75.53 MHz, $CDCl_3$): δ = 147.7 (CH), 146.5 (CH), 140.9 (C^{Ar}), 140.7 (C^{Ar}), 137.9 (C^{Ar}), 135.9 (C^{Ar}), 135.7 (C_q, C^{Ar}), 135.6 (C_q, C^{Ar}), 135.2 (C^{Ar}), 134.7 (C^{Ar}), 124.5 (C_q, C^{Ar}), 124.2 (C_q, C^{Ar}), 117.2 (C_q, CN), 115.9 (C_q, CN), 100.6 (CH), 99.9 (CH), 92.8 (C_q, C^{Ar}), 92.6 (C_q, C^{Ar}) ppm; **HRMS** (DI-EI) calcd. *(m/z)* for [M^+]: 332.8650; found: 332.8648.

2.5.6 3-(2-Bromo-5-iodophenyl)propanenitrile

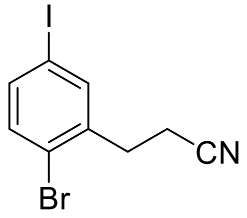

23

In a 250 mL round-bottom flask 1.39 g (4.19 mmol, 1.00 eq) 3-(2-bromo-5-iodophenyl)acrylonitrile (**22**), 6.25 g p-tosyl hydrazide (33.6 mmol, 8.00 eq) and 4.56 g NaOAc·3H_2O (33.6 mmol, 8.00 eq) were suspended in 70 mL THF. The suspension was heated under reflux for 14 h. The reaction mixture was cooled to RT and the solvent was removed under reduced pressure. The residue was diluted with 100 mL H_2O and extracted with DCM (2 x 100 mL). The organic phase was dried over Na_2SO_4 and the solvent was removed under reduced pressure. The crude product was purified via column chromatography (50 g SiO_2, 3.5 x 12 cm, eluent: cyclohexane/AcOEt = 30/1 → 20/1).

Yield: 1.38 g (98%), colorless powder, C_9H_7BrIN [335.97 g/mol].

TLC: Rf = 0.09 (cyclohexane/EtOAc = 20/1, UV); **mp$^{exp.}$** = 72-73 °C; **GC-MS** (Method_1): t_R = 6.66 min; *m/z* = 337 (66%), 335 (68%), 297 (97%), 295 (100%); **^{1}H-NMR** (300.36 MHz, $CDCl_3$): δ = 7.62 (d, ^4J (H,H) = 1.8 Hz, 1H, H^{Ar}), 7.46 (dd, ^3J (H,H) = 8.3, ^4J (H,H) = 2.0 Hz, 1H, H^{Ar}), 7.28 (d, ^3J (H,H) = 8.4 Hz, 1H, H^{Ar}), 3.03 (t, ^3J (H,H) = 7.4 Hz, 2H, CH_2), 2.67 (t, ^3J (H,H) = 7.4 Hz, 2H, CH_2) ppm; **^{13}C-NMR** (76 MHz, $CDCl_3$): δ = 139.6 (C_q, C^{Ar}), 139.5 (C^{Ar}), 138.3 (C^{Ar}), 134.9 (C^{Ar}), 124.0 (C_q, C^{Ar}), 118.5 (C_q, CN), 92.9 (C_q, C^{Ar}), 31.9 (CH_2), 17.5 (CH_2) ppm; **HRMS** (DI-EI) calcd (*m/z*) for [M^+]: 334.8807; found: 334.8817.

2.5.7 (2-Amino-2-oxoethyl)triphenylphosphonium chloride

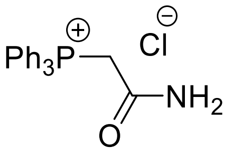

24

In a flame dried Schlenk-flask 7.86 g PPh_3 (30.0 mmol, 1.10 eq) and 2.67 g 2-chloroacetamide (28.6 mmol, 1.00 eq) were suspended in 30 mL nitromethane. The mixture was stirred for 19 h at 105 °C. The pale brown solution was allowed to cool to RT, and the formed colourless precipitate was isolated by filtration, washed with EtOAc (2x 10 mL), Et_2O (1x 15 mL) and dried in vacuum.

Yield: 10.1 g (99%), colourless powder, $C_{20}H_{19}ClNOP$ [355.80 g/mol].

mp$^{exp.}$ = 219-221 °C; **^{1}H NMR** (300 MHz, [D6]DMSO): δ = 8.43 (bs, 1H; $CONH_2$), 7.89 – 7.74 (m, 15H; H^{Ar}), 7.62 (bs, 1H; $CONH_2$), 5.12 (d, ^2J (H,P) = 14.8 Hz, 2H; CH_2) ppm; **^{13}C NMR** (76 MHz, [D6]DMSO): δ = 165.0 (d, ^2J (C,P) = 5 Hz; $CONH_2$), 134.7 (d, ^4J (C,P) = 3 Hz; C^{Ar}), 133.8 (d, ^3J (C,P) = 11 Hz; C^{Ar}), 129.9 (d, ^2J (C,P) = 13 Hz; C^{Ar}), 119.1 (d, ^1J (C,P) = 89 Hz; C^{Ar}), 31.2 (d, ^1J (C,P) = 59 Hz; CH_2) ppm.

Analytical data are in accordance with those reported.[8]

2.5.8 (*E*)-3-(2-Bromo-5-iodophenyl)acrylamide

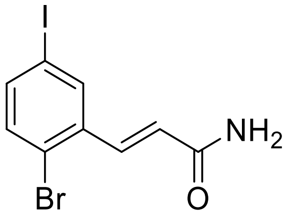

25

In a flame dried and N_2 flushed Schlenk-flask 3.36 g (9.45 mmol, 1.05 eq) (2-amino-2-oxoethyl)triphenylphosphonium chloride (**24**) and 1.06 g (9.45 mmol, 1.05 eq) KO*t*Bu were suspended in 50 mL absolute MeOH. The suspension was stirred for 10 min at RT and 2.80 g (9.00 mmol, 1.00 eq) 2-bromo-5-iodobenzaldehyde (**16**) were added. The reaction mixture was stirred at 50 °C until full conversion of the starting material was observed. When quantitative conversion was detected via TLC (16 h) the solvent was removed under reduced pressure and crude product was purified via column chromatography (125 g SiO_2, 7.5 x 11 cm, eluent: cyclohexane/AcOEt = 5/4 → 1/1).

Yield: 3.08 g (97%), colorless powder, C_9H_7BrINO [351.97 g/mol]

TLC: R_f = 0.35 (cyclohexane/EtOAc = 1/2, UV); **mp**$^{exp.}$ = 213-214 °C; **HPLC-MS** (Poroshell, ESI+, MV_general): t_R = 5.89 min, *m/z* = 452 [$M+H^+$], 454 [$M+H^+$]; **^{1}H-NMR** (300.36 MHz, [D6]DMSO): δ = 7.82 (d, ^4J (H,H) = 1.3 Hz, 1 H, H^{Ar}), 7.60 (bs, 1 H, NH), 7.55 (dd, ^3J (H,H) = 8.3 Hz, ^4J (H,H) = 1.5 Hz, 1H, H^{Ar}), 7.40 (d, ^3J (H,H) = 8.3 Hz, 1 H, H^{Ar}), 7.17 (bs, 1 H, NH), 6.67 (d, ^3J (H,H) = 12.2 Hz, 1 H, CH), 6.17 (d, ^3J (H,H) = 12.2 Hz, 1 H, CH) ppm; **^{13}C-NMR** (75.53 MHz, [D6]DMSO, APT): δ = 166.3 (C_q, CO), 138.9 (C^{Ar}), 138.3 (C_q, C^{Ar}), 137.9 (C^{Ar}), 134.5 (C^{Ar}), 133.8 (CH), 127.0 (CH), 122.5 (C_q, C^{Ar}), 92.5 (C_q, C^{Ar}) ppm; **HRMS** (DI-EI) calcd *(m/z)* for [M^+]: 352.8736; found: 352.8749.

2.5.9 3-(2-Bromo-5-iodophenyl)propanamide

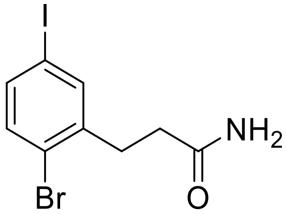

27

In a 250 mL round-bottom flask equipped with a reflux condenser 3.08 g (8.75 mmol, 1.00 eq) 3-(2-bromo-5-iodophenyl)propanamide (**26**) and 5.09 g (26.3 mmol, 3.00 eq) PADA (**26**)[9] were suspended in 75 mL 1,2-DME. 1.05 mL (26.3 mmol, 3.00 eq) AcOH were added to the yellow suspension. The reaction mixture was stirred at 50 °C for 16 h. The reaction mixture was cooled to RT and filtered via a glass frit. The filter cake was rinsed with 50 mL 1,2-DME. The filtrate was concentrated in vacuum and the residue was triturated with 40 mL DCM/Et_2O (1/1). The remaining solid was collected by filtration and dried in vacuum.

Yield: 1.85 g (60%), colorless powder, C_9H_9BrINO [353.99 g/mol]

mp$^{exp.}$ = 146-149 °C; **HPLC-MS** (Poroshell, ESI^+, MV_general): t_R = 5.74 min; *m/z* = 454 [M+H^+], 456 [M+H^+]; **^{1}H-NMR** (300.36 MHz, [D6]DMSO): δ = 7.67 (d, ^4J (H,H) = 1.8 Hz, 1 H, H^{Ar}), 7.48 (dd, ^3J (H,H) = 8.3 Hz, ^4J (H,H) = 2.0 Hz, 1 H, H^{Ar}), 7.42 – 7.25 (m, 2 H, H^{Ar} & NH), 6.84 (s, 1 H, NH), 2.84 (t, ^3J (H,H) = 7.6 Hz, 2 H, CH_2), 2.35 (t, ^3J (H,H) = 7.7 Hz, 2 H, CH_2) ppm; **^{13}C-NMR** (75.53 MHz, [D6]DMSO, APT): δ = 172.8 (C_q, CO), 143.0 (C_q, C^{Ar}), 138.8 (C^{Ar}), 136.8 (C^{Ar}), 134.4 (C^{Ar}), 123.7 (C_q, C^{Ar}), 93.6 (C_q, C^{Ar}), 34.4 (CH_2), 30.8 (CH_2) ppm; **HRMS** (DI-EI) calcd *(m/z)* for [M^+–H_2O]: 336.8787; found: 336.8802.

2.5.10 Ethyl 3-(2-bromo-5-iodophenyl)acrylate

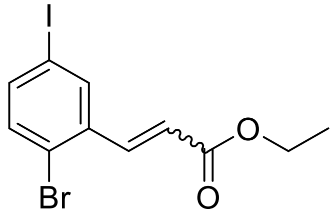

29

In a flame dried Schlenk-flask 4.98 g (11.6 mmol, 1.23 eq) (2-ethoxy-2-oxoethyl)triphenylphosphonium bromide **28** and 1.42 g (12.8 mmol, 1.34 eq) KO*t*Bu were dried in vacuum for 1 h. Then 40 mL abs., degassed THF were added. The yellowish suspension was stirred at RT for 1 h. Then 2.94 g (9.49 mmol, 1.00 eq) 2-bromo-5-iodobenzaldehyde (**16**) were added to the ylide solution. The orange/yellow suspension was stirred overnight (16 h) at 50 °C until complete conversion was detected via TLC. The solvent was removed under reduced pressure. The yellow-brown crude product was purified via flash column chromatography (500 g SiO2, 5.5 x 19.0 cm, eluent: cyclohexane/EtOAc = 75/1 → 50/1, fraction size: 50 mL).

Yield: 3.38 g (94%), *E/Z* mixture = 4/1, colorless solid, $C_{11}H_{10}BrIO_2$ [379.89 g/mol]

TLC: R_f = 0.53 (cyclohexane/EtOAc = 10/1, UV and $KMnO_4$); **mp**$^{exp.}$ = 65-72 °C; **^{1}H-NMR** (300.36 MHz, $CDCl_3$): δ = 7.92 – 7.87 (m, 2H, H^{Ar} & CH), 7.74 (d, ^4J (H,H) = 1.2 Hz, 0.2H, H^{Ar}), 7.51 (dd, ^3J (H,H) = 8.4 Hz, ^4J (H,H) = 1.8 Hz, 1H, H^{Ar}), 7.46 (d, ^4J (H,H) = 1.6 Hz, 0.1H, H^{Ar}), 7.32 (d, ^3J (H,H) = 8.4 Hz, 1H, H^{Ar}), 7.27 (s, 0.1H, H^{Ar}), 6.96 (d, ^3J (H,H) = 12.1 Hz, 0.2H, CH), 6.37 (d, ^3J (H,H) = 15.9 Hz, 1H, CH), 6.07 (d, ^3J (H,H) = 12.1 Hz, 0.2H, CH), 4.28 (q, ^3J (H,H) = 7.1 Hz, 2H, CH_2), 4.12 (q, ^3J (H,H) = 7.1 Hz, 0.5H, CH_2), 1.34 (t, ^3J (H,H) = 7.1 Hz, 3H, CH_3), 1.19 (t, ^3J (H,H) = 7.1 Hz, 0.7H, CH_3) ppm; **^{13}C-NMR** (75.53 MHz, $CDCl_3$): δ = 166.1 (C_q, CO), 141.5 (CH), 140.7 (CH), 139.9 (C^{Ar}), 139.4 (C^{Ar}), 138.6 (C^{Ar}), 136.9 (C_q, C^{Ar}), 136.7 (C^{Ar}), 135.0 (C^{Ar}), 133.9 (C^{Ar}), 125.0 (C_q, C^{Ar}), 123.0 (CH), 122.5 (CH), 92.7 (C_q, C^{Ar}), 91.5 (C_q, C^{Ar}), 61.0 (CH_2), 60.7 (CH_2), 14.4 (CH_3), 14.2 (CH_3) ppm; **HRMS** (DI-EI TOF): calcd. *(m/z)* for [M^+]: 379.8909; found: 379.8921.

2.5.11 Ethyl 3-(2-bromo-5-iodophenyl)propanoate

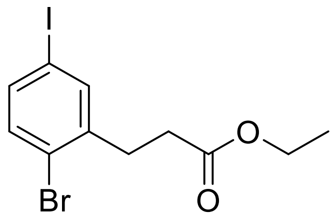

30

A 250 mL one-neck round-bottom flask, equipped with reflux-condenser, was charged with 3.30 g (8.69 mmol, 1.00 eq) ethyl 3-(2-bromo-5-iodophenyl)acrylate (**29**) which was then dissolved in 70 mL THF. At first 9.71 g (52.1 mmol, 6.00 eq) p-tosylhydrazide and afterwards 7.10 g (52.1 mmol, 6.00 eq) $NaOAc\cdot 3H_2O$ were added and the pale-yellow suspension was stirred at 70 °C until quantitative conversion (50 h) was detected via GC-MS. The reaction mixture was cooled to RT and 75 mL satd. $NaHCO_3$-solution were added. The phases were separated, and the aqueous layer was extracted with DCM (3 x 100 mL). The combined organic layers were dried over Na_2SO_4, filtered and the solvent was removed under reduced pressure. The crude product was purified via flash column chromatography (500 g SiO_2, 5.5 x 19.0 cm, eluent: cyclohexane/EtOAc = 75/1).

Yield: 3.05 g (92%), colorless solid, $C_{11}H_{12}BrIO_2$ [381.91 g/mol]

TLC: Rf = 0.17 (cyclohexane/EtOAc = 75/1, UV and $KMnO_4$); **mp**$^{exp.}$ = 42-46 °C; **^{1}H-NMR** (300.36 MHz, $CDCl_3$): δ = 7.58 (d, ^4J (H,H) = 1.6 Hz, 1H, H^{Ar}), 7.38 (dd, ^3J (H,H) = 8.3 Hz, ^4J (H,H) = 1.8 Hz, 1H, H^{Ar}), 7.25 (d, ^3J (H,H) = 8.5 Hz, 1H, H^{Ar}), 4.15 (q, ^3J (H,H) = 7.1 Hz, 2H, CH_2), 3.00 (t, ^3J (H,H) = 7.7 Hz, 2H, CH_2), 2.62 (t, ^3J (H,H) = 7.7 Hz, 2H, CH_2), 1.25 (t, ^3J (H,H) = 7.1 Hz, 3H, CH_3) ppm; **^{13}C-NMR** (75.53 MHz, $CDCl_3$, APT): δ = 172.4 (C_q, CO), 142.3 (C_q, C^{Ar}), 139.4 (C^{Ar}), 137.2 (C^{Ar}), 134.6 (C^{Ar}), 124.4 (C_q, C^{Ar}), 92.6 (C_q, C^{Ar}), 60.8 (CH_2), 34.0 (CH_2), 31.2 (CH_2), 14.4 (CH_3) ppm; **HRMS** (DI-EI TOF): calcd. *(m/z)* for [M^+]: 381.9065; found: 381.8986.

2.6 Synthesis of Phenylalanine and Tyrosine building blocks

2.6.1 2-Bromo-5-iodobenzoyl chloride

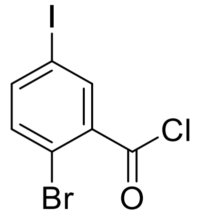

32a

This compound was prepared according to the literature.[10]

In a 100 mL one-neck round-bottom flask with Schlenk adapter 2.00 g (6.12 mmol, 1.00 eq) 2-bromo-5-iodobenzoic acid (**31**) were suspended in 25 mL DCM. The brown suspension was cooled to 0 °C. Then 790 µL (9.21 mmol, 1.51 eq) oxalylchloride were slowly added via a syringe. 0.2 mL DMF were added, and the brown suspension was stirred at 0 °C for 20 min. Afterwards the suspension was allowed to warm up to RT. After full conversion of the starting material was indicated by GC-MS (16 h) the solvent was removed under high vacuum using a cooling trap. The crude product was used without further purification in the next step.

Yield: 2.33 g (110% crude yield), brown solid, C_7H_3BrClO [343.81 g/mol].

2.6.2 (2-Bromo-5-iodophenyl)(phenyl)methanone

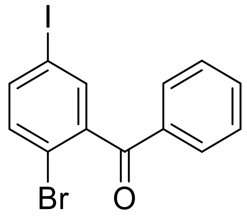

32

In a flame dried 8 mL Schlenk-flask 300 mg (869 µmol, 1.00 eq) 2-bromo-5-iodobenzoyl chloride (**32a**) were dissolved in 1 mL benzene. The orange-brown solution was cooled to 0 °C in an ice bath. Then 117 mg (878 µmol, 1.21 eq) $AlCl_3$ were added slowly over 5 min and the red/brown suspension was stirred at 0 °C for 20 min. Afterwards the suspension was allowed to warm up to RT. After full conversion of the starting material was indicated by TLC and GC-MS (13 h) the reaction mixture was poured onto ice. The phases were separated, and the aqueous phase was extracted with DCM (3 x 10 mL). The combined organic layers were dried over Na_2SO_4, filtered and the solvent was removed under reduced pressure. The yellow oil was purified via flash column chromatography (25 g SiO_2, 2.5 x 9.0 cm, eluent: cyclohexane/EtOAc = 30/1, fraction size: 8 mL).

Yield: 225 mg (67%), brown solid, $C_{13}H_8BrIO$ [387.01 g/mol]

TLC: R_f = 0.59 (cyclohexane/EtOAc = 9/1, UV and $KMnO_4$); **mp**$^{exp.}$ = 90-95°C; **GC-MS** (Method_1): t_R = 7.49 min, *m/z* = 386 (20%), 309 (8%), 281 (5%), 154 (7%), 105 (100%), 77 (48%); **^{1}H-NMR** (300.36 MHz, $CDCl_3$): δ = 7.80 (d, ^3J (H,H) = 7.2 Hz, 2H, H^{Ar}), 7.68 – 7.60 (m, 3H, H^{Ar}), 7.49 (t, ^3J (H,H) = 7.6 Hz, 2H, H^{Ar}), 7.37 (d, ^3J (H,H) = 8.1 Hz, 1H, H^{Ar}) ppm; **^{13}C-NMR** (75.53 MHz, $CDCl_3$): δ = 194.2 (C_q, CO), 142.8 (C_q, C^{Ar}), 140.2 (C^{Ar}), 137.5 (C^{Ar}), 135.7 (C_q, C^{Ar}), 134.9 (C^{Ar}), 134.2 (C^{Ar}), 130.4 (C^{Ar}), 128.9 (C^{Ar}), 119.4 (C_q, C^{Ar}), 92.3 (C_q, C^{Ar}) ppm; **HRMS** (DI-EI TOF): calcd. *(m/z)* for [M^+]: 385.8803; found: 385.8813.

2.6.3 (2-Bromo-5-iodophenyl)(phenyl)methanone

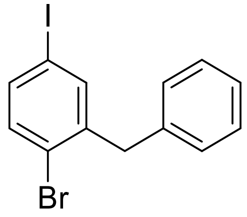

34

In an 8 mL Schlenk-flask 100 mg (258 µmol, 1.00 eq) ((2-bromo-5-iodophenyl)-(phenyl)methanone (**32**) and 170 µL (1.06 mmol, 4.12 eq) Et_3SiH were dissolved in 1.5 mL CH_3CN/DCM (2/1). The colorless suspension was cooled to 0 °C. Then 390 µL (3.16 µmol, 12.23 eq) $BF_3{\cdot}Et_2O$ were slowly added via a syringe. The red suspension was stirred at 50 °C overnight. After full conversion (96 h) of the starting material was indicated by GC-MS the solution was quenched by the addition of 10 mL conc. KOH solution, the phases were separated, and the aqueous layer was extracted with EtOAc (3 x 15 mL). The combined organic layers were dried over Na_2SO_4, filtered and the solvent was removed under reduced pressure. The orange oil was purified via flash column chromatography (10 g SiO_2, eluent: cyclohexane, fraction size: 15 mL).

Yield: 20.6 mg (21%), colorless oil, $C_{13}H_{10}BrI$ [373.03g/mol]

TLC: R_f = 0.69 (cyclohexane, UV and $KMnO_4$); **GC-MS** (Method_1): t_R = 7.14 min, *m/z* = 372 (37%), 293 (9%), 166 (100%), 139 (9%), 82 (9%); ^{1}**H-NMR** (300.36 MHz, $CDCl_3$): δ = 7.45 (d, ^4J (HH = 1.7 Hz, 1H, H^{Ar}), 7.39 (dd, ^3J (H,H) = 8.3 Hz, ^4J (H,H) = 1.9 Hz, 1H, H^{Ar}), 7.35 – 7.13 (m, 6H, H^{Ar}), 4.05 (s, 2H, CH_2) ppm; ^{13}**C-NMR** (75.53 MHz, $CDCl_3$): δ = 142.9 (C_q, C^{Ar}), 139.9 (C^{Ar}), 138.8 (C_q, C^{Ar}), 137.1 (C^{Ar}), 134.6 (C^{Ar}), 129.1 (C^{Ar}), 128.8 (C^{Ar}), 126.7 (C^{Ar}), 124.9 (C_q, C^{Ar}), 92.8 (C_q, C^{Ar}), 41.6 (CH_2) ppm; **HRMS** (DI-EI TOF): calcd. *(m/z)* for [M^+]: 371.9011; found: 371.9024.

2.6.4 (2-Bromo-5-iodophenyl)(4-methoxyphenyl)methanone

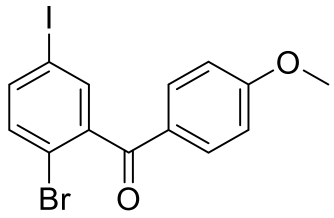

33

This compound was prepared according to the literature.[10]

In a flame dried 8 mL Schlenk-flask 100 mg (290 mmol, 1.00 eq) 2-bromo-5-iodobenzoyl chloride (**32a**) were suspended in 1 mL DCM. The red-brown suspension was cooled to 0 °C. Then 31.3 µL (287 µmol, 0.99 eq) anisole were slowly added via a syringe. 47.0 mg (348 µmol, 1.20 eq) $AlCl_3$ were added slowly over 10 min and the brown suspension was stirred at 0 °C for 20 min. Afterwards

the suspension was allowed to warm up to RT. After full conversion of the starting material was indicated by GC-MS (13 h) the reaction mixture was poured onto ice. The phases were separated, and the aqueous phase was extracted with DCM (3 x 10 mL). The combined organic layers were dried over Na_2SO_4, filtered and the solvent was removed under reduced pressure. The red-brown oil was purified via flash column chromatography (13 g SiO_2, 2.0 x 7.0 cm, eluent: cyclohexane/EtOAc = 30/1, fraction size: 5 mL).

Yield: 94.2 mg (78%), colorless solid, $C_{14}H_{10}BrIO_2$ [417.04 g/mol]

TLC: R_f = 0.17 (cyclohexane/EtOAc = 30/1, UV and $KMnO_4$); **mp**$^{exp.}$ = 114-118 °C; **GC-MS** (Method_1): t_R = 8.34 min, *m/z* = 416 (48%), 311 (5%), 281 (5%), 135 (100%), 77 (12%); **^{1}H-NMR** (300.36 MHz, $CDCl_3$): δ = 7.77 (d, ^3J (H,H) = 8.8 Hz, 2H, H^{Ar}), 7.65 – 7.62 (m, 2H, H^{Ar}), 7.36 (d, ^3J (H,H) = 8.0 Hz, 1H, H^{Ar}), 6.95 (d, ^3J (H,H) = 8.8 Hz, 2H, H^{Ar}), 3.89 (s, 3H, CH_3) ppm; **^{13}C-NMR** (75.53 MHz, $CDCl_3$): δ = 192.7 (C_q, CO), 164.5 (C_q, C^{Ar}), 143.2 (C_q, C^{Ar}), 139.9 (C^{Ar}), 137.3 (C^{Ar}), 134.8 (C^{Ar}), 132.8 (C^{Ar}), 128.7 (C_q, C^{Ar}), 119.4 (C_q, C^{Ar}), 114.2 (C^{Ar}), 92.3 (C_q, C^{Ar}), 55.7 (CH_3) ppm; **HRMS** (DI-EI TOF): calcd. *(m/z)* for [M^+]: 415.8909; found: 415.8912.

2.6.5 1-Bromo-4-iodo-2-(4-methoxybenzyl)benzene

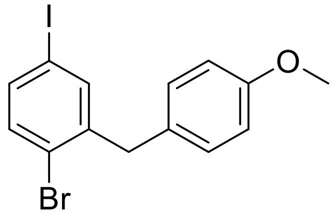

35

This compound was prepared according to the literature.[10]

In a 8 mL Schlenk-flask 200 mg (480 µmol, 1.00 eq) (2-bromo-5-iodophenyl)(4-methoxyphenyl)methanone (**33**) and 310 µL (1.94 mmol, 4.05 eq) Et_3SiH were dissolved in 1.8 mL CH_3CN/DCM (2/1). The colorless suspension was cooled to 0 °C. Then 90 µL (729 µmol, 1.52 eq) $BF_3{\cdot}Et_2O$ were slowly added via a syringe. The pink suspension was stirred at RT overnight. After full conversion (16 h) of the starting material was indicated by GC-MS the solution was quenched by the addition of 10 mL conc. KOH solution, the phases were separated, and the aqueous layer was extracted with EtOAc (3 x 20 mL). The combined organic layers were dried over Na_2SO_4,

filtered and the solvent was removed under reduced pressure. The orange oil was purified via flash chromatography (30 g SiO_2, eluent: cyclohexane → cyclohexane/EtOAc = 100/1, fraction size: 15 mL).

Yield: 185 mg (96%), colorless solid, $C_{14}H_{12}BrIO$ [403.06 g/mol]

TLC: R_f = 0.16 (cyclohexane, UV and $KMnO_4$); **mp**$^{exp.}$ = 120-123 °C; **GC-MS** (Method_1): t_R = 7.79 min, *m/z* = 402 (100%), 323 (20%), 275 (8%), 181 (24%), 152 (46%), 121 (46%); **^{1}H-NMR** (300.36 MHz, $CDCl_3$): δ = 7.35 (d, ^4J (H,H) = 1.7 Hz, 1H, H^{Ar}), 7.31 (dd, ^3J (H,H) = 8.3 Hz, ^4J (H,H) = 1.9 Hz, 1H, H^{Ar}), 7.19 (d, ^3J (H,H) = 7.9 Hz, 1H, H^{Ar}), 7.02 (d, ^3J (H,H) = 8.4 Hz, 2H, H^{Ar}), 6.78 (d, ^3J /H,H) = 8.5 Hz, 2H, H^{Ar}), 3.91 (s, 2H, CH_2), 3.72 (s, 3H, CH_3) ppm; **^{13}C-NMR** (75.53 MHz, $CDCl_3$): δ = 158.4 (C_q, C^{Ar}), 143.3 (C_q, C^{Ar}), 139.7 (C^{Ar}), 137.0 (C^{Ar}), 134.6 (C^{Ar}), 130.8 (C_q, C^{Ar}), 130.1 (C^{Ar}), 124.8 (C_q, C^{Ar}), 114.2 (C^{Ar}), 92.8 (C_q, C^{Ar}), 55.4 (CH_2), 40.7 (CH_3) ppm; **HRMS** (DI-EI TOF): calcd. *(m/z)* for [M^+]: 401.9116; found: 401.9120.

2.7 Synthesis of Lysine and Tryptophane building blocks

2.7.1 3-(2-Bromo-5-iodophenyl)propan-1-ol

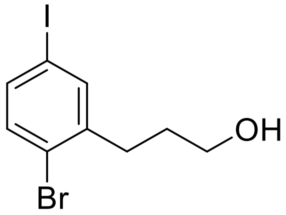

36

In a flame dried 8 mL Schlenk flask 500 mg (1.31 mmol, 1.00 eq) ethyl 3-(2-bromo-5-iodophenyl)propanoate (**30**) were dissolved in 2 mL abs DCM. The solution was cooled to -78 °C in a dry ice/acetone bath. Then 2.60 mL (1M in DCM, 2.61 mmol, 2.00 eq) DIBAL-H were added via a syringe. The colorless solution was stirred at RT. After full conversion of the starting material was indicated by GC-MS (90 min) the reaction mixture was quenched carefully by the addition of 5.0 mL MeOH. Then 15 mL satd. Rochelle salt solution were added, and the reaction was stirred for another 12 h. The phases were separated, and the aqueous phase was extracted with DCM (3 x 50 mL). The combined organic layers were dried over Na_2SO_4, filtered and the solvent was removed under reduced pressure. The crude product was purified via flash column chromatography (50 g SiO_2, 2.5 x 12.0 cm, eluent: cyclohexane/EtOAc = 8/1, fraction size: 20 mL).

Yield: 407 mg (91%), colorless solid, $C_9H_{10}BrIO$ [340.99 g/mol]

TLC: R_f = 0.14 (cyclohexane/EtOAc = 8/1, UV and $KMnO_4$); **mp**$^{exp.}$ = 49-53 °C; **GC-MS** (Method_1): t_R = 6.64 min, *m/z* = 342 (42%), 295 (21%), 260 (28%), 171 (25%), 116 (100%); **^{1}H-NMR** (300.36 MHz, $CDCl_3$): δ = 7.57 (d, ^4J (H,H) = 1.8 Hz, 1H, H^{Ar}), 7.37 (dd, ^3J (H,H) = 8.3 Hz, ^4J (H,H) = 1.9 Hz, 1H, H^{Ar}), 7.25 (d, ^3J (H,H) = 8.9 Hz, 1H, H^{Ar}), 3.70 (t, ^3J (H,H) = 6.2 Hz, 2H, CH_2), 2.78 (t, ^3J (H,H) = 7.6 Hz, 2H, CH_2), 1.95 – 1.74 (m, 2H, CH_2) ppm; **^{13}C-NMR** (75.53 MHz, $CDCl_3$): δ = 143.7 (C_q, C^{Ar}), 139.2 (C^{Ar}), 136.8 (C^{Ar}), 134.6 (C^{Ar}), 124.5 (C_q, C^{Ar}), 92.7 (C_q, C^{Ar}), 62.1 (CH_2), 32.6 (CH_2), 32.3 (CH_2) ppm; **HRMS** (DI-EI TOF): calcd. *(m/z)* for [M^+]: 339.8960; found: 339.8970.

2.7.2 4-(2-Bromo-5-iodophenyl)butanenitrile

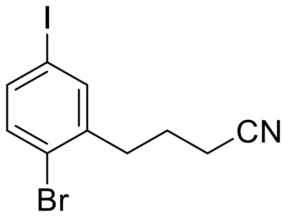

37

A 25 ml round-bottom flask was charged with 2 mL DCM, 160 mg (0.47 mmol, 1.0 eq) **36**, 200 µL (2.48 mmol, 5.3 eq) pyridine and 180 mg (0.94 mmol, 2.0 eq) p-toluenesulfonyl chloride. The pale-yellow solution was stirred at RT for 4 h. The reaction mixture was diluted with 10 mL DCM and 10 mL 1M HCl were added. The layers were separated and the organic layer was washed with 10 mL satd. $NaHCO_3$ solution. The organic layer was dried over Na_2SO_4 and filtered. The solvent was removed under reduced pressure and the crude product was used in the next step without further purification.

A 25 mL round-bottom flask was charged with the crude from the previous step dissolved in 5 mL DMF. 61 mg (0.94 mmol, 2.0 eq) potassium cyanide and 39 mg (0.23 mmol, 0.5 eq) potassium iodide were added. The colorless solution was stirred at 60 °C for 18 h. After full conversion was detected via GC-MS, the pale-yellow solution was diluted with 15 mL satd. $NaHCO_3$ solution and extracted with Et_2O (1 x 20 mL). The organic phase was washed with water (3 x 10 mL), dried over $MgSO_4$ and the solvent was removed under reduced pressure. The crude product was purified

via flash column chromatography (10 g SiO_2, 1.0 x 12 cm, eluent: cyclohexane/EtOAc = 20/1 → 10/1, fraction size: 5 mL).
Yield: 61 mg (37% over 2 steps), colorless oil, $C_{10}H_9BrIN$ [350.00 g/mol]

TLC: R_f = 0.27 (cyclohexane/EtOAc = 10/1, UV and CAM); **GC-MS** (Method_1): t_R = 7.21 min, *m/z* = 352 (100%), 297 (100%); **^{1}H-NMR** (300.36 MHz, $CDCl_3$): δ = 7.56 (d, ^4J (H,H) = 1.7 Hz, 1H, H^{Ar}), 7.41 (dd, ^3J (H,H) = 8.3 Hz, ^4J (H,H) = 1.9 Hz, 1H, H^{Ar}), 7.28 (d, ^3J (H,H) = 8.3 Hz, 1H, H^{Ar}), 2.84 (t, ^3J (H,H) = 7.4 Hz, 2H, CH_2), 2.40 (t, ^3J (H,H) = 7.1 Hz, 2H, CH_2), 2.03 – 1.93 (m, 2H, CH_2) ppm; **^{13}C-NMR** (75.53 MHz, $CDCl_3$): δ = 141.7 (C_q, C^{Ar}), 139.3 (C^{Ar}), 137.5 (C^{Ar}), 134.8 (C^{Ar}), 124.3 (C_q, C^{Ar}), 119.3 (C_q, CN), 92.8 (C_q, C^{Ar}), 34.8 (CH_2), 25.5 (CH_2), 16.9 (CH_2) ppm; **HRMS** (DI-EI TOF): calcd. *(m/z)* for [M^+-H]: 347.8885; found: 347.8896.

2.7.3 3-(2-Bromo-5-iodophenyl)propanal

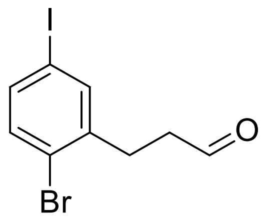

38a

A 50 mL round-bottom flask was charged with 5 mL DCM and 404 mg (1.19 mmol, 1.0 eq.) 3-(2-bromo-5-iodophenyl)propan-1-ol (**36**) were added. To the yellow solution 549 mg (1.29 mmol, 1.1 eq.) Dess-Martin-Periodinane was added. The yellow suspension was stirred at RT for 30 min. After full conversion was detected via TLC and GC-MS, the yellow suspension was filtered through a pad of celite (eluted with 3 x 50 mL DCM) and the solvent was removed under reduced pressure. Filtration through a short pad of SiO_2 (eluted with cyclohexane/EtOAc = 20/1) gave a yellow solid that was used in the following step without further purification.

Yield: 285mg (71% crude yield), yellow solid, C_9H_8BrIO [338.97 g/mol].

2.7.4 3-(2-Bromo-5-iodobenzyl)-1*H*-indole

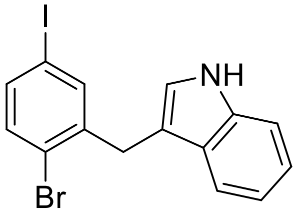

38

In a 50 mL round-bottom flask equipped with refluxing condenser 500 mg (1.47 mmol, 1.0 eq) 3-(2-bromo-5-iodophenyl)propanal (**38a**) were dissolved in a mixture of 290 µL (2.95 mmol, 2.0 eq) phenylhydrazine, 1 mL H_2SO_4 (96% w/w) and 9 mL THF. The orange solution was stirred at 70 °C for 1 h. The reaction mixture was cooled to RT and neutralized with 50 mL satd. Na_2CO_3 solution. The neutral solution was extracted with DCM (2 x 50 mL). The combined organic layers were dried over Na_2SO_4, filtered, and concentrated under reduced pressure. The orange crude was purified via flash column chromatography (20 g SiO_2, 2 x 10 cm, eluent: cyclohexane/EtOAc = 20/1 → 10/1).

Yield: 589 mg (97%), pale brown oil, $C_{15}H_{11}BrIN$ [412.06 g/mol]

TLC: R_f = 0.23 (cyclohexane/EtOAc = 10/1, UV and CAM); **^{1}H-NMR** (300 MHz, $CDCl_3$): δ = 8.01 (bs, 1H; NH), 7.61 – 7.46 (m, 2H; H^{Ar}), 7.44 – 7.33 (m, 2H; H^{Ar}), 7.33 – 7.17 (m, 2H; H^{Ar}), 7.13 (t, ^3J (H,H) = 7.1 Hz, 1H; H^{Ar}), 6.94 (s, 1H; H^{Ar}), 4.16 (s, 2H; CH_2); **^{13}C-NMR** (75.53 MHz, $CDCl_3$): δ = 143.0 (C_q; C^{Ar}), 139.4 (C^{Ar}), 136.8 (C^{Ar}), 136.5 (C_q; C^{Ar}), 134.4 (C^{Ar}), 127.3 (C_q; C^{Ar}), 124.6 (C_q; C^{Ar}), 123.0 (C^{Ar}), 122.4 (C^{Ar}), 119.8 (C^{Ar}), 119.1 (C^{Ar}), 113.4 (C_q; C^{Ar}), 111.3 (C^{Ar}), 92.8 (C_q; C^{Ar}), 31.7 (CH_2); **HRMS** (EI): calcd (*m/z*) for [M^+–H]: 410.9120; found: 410.9116.

2.8 Synthesis of Histidine building block

2.8.1 4-Iodo-1-trityl-1*H*-imidazole

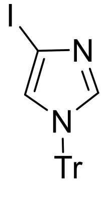

39

This compound was prepared according to the literature.[11]

A 50 mL round-bottom flask was charged with 951 mg (4.90 mmol, 1.0 eq) 4-iodo-1*H*-imidazole dissolved in 10 mL DMF. 713 µL (5.14 mmol, 1.1 eq) Et_3N and 1.37 g (4.90 mmol, 1.0 eq) triphenylmethylchloride were added to this pale brown solution, which turned into a colourless suspension within 60 min. After quantitative conversion had been detected by TLC (24 h), the reaction mixture was poured into 50 mL ice water. The beige precipitate was collected by filtration. The crude product was triturated in 8 mL Et_2O and stirred for 10 min. The colorless solid was collected by filtration and dried in oil pump vacuum.

Yield: 1.26 g (59%), colorless solid, $C_{22}H_{17}IN_2$ [436.30 g/mol]

TLC: R_f = 0.55 (cyclohexane/EtOAc = 3/1, UV and CAM); **mp**$^{exp.}$ = 235-237 °C; **^{1}H-NMR** (300.36 MHz, $CDCl_3$): δ = 7.34 – 7.28 (m, 10H, H^{Ar}), 7.12 – 7.06 (m, 6H, H^{Ar}), 6.88 (s, 1H, H^{Ar}) ppm; **^{13}C-NMR** (75.53 MHz, $CDCl_3$): δ = 142.0 (C_q, C^{Ar}), 140.7 (C^{Ar}), 129.9 (C^{Ar}), 128.4 (C^{Ar}), 128.3 (C^{Ar}), 127.0 (C^{Ar}), 81.5 (C_q, C^{Ar}), 76.0 (C_q) ppm.

2.8.2 (2-Bromo-5-iodophenyl)(1-trityl-1*H*-imidazol-4-yl)methanol

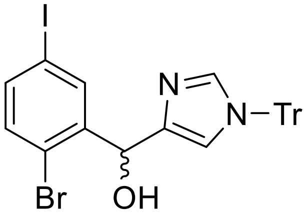

40

In a flame dried and argon flushed Schlenk-flask 140 mg (321 µmol, 1.00 eq) iodo-1-trityl-1*H*-imidazole (**39**) were dissolved in 2 mL abs. THF. 115 µL (322 µmol, 1.00 eq) EtMgBr solution (2.80 M in Et_2O) were added and the yellowish solution was stirred at RT for 90 min. After quantitative iodine-magnesium exchange had been detected by HPLC-MS, 100 mg (322 µmol, 1.00 eq) 2-bromo-5-iodobenzaldehyde (**16**) were added. The color turned into yellow. After full conversion was detected by HPLC-MS (14 h), the reaction was quenched by the addition of 20 mL satd. NH_4Cl solution. The phases were separated and the aqueous phase was extracted with DCM

(3 x 30 mL). The combined organic layers were dried over Na_2SO_4, filtered and the solvent was removed under reduced pressure. The colorless crude product was purified via flash column chromatography (20 g SiO_2, 1.5 x 9.0 cm, eluent: cyclohexane/EtOAc = 2/1, Rf = 0.11, UV and CAM, fraction size: 10 mL).

Yield: 174 mg (87%), colorless solid, $C_{29}H_{22}BrIN_2O$ [621.32 g/mol]

TLC: R_f = 0.11 (cyclohexane/EtOAc = 2/1, UV and CAM); **mp**$^{exp.}$ = 199-201 °C; **^{1}H-NMR** (300.36 MHz, [D6]DMSO): δ = 7.82 (d, ^3J (H,H) = 1.6 Hz, 1H, H^{Ar}), 7.49 (dd, ^3J (H,H) = 8.3 Hz, ^4J (H,H) = 1.8 Hz, 1H, H^{Ar}), 7.48 – 7.32 (m, 9H, H^{Ar}), 7.30 (m, 2H, H^{Ar}), 7.09 – 7.00 (m, 6H, H^{Ar}), 6.68 (s, 1H, H^{Ar}), 5.94 (d, ^3J (H,H) = 5.1 Hz, 1H, OH), 5.74 (d, ^3J (H,H) = 5.0 Hz, 1H, CH); **^{13}C-NMR** (75.53 MHz, [D6]DMSO): δ = 145.5 (C_q, C^{Ar}), 142.5 (C_q, C^{Ar}), 142.1 (C_q, C^{Ar}), 138.0 (C^{Ar}), 137.6 (C^{Ar}), 137.3 (C^{Ar}), 134.1 (C^{Ar}), 129.2 (C^{Ar}), 128.2 (C^{Ar}), 128.1 (C^{Ar}), 122.0 (C_q, C^{Ar}), 119.0 (C^{Ar}), 93.3 (C_q, C^{Ar}), 74.6 (C_q), 68.4 (CH) ppm; **HRMS** (DI-EI TOF): calcd. *(m/z)* for [M^+]: 619.9960; found: 619.9950.

2.8.3 2-(2-Bromo-5-iodophenyl)acetaldehyde

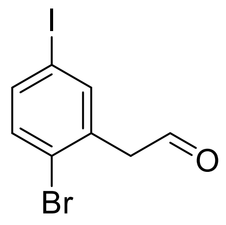

41

In a flame dried 10 mL Schlenk-flask 200 mg (563 µmol, 2.50 eq) methyl 2-(2-bromo-5-iodophenyl)acetate (**13**) were dissolved in 2 mL abs DCM. Then the colorless solution was cooled to –78 °C. Afterwards 565 µL (565 µmol, 1.00 eq) DIBAL-H (1.0M in DCM) were slowly added over 5 min. The yellowish solution was stirred at -78 °C for 30 min. After full conversion was indicated by GC-MS, 5 mL 25% tartaric acid solution were added, and the reaction mixture was extracted with DCM (3 x 10 mL). The organic phase was dried over Na_2SO_4, filtered and the solvent was removed under reduced pressure. The crude product was purified via flash column chromatography (30 g SiO_2, 2.5 x 9.5 cm, eluent: cyclohexane/EtOAc = 30/1, fraction size: 10 mL).

Yield: 121 mg (66%), colorless solid, C_8H_6BrIO [324.94 g/mol]

TLC: R_f = 0.40 (cyclohexane/EtOAc = 9/1, UV and $KMnO_4$); **mp**$^{exp.}$ = 58-66 °C; **GC-MS** (Method_1): t_R = 6.13 min, *m/z* = 324 (50%), 295 (60%), 245 (100%), 217 (30%), 171 (28%), 89 (80%); **^{1}H-NMR** (300.36 MHz, $CDCl_3$): δ = 9.75 (s, 1H, COH), 7.57 (d, ^{4}J (H,H) = 1.6 Hz, 1H, H^{Ar}), 7.49 (dd, ^{3}J (H,H) = 8.4 Hz, ^{4}J (H,H) = 1.9 Hz, 1H, H-6), 7.33 (d, ^{3}J (H,H) = 8.4 Hz, 1H, H^{Ar}), 3.82 (s, 2H, CH_2) ppm; **^{13}C-NMR** (75.53 MHz, $CDCl_3$): δ = 197.3 (CO), 140.5 (C^{Ar}), 138.4 (C^{Ar}), 135.1 (C_q, C^{Ar}), 134.7 (C^{Ar}), 125.0 (C_q, C^{Ar}), 92.8 (C_q, C^{Ar}), 50.2 (CH_2) ppm; **HRMS** (DI-EI TOF): calcd. *(m/z)* for [M^+]: 323.8647; found: 323.8658.

2.8.4 4-(2-Bromo-5-iodobenzyl)-1H-imidazole

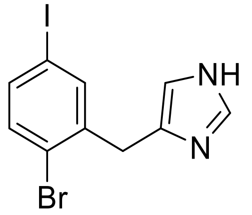

42

In a flame dried 8 mL Schlenk-flask 150 mg (769 µmol, 1.00 eq) p-tosylmethylisocyanide were dried in vacuum for 30 min. Then 2.0 mL abs. EtOH were added, and the colorless suspension was cooled to 0 °C. Afterwards 250 mg (769 µmol, 1.00 eq) 2-(2-bromo-5-iodophenyl) acetaldehyde (**41**) were added at 0 °C. 245 mg (1.15 mmol, 1.50 eq) K_3PO_4 were added to the yellow suspension, which was stirred at 0 °C for 30 min, followed by 60 min at RT. After full conversion was indicated via GC-MS the reaction mixture was transferred to a separation funnel. The aqueous phase was extracted with DCM (3 x 10 mL). The combined organic phases were dried over Na_2SO_4, filtered and the solvent was removed under reduced pressure. The crude product was used without further purification in the next step.

In an 8 mL pressure tube 100 mg (769 µmol, 1.00 eq) crude 5-(2-bromo-5-iodobenzyl)-4-tosyl-4,5-dihydrooxazole were suspended in 2.0 mL NH_3 (7M in MeOH). The suspension was stirred overnight at 70 °C. After full conversion was indicated via HPLC-MS the solvent was removed under reduced pressure. The crude product was purified via flash column chromatography (20 g SiO_2, eluent: DCM/MeOH = 10/1, fraction size: 5.0 mL).

Yield: 59 mg (21%), yellowish solid, $C_{10}H_8BrIN_2$ [363.00 g/mol]

HPLC-MS (Poroshell, ESI+, MV_general): t_R = 3.88 min, *m/z* = 364 [$M+H^+$]; **^{1}H-NMR** (300.36 MHz, [D6]DMSO): δ = 7.62 (s, 1H, H^{Ar}), 7.58 (d, ^4J (H,H) = 1.4 Hz, 1H, H^{Ar}), 7.49 (dd, ^3J (H,H) = 8.4 Hz, ^4J (H,H) = 1.4 Hz, 1H, H^{Ar}), 7.37 (d, ^3J (H,H) = 8.3 Hz, 1H, H^{Ar}), 6.82 (s, 1H, H^{Ar}), 3.89 (s, 2H, CH_2) ppm; **^{13}C-NMR** (75.53 MHz, [D6]DMSO): δ = 142.1 (C_q, C^{Ar}), 139.2 (C^{Ar}), 136.8 (C^{Ar}), 135.2 (C_q, C^{Ar}), 134.6 (C^{Ar}), 134.3 (C^{Ar}), 123.7 (C_q, C^{Ar}), 116.6 (C^{Ar}), 93.5 (C_q, C^{Ar}), 33.0 (CH_2) ppm; **HRMS** (DI-EI TOF): calcd. *(m/z)* for [M^+]: 361.8916; found: 361.8915.

3 Experimental Procedures and Analytical Data for Teraryl Synthesis

3.1 Representative procedure for the synthesis of teraryls by consecutive double Suzuki-Coupling (1st step)

A flame dried Schlenk-flask was charged with 1.0 eq of the corresponding boronic acid pinacol ester, 2.0-3.0 eq K_2CO_3, and 5 mol% $PdCl_2$(dppf). After drying in vacuo, a solution of 1.0 eq middle building block in absolute, degassed DMF (~0.2M) was added. The reaction mixture was stirred at 80 °C until full conversion was detected via GC-MS or TLC. The typically brown suspension was filtered through a pad of SiO_2 (3 x 2 cm, eluted with EtOAc) and the filtrate was concentrated to dryness using a rotary evaporator. The crude product was purified via flash column chromatography or used in the next step without further purification.

3.2 Representative procedure for the synthesis of teraryls by consecutive double Suzuki-Coupling (2nd step)

Another flame dried Schlenk-flask was charged with 1.0-1.2 eq of the second boronic acid pinacol ester, 2.0-3.0 eq cesium carbonate (Cs_2CO_3), and 5 mol% $PdCl_2$(dppf). After drying in vacuo, a solution of the previously prepared intermediate in absolute, degassed DMF (~0.2M) was added. The reaction mixture was stirred at 80 °C overnight. The typically black suspension was filtered through a pad of SiO_2 (3 x 2 cm, eluent: MeOH) and after concentrating to dryness, the crude product was purified via flash column chromatography.

3.3 3-Chloro-5-isobutylpyridine

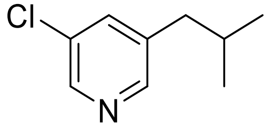

43a

A three-neck round-bottom flask equipped with a reflux condenser, inline oil bubbler (after flame drying), dropping funnel and vacuum adapter with stopcock was charged with 2.00 g (82.3 mmol, 1.0 eq) Mg turnings. Then the setup was flame dried and flushed with argon. Under inert conditions 10 mL abs. THF were added and the Mg was activated by adding I_2. A solution of 8.90 mL (11.3 g, 82.3 mmol, 1.0 eq) isobutyl bromide in 20 mL abs. THF were added dropwise via the dropping funnel. After stirring the grey, turbid reaction mixture for 2 h at 70 °C the isobutyl magnesium bromide solution was transferred to a flame dried and argon flushed Schlenk-flask. For determination of the actual concentration of the Grignard solution, a titration was performed as described in 1.

A flame dried and argon flushed three-neck round-bottom flask equipped with dropping funnel and vacuum adapter with stopcock was charged with 3.0 g (20.3 mmol, 1.0 eq) 3,5-dichloropyridine and 358 mg (1.01 mmol, 5 mol%) $Fe(acac)_2$. 120 mL abs. THF and 6.5 mL abs. NMP were added and a red-orange solution was formed. The solution was cooled to 0 °C in an ice bath and 6.9 mL (3.27 g, 20.3 mmol, 1.0 eq) isobutylmagnesium bromide (2.95M in THF) were added dropwise via the dropping funnel. The reaction mixture turned dark brownish purple and was stirred overnight at RT. When quantitative conversion was detected via GC-MS (16 h) the catalyst was removed by filtration through a pad of silica gel (eluted with 150 mL EtOAc) and the solvent of the collected filtrate was removed under reduced pressure to yield a brown, oily crude product, which was purified via flash column chromatography (250 g SiO_2, 6.0 x 16 cm, eluent: cyclohexane/EtOAc = 5/1, R_f = 0.52, UV).

Yield: 2.88 g (84%), pale yellow oil, $C_9H_{12}ClN$ [169.65 g/mol]

TLC: R_f = 0.52 (cyclohexane/EtOAc = 5/1, UV and CAM); **GC-MS** (Method_1): t_R = 9.70 min; *m/z* (%): 169 (40) [M^+], 127 (100) [$M^+-C_3H_7$], 92 (16) [$M^+-C_3H_7Cl$]; **^{1}H NMR** (300 MHz, $CDCl_3$): δ = 8.38 (d, ^4J (H,H) = 2.1 Hz, 1H, H^{Ar}), 8.25 (d, ^4J (H,H) = 1.3 Hz, 1H, H^{Ar}), 7.43 (bs, 1H, CH), 2.44 (d, ^3J (H,H) = 7.2 Hz, 2H, CH_2), 1.84 (h, ^3J (H,H) = 6.8 Hz, 1H, CH), 0.89 (d, ^3J (H,H) = 6.6 Hz, 6H, CH_3) ppm; **^{13}C NMR** (76 MHz, $CDCl_3$, APT): δ = 148.4 (C^{Ar}), 146.3 (C^{Ar}), 138.2 (C_q, C^{Ar}), 136.2 (C^{Ar}), 131.7 (C_q, C^{Ar}), 41.9 (CH_2), 30.0 (CH), 22.2 (CH_3) ppm.

Analytical data are in accordance with those reported.[12]

3.3.1 3-Isobutyl-5-(4,4,5,5-tetramethyl-1,3,2-dioxaborolan-2-yl)pyridine

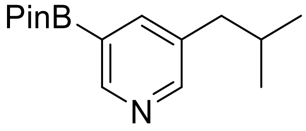

43

A flame dried round bottom flask with Schlenk adapter was charged with 3.01 g (17.7 mmol, 1.0 eq) 3-chloro-5-isobutylpyridine (**43a**), 4.96 g (19.5 mmol, 1.1 eq) bis(pinacolato)diboron, 98.0 mg (0.11 mmol, 0.6 mol%) Pd_2dba_3 and 143 mg (0.30 mmol, 1.7 mol%) XPhos. The round bottom flask was evacuated and flushed with argon (3x). Then, 30 mL abs., degassed 1,4-dioxane were added. The obtained brown-red suspension was stirred at 105 °C for 24 h. After full conversion was detected by GC-MS, the solvent was removed under reduced pressure. The crude product was dissolved in 100 mL EtOAc, washed with H_2O (100 mL) and the aqueous layer was extracted with EtOAc (2 x 100 mL). The combined organic layers were dried over Na_2SO_4, the solvent was removed under reduced pressure and the crude product was further purified via Kugelrohr-distillation (125 °C, 0.1 mbar).

Yield: 4.31 g (93%) colourless solid, $C_{15}H_{24}BNO_2$ [261.17 g/mol].

m.p.$^{exp.}$ = 71-73 °C, (m.p.$^{lit.}$ = 75-77 °C);[13] **GC-MS** (Method_1): t_R = 6.53 min; *m/z* (%): 261 (64) [M^+], 246 (100) [M^+−CH_3], 218 (46) [M^+−C_3H_7], 162 (69) [M^+−$C_6H_{12}O$]; **^{1}H NMR** (300 MHz, $CDCl_3$): δ = 8.77 (d, ^4J (H,H) = 1.3 Hz, 1H, H^{Ar}), 8.46 (d, ^4J (H,H) = 2.2 Hz, 1H, H^{Ar}), 7.88 (bs, 1H, H^{Ar}), 2.48 (d, ^3J (H,H) = 7.2 Hz, 2H, CH_2), 1.95-1.82 (m, 1H, CH), 1.35 (s, 12H, CH_3), 0.91 (d, ^3J (H,H) = 6.6 Hz, 6H, CH_3) ppm; **^{13}C NMR** (76 MHz, $CDCl_3$): δ = 152.3 (C^{Ar}), 151.8 (C^{Ar}), 143.4 (C^{Ar}), 136.5 (C_q, C^{Ar}), 84.4 (C_q, C^{Ar}), 42.4 (CH_2), 30.2 (CH), 25.0 (CH_3), 22.4 (CH_3) ppm;[1] **HRMS** (EI): calcd (*m/z*) for [M^+]: 261.1903; found: 261.1884.

Analytical data are in accordance with those reported.[13]

3.3.2 5-Iodonicotinaldehyde

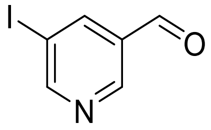

[1] Signal for the quaternary *ipso*-pyridine carbon (C_q; C^{Ar}) at the boronic acid pinacol ester function was not observed.

44a

In a flame dried, nitrogen flushed 100 mL Schlenk-flask 1.0 g (3.0 mmol, 1.0 eq) 3,5-diiodopyridine was dissolved in 10 mL abs. THF. After cooling to -78 °C in a dry ice/acetone bath 2.5 mL (3.2 mmol, 1.1 eq) isopropylmagnesium chloride lithium chloride solution (1.27M in THF) were added under N_2 counter flow and kept stirring at -78 °C until full conversion of the metal-halogen exchange was detected by GC-MS. After full conversion (3.5 h) 460 µL (5.9 mmol, 2.0 eq) abs. DMF were added at -78 °C under inert conditions. Subsequently the reaction mixture was brought to RT and stirred overnight (12 h). After quantitative conversion the reaction mixture was quenched by the addition of 10 mL satd. NH_4Cl solution and the aqueous layer was extracted with Et_2O (3 x 20 mL). The combined organic layers were dried over Na_2SO_4, filtered and the solvent was removed under reduced pressure to give a pale orange solid. The crude product was purified via flash column chromatography (100 g SiO_2, 4.5 x 15 cm, eluent: cyclohexane/EtOAc = 4/1, R_f = 0.21, UV and CAM).

Yield: 526 mg (78%), colorless powder, C_6H_4INO [233.01 g/mol]

TLC: R_f = 0.21 (cyclohexane/EtOAc = 4/1, UV and CAM); **m.p.**$^{exp.}$ = 143-145 °C; **GC-MS** (Method_1): t_R = 5.28 min; *m/z* (%): 233 (100) [M^+], 204 (16) [M^+-CHO]; **^{1}H NMR** (300 MHz, $CDCl_3$): δ = 10.03 (s, 1H, CHO), 9.05 (d, ^4J (H,H) = 2.0 Hz, 1H, H^{Ar}), 9.00 (t, ^4J (H,H) = 1.6 Hz, 1H, H^{Ar}), 8.49-8.48 (m, 1H, H^{Ar}) ppm; **^{13}C NMR** (76 MHz, $CDCl_3$, APT): δ = 189.4 (CO), 160.8 (C^{Ar}), 150.31 (C^{Ar}), 144.0 (C^{Ar}), 132.9 (C_q, C^{Ar}), 94.0 (C_q, C^{Ar}) ppm.

3.3.3 (E/Z)-3-Iodo-5-(2-(methylthio)vinyl)pyridine

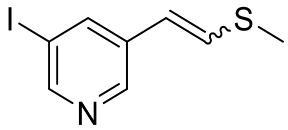

44b

A flame dried and nitrogen flushed 250 mL two-neck round-bottom flask equipped with an N_2-inlet was charged with 4.85 g (13.5 mmol, 1.1 eq) **18** suspended in 96 mL abs. methanol and cooled to 0 °C. After adding 1.52 g (13.5 mmol, 1.1 eq) KO*t*Bu and stirring for 50 min 3.00 g (12.9 mmol, 1.0 eq) 5-iodonicotinaldehyde (**44a**) were added. The reaction mixture was allowed to warm up to RT and stirred overnight. After full conversion was detected by GC-MS, the solvent removed under

vacuum. The crude product was purified via flash column chromatography (155 g SiO2, 7 x 12 cm, cyclohexane/EtOAc = 8/1, R_f = 0.20, UV).

Yield: 3.28 g (E/Z = 5.7/1, 79%), colorless solid, C_8H_8INS [277.12 g/mol]

(E): **m.p.**$^{exp.}$ = 45-55°C; **GC-MS** (Method_1): t_R = 6.84 min; *m/z* (%): 277 (100) [M^+]; **^{1}H NMR** (300 MHz, $CDCl_3$): δ = 8.60 (d, ^4J (H,H) = 1.7 Hz, 1H, H^{Ar}), 8.44 (d, ^4J (H,H) = 1.7 Hz, 1H, H^{Ar}), 7.93 (bs, 1H, H^{Ar}), 6.92 (d, ^3J (H,H) = 15.5 Hz, 1H, CH), 6.11 (d, ^3J (H,H) = 15.5 Hz, 1H, CH), 2.40 (s, 3H, CH_3) ppm; **^{13}C NMR** (76 MHz, $CDCl_3$): 153.2 (C^{Ar}), 145.6 (C^{Ar}), 139.8 (C^{Ar}), 134.8 (C_q, C^{Ar}), 130.6 (CH), 118.7 (CH), 93.7 (C_q, C^{Ar}), 18.9 (CH_3) ppm; **HRMS** (EI): calcd (*m/z*) for [M^+]: 276.9422; found: 276.9413.
(Z): **m.p.**$^{exp.}$ = 45-55°C; **GC-MS** (Method_1): t_R = 6.759 min; *m/z* (%): 277 (100) [M^+]; **^{1}H NMR** (300 MHz, $CDCl_3$): δ = 8.63 (d, ^4J (H,H) = 1.7 Hz, 1H, H^{Ar}), 8.58 (d, ^4J (H,H) = 1.8 Hz, 1H, H^{Ar}), 8.18 (bs, 1H, H^{Ar}), 6.45 (d, ^3J (H,H) = 11.0 Hz, 1H, CH), 6.27 (d, ^3J (H,H) = 11.0 Hz, 1H, CH), 2.45 (s, 3H, CH_3) ppm; **^{13}C NMR** (76 MHz, $CDCl_3$): 153.0 (C^{Ar}), 184.4 (C^{Ar}), 142.9 (C^{Ar}), 134.8 (C_q, C^{Ar}), 133.8 (CH), 120.0 (CH), 93.7 (C_q, C^{Ar}), 18.9 (CH_3) ppm; **HRMS** (EI): calcd (*m/z*) for [M^+]: 276.9422; found: 276.9413.

3.3.4 3-Iodo-5-(2-(methylthio)ethyl)pyridine

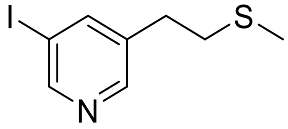

44c

A 100 mL round-bottom flask equipped with a reflux condenser was charged with 2.76 g (10.0 mmol, 1.0 eq) **44b**, 5.56 g (29.9 mmol, 3.0 eq) *p*-tosylhydrazide and 4.07 g (29.9 mmol, 3.0 eq) $NaOAc \cdot 3H_2O$ suspended in 56 mL THF. The reaction mixture was stirred at 70 °C for 8 d. During this time additional p-tosylhydrazide and $NaOAc \cdot 3H_2O$ were added in 4 equal portions to a total of 12 eq. After complete conversion was detected by GC-MS, 170 mL half-satd. $NaHCO_3$ solution and 250 mL DCM were added to the reaction mixture. The phases were separated and the aqueous layer was extracted with DCM (2 x 210 mL). The combined organic layers were washed with brine (1 x 400 mL), dried over Na_2SO_4 and the solvent was removed in vacuum. The crude product was purified via flash column chromatography (80 g SiO_2, 3.5 x 30 cm) cyclohexane/EtOAc = 5/1, R_f = 0.31, UV).

Yield: 5.16 g (89%), yellow oil, $C_8H_{10}INS$ [279.14 g/mol]

GC-MS (Method_1): t_R = 6.56 min; *m/z* (%): 279 (54) [M^+]; **^{1}H NMR** (300 MHz, $CDCl_3$): δ = 8.69 (d, ^4J (H,H) = 1.8 Hz, 1H, H^{Ar}), 8.41 (d, ^4J (H,H) = 1.6 Hz, 1H, H^{Ar}), 7.88 (bs, 1H, H^{Ar}), 2.87-2.82 (m, 2H, CH_2), 2.76-2.70 (m, 2H, CH_2), 2.12 (s, 3H, CH_3) ppm; **^{13}C NMR** (76 MHz, $CDCl_3$): 154.2 (C^{Ar}), 148.8 (C^{Ar}), 144.6 (C^{Ar}), 138.2 (C_q, C^{Ar}), 93.8 (C_q, C^{Ar}), 35.5 (CH_2), 32.8 (CH_2), 16.1 (CH_3) ppm; **HRMS** (EI): calcd (*m*/*z*) for [M^+]: 278.9579; found: 278.9576.

3.3.5 3-(2-(Methylthio)ethyl)-5-(4,4,5,5-tetramethyl-1,3,2-dioxaborolan-2-yl)pyridine

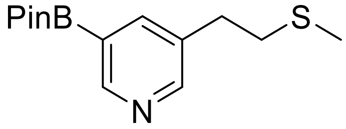

44

In a flame-dried, Ar flushed Schlenk flask 2.33 g (8.36 mmol, 1.0 eq) **44c** were dissolved in 30 mL abs. THF. The pale-yellow solution was cooled to -78 °C and 5.48 mL (10.03 mmol, 1.2 eq) *i*PrMgCl·LiCl solution (1.83M in THF) were added. The reaction mixture was stirred at -78 °C until full metal-halide exchange was detected via GC-MS (2 h). Then, 2.6 mL (12.5 mmol, 1.5 eq) PinBO*i*Pr were added and the reaction mixture was allowed to warm to RT overnight. The reaction was quenched by addition of 50 mL satd. NH_4Cl solution and extracted with DCM (3x 100 mL). The combined organic layers were dried over Na_2SO_4, filtered and concentrated under reduced pressure. The crude product was purified via Kugelrohr-distillation (165 °C, $8 \cdot 10^{-3}$ mbar).

Yield: 2.04 g (88%), colorless oil, $C_{14}H_{22}BNO_2$ [279.20 g/mol]

GC-MS (Method_1): t_R = 7.25 min; *m/z* (%): 279 (75) [M^+]; **^{1}H NMR** (300 MHz, $CDCl_3$): δ = 8.80 (d, ^4J (H,H) = 1.3 Hz, 1H, H^{Ar}), 8.53 (d, ^4J (H,H) = 2.2 Hz, 1H, H^{Ar}), 7.89 (bs, 1H, H^{Ar}), 2.91-2.86 (m, 2H, CH_2), 2.77-2.72 (m, 2H, CH_2),), 2.12 (s, 3H, CH_3), 1.35 (s, 12H, CH_3) ppm; **^{13}C NMR** (76 MHz, $CDCl_3$, APT): 153.7 (C^{Ar}), 152.4 (C^{Ar}), 142.2 (C^{Ar}), 135.1 (C_q, C^{Ar}), 84.4 (C_q), 35.5 (CH_2), 33.1 (CH_2), 25.0 (CH_3), 15.8 (CH_3) ppm;[2] **HRMS** (EI): calcd (*m*/*z*) for [M^+]: 279.1467; found: 279.1474.

[2] Signal for the quaternary *ipso*-pyridine carbon (C_q; C^{Ar}) at the boronic acid pinacol ester function was not observed.

3.3.6 3-(2-(1,3-Dioxolan-2-yl)ethyl)-5-bromopyridine

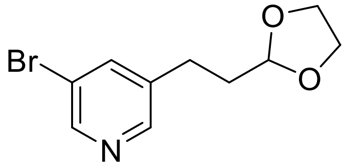

45a

A Schlenk flask was charged with 2.94 g (45.0 mmol, 1.5 eq) Zn-powder. The flask was evacuated and heated to 70 °C for 30 min. After cooling to RT the flask was flushed with argon and evacuated again for three times. The Zn-powder was suspended in 20 mL abs., degassed DMA and 189 mg (750 µmol, 3 mol%) I_2 were added. The resulting mixture was heated to 70 °C and stirred until discoloration (5 min) and 3.60 mL (30 mmol, 1.0 eq) 2-(2-bromoethyl)-1,3-dioxolane were added. The reaction mixture was stirred at 70 °C for another 16 h under Ar atmosphere. After cooling to RT the suspension was filtered into another flame dried Schlenk flask under argon.

A flame dried Schlenk flask was charged with 2.97 g (16.8 mmol, 1.0 eq) 3,5-dibromopyridine and 387 mg (336 µmol, 2 mol%) $Pd(PPh_3)_4$ which were dissolved in 20 mL abs., degassed THF. Then 21 mL (0.80M; 16.8 mmol, 1.0 eq) (2-(1,3-dioxolan-2-yl)ethyl)zinc(II) bromide solution in DMA were added. The reaction mixture was stirred at 45 °C until full conversion was detected by GC-MS (3 h). The reaction was quenched by the addition of 100 mL satd. NH_4Cl solution. The mixture was extracted with DCM (2 x 150 mL). The combined organic layers were dried over Na_2SO_4, filtered and the solvent was removed under reduced pressure. The crude product was purified via flash column chromatography (120 g SiO_2, 4 x 18 cm, eluent: cyclohexane/EtOAc = 7/2 → 5/2).

Yield: 2.15 g (50%) colorless crystals, $C_{10}H_{12}BrNO_2$ [258.12 g/mol]

TLC R_f = 0.37 (cyclohexane/EtOAc = 2/1, UV and CAM); **m.p.**$^{exp.}$ = 39-40 °C; **GC-MS** (Method_1): t_R = 6.39 min; *m/z* (%): 257 [M^+], 259 [M^+], 184 [M^+–$C_3H_5O_2$]; **^{1}H NMR** (300 MHz, $CDCl_3$): δ = 8.50 (d, ^4J (H,H) = 1.8 Hz, 1H, H^{Ar}), 8.38 (s, 1H, H^{Ar}), 7.68 (s, 1H, H^{Ar}), 4.89 (t, ^3J (H,H) = 4.4 Hz, 1H, CH), 4.05-3.78 (m, 4H, CH_2), 2.73 (t, ^3J (H,H) = 16.1 Hz, 2H, CH_2), 2.03-1.91 (m, 2H, CH_2) ppm; **^{13}C NMR** (75.53 MHz, $CDCl_3$, APT): δ = 148.7 (C^{Ar}), 148.2 (C^{Ar}), 139.0 (C_q, C^{Ar}), 138.6 (C^{Ar}), 120.7 (C_q, C^{Ar}), 103.3 (CH), 65.2 (CH_2), 34.9 (CH_2), 26.8 (CH_2) ppm; **HRMS** (EI): calcd (*m/z*) for [M^+–H]: 257.0051; found: 257.0054.

3.3.7 3-((5-Bromopyridin-3-yl)methyl)-1H-indole

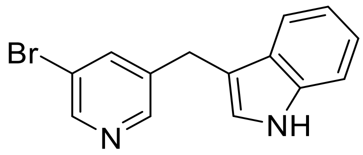

45b

In a 250 mL round-bottom flask 4.00 g (15.5 mmol, 1.0 eq) **45a** were dissolved in a mixture of 3.04 mL (31.0 mmol, 2.0 eq) phenylhydrazine, 12 mL H_2SO_4 (96% w/w) and 120 mL THF. The orange solution was stirred at 80 °C for 4 h. The reaction mixture was cooled to RT and neutralized with 200 mL satd. Na_2CO_3 solution. The neutral solution was extracted with DCM (1 x 100 mL, 2 x 50 mL). The combined organic layers were dried over Na_2SO_4 and concentrated under reduced pressure. The orange oil was purified via flash column chromatography (90 g SiO_2, 4 x 18 cm, eluent: cyclohexane/EtOAc = 4/1 → 3/1), followed by recrystallization from cyclohexane/EtOAc = 8/1 (90 mL).

Yield: 2.25 g (51%), pale orange powder, $C_{14}H_{11}BrN_2$ [287.15 g/mol]

TLC R_f = 0.38 (cyclohexane/EtOAc = 2/1, UV and CAM); **m.p.**$^{exp.}$ = 157-158 °C; **GC-MS** (Method_1): t_R = 8.29 min; *m/z* (%): 288 [M^+], 286 [M^+], 207 [M^+–Br], 155 [M^+–C_5H_3BrN], 130 [M^+–C_9H_8N]; **^{1}H NMR** (300 MHz, $CDCl_3$): δ = 8.52 (s, 2H, H^{Ar}), 8.24 (bs, 1H, NH), 7.70 (s, 1H, H^{Ar}), 7.47 (d, ^3J (H,H) = 7.9 Hz, 1H, H^{Ar}), 7.38 (d, ^3J (H,H) = 8.1 Hz, 1H, H^{Ar}), 7.22 (t, ^3J (H,H) = 7.5 Hz, 1H, H^{Ar}), 7.11 (t, ^3J (H,H) = 7.4 Hz, 1H, H^{Ar}), 6.97 (s, 1H, H^{Ar}), 4.10 (s, 2H, CH_2) ppm; **^{13}C NMR** (75.53 MHz, $CDCl_3$, APT): δ = 148.6 (C^{Ar}), 148.2 (C^{Ar}), 138.9 (C^{Ar}), 138.8 (C_q, C^{Ar}), 136.6 (C_q, C^{Ar}), 127.0 (C_q, C^{Ar}), 122.7 (C^{Ar}), 122.6 (C^{Ar}), 120.9 (C_q, C^{Ar}), 119.9 (C^{Ar}), 118.8 (C^{Ar}), 113.7 (C_q, C^{Ar}), 111.5 (C^{Ar}), 28.6 (CH_2) ppm; **HRMS** (EI): calcd (*m/z*) for [M^+–H]: 286.0106; found: 286.0106.

3.3.8 3-((5-(4,4,5,5-Tetramethyl-1,3,2-dioxaborolan-2-yl)pyridin-3-yl)methyl)-1H-indole

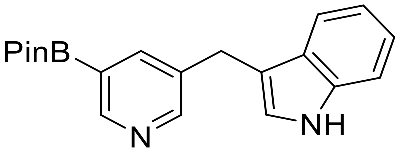

45

A flame dried Schlenk flask was charged with 1.86 g (7.34 mmol, 1.1 eq) B_2Pin_2, 285 mg (50.0 µmol, 5 mol%) Pd(dppf)Cl_2, 891 mg (9.09 mmol, 1.3 eq) KOAc and 2.00 g (6.99 mmol, 1.0

eq) 3-((5-bromopyridin-3-yl)methyl)-1*H*-indole (**45b**). After drying in vacuum for 30 min, 22 mL abs., degassed 1,4-dioxane were added and the reaction mixture was stirred for 16 h at 80 °C. The black suspension was cooled to RT, diluted with 10 mL MeOH and 100 mL satd. NH_4Cl solution. The resulting mixture was extracted with EtOAc (1 x 100 mL, 2 x 50 mL). The combined organic layers were dried over Na_2SO_4, filtered and concentrated under reduced pressure to dryness. The residue was suspended in 100 mL EtOAc, filtered and the filter cake was washed with 50 mL EtOAc. The filtrate was concentrated to dryness using a rotary evaporator. The residue was dissolved in 8 mL DCM and slowly dropped into vigorously stirred 400 mL *n*-pentane. The resulting suspension was filtered and the collected solid was dried under reduced pressure.

Yield: 2.16 g (93%), pale grey powder, $C_{20}H_{23}BN_2O_2$ [334.23 g/mol]

m.p.$^{exp.}$ = 53-55°C; **GC-MS** (Method_1): t_R = 9.91 min; *m/z* (%): 334 [M^+], 207 [M^+–$C_6H_{12}BO_2$], 130 [M^+–$C_{11}H_{15}BO_2$]; **^{1}H NMR** (300 MHz, $CDCl_3$): δ = 8.81 (s, 1H, H^{Ar}), 8.64 (s, 1H, H^{Ar}), 8.34 (bs, 1H, NH), 8.00 (s, 1H, H^{Ar}), 7.51 (d, ^3J (H,H) = 7.7 Hz, 1H, H^{Ar}), 7.35 (d, ^3J (H,H) = 8.0 Hz, 1H, H^{Ar}), 7.18 (t, ^3J (H,H) = 7.4 Hz, 1H, H^{Ar}), 7.08 (t, ^3J (H,H) = 7.3 Hz, 1H, H^{Ar}), 6.88 (s, 1H, H^{Ar}), 4.09 (s, 2H, CH_2) 1.34 (s, 12H, CH_3) ppm; **^{13}C NMR** (75.53 MHz, $CDCl_3$, APT): δ = 153.2 (C^{Ar}), 152.4 (C^{Ar}), 142.7 (C^{Ar}), 136.6 (C_q, C^{Ar}), 136.0 (C_q, C^{Ar}), 127.2 (C_q, C^{Ar}), 122.7 (C^{Ar}), 122.3 (C^{Ar}), 119.6 (C^{Ar}), 119.0 (C^{Ar}), 114.8 (C_q, C^{Ar}), 111.3 (C^{Ar}), 84.3 (C_q), 29.1 (CH_2), 25.0 (CH_3) ppm;[3] **HRMS** (MALDI): calcd for [M^++H]: 335.1935; found: 335.1934.

3.3.9 (5-Bromopyridin-3-yl)methanol

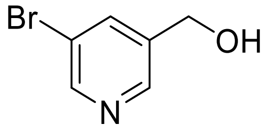

46a

In a flame dried and nitrogen flushed 500 mL two-neck round-bottom flask, equipped with an addition funnel and N_2-inlet 3.53 g (46.54 mmol, 2.0 eq) $LiAlH_4$ were suspended in 84 mL abs. THF and cooled to -78 °C in a dry ice/acetone bath. Through the addition funnel a solution of 10.0 g (46.54 mmol, 1.0 eq) methyl 5-bromonicotinate dissolved in 84 mL abs. THF was added dropwise to the reaction mixture over a period of 20 min. After stirring the suspension at -78 °C

[3] Signal for the quaternary *ipso*-pyridine carbon (C_q; C^{Ar}) at the boronic acid pinacol ester function was not observed.

for 60 min the reaction was quenched by adding 12.5 mL EtOAc. After the consecutive addition of 3.53 mL H_2O, 3.53 mL 15% NaOH and 10.59 mL H_2O the mixture was allowed to warm up to RT and was stirred vigorously for 60 min. The reaction was dried over anhydrous $MgSO_4$ and filtrated through a glass frit. The solvent was removed under reduced pressure to give a brown oil. The crude product was used without further purification. An analytical sample was purified via flash column chromatography (cyclohexane/EtOAc = 4/1, R_f = 0.17, CAM).

Yield: 8.26 g (94%), brown oil, C_6H_6BrNO [188.02 g/mol]

TLC: R_f = 0.17 (cyclohexane/EtOAc = 4/1, UV and CAM); **GC-MS** (Method_1): t_R = 5.44 min; *m/z* (%): 189 (80) [M^+], 187 (82) [M^+]; **^{1}H NMR** (300 MHz, $CDCl_3$): δ = 8.57 (d, ^4J (H,H) = 1.8 Hz, 1H, H^{Ar}), 8.46 (bs, 1H, H^{Ar}), 7.89 (bs, 1H, H^{Ar}), 4.72 (s, 2H, CH_2), 2.70 (bs, 1H, OH) ppm; **^{13}C NMR** (76 MHz, $CDCl_3$, APT): δ = 150.0 (C^{Ar}), 145.7 (C^{Ar}), 138.2 (C_q, C^{Ar}), 137.5 (C^{Ar}), 121.1 (C_q, C^{Ar}), 61.9 (CH_2) ppm.

Analytical data are in accordance with those reported.[14]

3.3.10 (5-Iodopyridin-3-yl)methanol

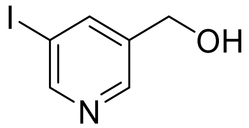

46b

In a flame-dried, Ar flushed Schlenk flask equipped with stirring bar 4.02 g (21.39 mmol, 1.0 eq) **46a**, 6.41 g (42.78 mmol, 2.0 eq) NaI, 407 mg (2.14 mmol, 10 mol%) CuI and 230 µL (2.14 mmol, 10 mol%) *N*,*N'*-dimethylethylenediamine were suspended in 36 mL abs., degassed 1,4-dioxane. The reaction mixture was stirred at 110 °C for 24 h and full conversion was detected via GC-MS. The reaction mixture was cooled to RT and 100 mL satd. NH_4Cl solution were added. After filtration, the filtrate was transferred to a separatory funnel and the aqueous layer was extracted with DCM (3x 100 mL). The combined organic layers were dried over Na_2SO_4, filtered and concentrated under reduced pressure. The crude product was purified via flash column chromatography (130 g SiO_2, 6.5 x 9 cm, cyclohexane/EtOAc = 2/1, R_f = 0.24, CAM).

Yield: 3.14 g (79%), colorless solid, C_6H_6INO [235.02 g/mol]

TLC: R_f = 0.24 (cyclohexane/EtOAc = 2/1, UV and CAM); **GC-MS** (Method_1): t_R = 5.60 min; *m/z* (%): 235 (100) [M^+], 206 (36) [M^+−CH_2OH], 108 (14) [M^+−I]; **m.p.**$^{exp.}$ = 63-65 °C; **^{1}H NMR** (300 MHz, $CDCl_3$): δ = 8.69 (d, ^4J (H,H) = 1.4 Hz, 1H, H^{Ar}), 8.46 (s, 1H, H^{Ar}), 8.07 (s, 1H, H^{Ar}), 4.67 (s, 2H, CH_2), 2.78 (bs, 1H, OH) ppm; **^{13}C NMR** (76 MHz, $CDCl_3$, APT): δ = 154.8 (C^{Ar}), 146.7 (C^{Ar}), 143.2 (C^{Ar}), 138.6 (C_q, C^{Ar}), 93.6 (C_q, C^{Ar}), 61.9 (CH_2) ppm.

3.3.11 3-(((tert-Butyldiphenylsilyl)oxy)methyl)-5-iodopyridine

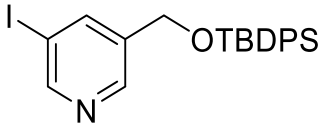

46c

In a 50 mL one-neck round-bottom-flask 1.00 g (4.17 mmol, 1.0 eq) **46b** and 710 mg (10.42 mmol, 2.5 eq) imidazole were dissolved in 19 mL DCM. After addition of 1.0 mL (3.75 mmol, 0.9 eq) TBDPSCl a white precipitate started forming. The reaction mixture was stirred overnight and transferred into a separation funnel. After addition of 30 mL NaOH (3M) the phases were separated and the aqueous phase extracted with DCM (1 x 30 mL). The combined organic phases were washed with satd. NaCl solution (1 x 40 mL), dried over Na_2SO_4 and concentrated in vacuum. The crude product was purified via flash column chromatography (100 g SiO_2, 6 x 9 cm, cyclohexane/EtOAc = 20/1, R_f = 0.17, UV).

Yield: 1.47 g (83%), colorless oil, $C_{22}H_{24}INOSi$ [473.43 g/mol]

GC-MS (Method_1): t_R = 9.97 min; *m/z* (%): 416 (100) [M^+−*t*Bu]; **^{1}H NMR** (300 MHz, $CDCl_3$): δ = 8.72 (d, ^4J (H,H) = 1.6 Hz, 1H, H^{Ar}), 8.47 (bs, 1H, H^{Ar}), 7.94 (bs, 1H, H^{Ar}), 7.67-7.65 (m, 4H, H^{Ar}), 7.48-7.37 (m, 6H, H^{Ar}), 4.70 (s, 2H, CH_2), 1.10 (s, 9H, CH_3) ppm; **^{13}C NMR** (76 MHz, $CDCl_3$): δ = 154.4 (C^{Ar}), 146.5 (C^{Ar}), 142.2 (C^{Ar}), 138.3 (C_q, C^{Ar}), 135.6 (C^{Ar}), 132.8 (C_q, C^{Ar}), 130.0 (C^{Ar}), 127.9 (C^{Ar}), 93.3 (C_q, C^{Ar}), 62.8 (CH_2), 26.8 (CH_3), 19.3 (C_q) ppm; **HRMS** (EI): calcd (*m*/*z*) for [M^+]: 473.0672; found: 473.0697.

3.3.12 3-(((tert-Butyldiphenylsilyl)oxy)methyl)-5-(4,4,5,5-tetramethyl-1,3,2-dioxaborolan-2-yl)pyridine

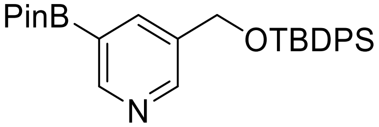

46

In a flame-dried, Ar flushed Schlenk flask 1.19 g (2.51 mmol, 1.0 eq) **46c** were dissolved in 13 mL abs. THF. The colourless solution was cooled to -78 °C and 1.8 mL (3.0 mmol, 1.2 eq) *i*PrMgCl·LiCl solution (1.71M in THF) were added. The reaction was stirred at -78 °C until full metal-halide exchange was detected via GC-MS (2 h). Then, 0.77 mL (3.8 mmol, 1.5 eq) PinBO*i*Pr were added and the reaction mixture was allowed to warm ro RT overnight. 30 mL satd. NH_4Cl solution were added and the aqueous layer was extracted with DCM (3x 40 mL) The combined organic layers were dried over Na_2SO_4, filtered and concentrated under reduced pressure. The crude product was purified via sublimation (160 °C, $3 \cdot 10^{-3}$ mbar).

Yield: 0.94 g (79%), colorless wax like solid, $C_{28}H_{36}BNO_3Si$ [473.50 g/mol]

GC-MS (Method_1): t_R = 11.48 min; *m/z* (%): 416 (100) [M^+−*t*Bu]; **^{1}H NMR** (300 MHz, $CDCl_3$): δ = 8.84 (bs, 1H, H^{Ar}), 8.70 (bs, 1H, H^{Ar}), 7.92 (bs, 1H, H^{Ar}), 7.69-7.67 (m, 4H, H^{Ar}), 7.44-7.36 (m, 6H, H^{Ar}), 4.75 (s, 2H, CH_2), 1.35 (s, 12H, CH_3), 1.09 (s, 9H, CH_3) ppm; **^{13}C NMR** (76 MHz, $CDCl_3$): δ = 154.3 (C^{Ar}), 150.7 (C^{Ar}), 140.5 (C^{Ar}), 135.7 (C^{Ar}) 135.4 (C_q, C^{Ar}), 133.3 (C_q, C^{Ar}), 130.0 (C^{Ar}), 127.9 (C^{Ar}); 84.3 (C_q), 63.8 (CH_2), 27.0 (CH_3), 25.0 (CH_3), 19.4 (C_q) ppm;[4] **HRMS** (EI): calcd (*m*/*z*) for [M-H^+]: 472.2485; found: 472.2533.

3.3.13 3-Benzyl-5-bromopyridine

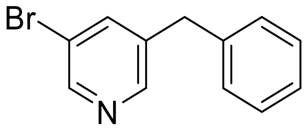

47a

A two-neck round-bottom flask was charged with Zn-powder 1.15 g (17.5 mmol, 4.4 eq) Zn-powder. Subsequently the flask was evacuated, heated with a heat gun and after cooling to RT back flushed with argon three times. The Zn was suspended in 3 mL abs. THF and 80 µL (0.88 mmol, 22 mol%) 1,2-dibromoethane was added. The mixture was heated to reflux temperature and cooled again to RT for three times. After the third cycle, 110 µL (0.88 mmol, 22 mol%) TMSCl was added and stirred at RT for 10 min. A solution of 1.00 mL (8.77 mmol, 2.2 eq) benzylbromide in 2 mL

[4] Signal for the quaternary *ipso*-pyridine carbon (C_q; C^{Ar}) at the boronic acid pinacol ester function was not observed.

abs. THF was added slowly via a dropping funnel. A water bath was used to keep the reaction at RT. When addition was finished the reaction was stirred at RT for another 2 h.

A flame dried and argon flushed Schlenk-flask was charged with 950 mg (4.01 mmol, 1.0 eq) 3,5-dibromopyridine, 32.3 mg (40.1 µmol, 1 mol%) $PdCl_2$(dppf) and 5 mL abs. THF. The previously prepared organozinc solution was added to this orange suspension and the brown solution was stirred at 70 °C. At 93% conversion (2 h) already 5% dibenzylation was detected, the reaction was cooled to RT, and the catalyst was removed by filtration through a pad of silica gel and the product was eluted with 100 mL EtOAc. The solvent was removed under reduced pressure and the brown oil was purified via flash column chromatography (100 g SiO_2, 2.5 x 20 cm, eluent: cyclohexane/EtOAc = 10/1 changing to cyclohexane/EtOAc = 5/1 when the product starts eluting, R_f = 0.40, UV and CAM).

Yield: 620 mg (61%), colorless oil, $C_{12}H_{10}BrN$ [248.12 g/mol]

TLC: R_f = 0.40 (cyclohexane/EtOAc = 5/1, UV and CAM); **GC-MS** (Method_1): t_R = 6.46 min; *m/z* (%): 247 (100) [M^+], 167 (100) [M^+−Br], 91 (36) [M^+−C_6H_5Br]; **^{1}H NMR** (300 MHz, $CDCl_3$): δ = 8.44 (d, ^4J (H,H) = 1.9 Hz, 1H, H^{Ar}), 8.34 (s, 1H, H^{Ar}), 7.53 (s, 1H, H^{Ar}), 7.25-7.15 (m, 3H, H^{Ar}), 7.10 (d, ^3J (H,H) = 7.0 Hz, 2H, H^{Ar}), 3.88 (s, 2H, CH_2) ppm; **^{13}C NMR** (76 MHz, $CDCl_3$, APT): δ = 148.9 (C^{Ar}), 148.4 (C^{Ar}), 139.0 (C_q, C^{Ar}) 138.9 (C^{Ar}), 138.5 (C_q, C^{Ar}), 129.0 (C^{Ar}), 126.9 (C^{Ar}), 120.9 (C_q, C^{Ar}), 38.8 (CH_2) ppm.

3.3.14 3-Benzyl-5-(4,4,5,5-tetramethyl-1,3,2-dioxaborolan-2-yl)pyridine

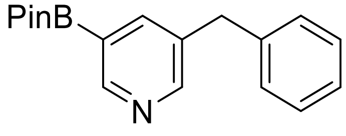

47

In a flame-dried, Ar flushed Schlenk flask 860 mg (3.47 mmol, 1.0 eq) 3-benzyl-5-bromopyridine (**47a**) were dissolved in 10 mL absolute THF. The colourless solution was cooled to 0 °C and 1.80 mL (3.81 mmol, 1.1 eq) *i*PrMgCl.LiCl (1.5M in THF) were added. The reaction was stirred at 0 °C until full metal-halide exchange was detected via GC-MS (3 h). Then, (3.81 mmol, 1.1 eq) 780 µL PinBO*i*Pr were added and the reaction mixture was stirred at RT overnight. 20 mL satd. NH_4Cl solution were added and the aqueous layer was extracted with DCM (3x 20

mL). The combined organic layers were dried over Na_2SO_4, filtered and concentrated under reduced pressure. The crude product was purified via recrystallization from pentane.

Yield: 187 mg (18%), colorless powder, $C_{18}H1_{22}BNO_2$ [295.19 g/mol]

m.p.$^{exp.}$ = 95-97 °C; **GC-MS** (Method_1): t_R = 7.53 min; *m/z* (%): 295 (97) [M^+], 280 (100) [M^+−CH_3], 238 (55) [M^+−C_4H_{13}], 194 (80) [M^+−$C_6H_{13}O$]; **^{1}H NMR** (300 MHz, $CDCl_3$): δ = 8.80 (d, ^4J (H,H) = 1.3 Hz, 1H, H^{Ar}), 8.53 (d, ^4J (H,H) = 2.3 Hz, 1H, H^{Ar}), 7.99 (bs, 1H, H^{Ar}), 7.33-7.16 (m, 5H, H^{Ar}), 3.99 (s, 2H, CH_2), 1.34 (s, 12H, CH_3) ppm; **^{13}C NMR** (76 MHz, $CDCl_3$): δ = 152.0 (C^{Ar}), 150.9 (C^{Ar}), 144.0 (C^{Ar}), 139.6 (C_q, C^{Ar}), 136.6 (C_q, C^{Ar}), 129.0 (C^{Ar}), 128.9 (C^{Ar}), 126.8 (C^{Ar}), 84.6 (C_q), 39.2 (CH_2), 25.0 (CH_3) ppm;[5] **HRMS** (EI): calcd (*m/z*) for [M^+]: 295.1747; found: 295.1749.

Analytical data are in accordance with those reported.[13]

3.4 Leu-Trp-Met

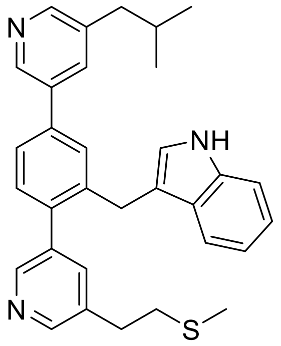

48

The first coupling step was performed according to general procedure 3.1 from 104 mg (400 µmol, 1.0 eq) pyridine building block **43**, 110 mg (800 µmol, 2.0 eq) K_2CO_3, 14.6 mg (20.0 µmol, 5 mol%) $PdCl_2$(dppf) and 165 mg (400 µmol, 1.0 eq) core building block **38** in 3 mL abs., degassed DMF. The crude product was purified via column chromatography (5 g SiO_2, 1.5 x 10 cm, eluent: cyclohexane/EtOAc = 3/1 → 2/1, fraction size: 2.5 mL) to isolate 105 mg (63%) diaryl intermediate as a brown oil.

---

[5] Signal for the quaternary *ipso*-pyridine carbon (C_q; C^{Ar}) at the boronic acid pinacol ester function was not observed.

The second coupling step was performed according to general procedure 3.2 from 50 mg (180 µmol, 1.1 eq) pyridine building block **44**, 112 mg (344 µmol, 2.0 eq) Cs_2CO_3, 6.3 mg (8.6 µmol, 5 mol%) $PdCl_2$(dppf) and 72 mg (172 µmol, 1.0 eq) previously prepared intermediate in 2 mL abs., degassed DMF. The crude product was purified via column chromatography (5 g SiO_2, 1.8 x 22 cm, eluent: cyclohexane/EtOAc = 1/4, fraction size: 2.5 mL).

Yield: 75 mg (57% over 2 steps), yellow oil, $C_{32}H_{33}N_3S$ [491.70 g/mol]

HPLC-MS (Poroshell, ESI+, MV_general): t_R = 5.26 min, *m/z* = 492 [M+H^+]; **^{1}H-NMR** (500 MHz, $CDCl_3$: δ = 8.67 (d, ^4J (H,H) = 1.8 Hz, 1H, H^{Ar}), 8.50 (d, ^4J (H,H) = 1.5 Hz, 1H, H^{Ar}), 8.43 (d, ^4J (H,H) = 1.5 Hz, 1H, H^{Ar}), 8.38 (d, ^4J (H,H) = 1.4 Hz, 1H, H^{Ar}), 8.10 (bs, 1H, NH), 7.65 – 7.63 (m, 2H, H^{Ar}), 7.54 (dd, ^3J (H,H) = 7.9 Hz, ^4J (H,H) = 1.7 Hz, 1H, H^{Ar}), 7.43 (s, 1H, H^{Ar}), 7.38 – 7.31 (m, 3H, H^{Ar}), 7.18 – 7.13 (m, 1H, H^{Ar}), 7.06 – 7.01 (m, 1H, H^{Ar}), 6.68 (s, 1H, H^{Ar}), 4.11 (s, 2H, CH_2), 2.79 (t, ^3J (H,H) = 7.6 Hz, 2H, CH_2), 2.58 – 2.52 (m, 4H, CH_2), 2.01 (s, 3H, CH_3), 1.95 – 1.86 (m, 1H, CH), 0.94 (d, ^3J (H,H) = 6.6 Hz, 6H, CH_3) ppm; **^{13}C-NMR** (125 MHz, $CDCl_3$): δ = 149.5 (C^{Ar}), 148.7 (C^{Ar}), 147.9 (C^{Ar}), 145.9 (C^{Ar}), 139.6 (C_q, C^{Ar}), 138.1 (C_q, C^{Ar}), 138.0 (C_q, C^{Ar}), 136.9 (C_q, C^{Ar}), 136.6 (C^{Ar}), 136.6 (C_q, C^{Ar}), 136.5 (C_q, C^{Ar}), 135.7 (C_q, C^{Ar}), 135.2 (C_q, C^{Ar}), 135.0 (C^{Ar}), 131.0 (C^{Ar}), 129.3 (C^{Ar}), 127.1 (C_q, C^{Ar}), 125.3 (C^{Ar}), 122.7 (C^{Ar}), 122.4 (C^{Ar}), 119.6 (C^{Ar}), 118.9 (C^{Ar}), 116.0 (C_q, C^{Ar}), 111.3 (C^{Ar}), 42.5 (CH_2), 35.3 (CH_2), 32.7 (CH_2), 30.2 (CH), 29.8 (CH_2), 22.4 (CH_3), 15.7 (CH_3) ppm; **HRMS** (MALDI TOF): calcd. *(m/z)* for [M^++H]: 492.2473; found: 492.2469.

3.5 Leu-Val-Trp

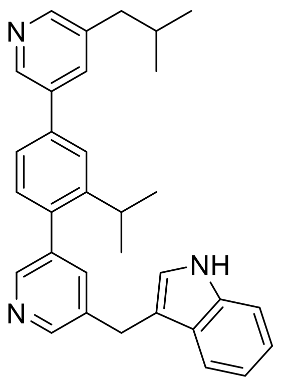

49

The first coupling step was performed according to general procedure 3.1 from 104 mg (400 µmol, 1.0 eq) pyridine building block **43**, 110 mg (800 µmol, 2.0 eq) K_2CO_3, 14.6 mg (20.0 µmol,

5 mol%) $PdCl_2$(dppf) and 130 mg (400 µmol, 1.0 eq) core building block **7** in 3 mL abs., degassed DMF. The crude product was purified via column chromatography (5 g SiO_2, 1.5 x 10 cm, eluent: cyclohexane/EtOAc = 3/1 → 2/1, fraction size: 2.5 mL) to isolate 78 mg (59%) diaryl intermediate as a brown oil.

The second coupling step was performed according to general procedure 3.2 from 53 mg (158 µmol, 1.1 eq) pyridine building block **45**, 97 mg (298 µmol, 2.0 eq) Cs_2CO_3, 5.4 mg (7.4 µmol, 5 mol%) $PdCl_2$(dppf) and 49 mg (148 µmol, 1.0 eq) previously prepared intermediate in 2 mL abs., degassed DMF. The crude product was purified via column chromatography (5 g SiO_2, 1.8 x 22 cm, eluent: cyclohexane/EtOAc = 2/3 → 1/2, fraction size: 2.5 mL).

Yield: 44 mg (38% over 2 steps), yellow oil, $C_{32}H_{33}N_3$ [459.64 g/mol]

HPLC-MS (Poroshell, ESI+, MV_general): t_R = 5.30 min, *m/z* = 460 [M+H^+]; **^{1}H-NMR** (500 MHz, $CDCl_3$: δ = 8.69 (d, ^4J (H,H) = 2.1 Hz, 1H, H^{Ar}), 8.64 (d, ^4J (H,H) = 1.9 Hz, 1H, H^{Ar}), 8.46 – 8.37 (m, 2H, H^{Ar}, NH), 8.41 (d, ^4J (H,H) = 1.8 Hz, 1H, H^{Ar}), 7.65 (s, 1H, H^{Ar}), 7.54 (d, ^4J (H,H) = 1.6 Hz, 1H, H^{Ar}), 7.50 – 7.47 (m, 2H, H^{Ar}), 7.41 (dd, ^3J (H,H) = 7.9 Hz, ^4J (H,H) = 1.8 Hz, 1H, H^{Ar}), 7.37 (d, ^3J (H,H) = 8.1 Hz, 1H, H^{Ar}), 7.23 (d, ^3J (H,H) = 7.9 Hz, 1H, H^{Ar}), 7.21-7.18 (m, 1H, H^{Ar}), 7.10 – 7.07 (m, 1H, H^{Ar}), 7.03 (d, ^4J (H,H) = 1.9 Hz, 1H, H^{Ar}), 4.21 (s, 2H, CH_2), 2.95 – 2.90 (m, 1H, CH), 2.57 (d, ^3J (H,H) = 7.2 Hz, 2H, CH_2), 1.97 – 1.91 (m, 1H, CH), 1.11 (d, ^3J (H,H) = 6.9 Hz, 6H, CH_3), 0.96 (d, ^3J (H,H) = 6.6 Hz, 6H, CH_3) ppm; **^{13}C-NMR** (125 MHz, $CDCl_3$): δ = 149.4 (C^{Ar}), 148.7 (C^{Ar}), 147.7 (C_q, C^{Ar}), 147.5 (C^{Ar}), 145.9 (C^{Ar}), 138.2 (C_q, C^{Ar}), 137.2 (C_q, C^{Ar}), 136.9 (C^{Ar}), 136.8 (C_q, C^{Ar}), 136.8 (C_q, C^{Ar}), 136.7 (C_q, C^{Ar}), 136.2 (C_q, C^{Ar}), 136.2 (C_q, C^{Ar}), 135.1 (C^{Ar}), 130.8 (C^{Ar}), 127.2 (C_q, C^{Ar}), 124.8 (C^{Ar}), 124.6 (C^{Ar}), 122.7 (C^{Ar}), 122.4 (C^{Ar}), 119.7 (C^{Ar}), 119.0 (C^{Ar}), 114.2 (C_q, C^{Ar}), 111.4 (C^{Ar}), 42.5 (CH_2), 30.2 (CH), 29.7 (CH), 28.9 (CH), 24.2 (CH_3), 22.4 (CH_3) ppm; **HRMS** (MALDI TOF): calcd. *(m/z)* for [M^++H]: 460.2753; found: 460.2747.

3.6 Leu-Asn-Ser

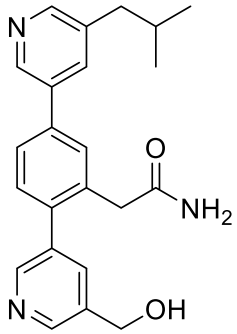

50

The first coupling step was performed according to general procedure 3.1 from 104 mg (400 µmol, 1.0 eq) pyridine building block **43**, 110 mg (800 µmol, 2.0 eq) K_2CO_3, 14.6 mg (20.0 µmol, 5 mol%) $PdCl_2$(dppf) and 136 mg (400 µmol, 1.0 eq) core building block **12** in 3 mL abs., degassed DMF. The reaction was performed at 70 °C to avoid side reactions involving the amide group. The crude product was purified via column chromatography (5 g SiO_2, 1.5 x 10 cm, eluent: DCM/MeOH = 19/1 → 9/1, fraction size: 2.5 mL) to isolate 75 mg (54%) diaryl intermediate as a colourless solid.

The second coupling step was performed according to general procedure 3.2 from 72 mg (150 µmol, 1.1 eq) pyridine building block **46**, 91 mg (280 µmol, 2.0 eq) Cs_2CO_3, 5.4 mg (7.4 µmol, 5 mol%) $PdCl_2$(dppf) and 48 mg (140 µmol, 1.0 eq) previously prepared intermediate in 1 mL abs., degassed DMF. The reaction was heated at 70 °C for 48 h to avoid side reactions involving the amide group while ensuring full deprotection of the TBDPS group. The crude product was purified via column chromatography (5 g SiO_2, 1.2 x 22 cm, eluent: EtOAc/MeOH = 10/1 → 3/1, fraction size: 2.5 mL).

Yield: 38 mg (39% over 2 steps), brown oil, $C_{23}H_{25}N_3O_2$ [375.47 g/mol]

HPLC-MS (Poroshell, ESI+, MV_general): t_R = 3.42 min, *m/z* = 376 [$M+H^+$]; **^{1}H-NMR** (300.36 MHz, MeOD: δ = 8.69 (s, 1H, H^{Ar}), 8.57 (s, 1H, H^{Ar}), 8.47 (s, 1H, H^{Ar}), 8.36 (s, 1H, H^{Ar}), 7.97 (s, 1H, H^{Ar}), 7.87 (s, 1H, H^{Ar}), 7.71 – 7.66 (m, 2H, H^{Ar}), 7.40 (d, ^3J (H,H) = 7.9 Hz, 1H, H^{Ar}), 4.74 (s, 2H, CH_2), 3.61 (s, 2H, CH_2), 2.62 (d, ^3J (H,H) = 7.1 Hz, 2H, CH_2), 2.01 – 1.94 (m, 1H, CH), 0.97 (d, ^3J (H,H) = 6.6 Hz, 6H, CH_3) ppm; **^{13}C-NMR** (75.53 MHz, MeOD): δ = 176.2 (C_q, $CONH_2$), 149.6 (C^{Ar}), 148.9 (C^{Ar}), 147.7 (C^{Ar}), 145.9 (C^{Ar}), 139.8 (C_q, C^{Ar}), 139.2 (C_q, C^{Ar}), 138.8 (C_q, C^{Ar}), 138.7 (C_q, C^{Ar}), 138.0 (C_q, C^{Ar}), 137.5 (C^{Ar}), 137.4 (C_q, C^{Ar}), 137.1 (C^{Ar}), 135.7 (C_q,

C^{Ar}), 132.2 (C^{Ar}), 130.9 (C^{Ar}), 127.1 (C^{Ar}), 62.4 (CH_2), 43.0 (CH_2), 40.7 (CH_2), 31.3 (CH), 22.5 (CH_3) ppm; **HRMS** (MALDI TOF): calcd. *(m/z)* for [M^++H]: 376.2025; found: 376.2018.

3.7 Ethyl 3-(2-(5-benzylpyridin-3-yl)-5-(5-isobutylpyridin-3-yl)phenyl)propanoate

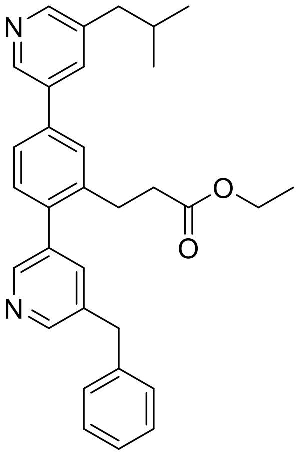

51a

The first coupling step was performed according to general procedure 3.1 from 411 mg (1.58 mmol, 1.0 eq) pyridine building block **43**, 621 mg (4.50 mmol, 3.0 eq) K_2CO_3, 61.2 mg (75.0 µmol, 5 mol%) $PdCl_2$(dppf)*DCM and 574 mg (400 µmol, 1.0 eq) core building block **30** in 3 mL abs., degassed DMF. The crude product was purified via column chromatography (25 g SiO_2, 1.5 x 10 cm, eluent: cyclohexane/EtOAc = 5/1 → 3/1, fraction size: 12 mL) to isolate 464 mg (80%) diaryl intermediate as a pale-yellow powder.

The second coupling step was performed according to general procedure 3.2 from 168 mg (570 µmol, 1.1 eq) pyridine building block **47**, 497 mg (1.53 mmol, 3.0 eq) Cs_2CO_3, 20.8 mg (25.5 µmol, 5 mol%) $PdCl_2$(dppf)*DCM and 200 mg (510 µmol, 1.0 eq) previously prepared intermediate in 2 mL abs., degassed DMF. The crude product was purified via column chromatography (25 g SiO_2, 1.8 x 22 cm, eluent: cyclohexane/EtOAc = 3/1 → 3/4, fraction size: 12 mL).

Yield: 189 mg (62% over 2 steps), colourless oil, $C_{32}H_{34}N_2O_2$ [478.62 g/mol]

HPLC-MS (Poroshell, ESI+, MV_general): t_R = 7.02 min, *m/z* = 478 [M+H^+]; **^{1}H-NMR** (300 MHz, $CDCl_3$: δ = 8.68 (d, ^4J (H,H) = 1.3 Hz, 1H, H^{Ar}), 8.53 (d, ^4J (H,H) = 0.9 Hz, 1H, H^{Ar}), 8.46 (d, ^4J (H,H) = 1.2 Hz, 1H, H^{Ar}), 8.41 (d, ^4J (H,H) = 0.6 Hz, 1H, H^{Ar}), 7.67 (s, 1H, H^{Ar}), 7.54 – 7.42 (m, 3H, H^{Ar}), 7.38 – 7.15 (m, 6H, H^{Ar}), 4.21 – 3.90 (m, 4H, CH_2), 2.94 (t, ^3J (H,H) = 7.8

Hz, 2H, CH_2), 2.56 (d, ^3J (H,H) = 7.1 Hz, 2H, CH_2), 2.43 (t, ^3J (H,H) = 7.8 Hz, 2H, CH_2), 2.05 – 1.83 (m, 1H, CH), 1.18 (t, ^3J (H,H) = 7.1 Hz, 3H, CH_2), 0.96 (d, ^3J (H,H) = 6.6 Hz, 6H, CH_3) ppm; **^{13}C-NMR** (125 MHz, $CDCl_3$): δ = 172.5 (C_q; C=O), 149.2 (C^{Ar}), 149.1 (C^{Ar}), 147.6 (C^{Ar}), 145.5 (C^{Ar}), 139.6 (C_q, C^{Ar}), 139.3 (C_q, C^{Ar}), 138.1 (C_q, C^{Ar}), 138.0 (C_q, C^{Ar}), 137.1 (C_q, C^{Ar}), 137.0 (C^{Ar}), 136.4 (C_q, C^{Ar}), 136.3 (C_q, C^{Ar}), 135.8 (C_q, C^{Ar}), 135.3 (C^{Ar}), 131.2 (C^{Ar}), 129.0 (C^{Ar}), 128.9 (C^{Ar}), 128.2 (C^{Ar}), 126.8 (C^{Ar}), 125.4 (C^{Ar}), 60.6 (CH_2), 42.5 (CH_2), 39.1 (CH_2), 35.4 (CH_2), 30.2 (CH), 28.4 (CH_2), 22.4 (CH_3), 14.3 (CH_3) ppm; **HRMS** (DI-EI): calcd. *(m/z)* for [M^+]: 478.2620; found: 478.2636.

3.8 Leu-Glu-Phe

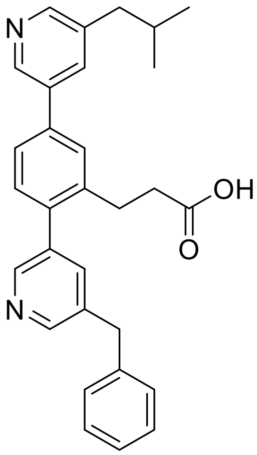

51

In a 25 mL round bottom flask 180 mg compound **51a** (375 µmol, 1 eq) were dissolved in 7 mL THF/H_2O (2/1) and 315 mg LiOH·H_2O (7.53 mmol, 20.0 eq) were added. The reaction mixture was intensively stirred at RT for 16 h. Then 7.53 mL 1M HCl (7.53 mmol, 20.0 eq) were added for neutralization and the solution was extracted with DCM (3 x 10 mL). The organic phase was dried over Na_2SO_4 and the solvent was removed under reduced pressure. The crude product was purified via semi-preparative HPLC (MV_NucleodurC18_001HCOOH_70to100).

Yield: 124 mg (74%), colourless foam, $C_{30}H_{30}N_2O_2$ [450.57 g/mol]

HPLC-MS (Poroshell, ESI+, MV_general): t_R = 7.04 min, *m/z* = 451 [$M+H^+$]; **^{1}H-NMR** (300 MHz, $CDCl_3$: δ = 8.68 (s, 1H, H^{Ar}), 8.62 – 8.45 (m, 2H, H^{Ar}), 8.38 (s, 1H, H^{Ar}), 7.71 (s, 1H, H^{Ar}), 7.61 (s, 1H, H^{Ar}), 7.52 (s, 1H, H^{Ar}), 7.46 (d, ^3J (H,H) = 6.7 Hz, 1H, H^{Ar}), 7.38 – 7.14 (m, 6H, H^{Ar}), 4.06 (s, 2H, CH_2), 2.99 (t, ^3J (H,H) = 7.1 Hz, 2H, CH_2), 2.69 – 2.41 (m, 4H, CH_2), 2.04 –

1.79 (m, 1H, CH), 0.94 (d, ^3J (H,H) = 6.6 Hz, 6H, CH_3) ppm; **^{13}C-NMR** (125 MHz, $CDCl_3$): δ = 175.4 (C_q, C=O), 148.3 (C^{Ar}), 148.0 (C^{Ar}), 146.9 (C^{Ar}), 144.9 (C^{Ar}), 139.9 (C_q, C^{Ar}), 139.5 (C_q, C^{Ar}), 137.9 (C_q, C^{Ar}), 137.8 (C^{Ar}), 137.4 (C_q, C^{Ar}), 136.9 (C_q, C^{Ar}), 136.7 (C_q, C^{Ar}), 136.1 (C_q, C^{Ar}), 135.8 (C^{Ar}), 131.2 (C^{Ar}), 129.1 (C^{Ar}), 130.0 (C^{Ar}), 128.2 (C^{Ar}), 126.8 (C^{Ar}), 125.3 (C^{Ar}), 42.4 (CH_2), 39.1 (CH_2), 35.7 (CH_2), 30.2 (CH), 28.4 (CH_2), 22.4 (CH_3) ppm; **HRMS** (MALDI TOF): calcd. *(m/z)* for [M^+]: 451.2386; found: 451.2400.

4 Literature:

[1] W. G. Kofron, L. M. Baclawski, *J. Org. Chem.* **1976**, *41*, 1879–1880.

[2] S. C. Watson, J. F. Eastham, *J. Organomet. Chem.* **1967**, *9*, 165–168.

[3] R. Joseph, E. Masson, *Org. Biomol. Chem.* **2013**, *11*, 3116–3127.

[4] F. A. Esteve-Turrillas, J. Parra, A. Abad-Fuentes, C. Agulló, A. Abad-Somovilla, J. V. Mercader, *Anal. Chim. Acta* **2010**, *682*, 93–103.

[5] S. Luliński, J. Serwatowski, M. Szczerbińska, *Eur. J. Org. Chem.* **2008**, *2008*, 1797–1801.

[6] G. Wittig, M. Schlosser, *Chem. Ber.* **1961**, *94*, 1373–1383.

[7] C. Quinet, L. Sampoux, I. E. Markó, *Eur. J. Org. Chem.* **2009**, *2009*, 1806–1811.

[8] M. Peters, M. Trobe, H. Tan, R. Kleineweischede, R. Breinbauer, *Chem. - Eur. J.* **2013**, *19*, 2442–2449.

[9] a) John Wiley & Sons Ltd. (Ed.) *e-EROS Encycl. Reagents Org. Synth.*, John Wiley & Sons, Ltd, Chichester, UK, **2001**; b) M. Trobe, R. Breinbauer, *Monatsh. Chem.* **2016**, *147*, 509–521.

[10] S. Y. Kang, M. J. Kim, J. S. Lee, J. Lee, *Bioorg. Med. Chem. Lett.* **2011**, *21*, 3759–3763.

[11] K. L. Kirk, *J. Heterocylic Chem.* **1985**, *22*, 57–59.

[12] O. Tsuge, S. Kanemasa, T. Naritomi, J. Tanaka, *Bull. Chem. Soc. Jpn.* **1987**, *60*, 1497–1504.

[13] M. Peters, M. Trobe, R. Breinbauer, *Chem. - Eur. J.* **2013**, *19*, 2450–2456.

[14] R. Heim, S. Lucas, C. M. Grombein, C. Ries, K. E. Schewe, M. Negri, U. Müller-Vieira, B. Birk, R. W. Hartmann, *J. Med. Chem.* **2008**, *51*, 5064–5074.

5 NMR

4-Bromo-2-isopropylaniline (**4**)

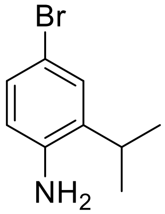

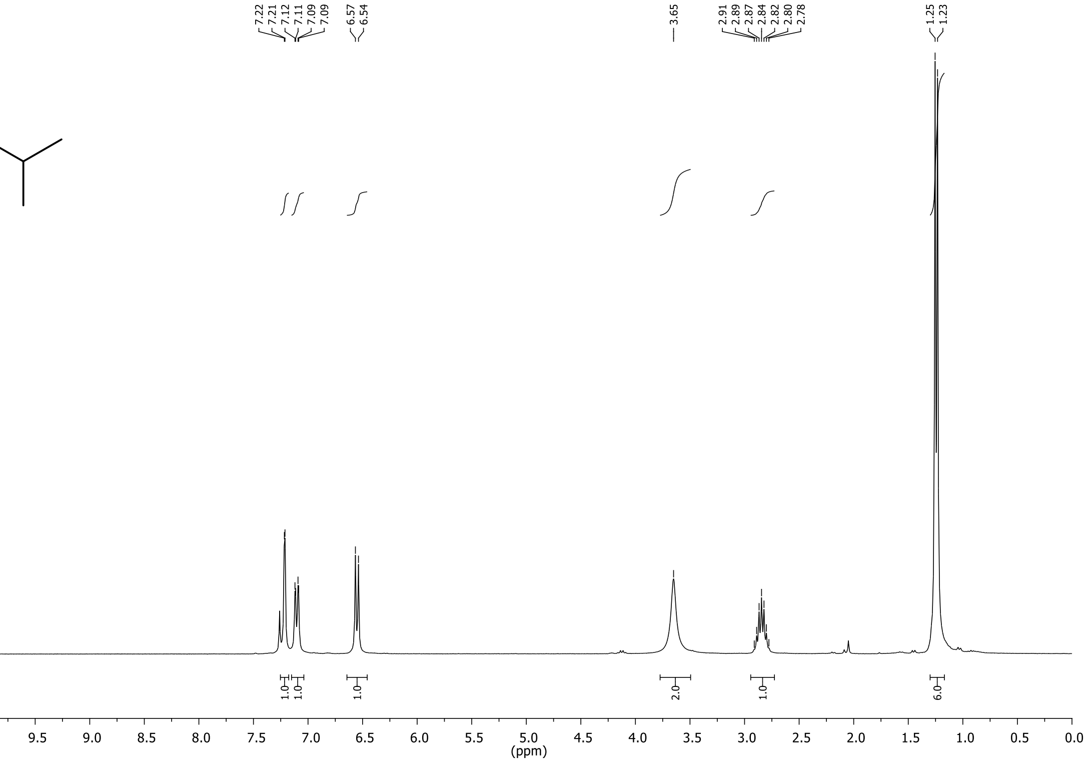

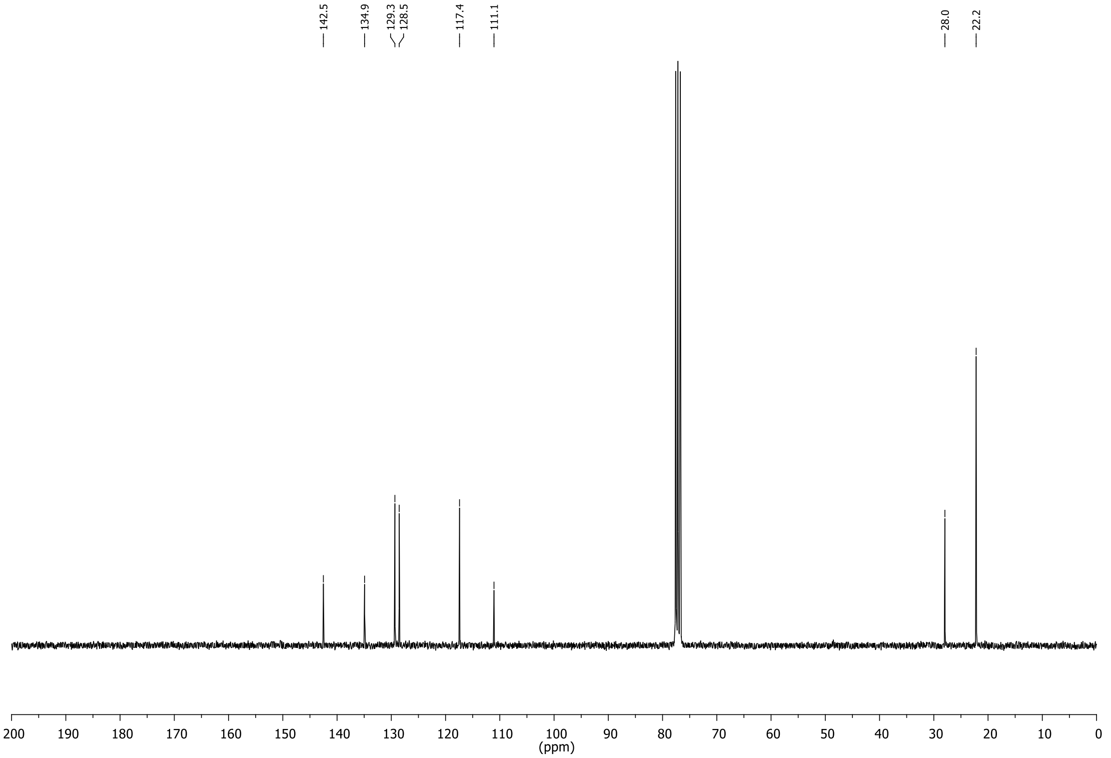

1-Bromo-4-iodo-2-isopropylbenzene (**7**)

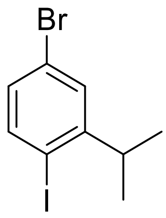

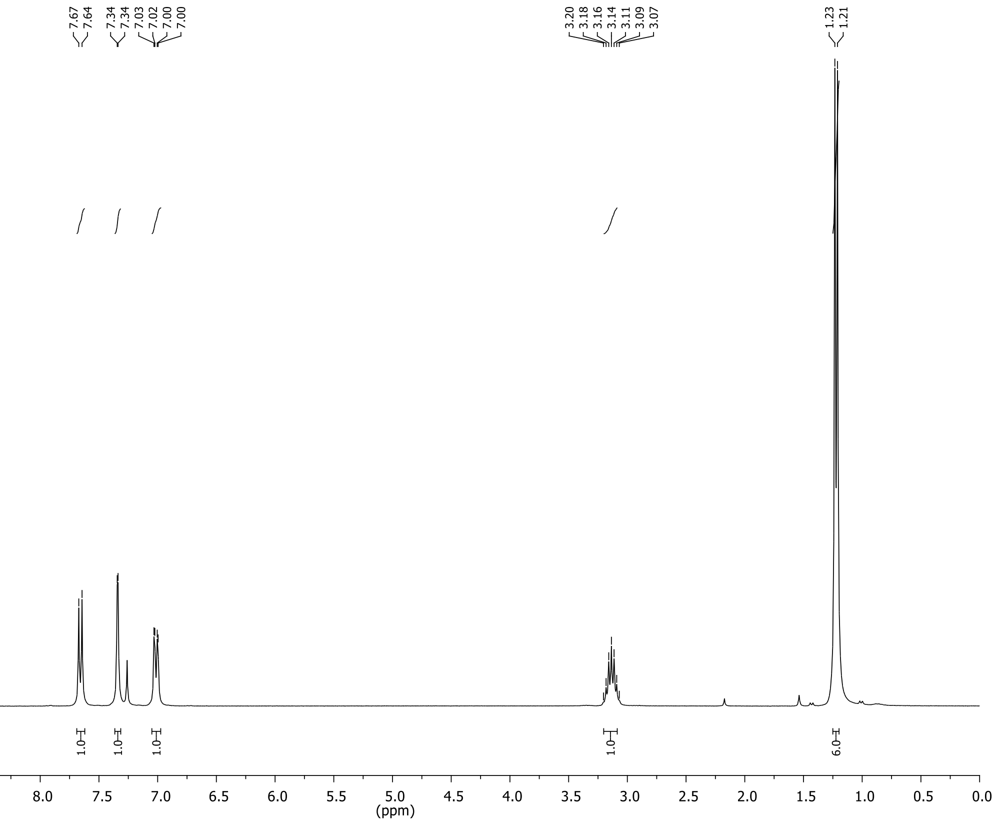

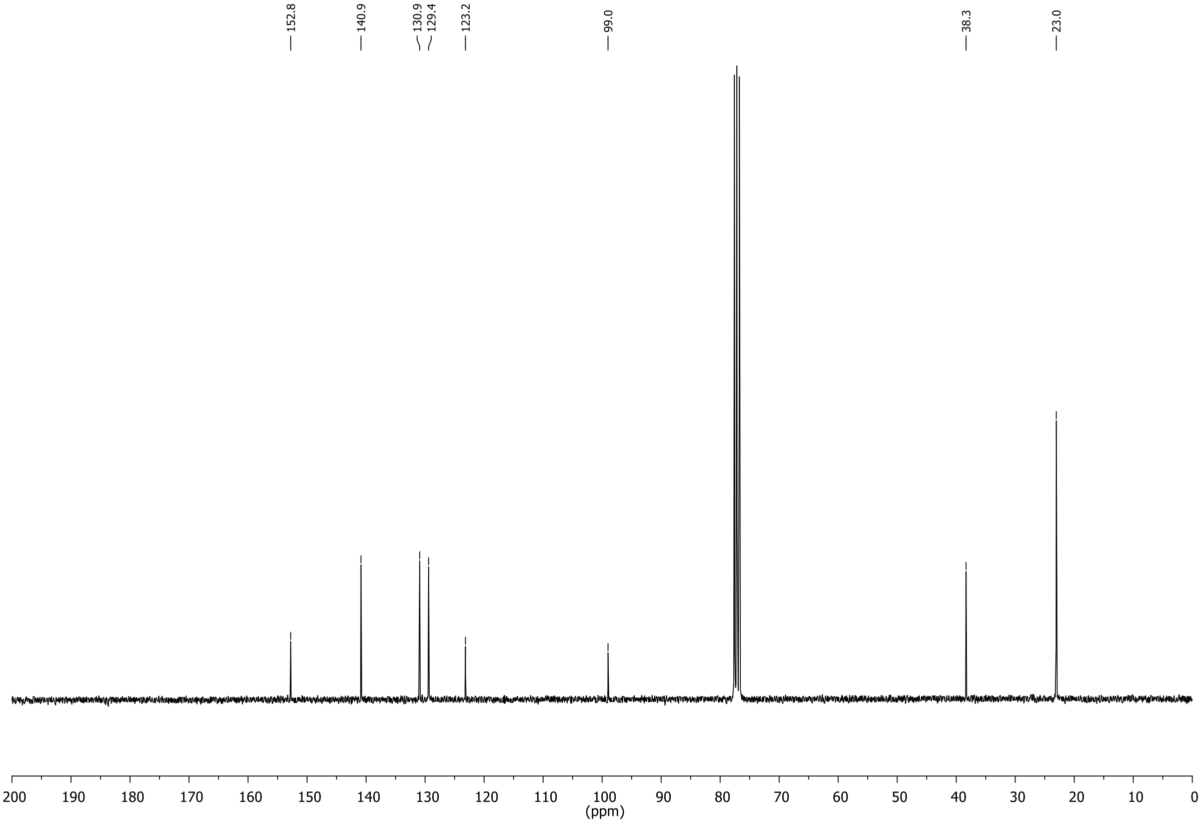

4-Bromo-2-isobutylaniline (**5**)

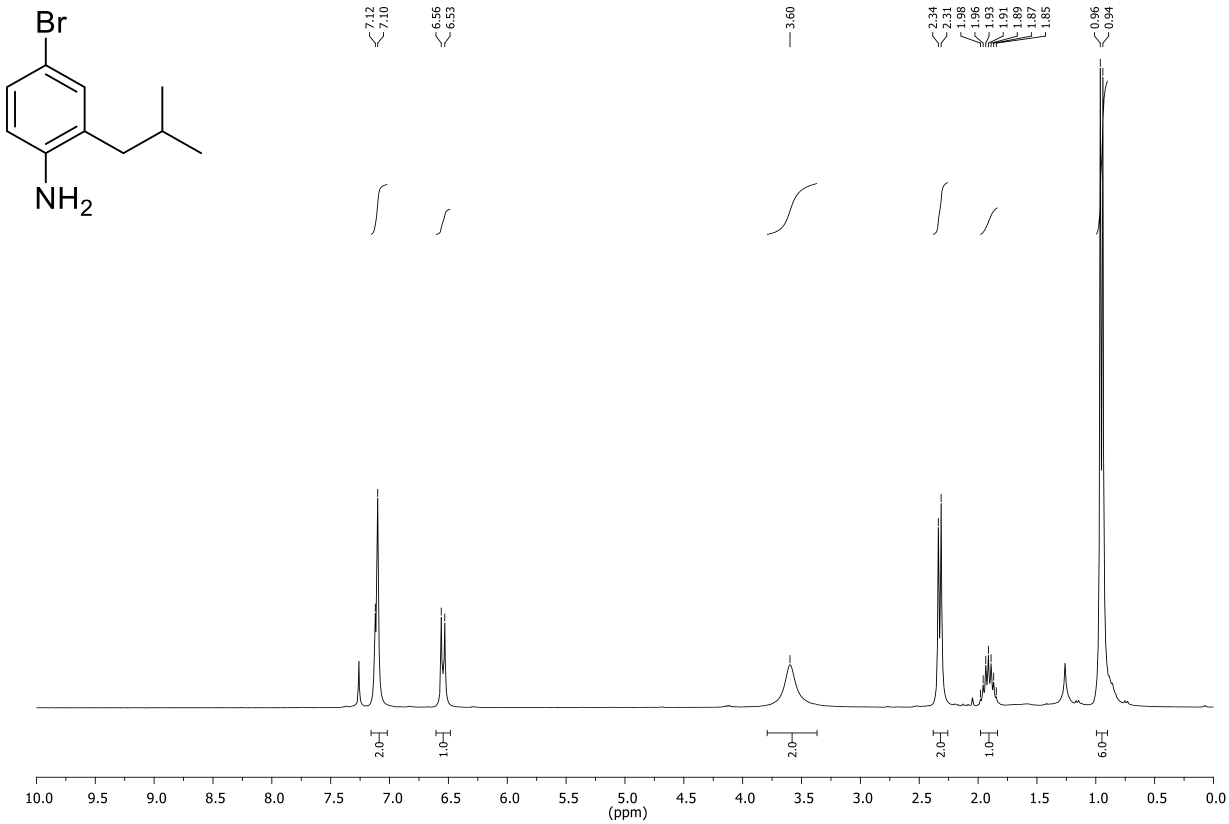

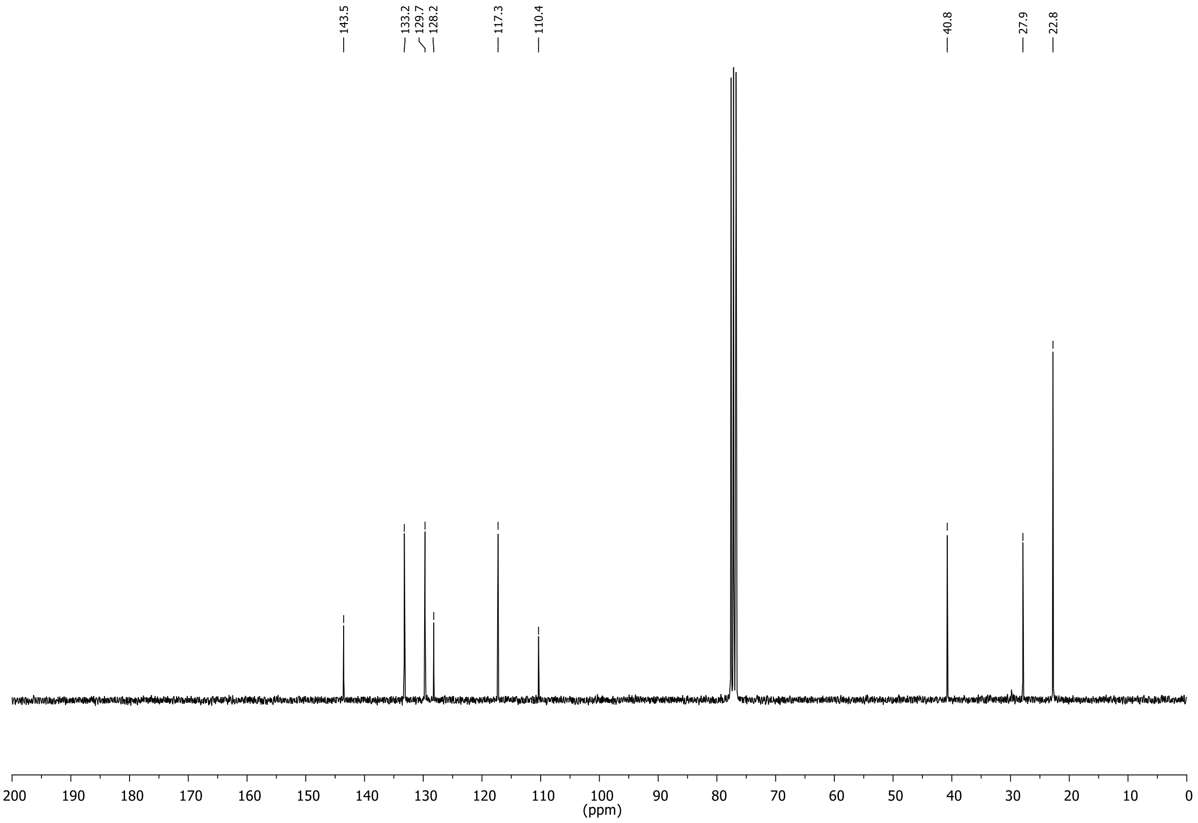

4-Bromo-1-iodo-2-isobutylbenzene (**8**)

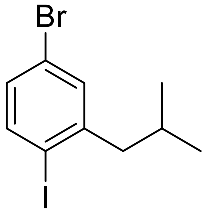

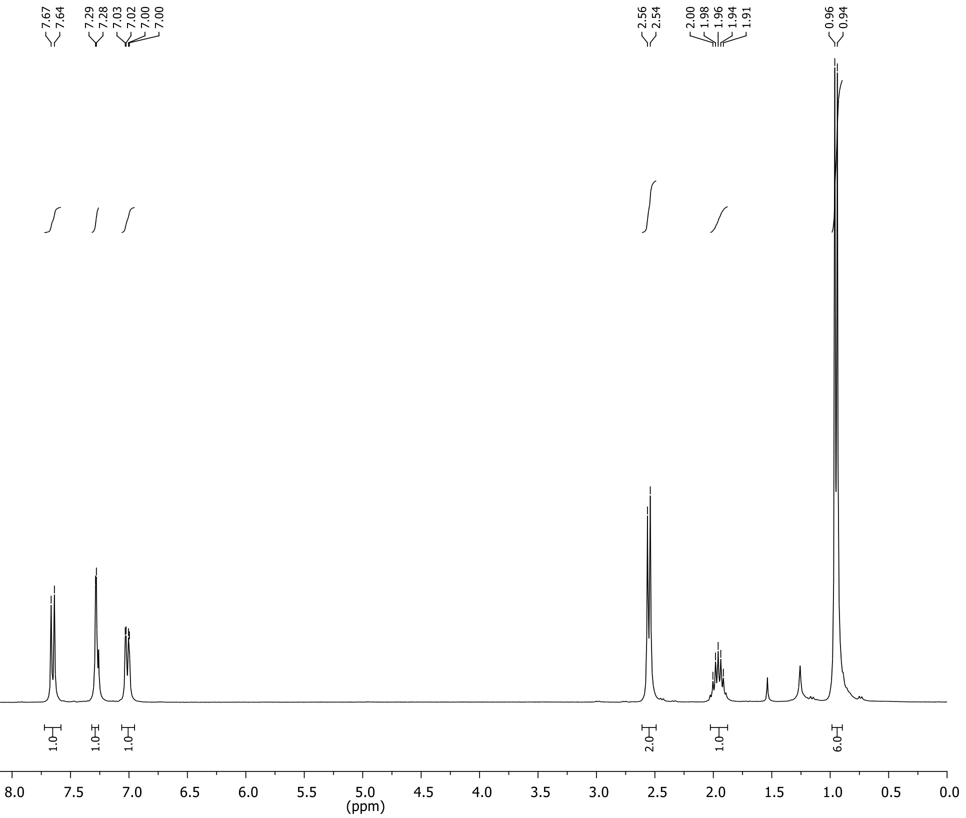

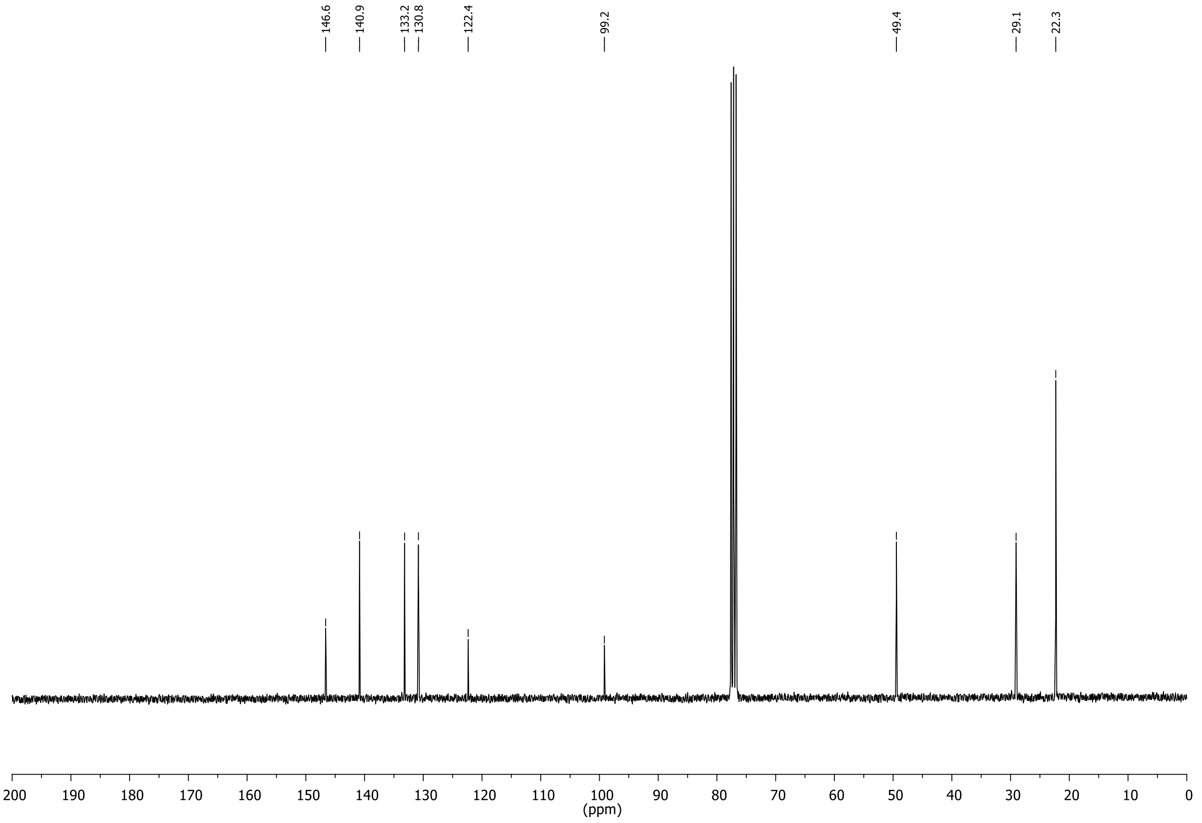

4-Bromo-2-(*sec*-butyl)aniline (**6**)

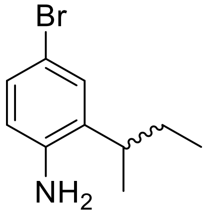

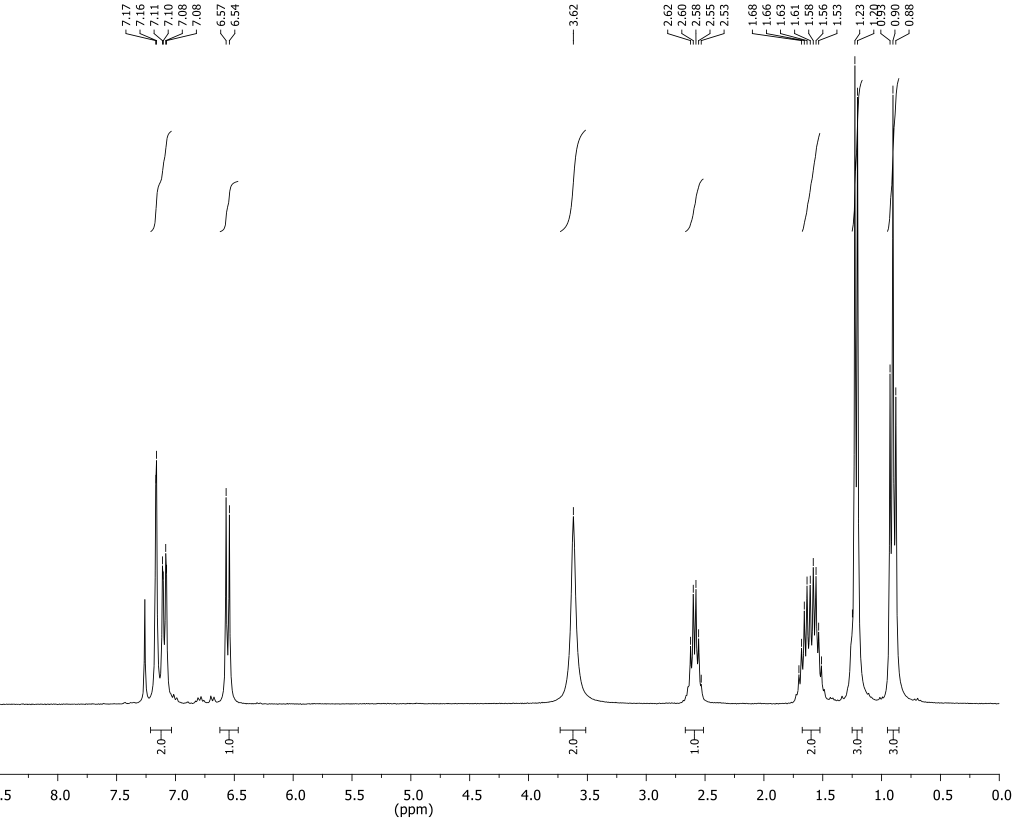

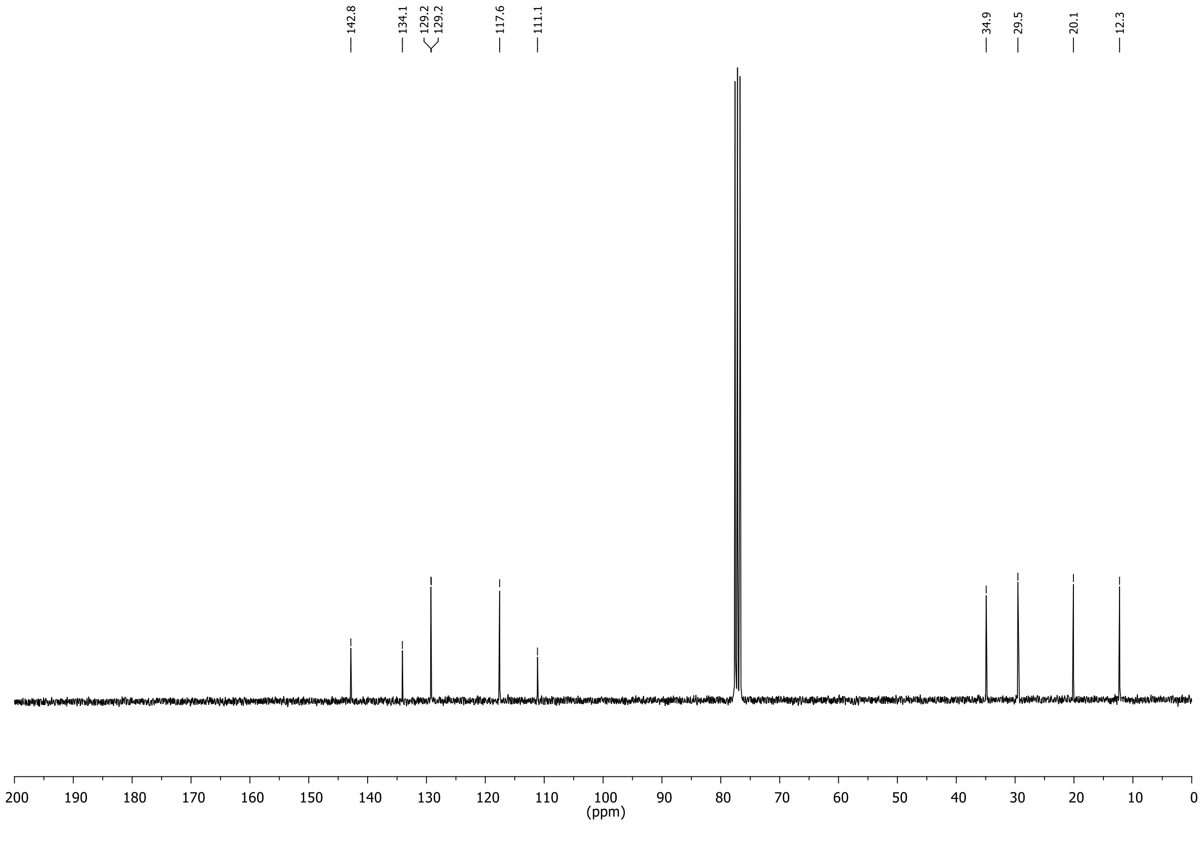

4-Bromo-2-(*sec*-butyl)-1-iodobenzene (**9**)

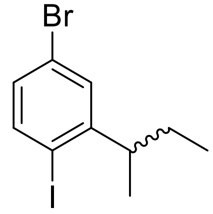

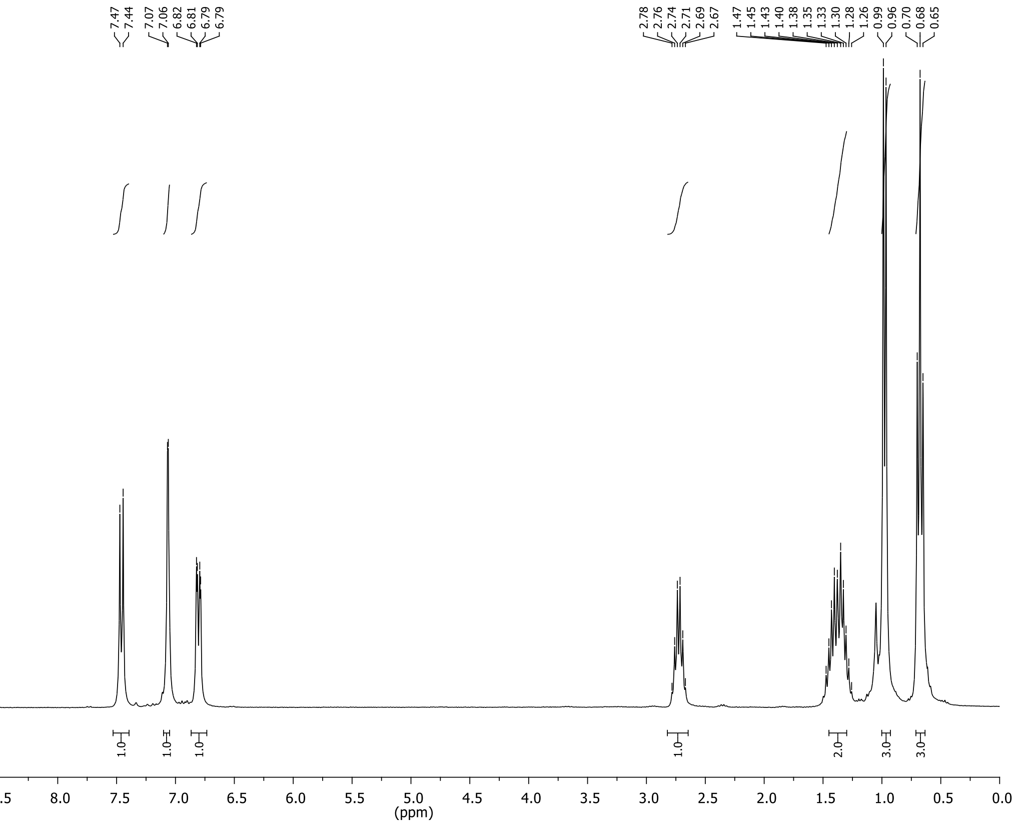

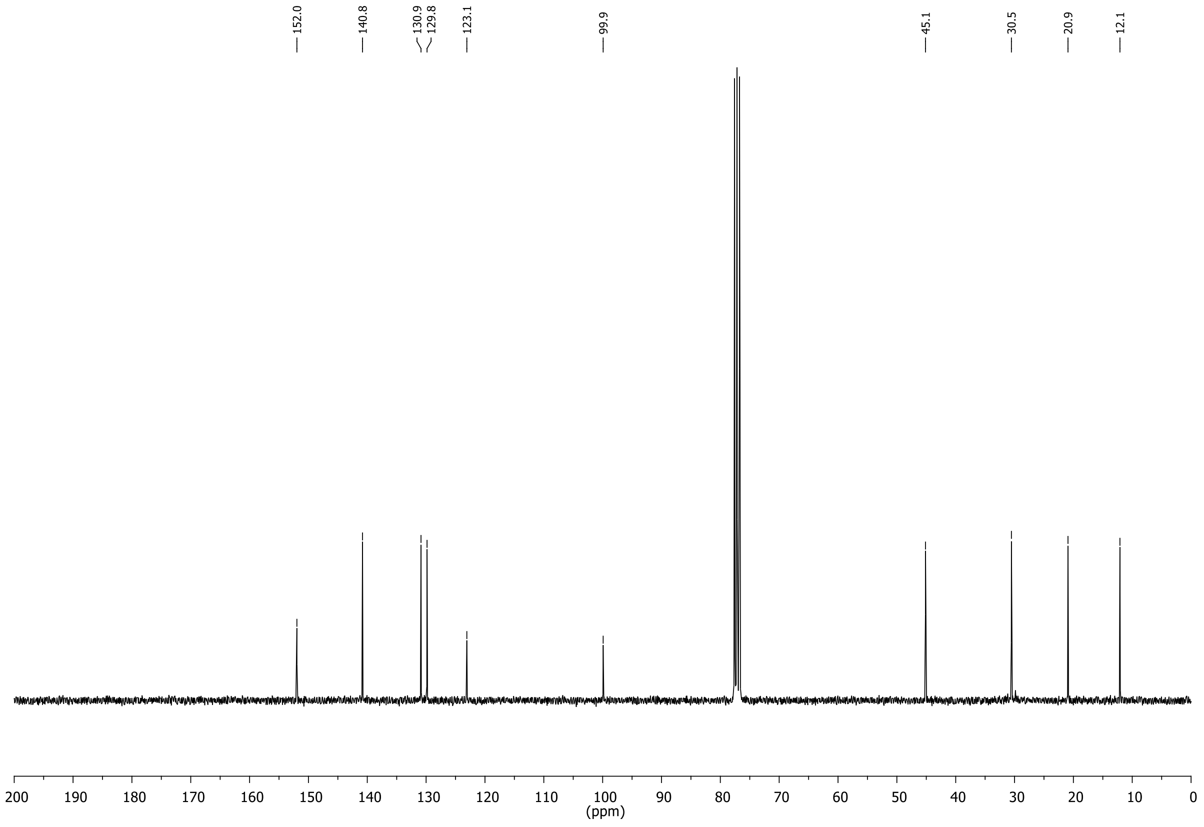

2-(2-Bromo-5-iodophenyl)acetonitrile (**11**)

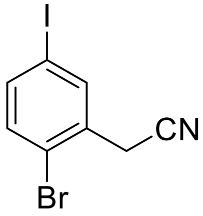

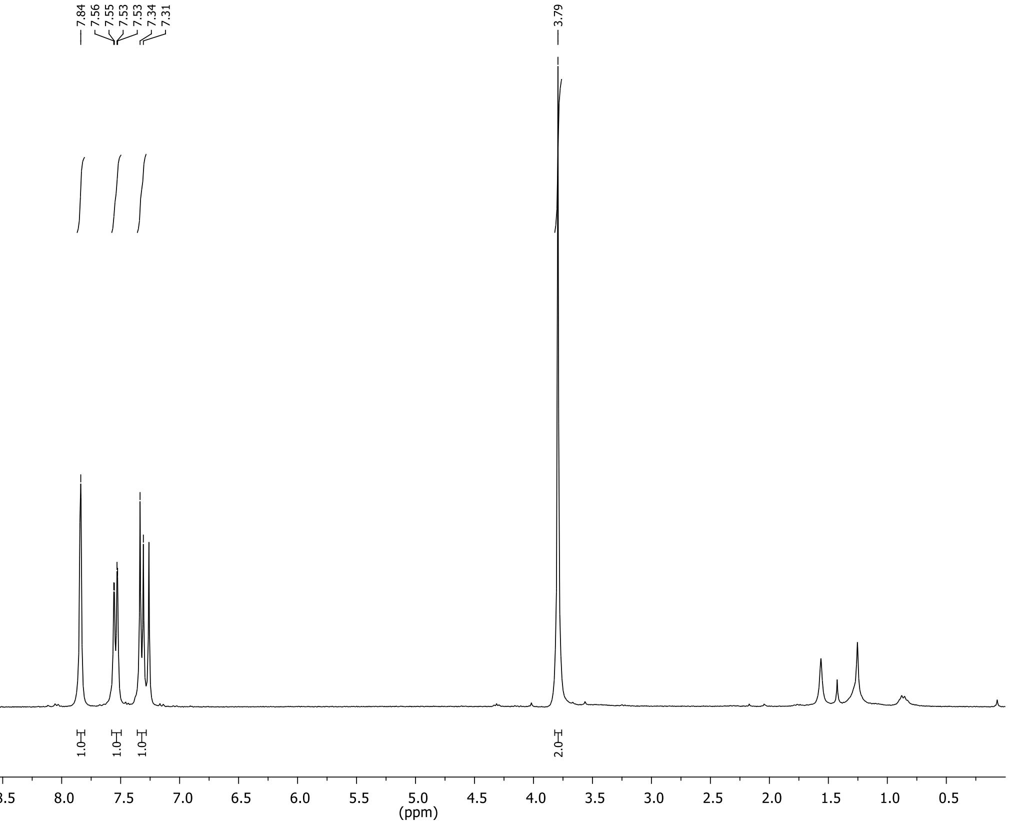

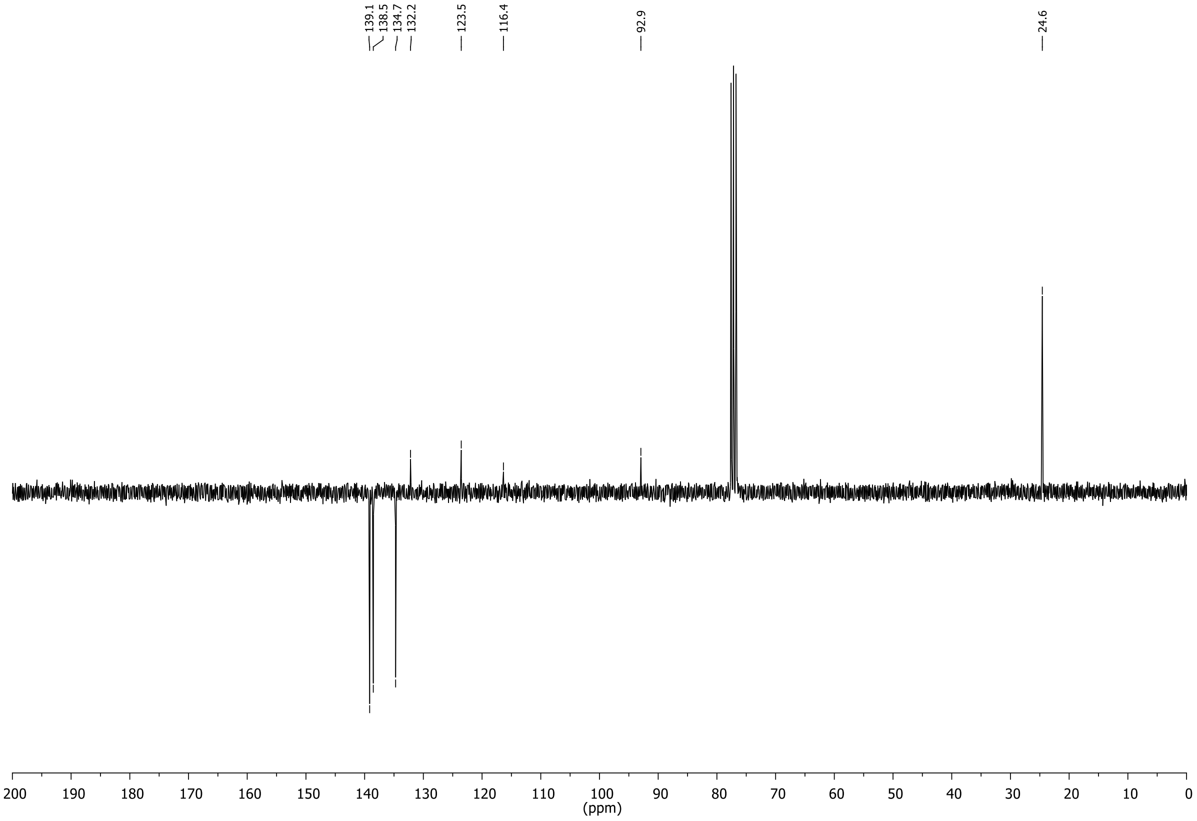

2-(2-Bromo-5-iodophenyl)acetamide (**12**)

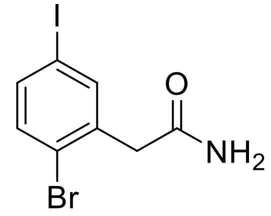

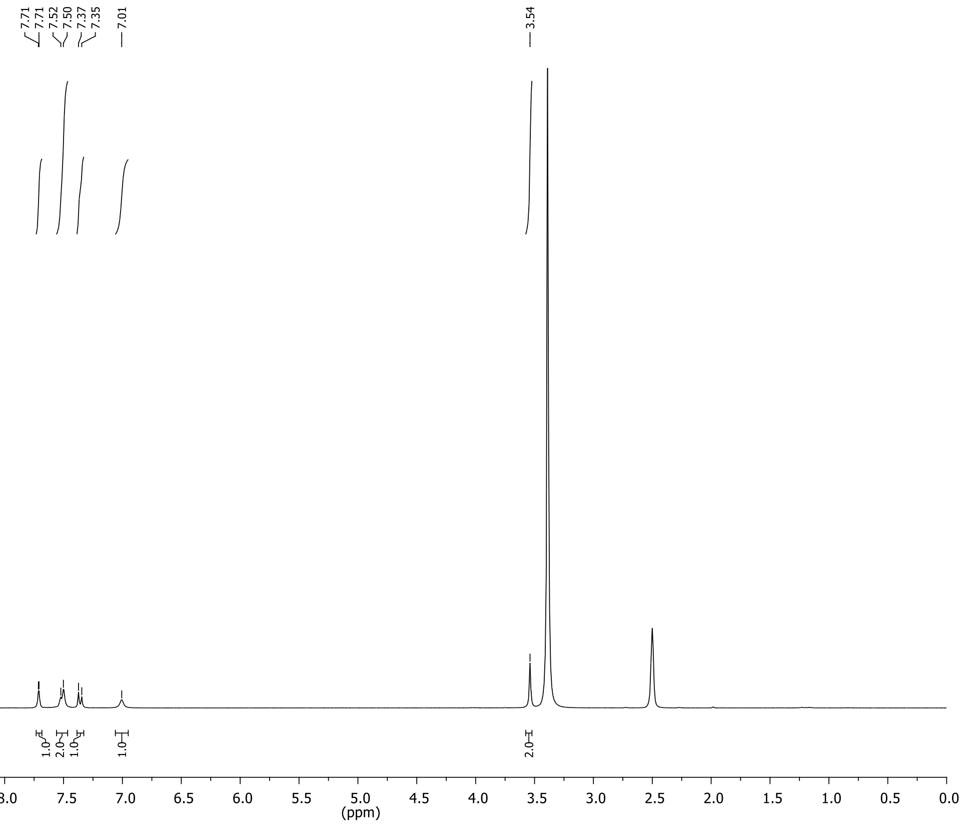

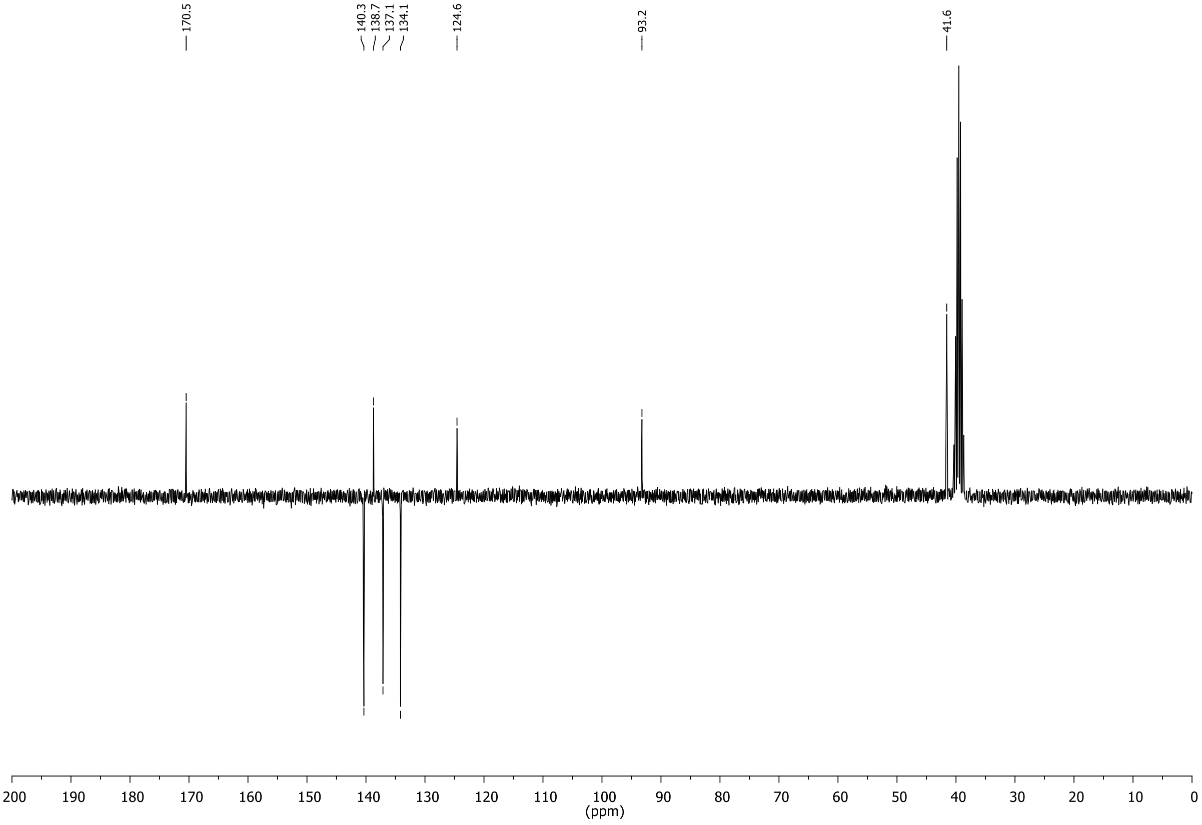

Methyl 2-(2-bromo-5-iodophenyl)acetate (**13**)

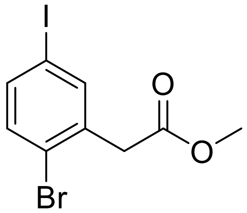

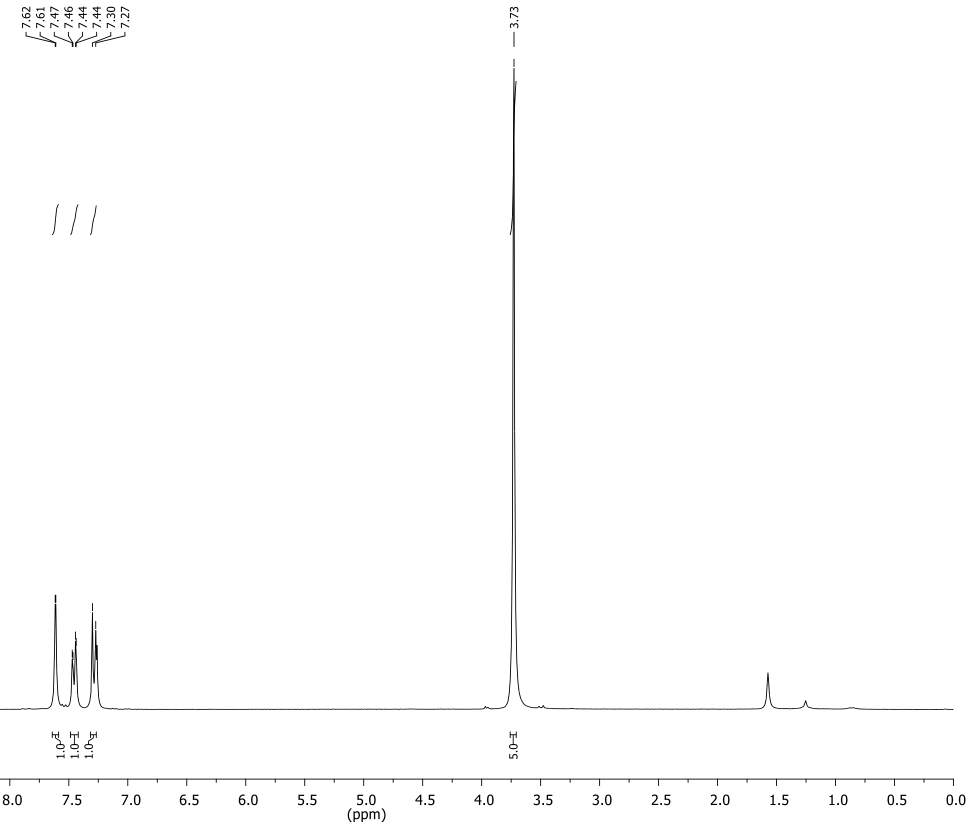

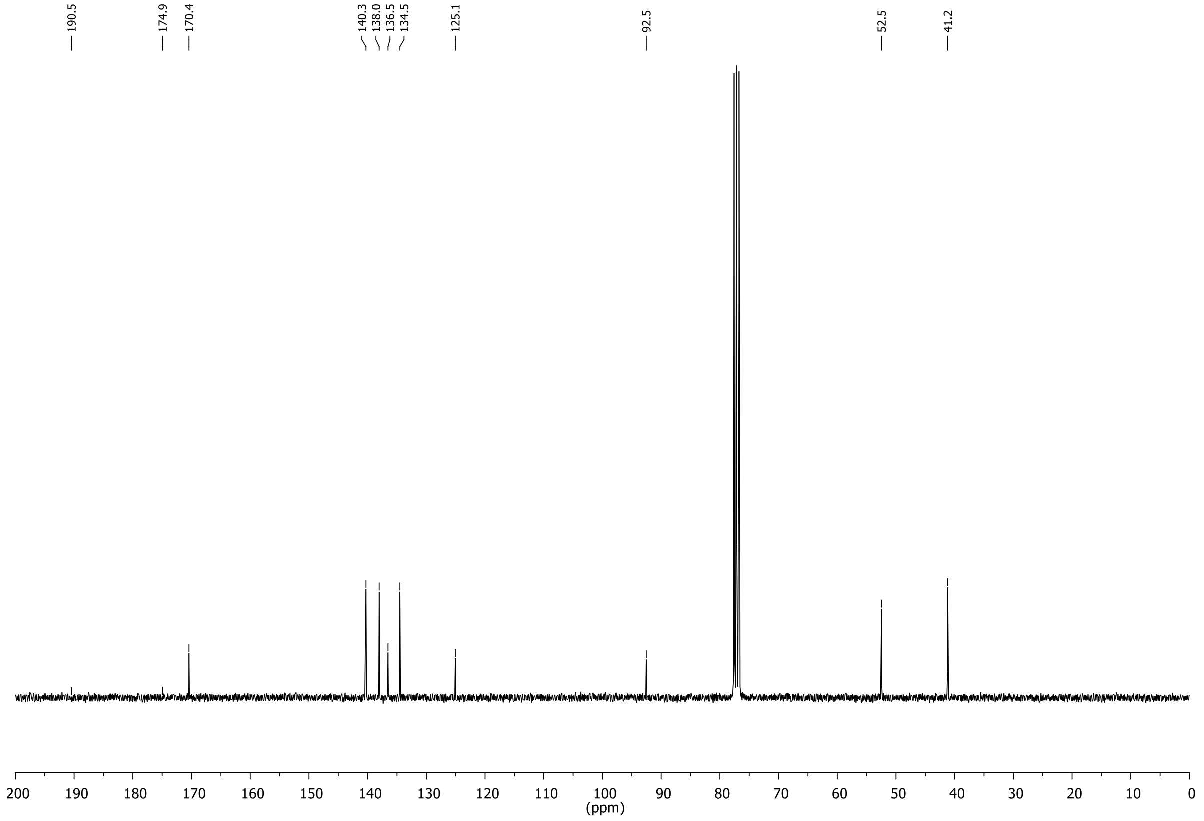

1-Bromo-2-(bromomethyl)-4-iodobenzene (**14**)

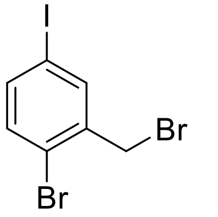

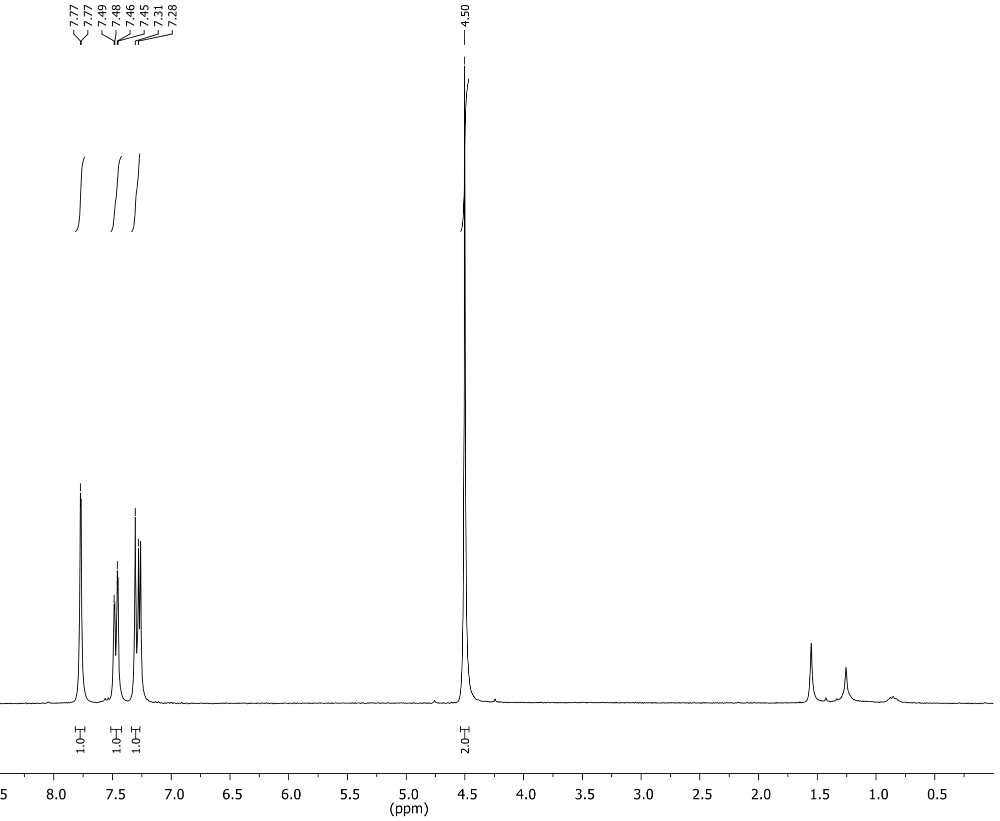

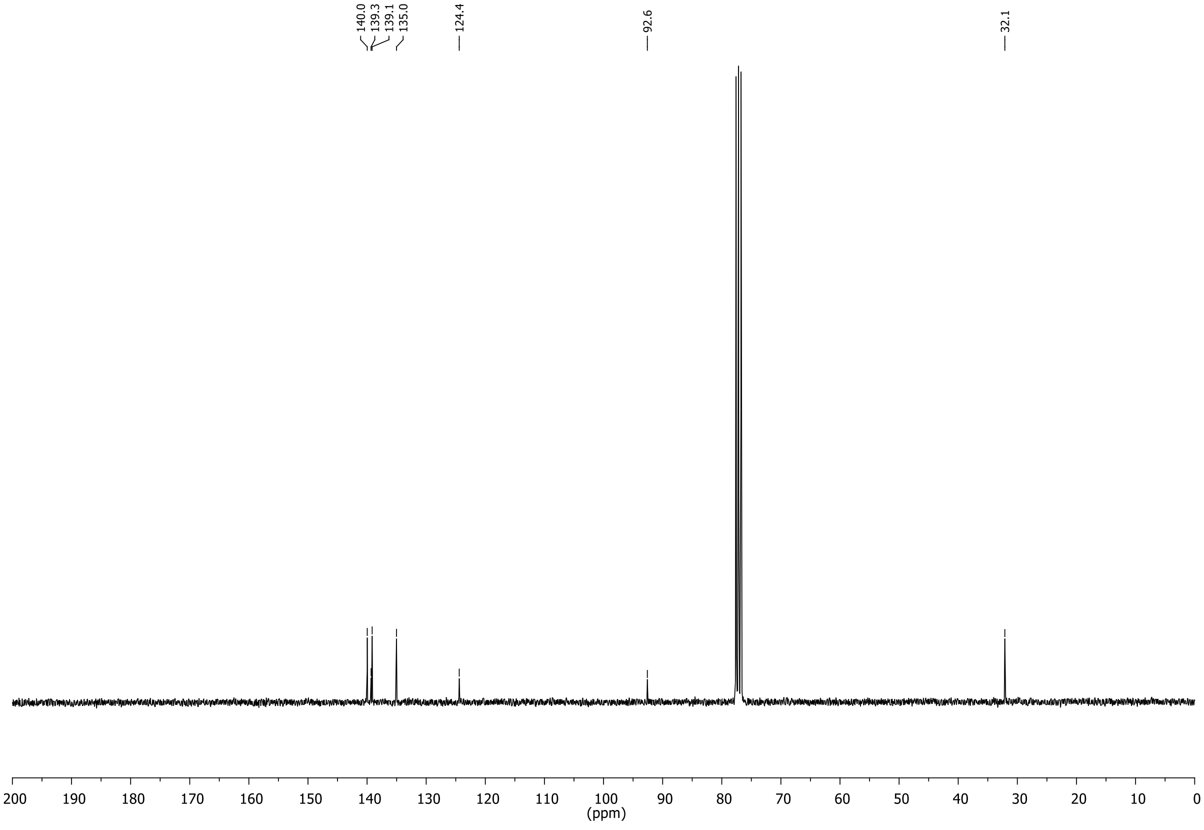

(2-Bromo-5-iodobenzyl) ethanethioate (**15**)

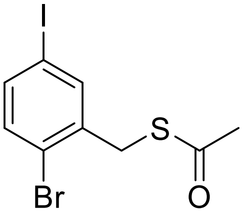

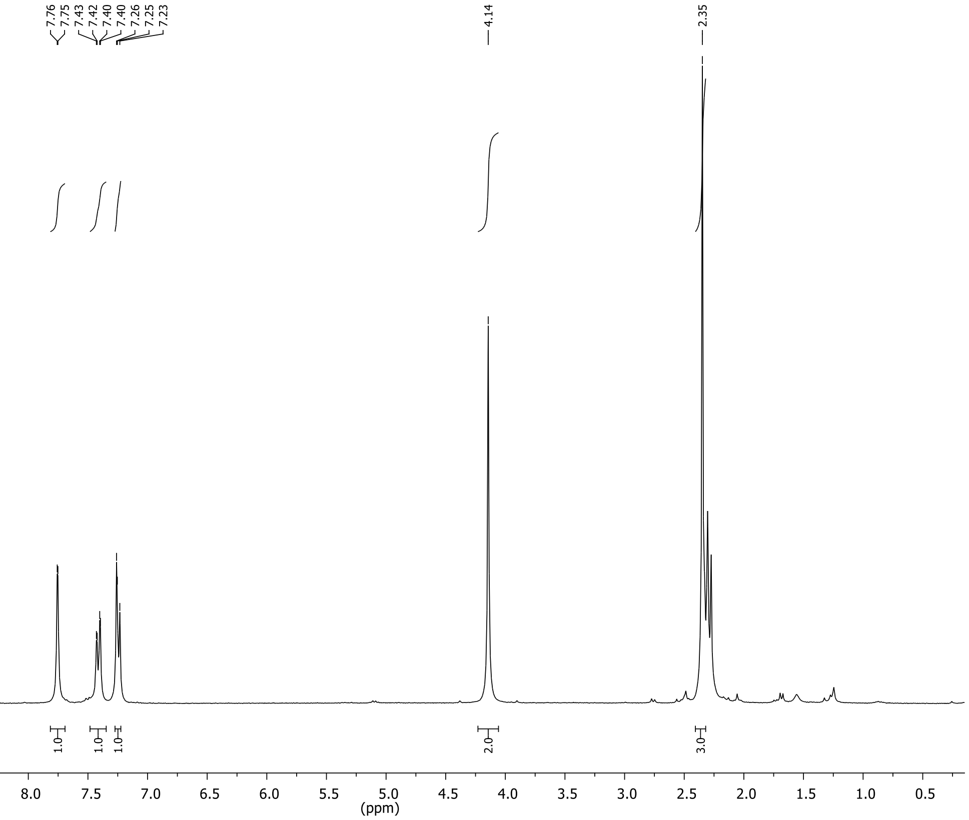

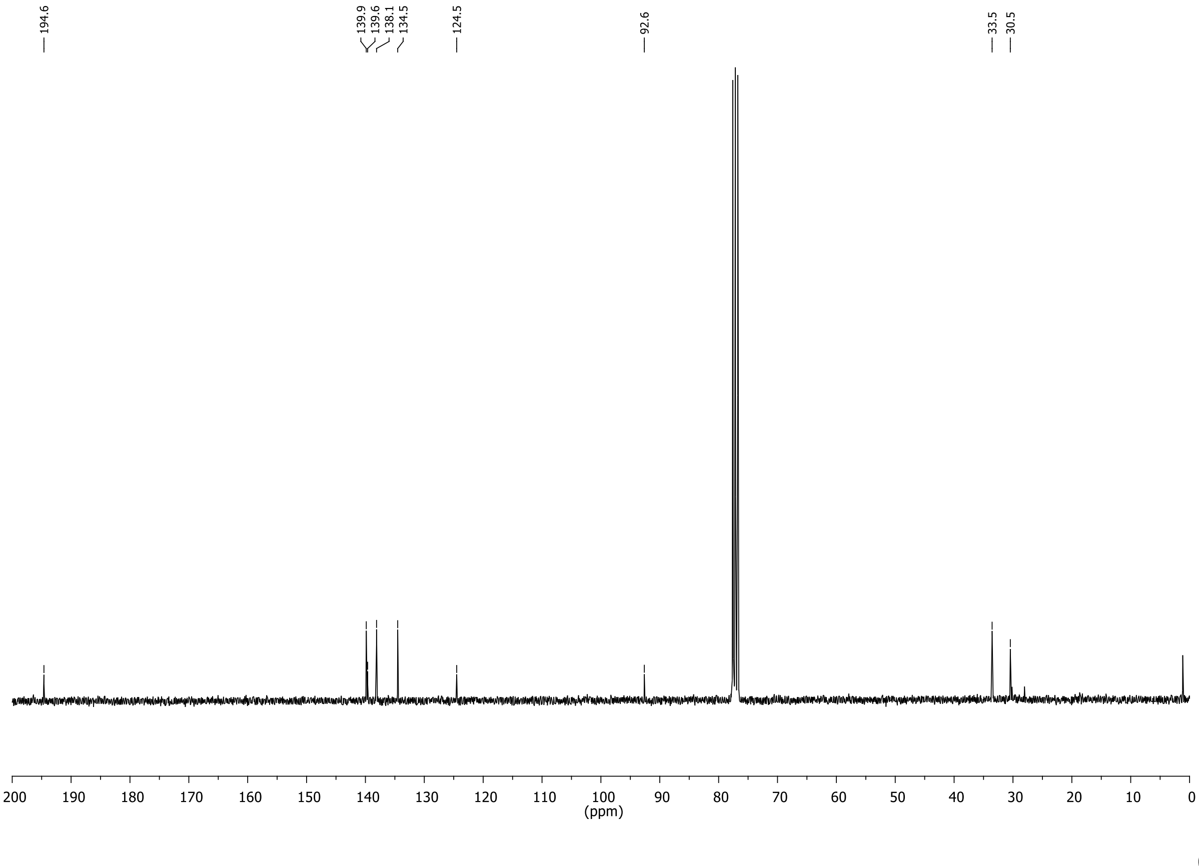

2-Bromo-5-iodobenzaldehyde (**16**)

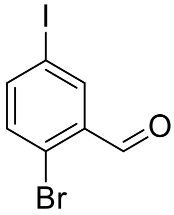

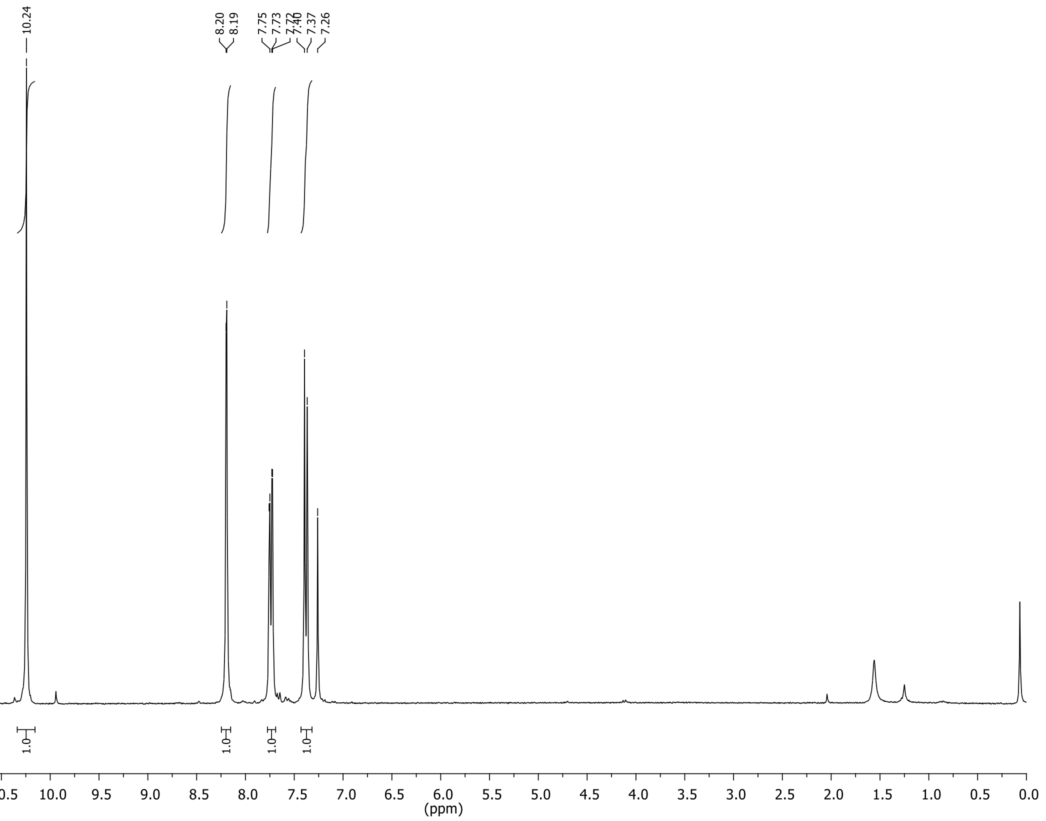

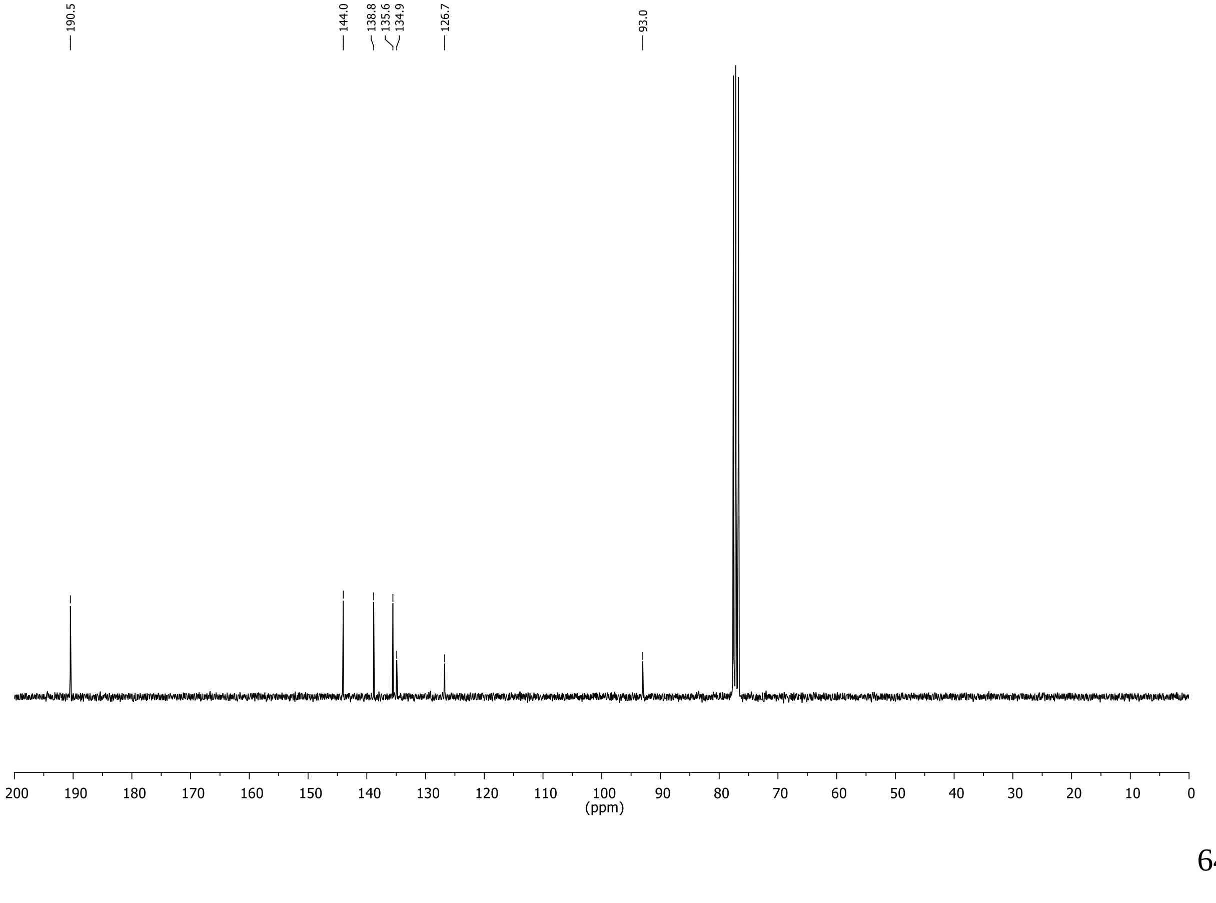

1-(2-Bromo-5-iodophenyl)ethan-1-ol **(17)**

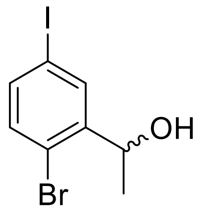

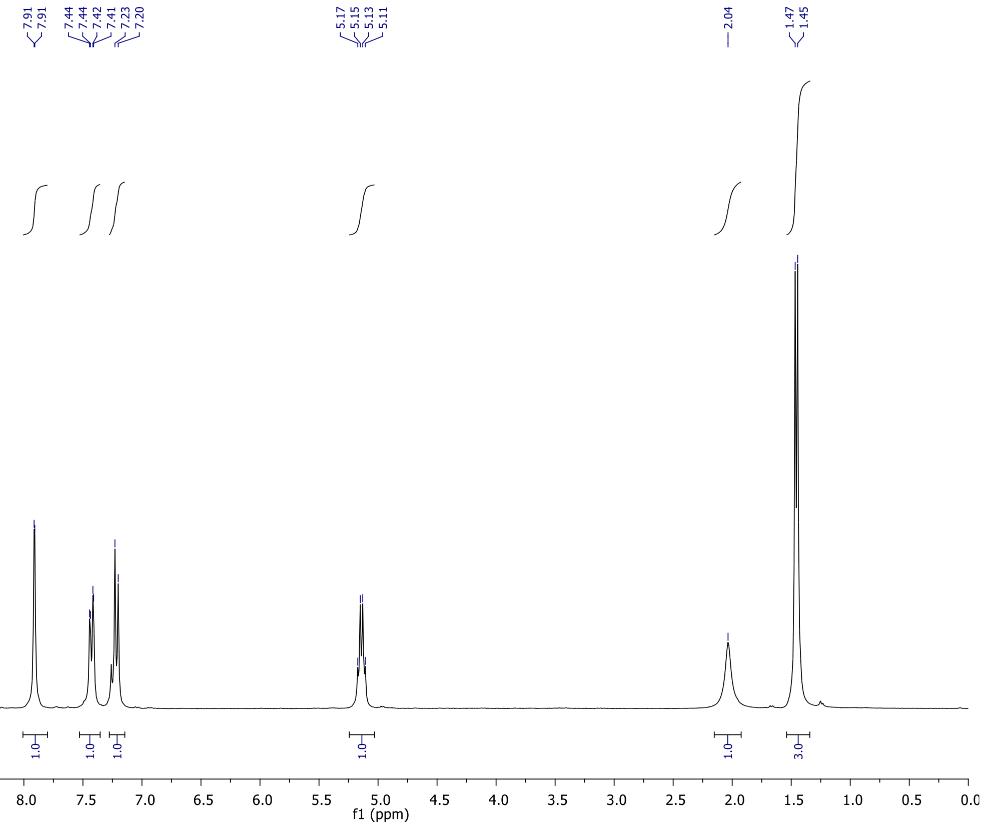

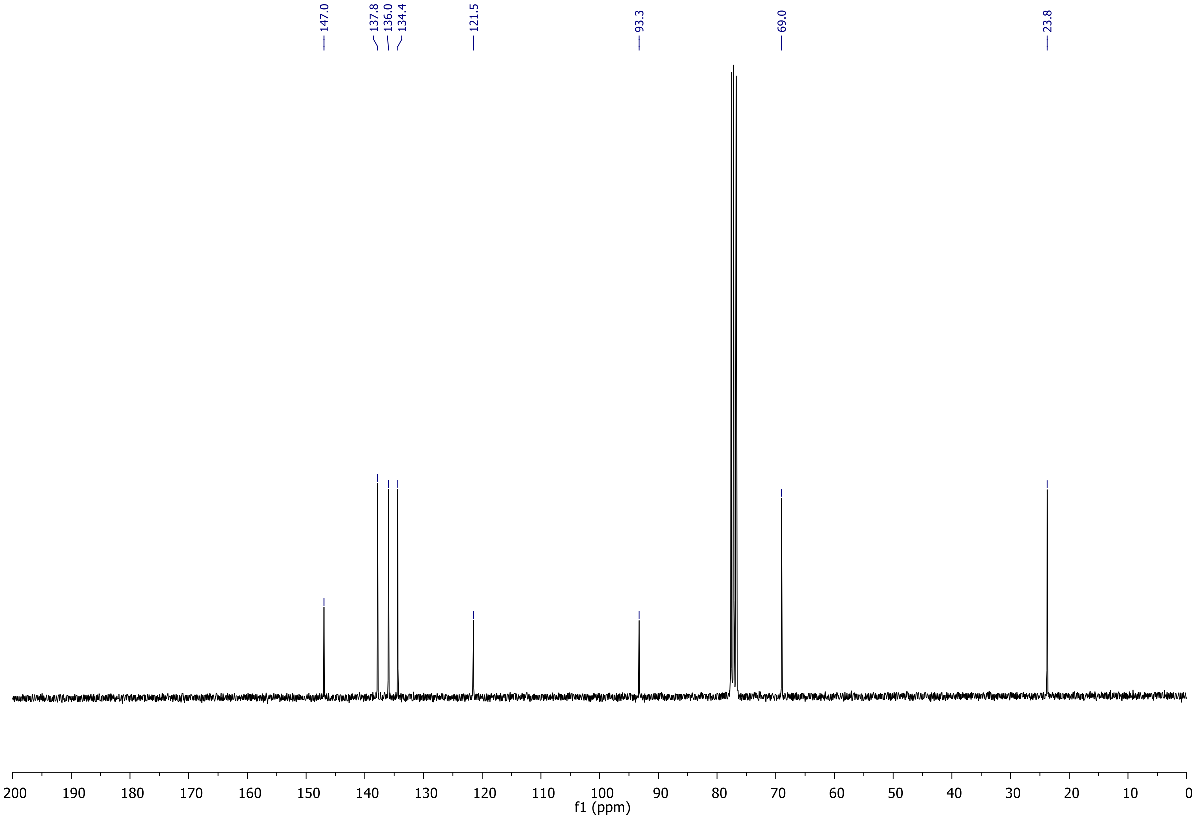

((Methylthio)methyl)triphenylphosphonium chloride (**18**)

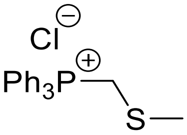

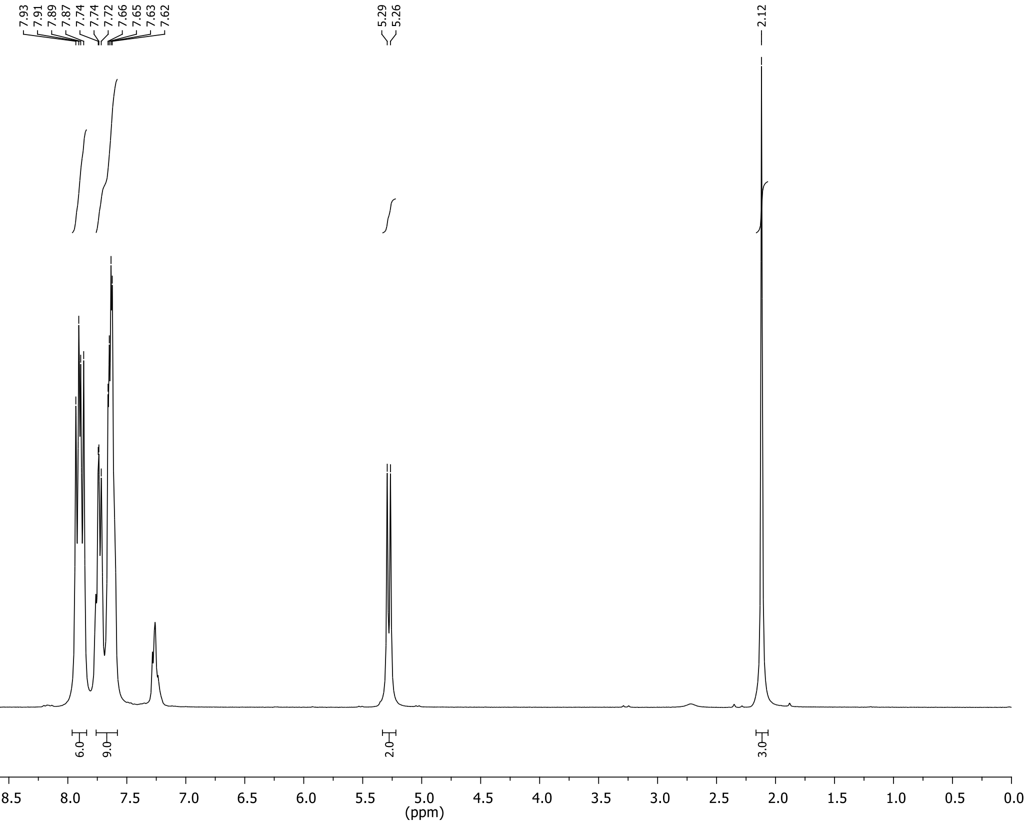

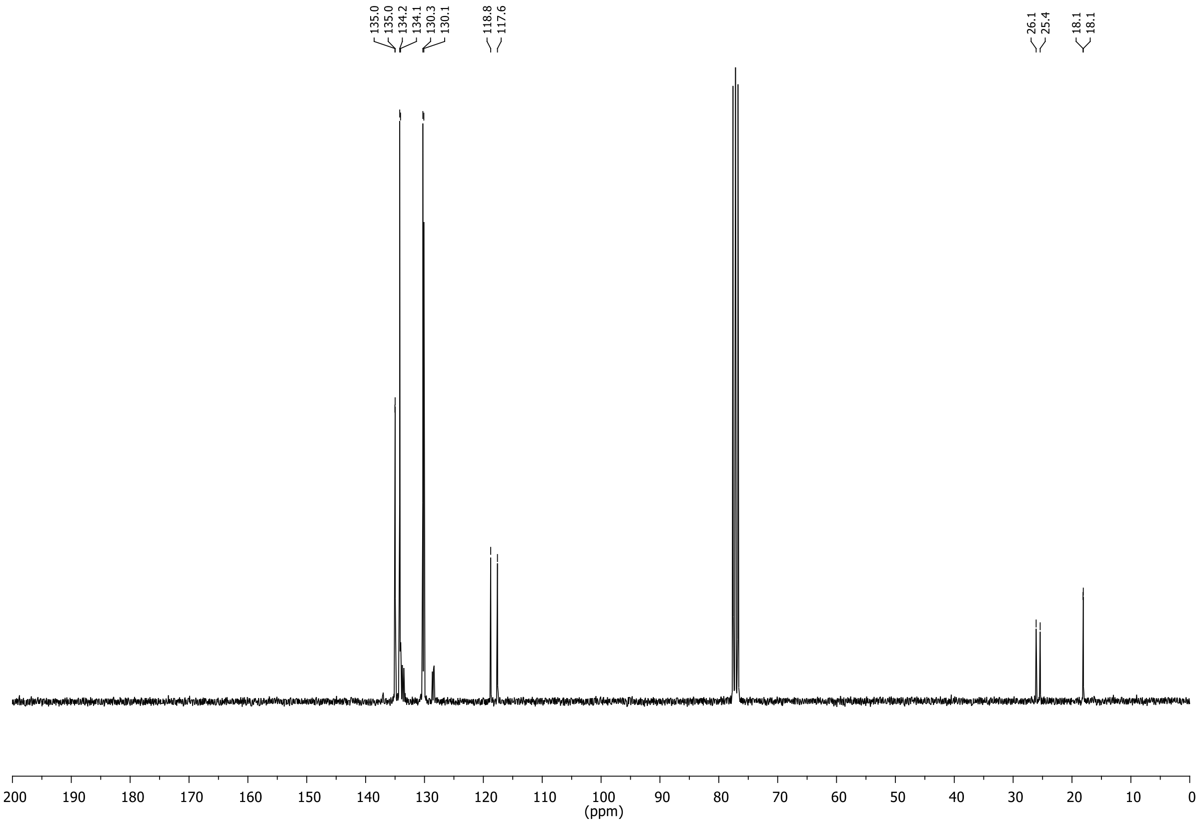

(2-bromo-5-iodostyryl)(methyl)sulfane (**19**)

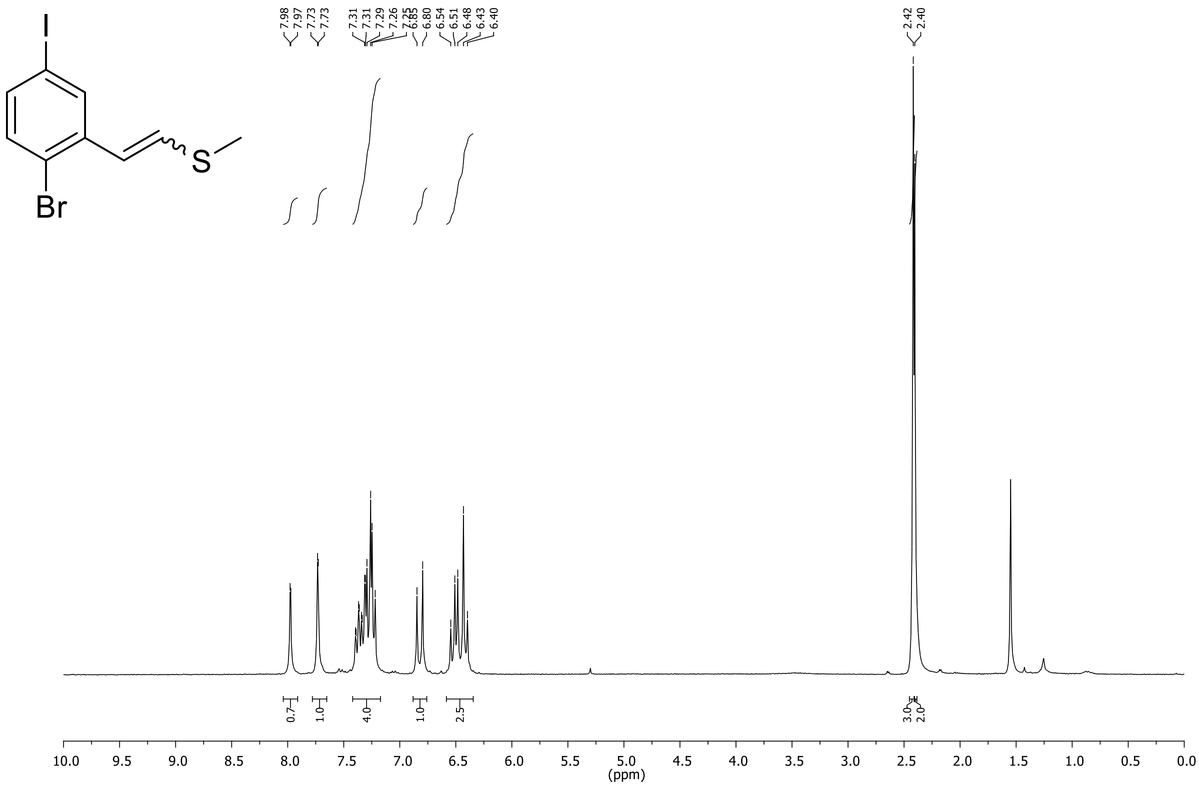

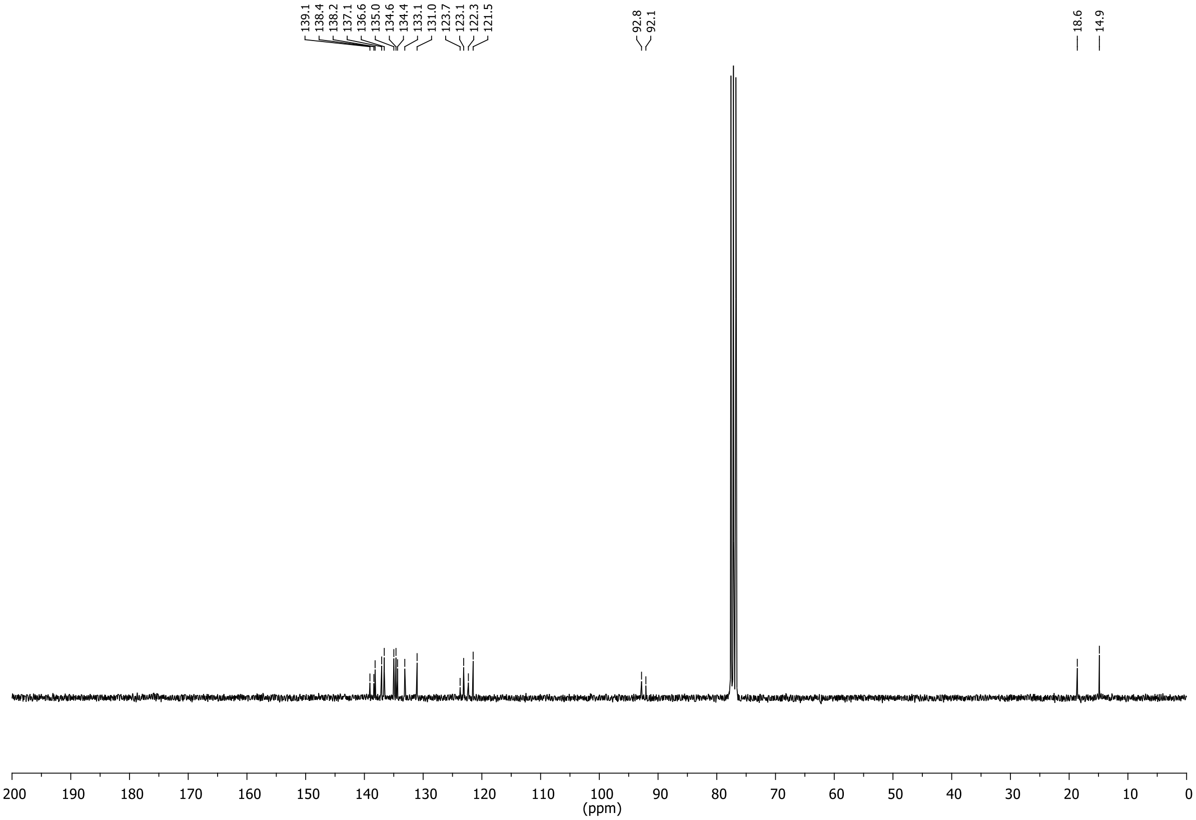

(2-Bromo-5-iodophenethyl)(methyl)sulfane (**20**)

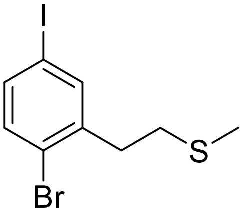

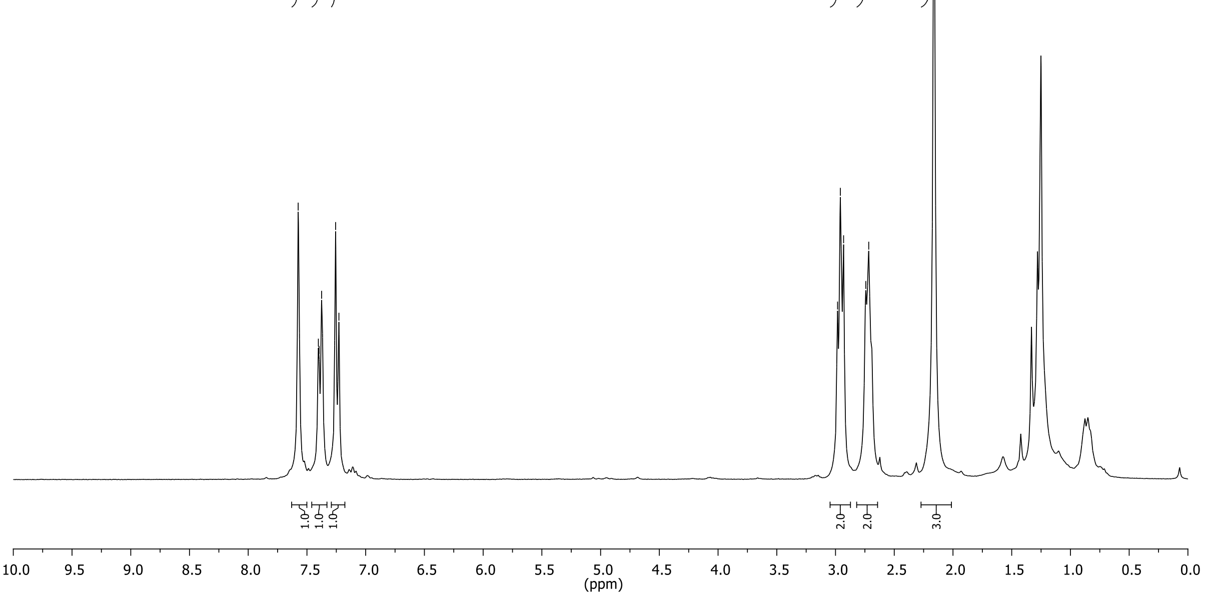

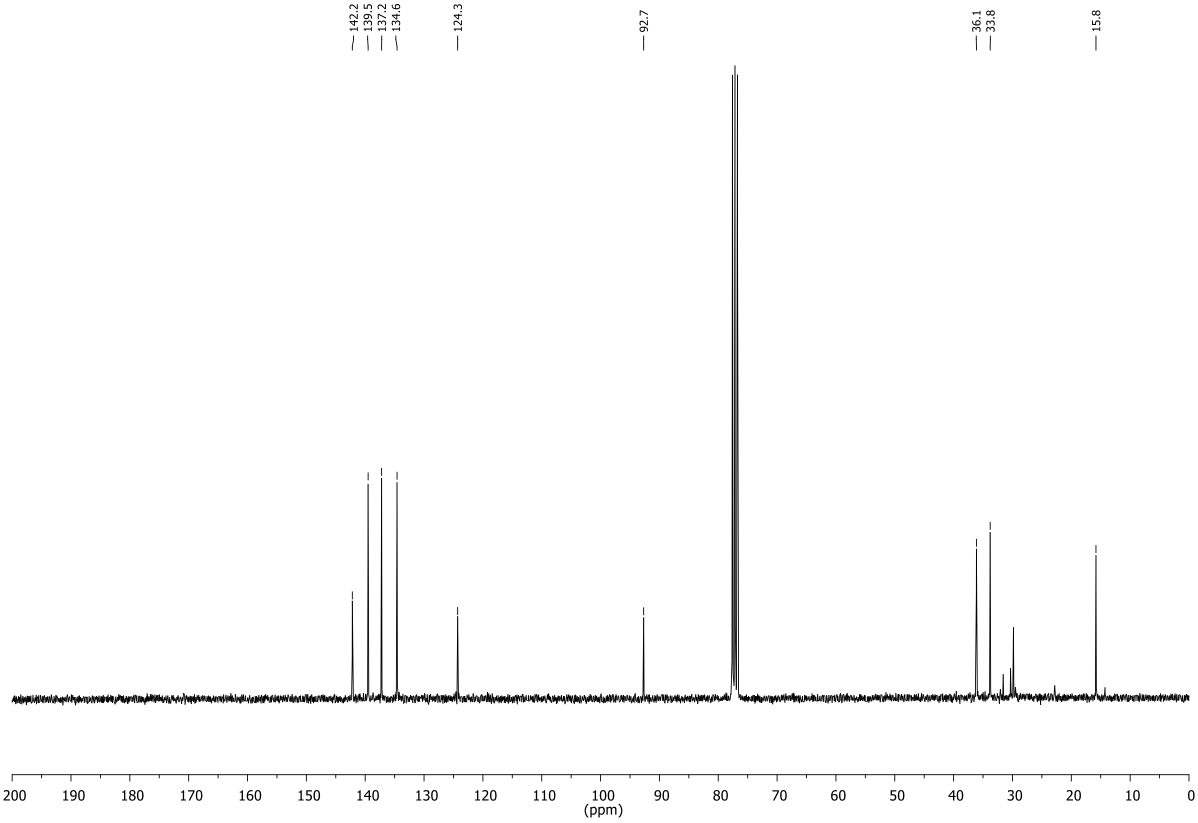

(Cyanomethyl)triphenylphosphonium chloride (**21**)

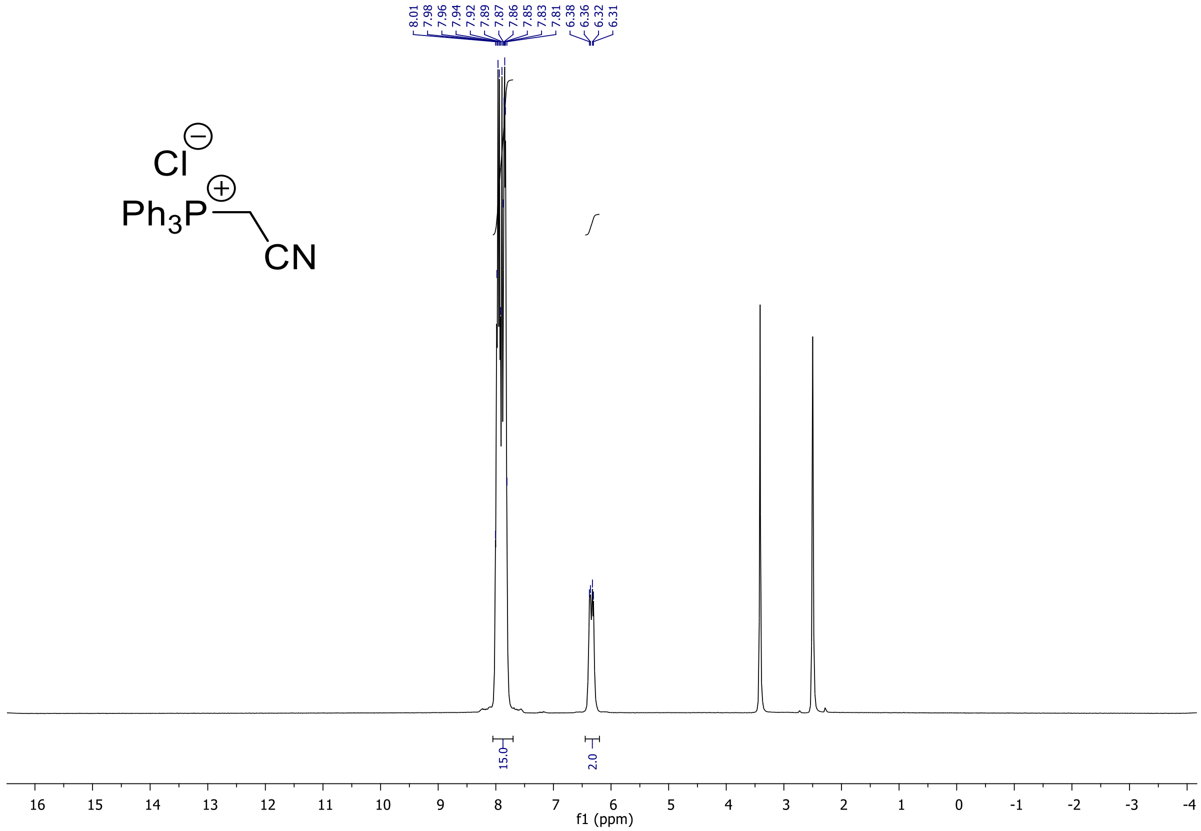

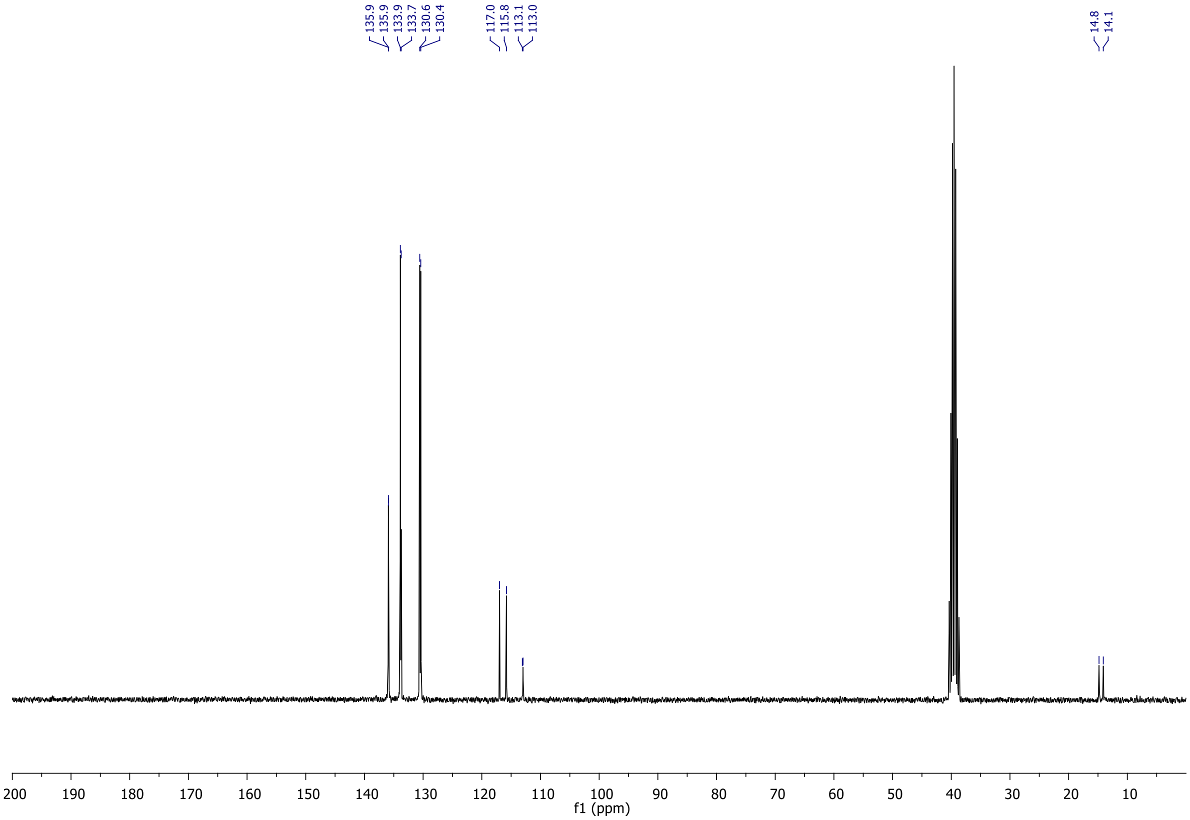

3-(2-bromo-5-iodophenyl)acrylonitrile (**22**)

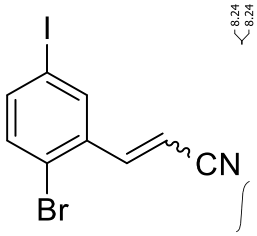

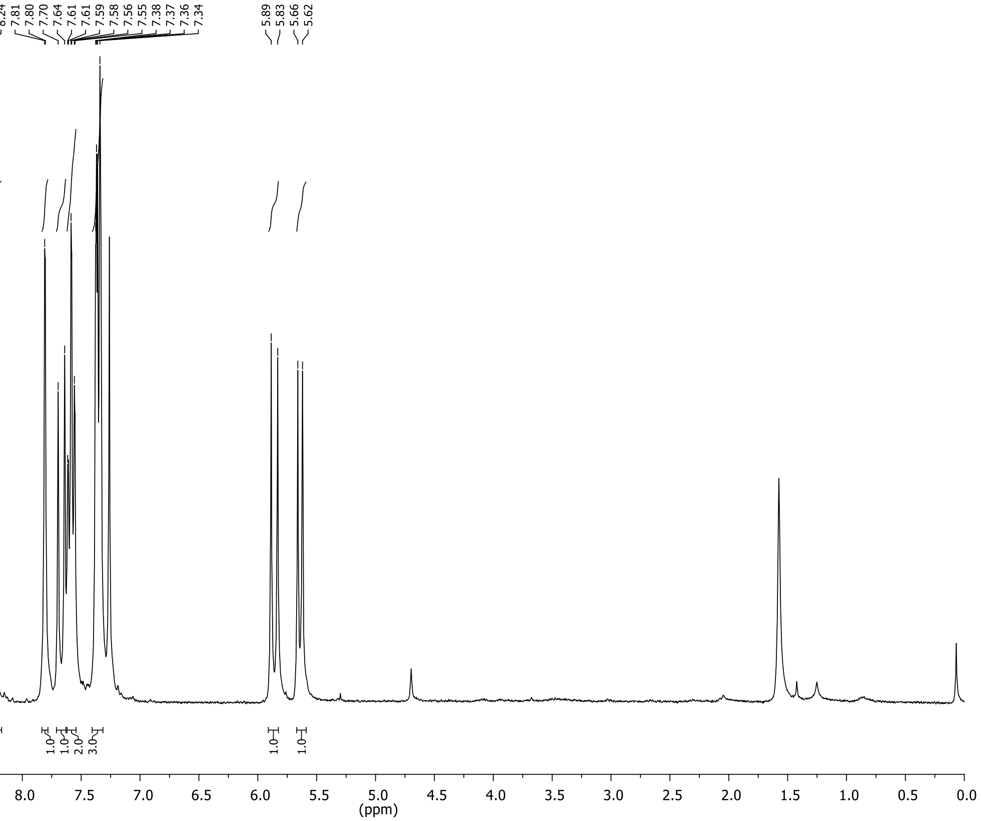

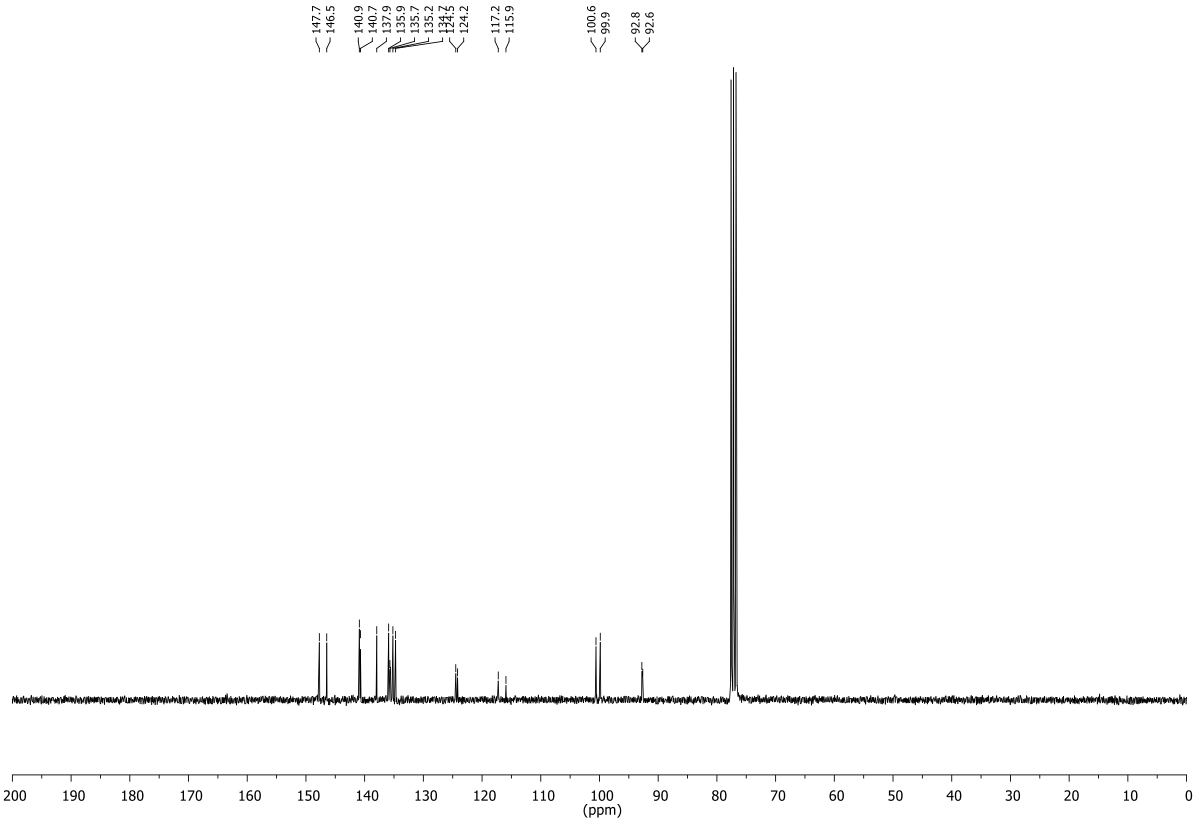

3-(2-Bromo-5-iodophenyl)propanenitrile (**23**)

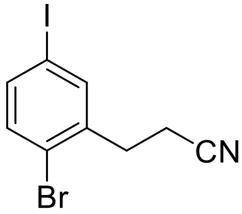

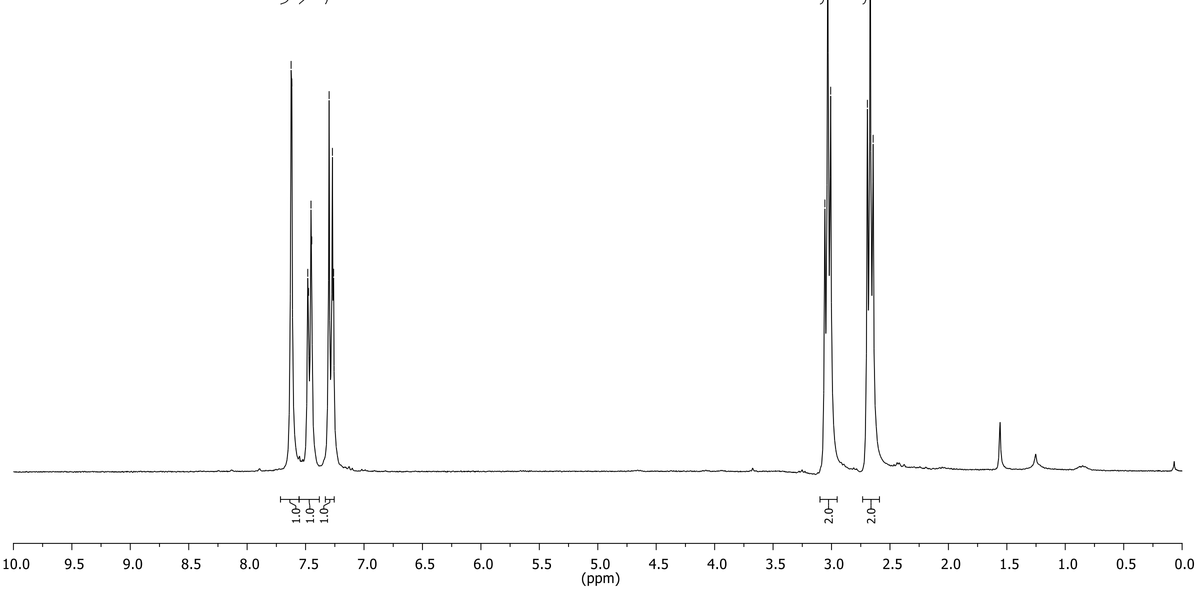

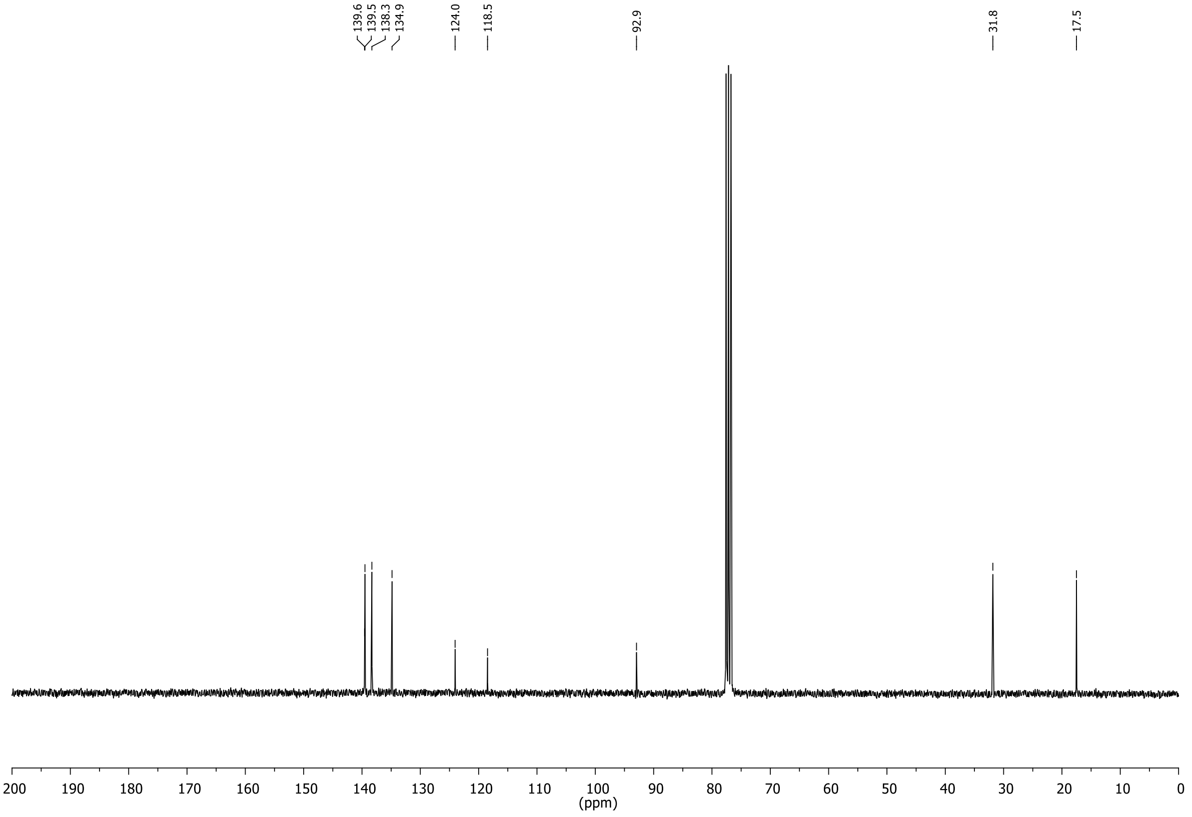

(2-Amino-2-oxoethyl)triphenylphosphonium chloride (**24**)

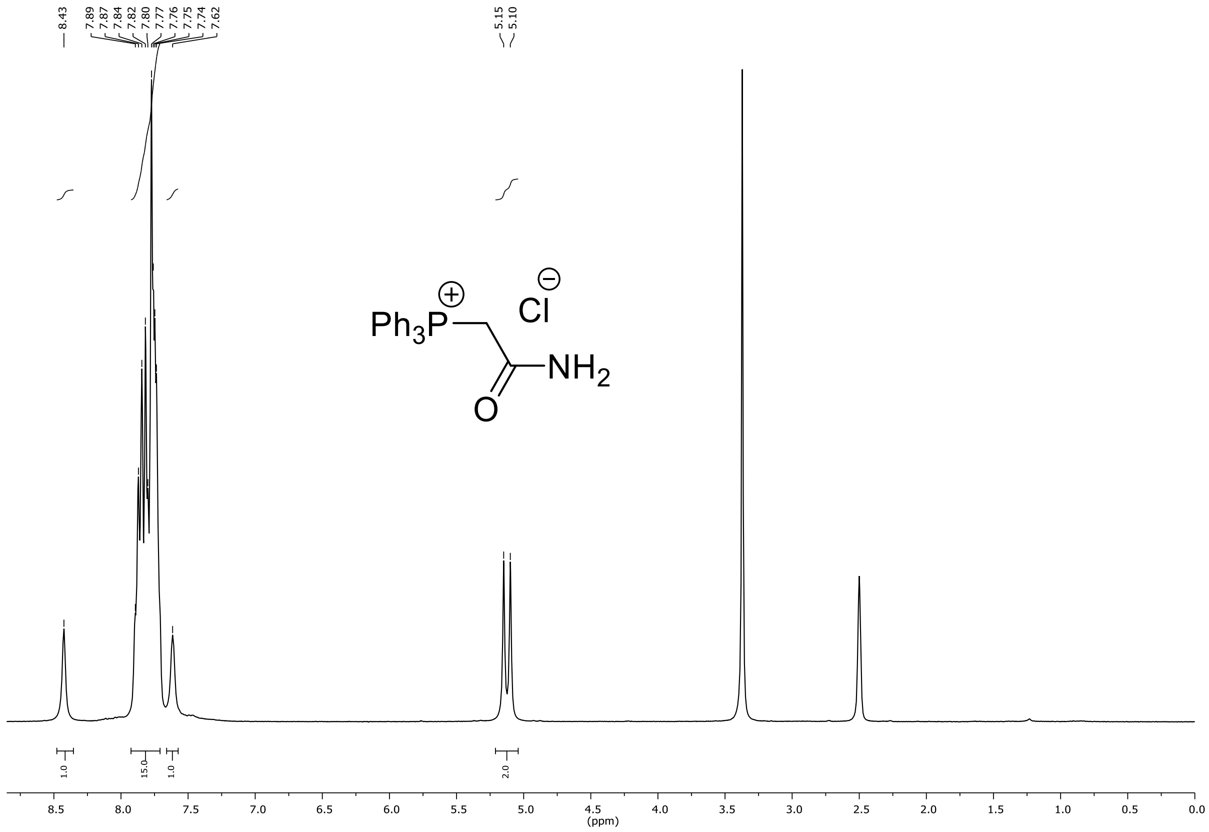

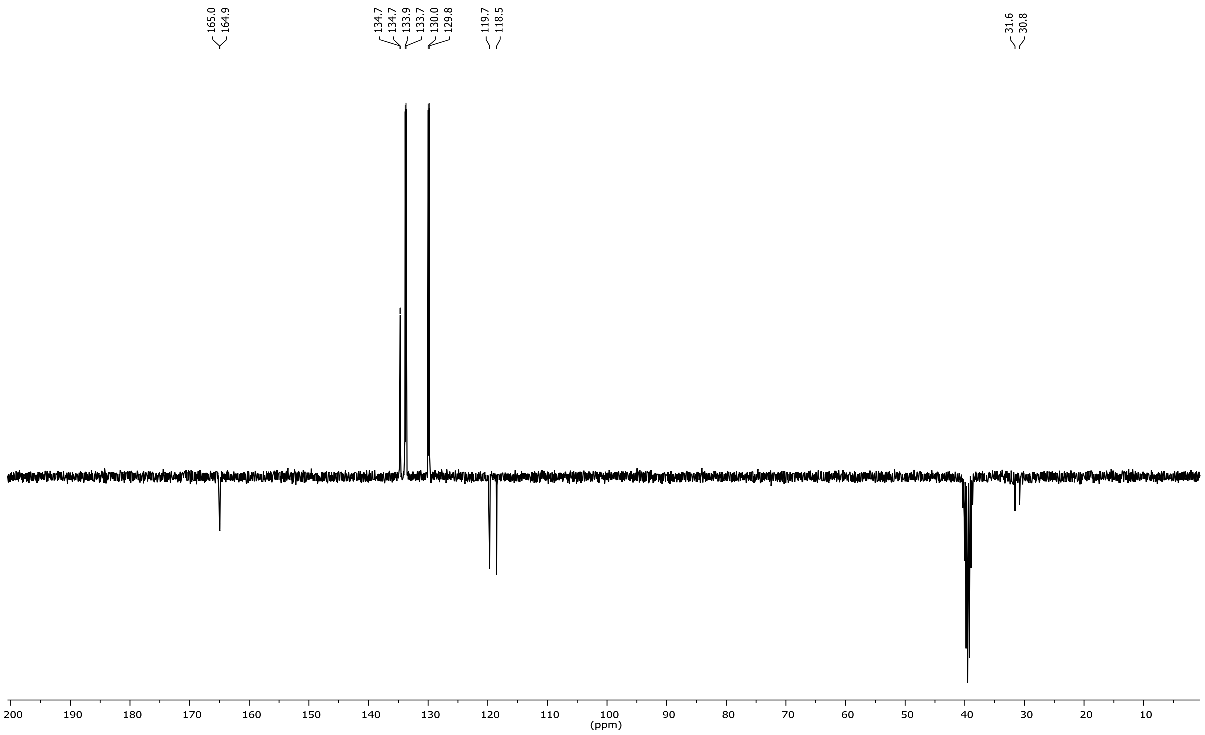

(*E*)-3-(2-bromo-5-iodophenyl)acrylamide (**25**)

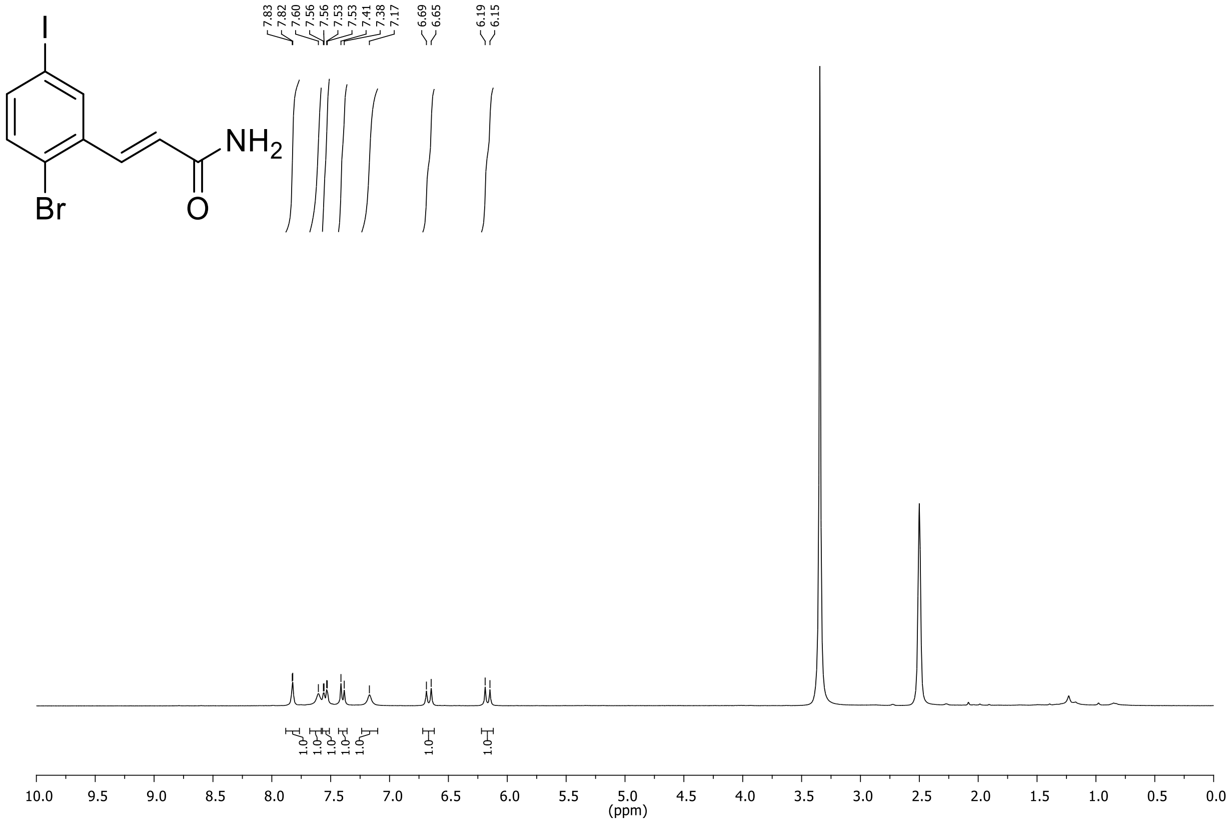

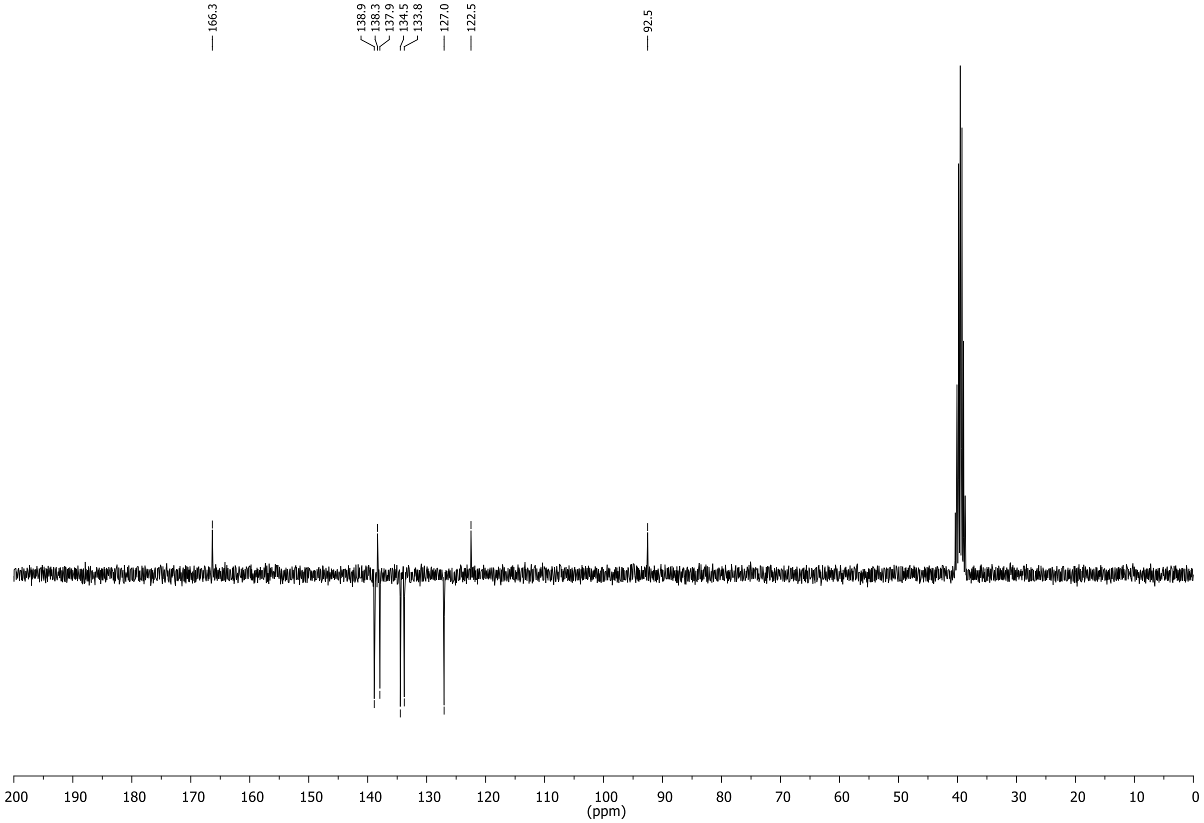

3-(2-Bromo-5-iodophenyl)propanamide **(27)**

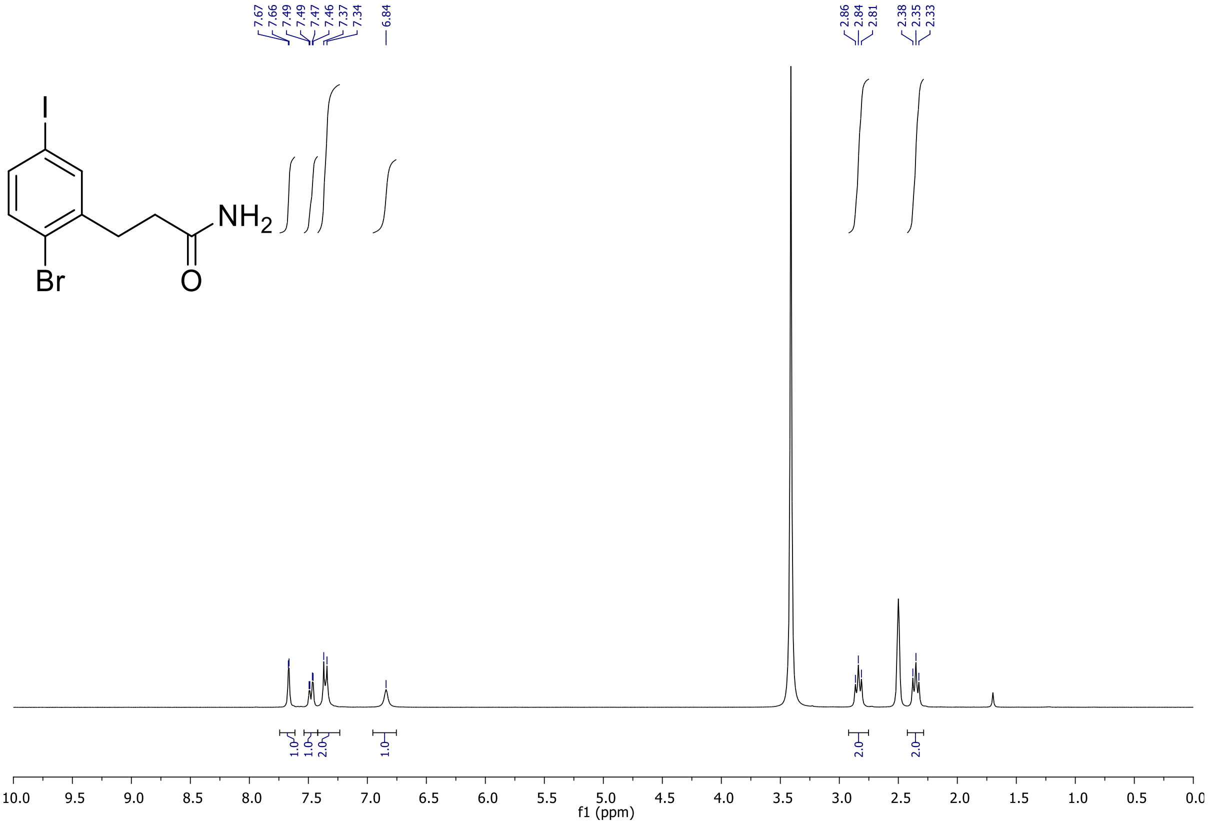

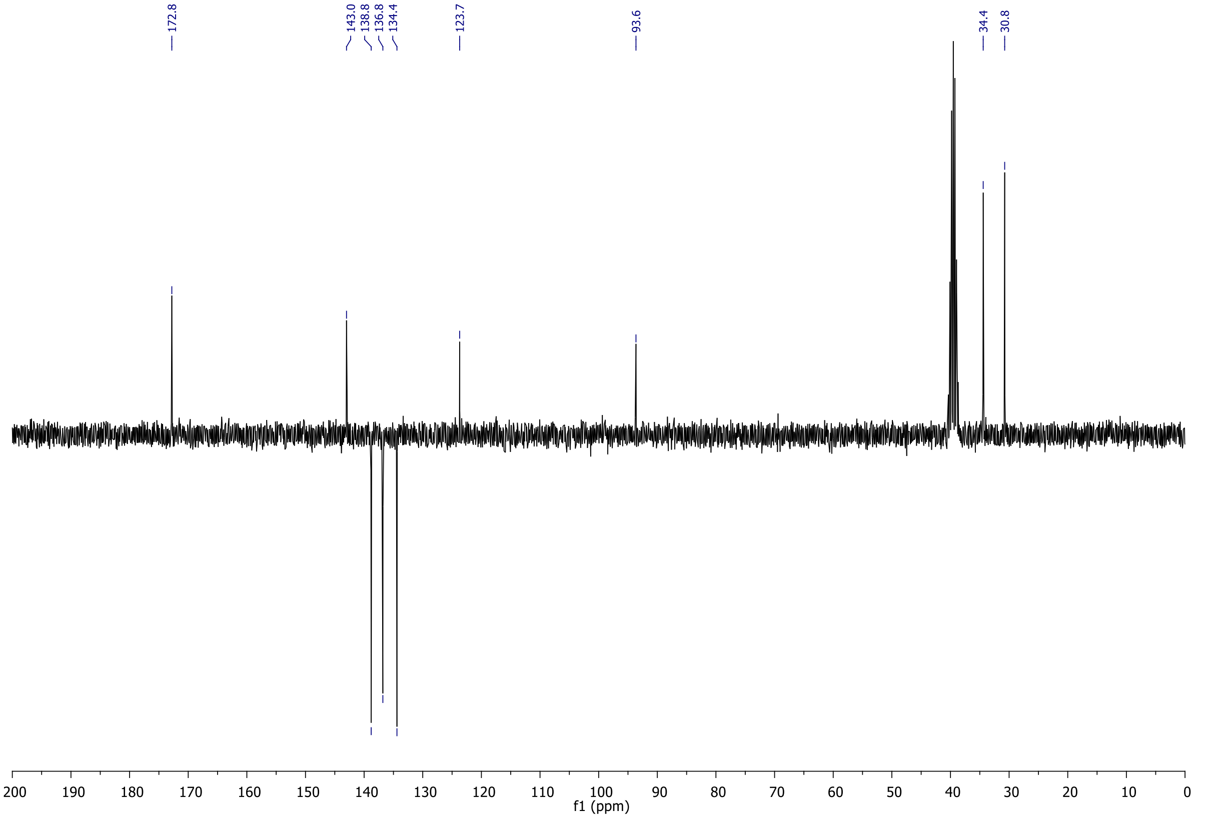

Ethyl 3-(2-bromo-5-iodophenyl)acrylate (**29**)

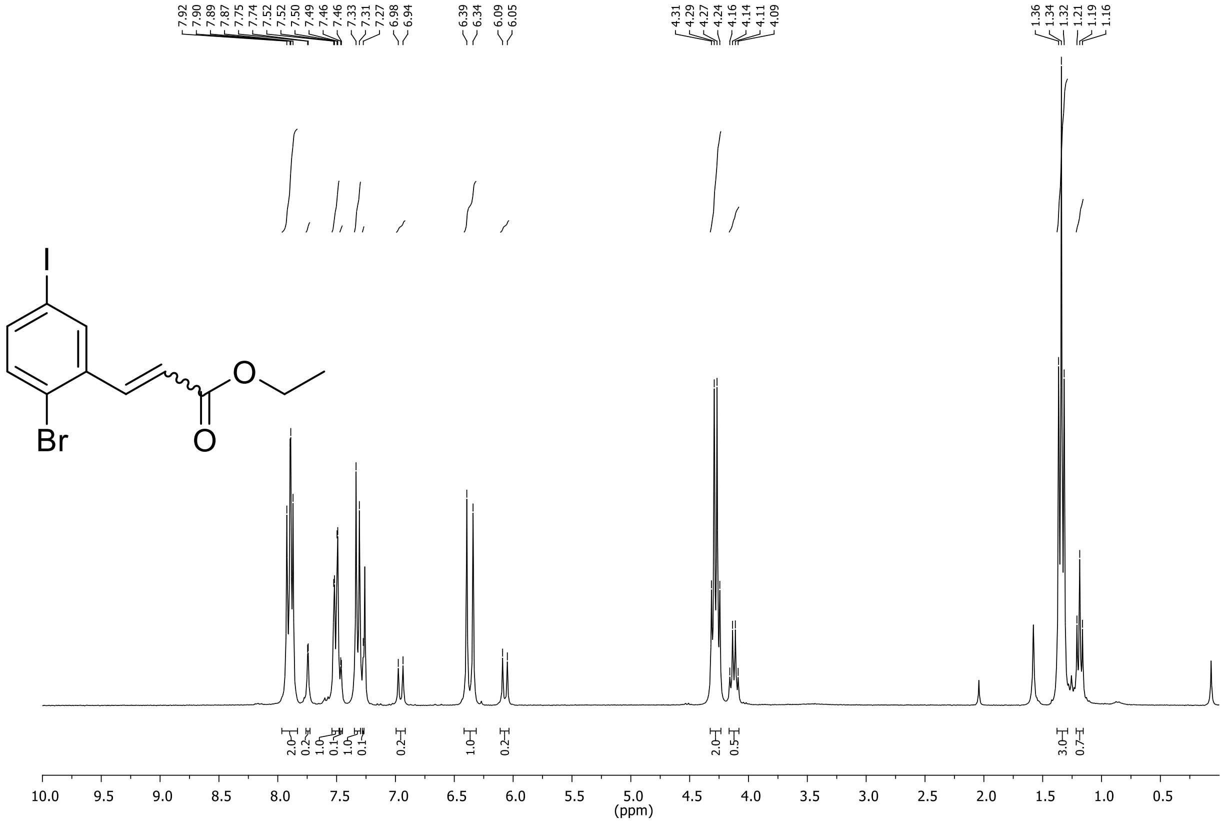

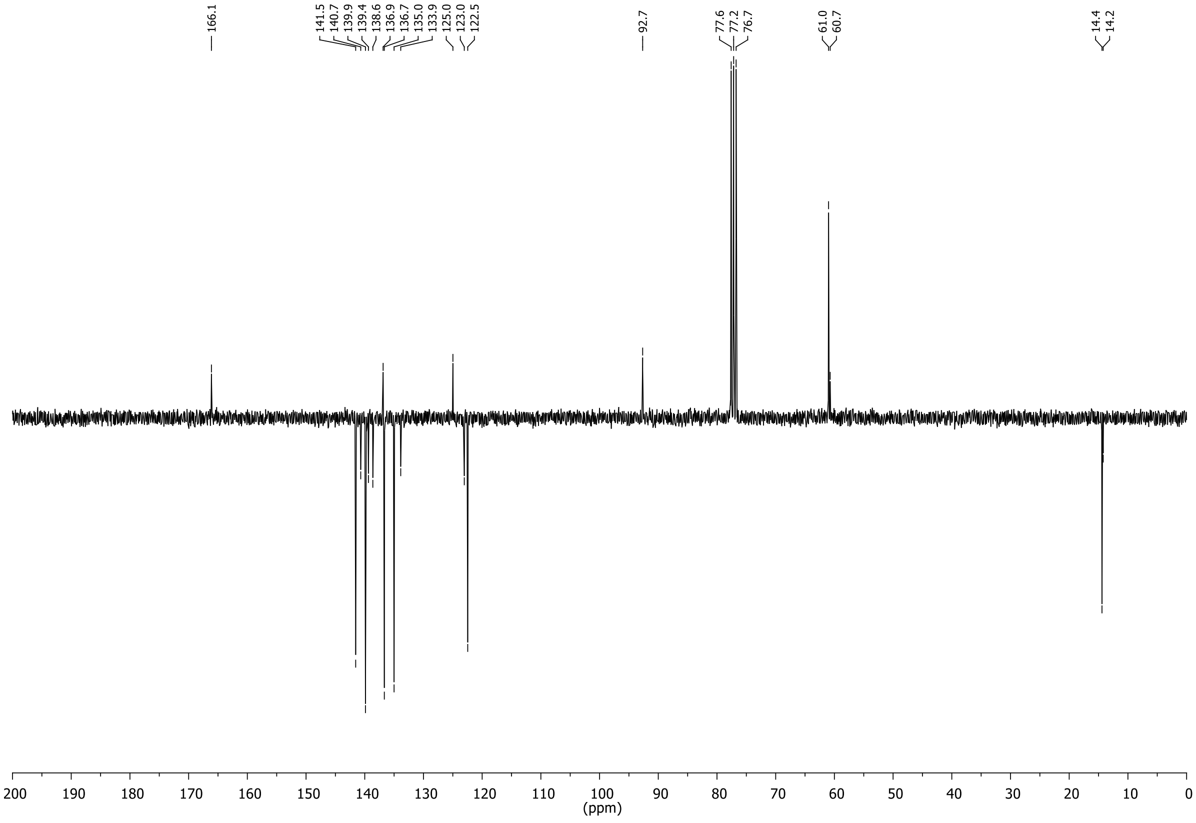

Ethyl 3-(2-bromo-5-iodophenyl)propanoate (**30**)

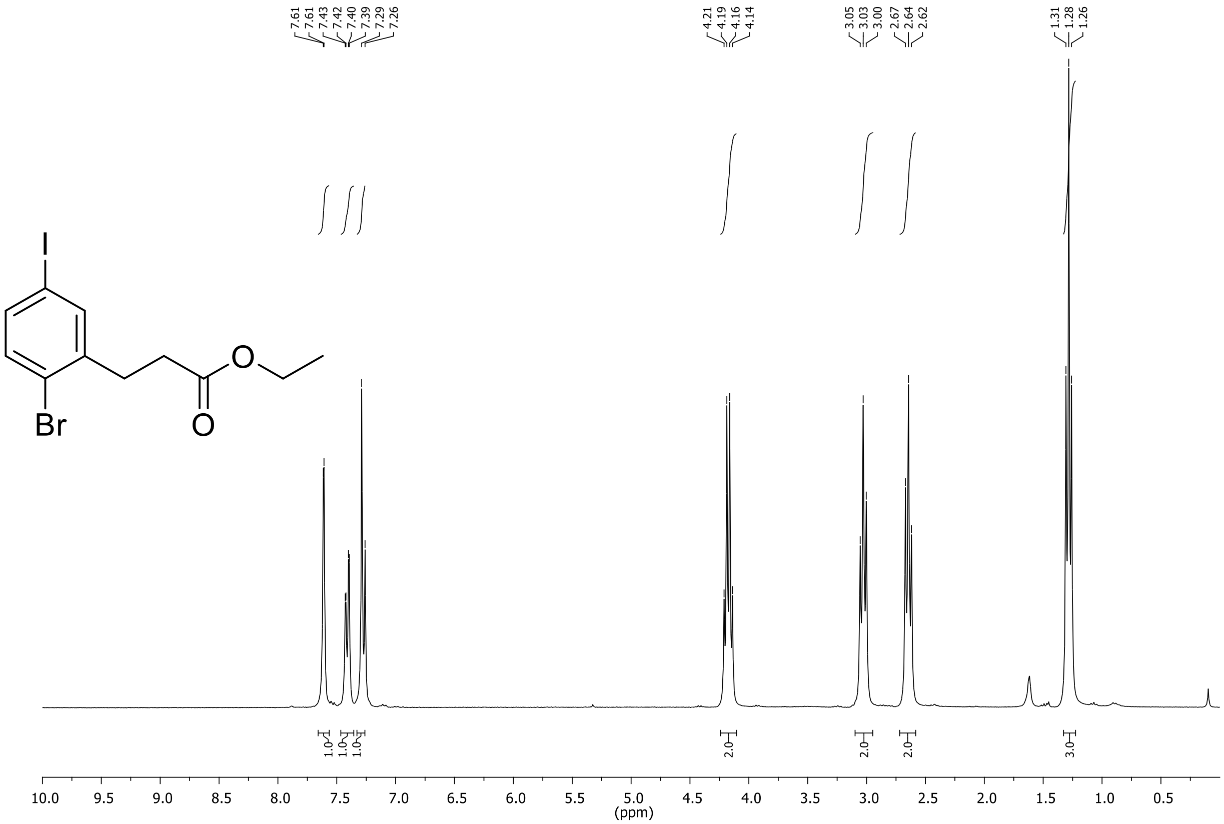

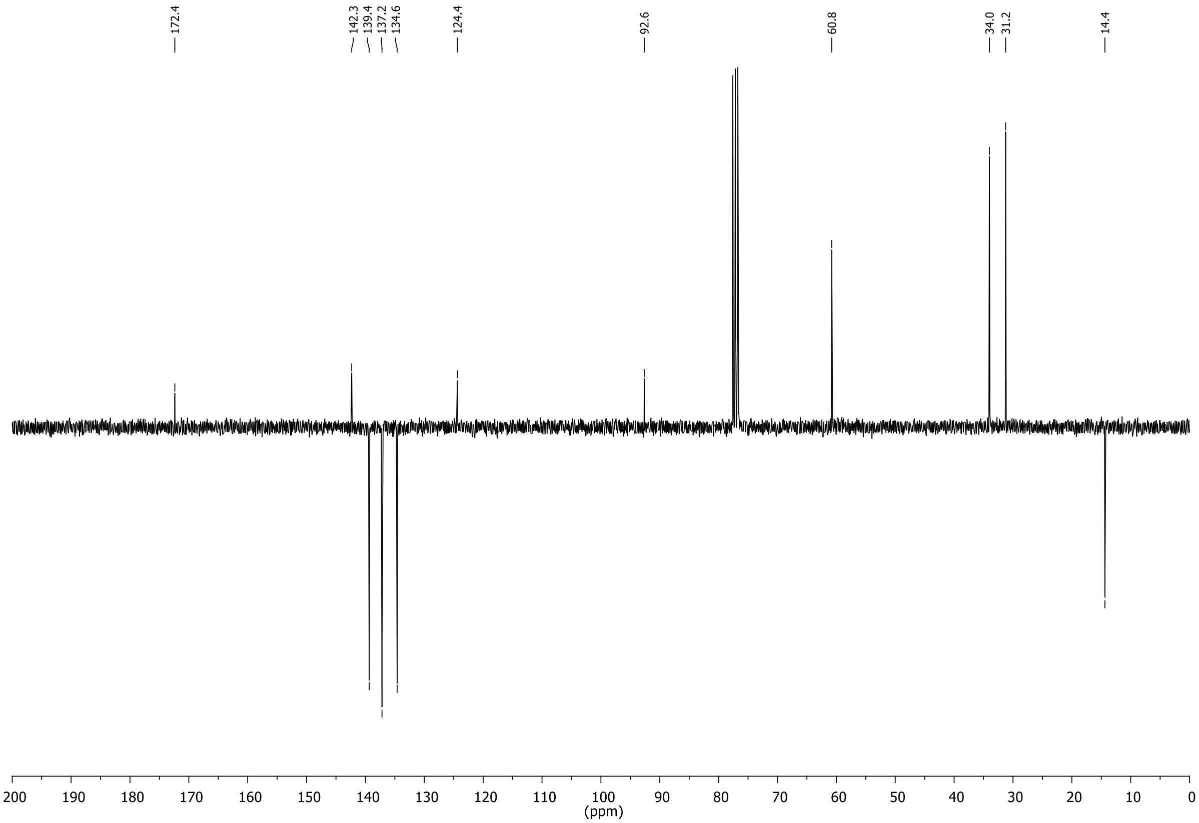

(2-Bromo-5-iodophenyl)(phenyl)methanone (**32**)

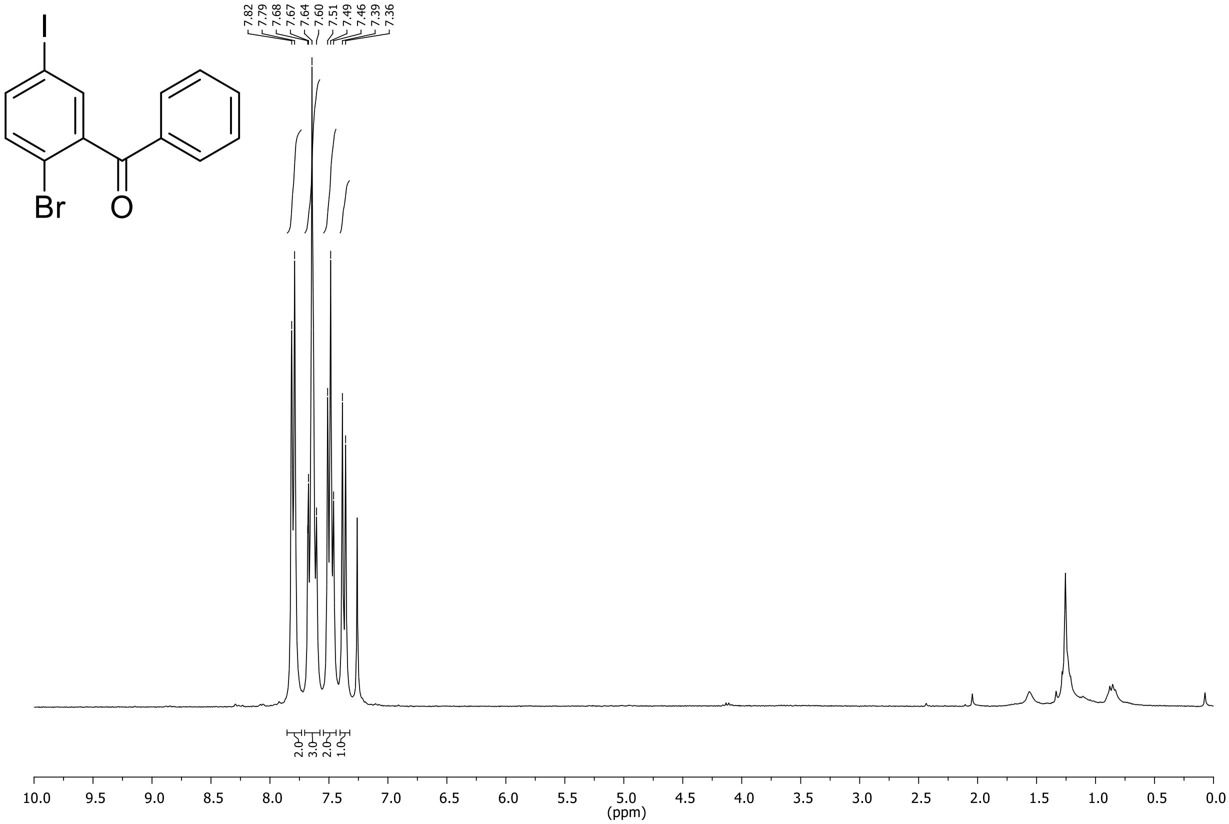

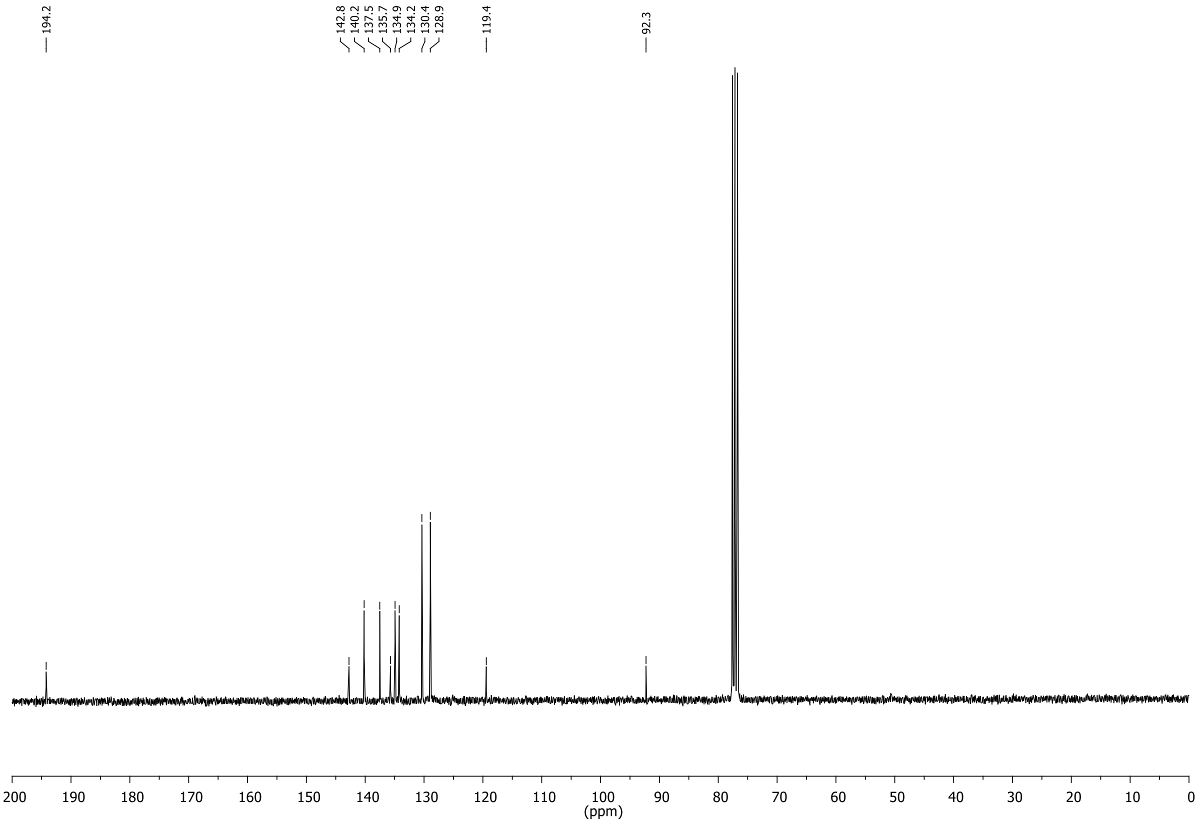

2-Benzyl-1-bromo-4-iodobenzene (**34**)

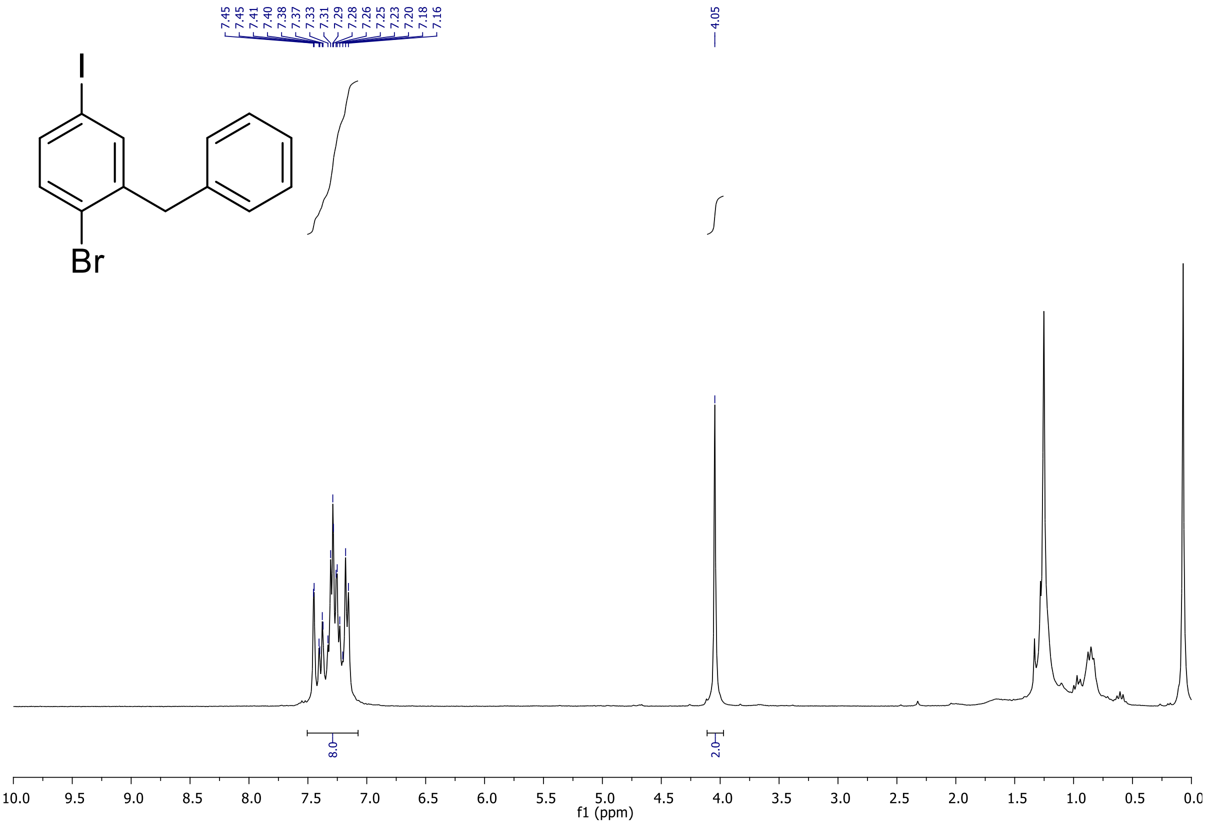

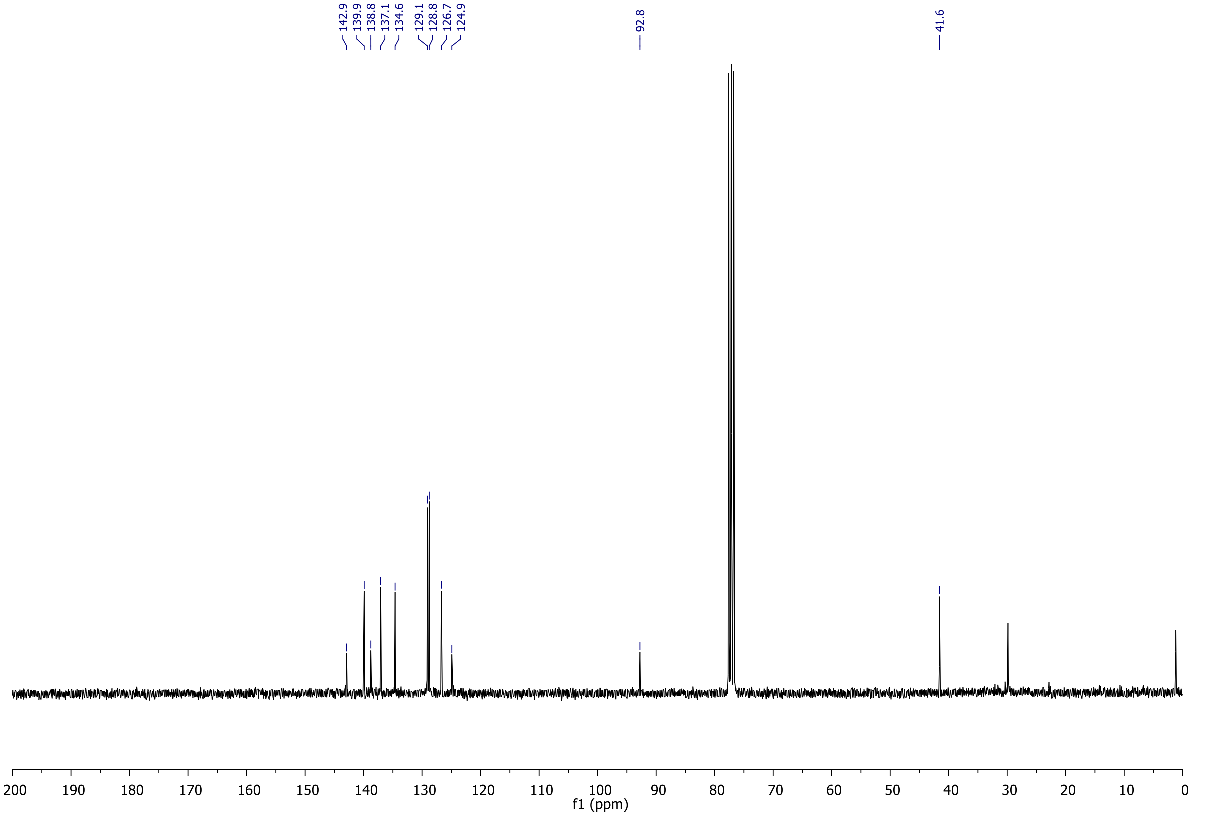

(2-Bromo-5-iodophenyl)(4-methoxyphenyl)methanone (**33**)

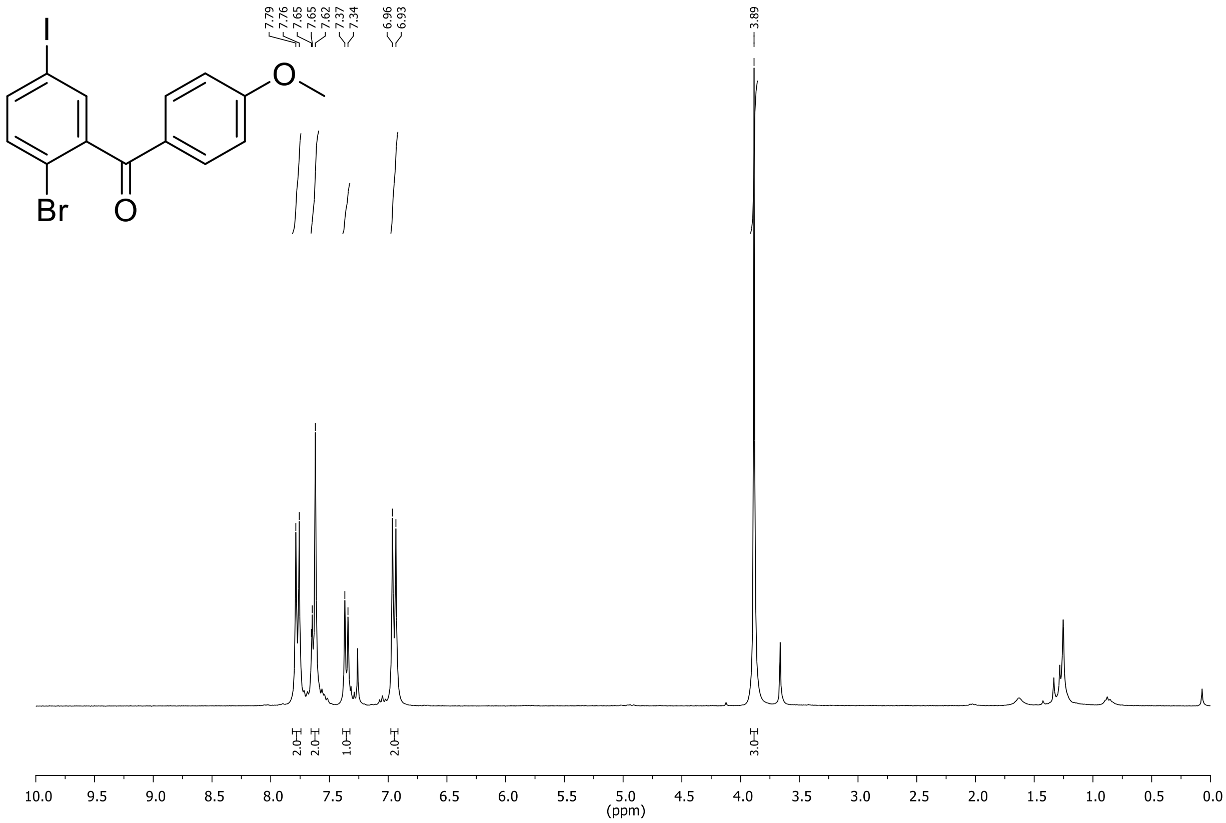

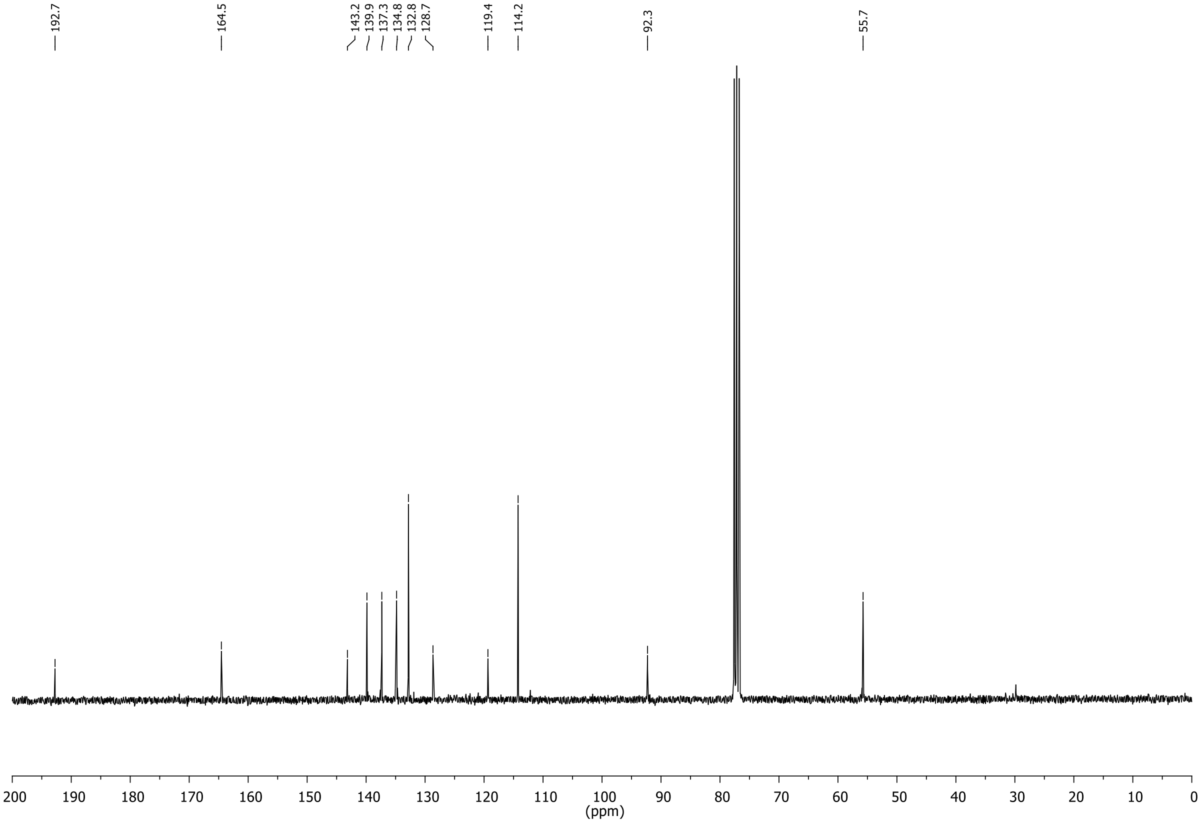

1-Bromo-4-iodo-2-(4-methoxybenzyl)benzene (**35**)

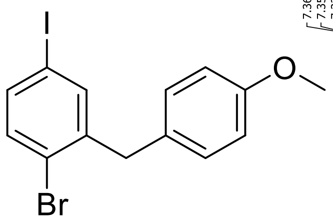

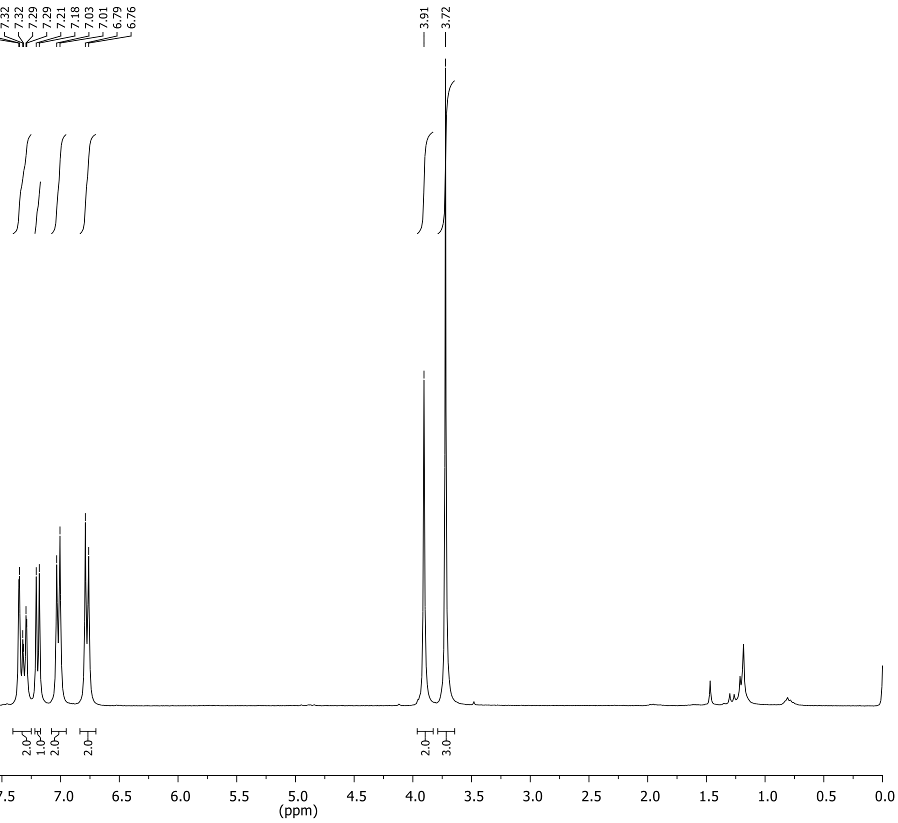

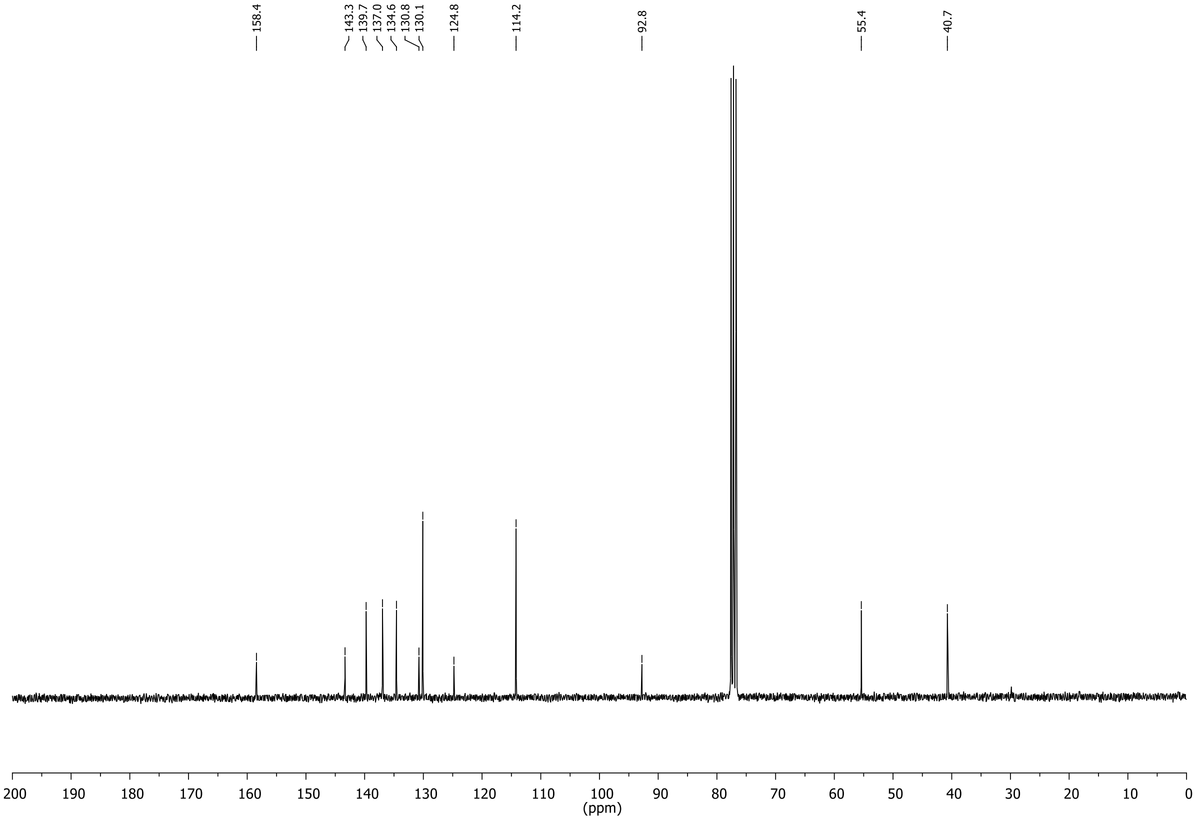

3-(2-bromo-5-iodophenyl)propan-1-ol **(36)**

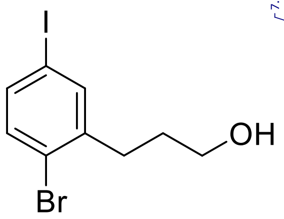

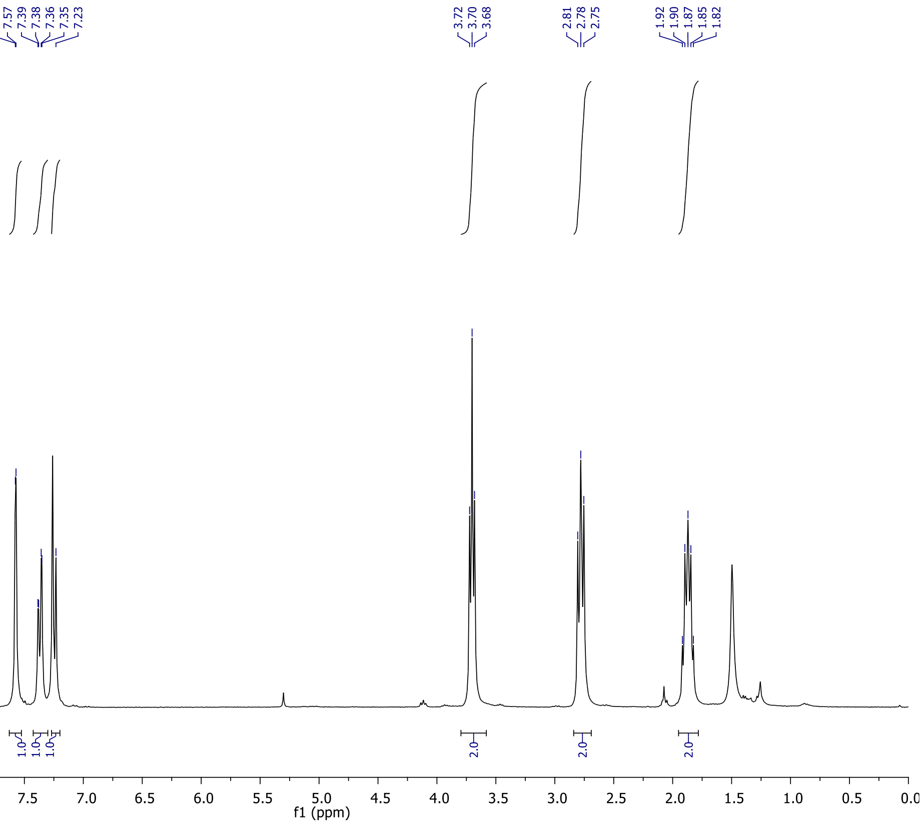

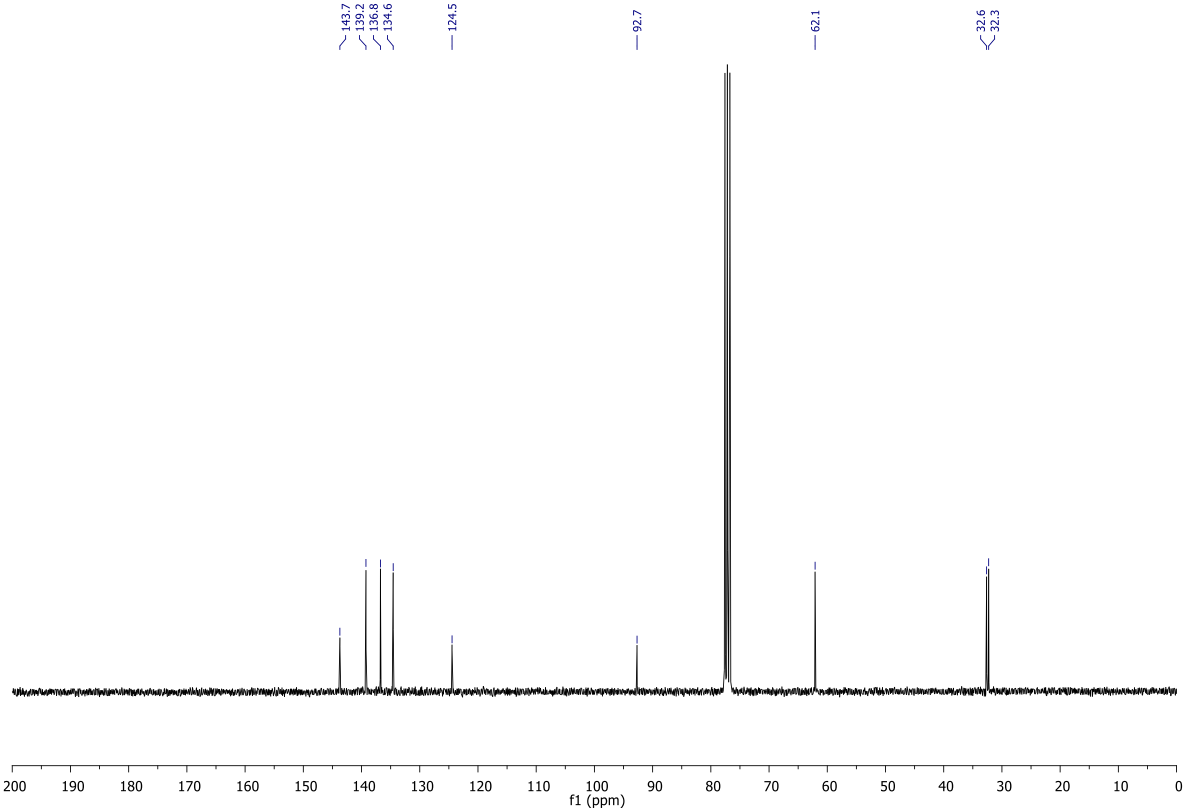

4-(2-bromo-5-iodophenyl)butanenitrile **(37)**

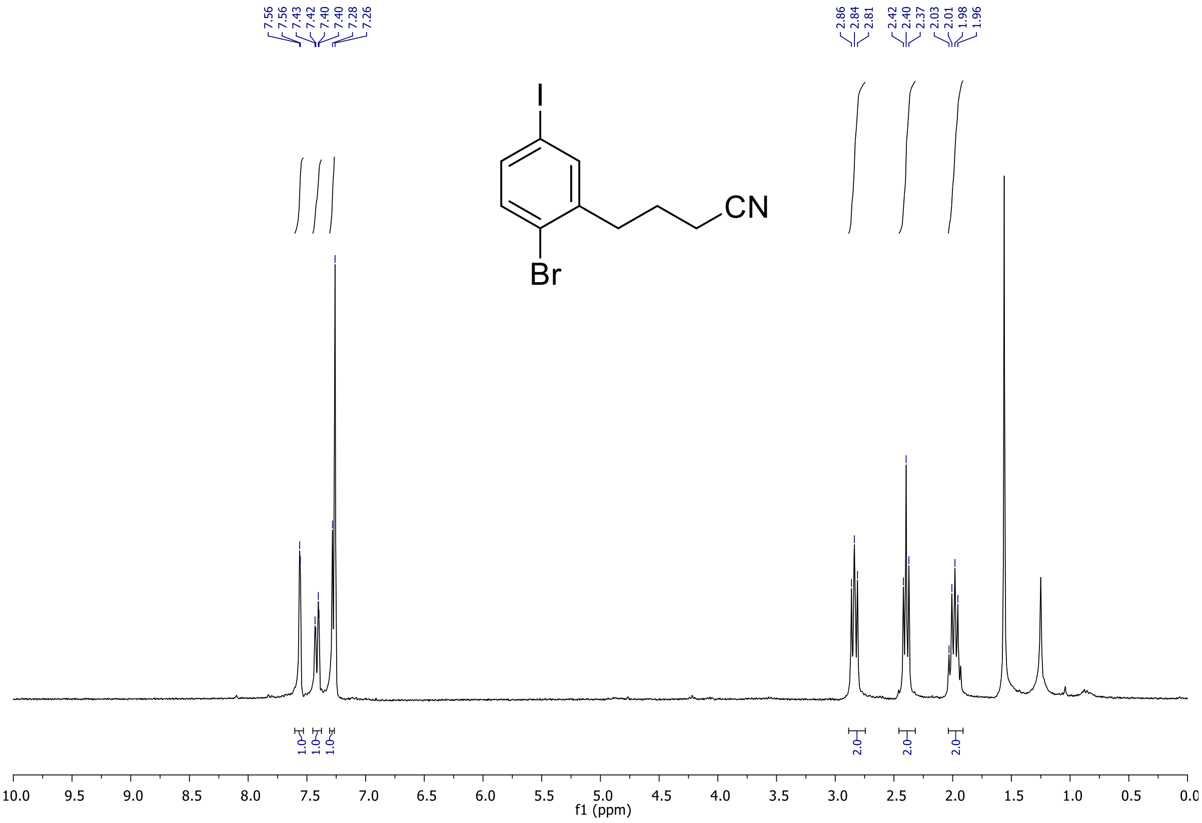

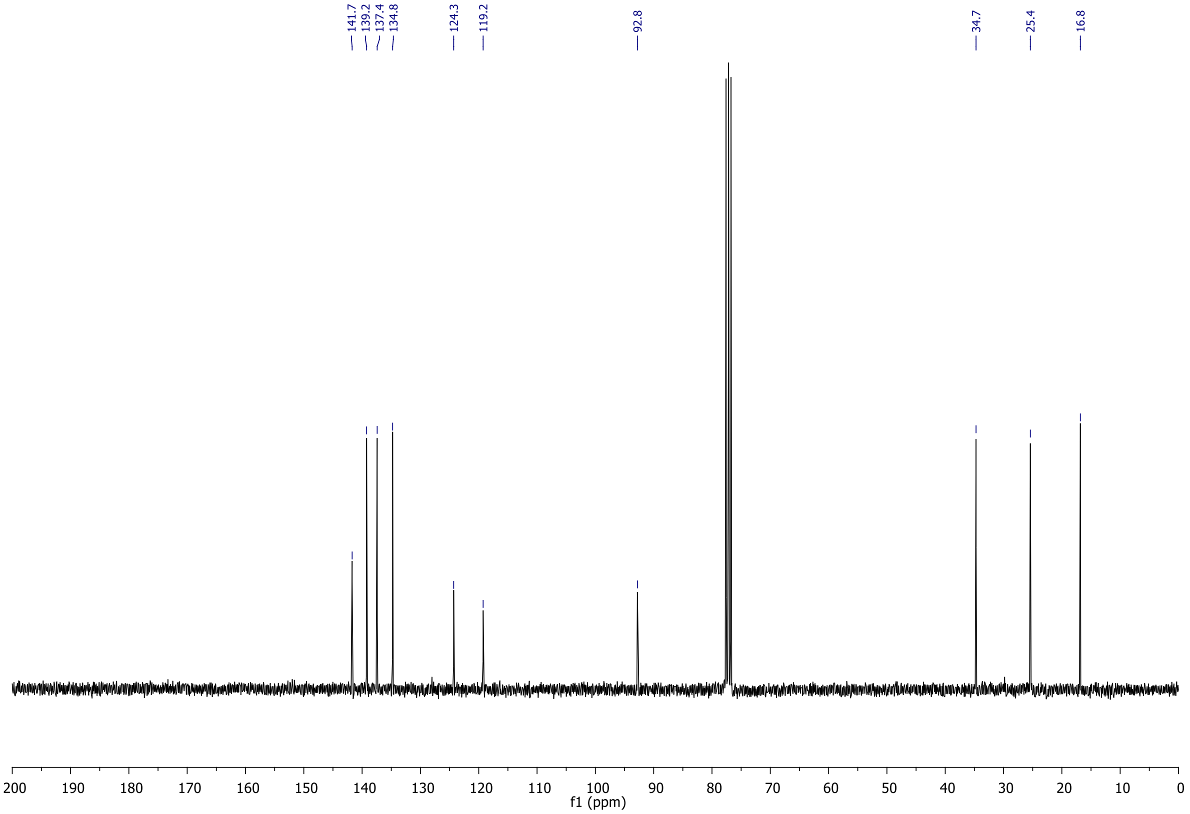

2-(2-Bromo-5-iodobenzyl)-1*H*-indole (**38**)

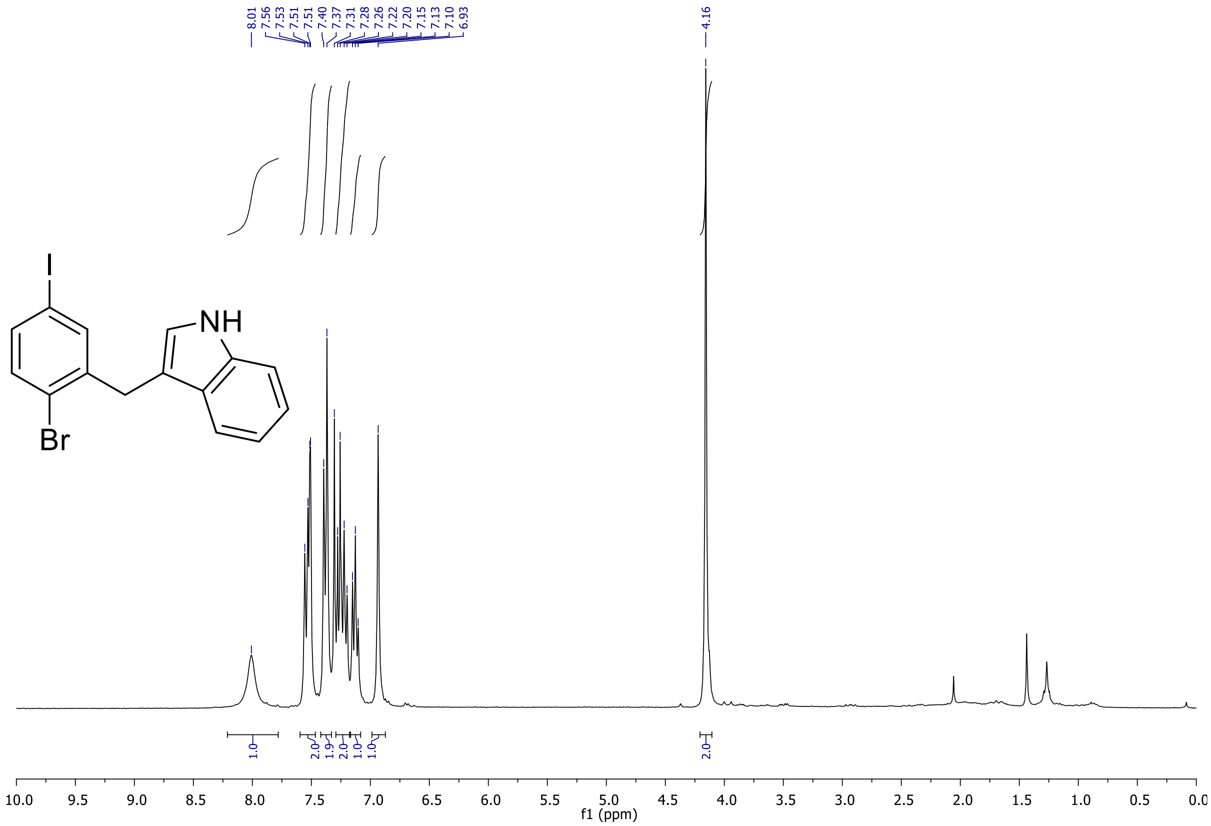

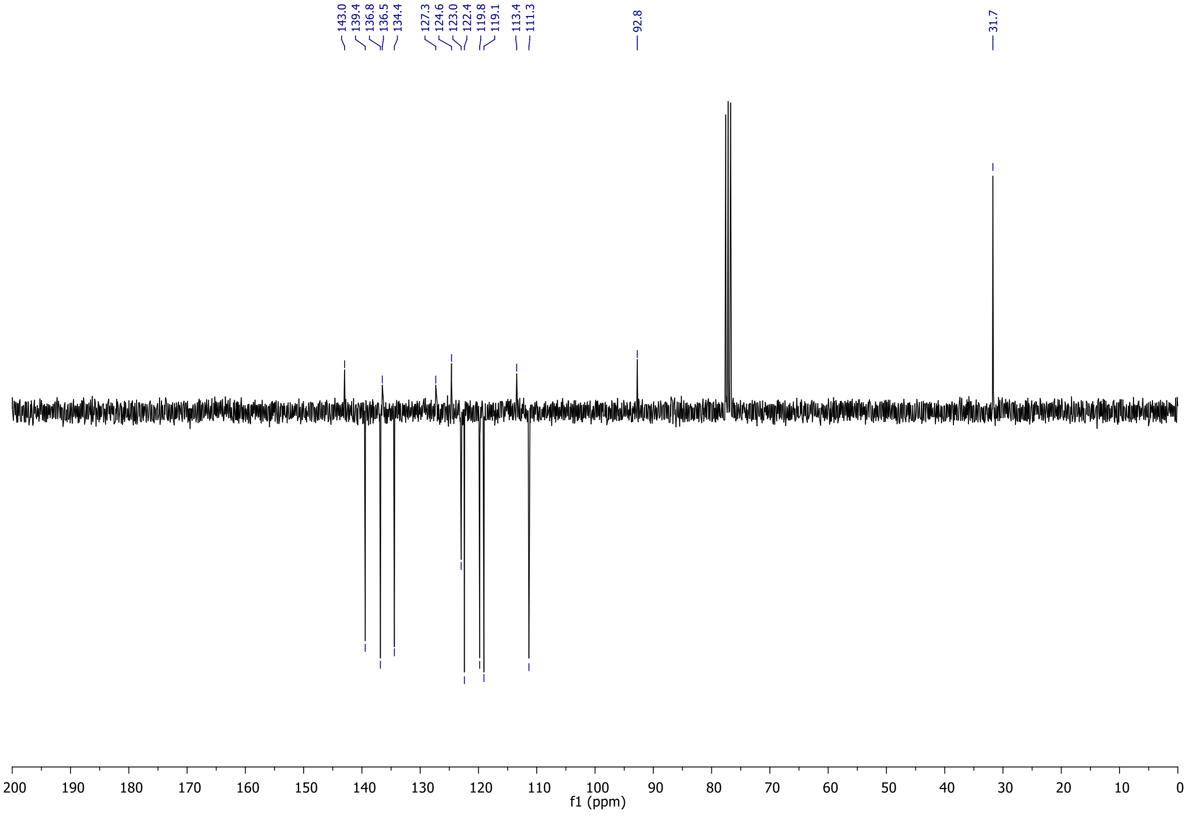

4-Iodo-1-trityl-1*H*-imidazole (**39**)

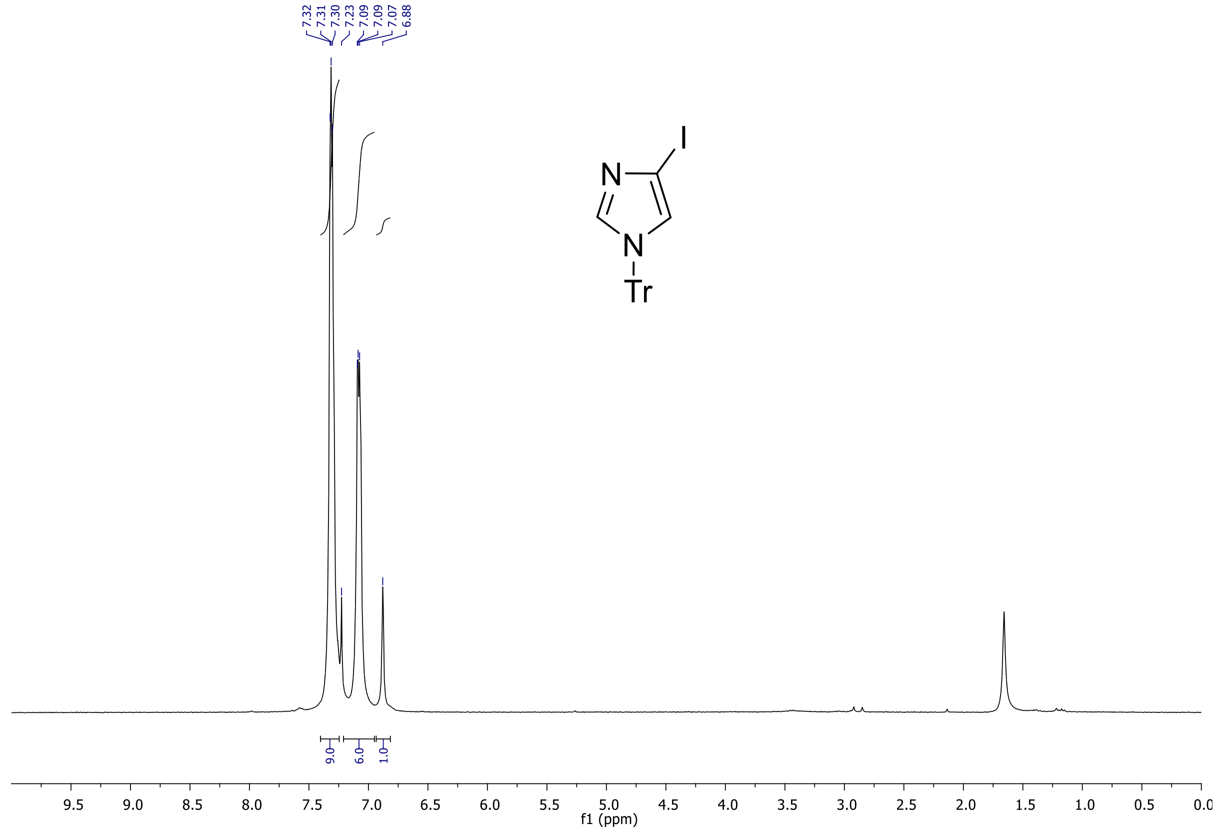

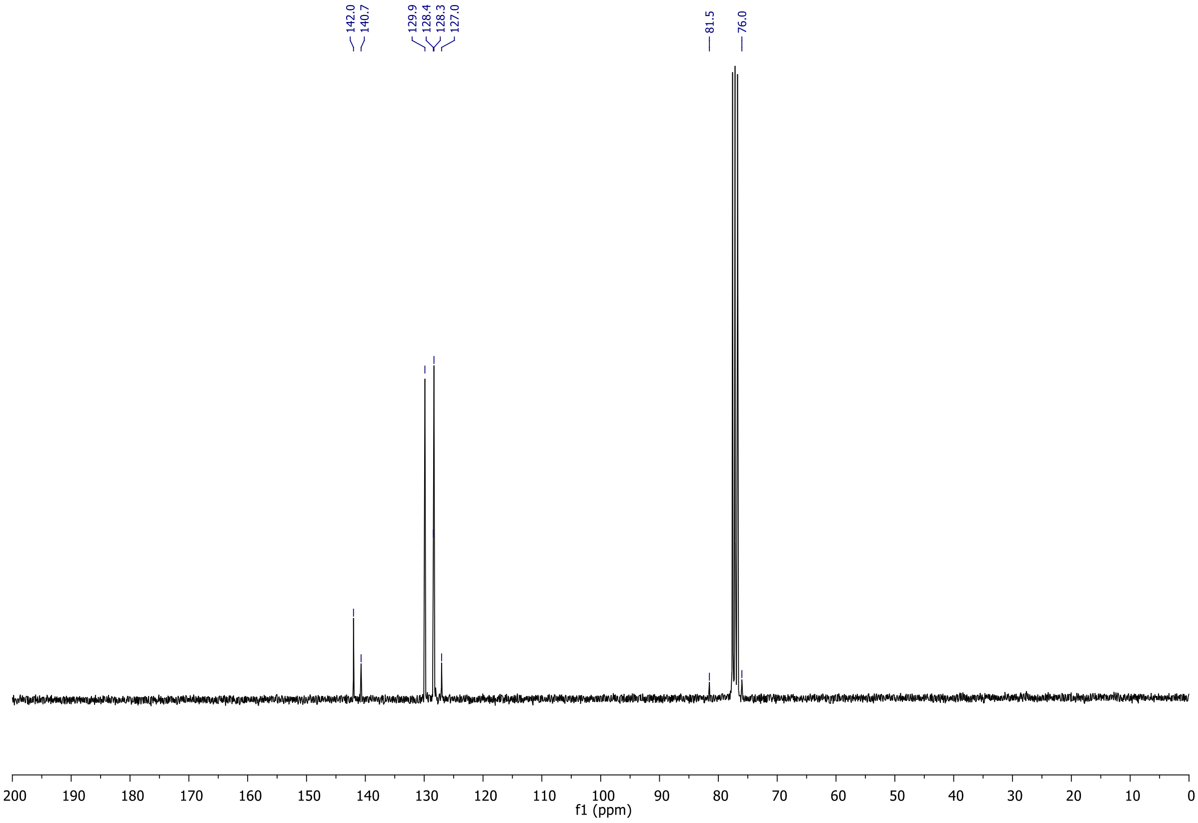

(2-Bromo-5-iodophenyl)(1-trityl-1*H*-imidazol-4-yl)methanol (**40**)

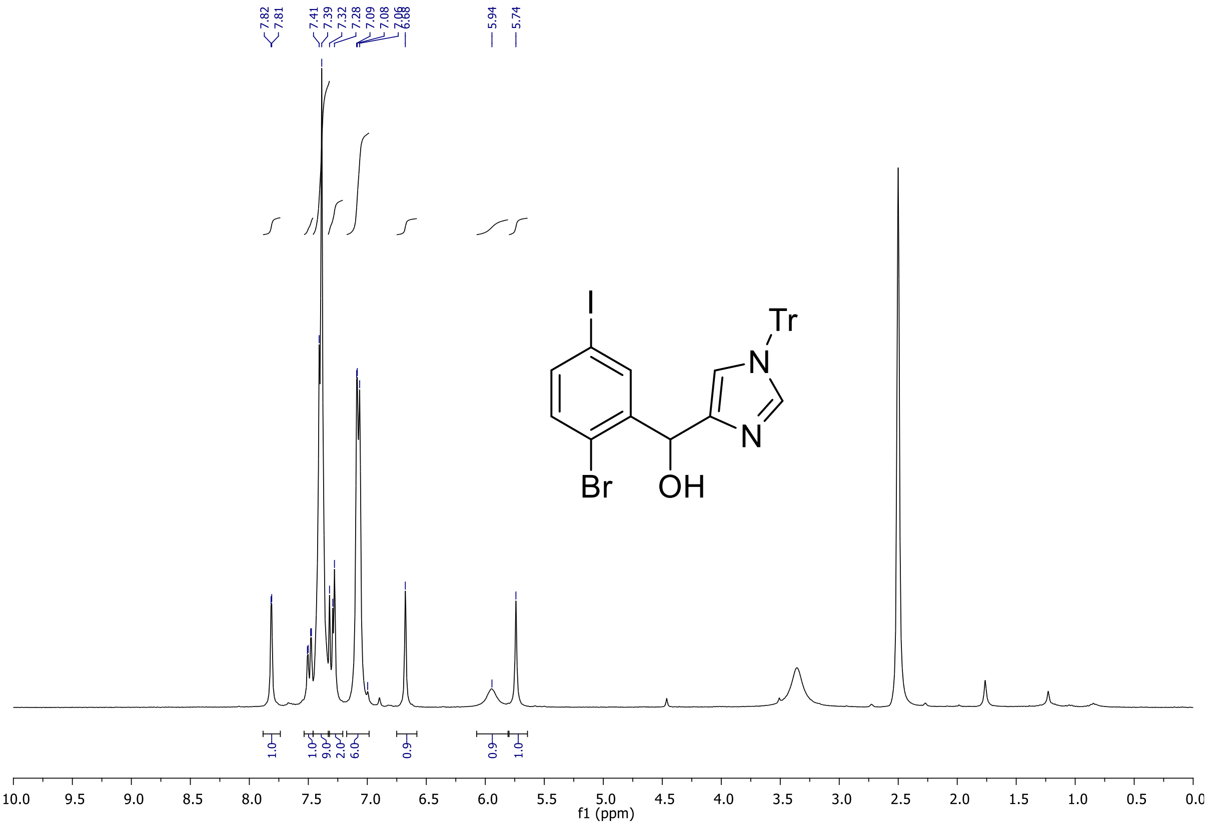

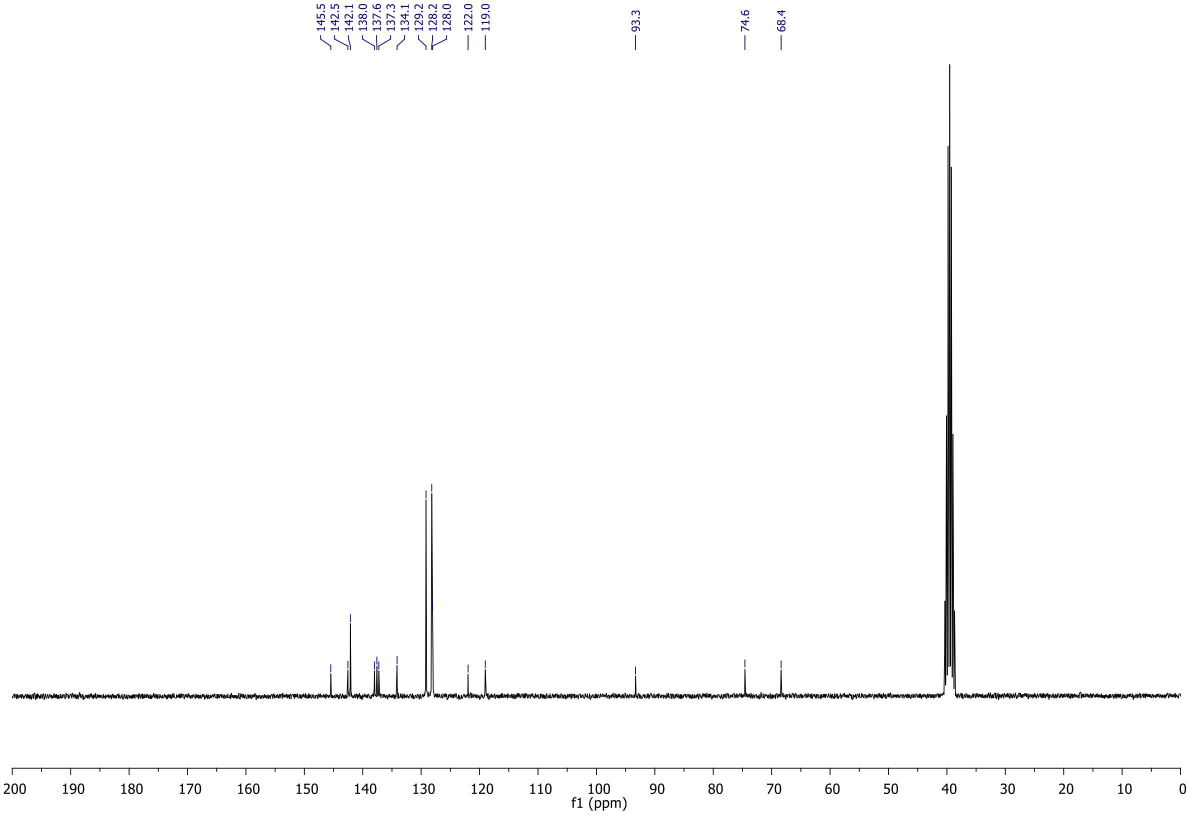

2-(2-Bromo-5-iodophenyl)acetaldehyde (**41**)

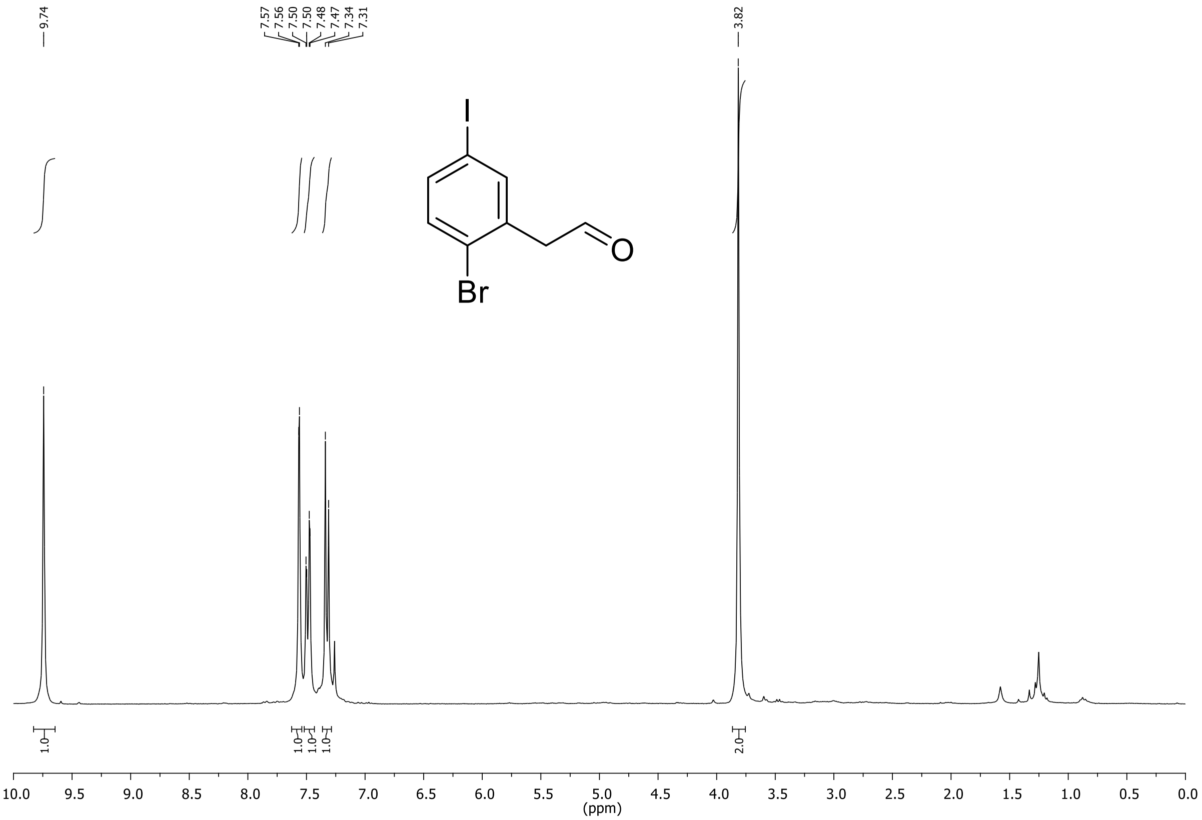

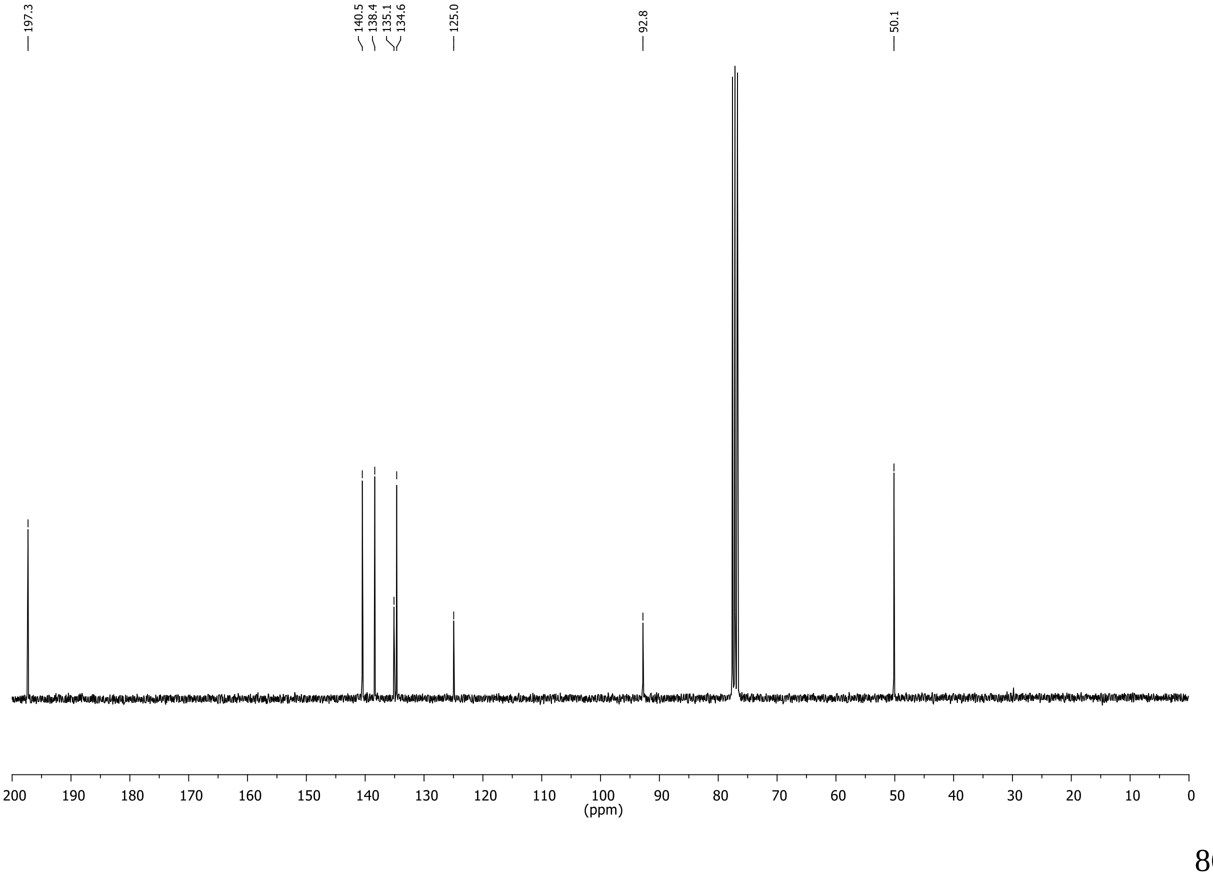

4-(2-bromo-5-iodobenzyl)-1H-imidazole **(42)**

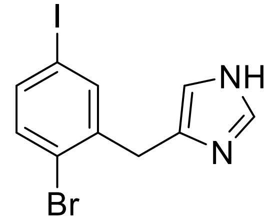

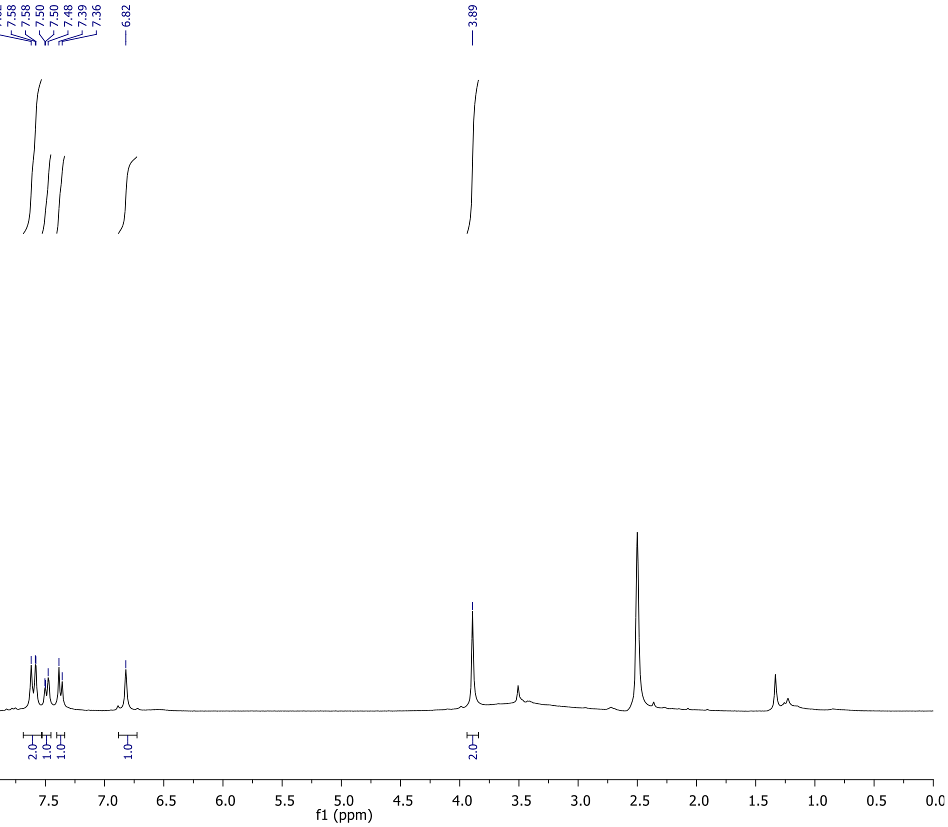

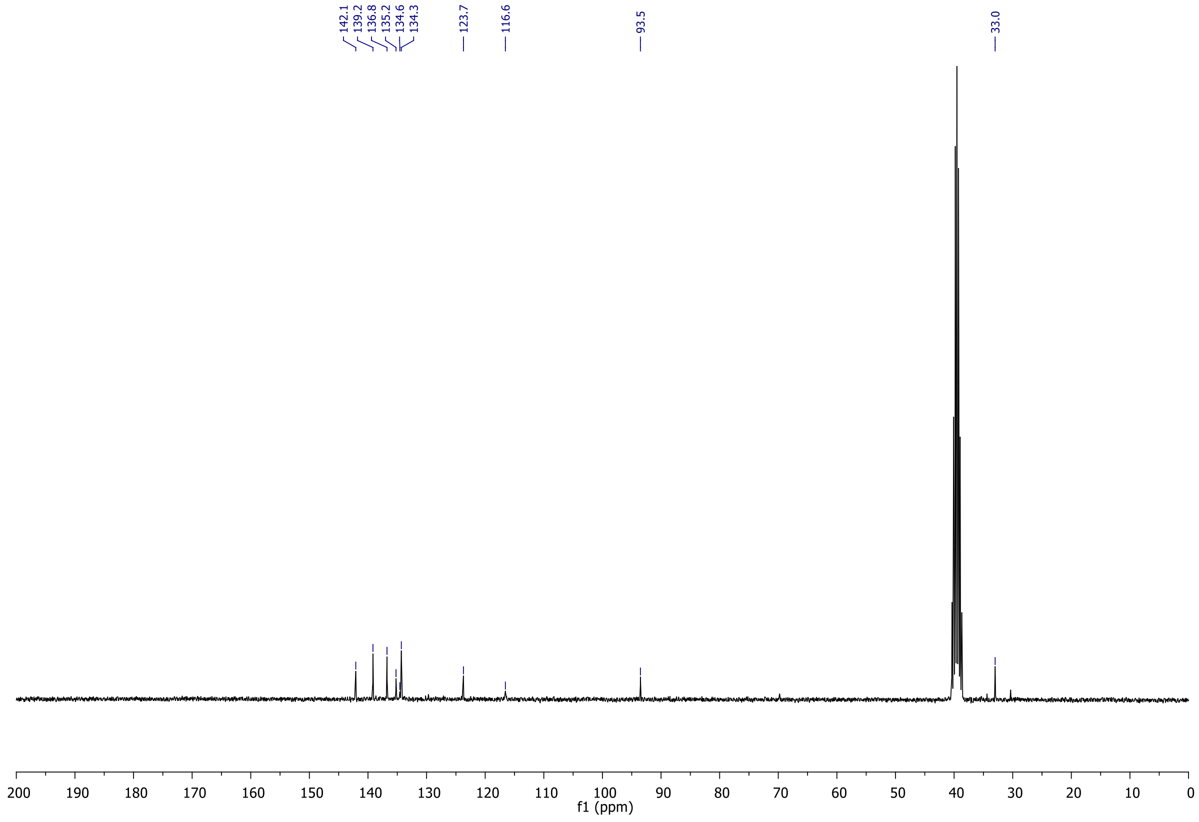

3-Chloro-5-isobutylpyridine (**43a**)

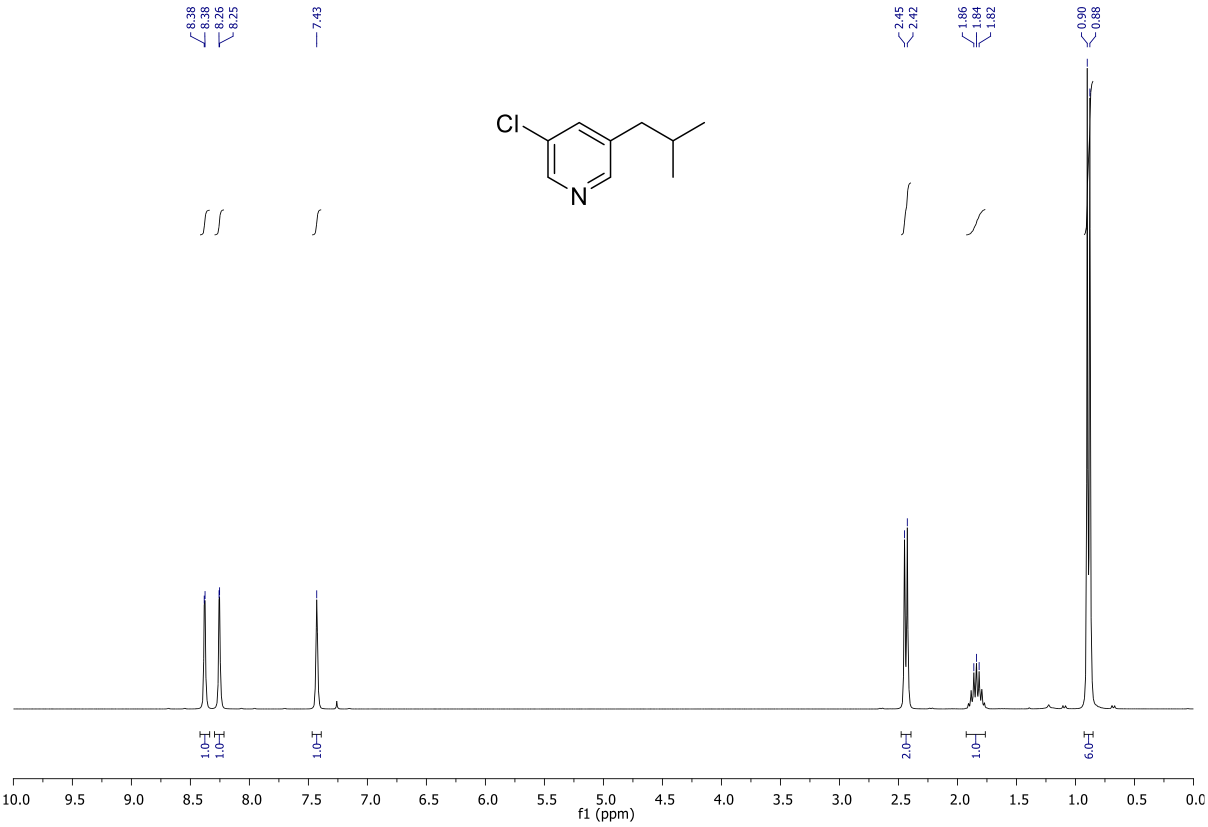

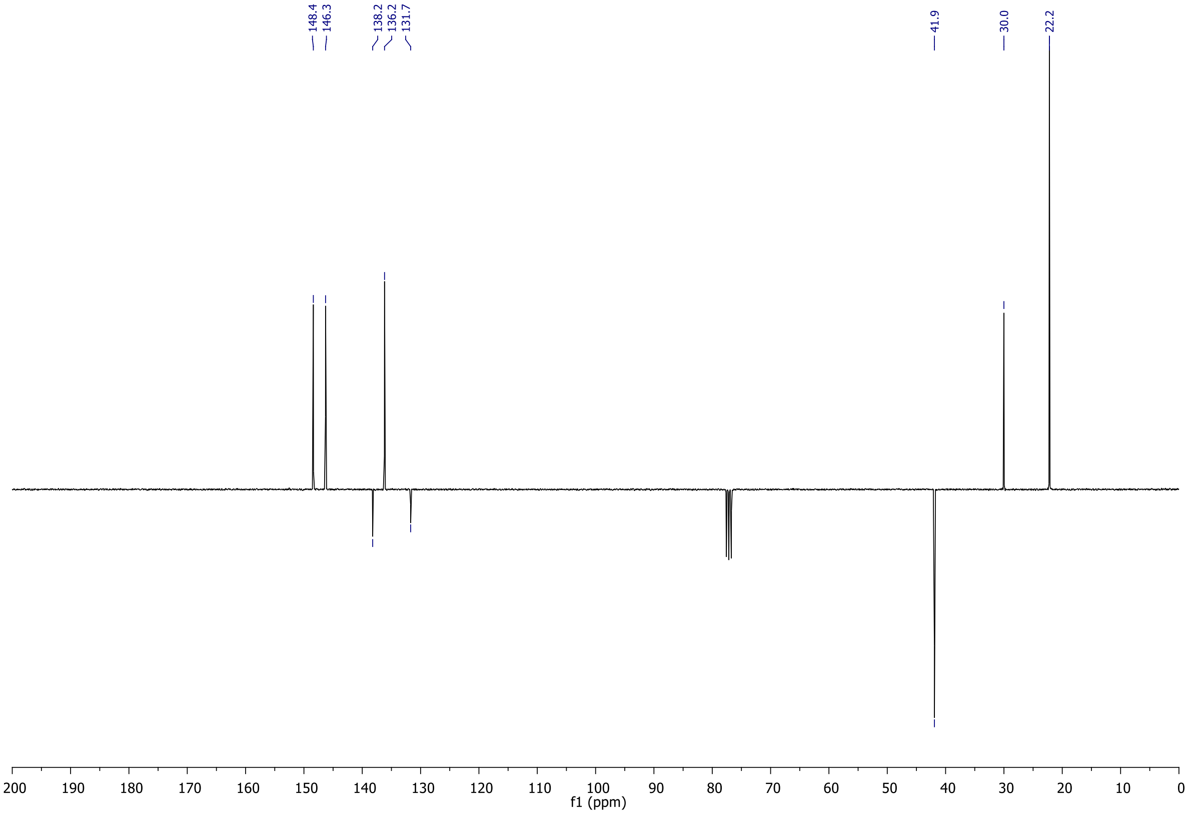

3-Isobutyl-5-(4,4,5,5-tetramethyl-1,3,2-dioxaborolan-2-yl)pyridine (**43**)

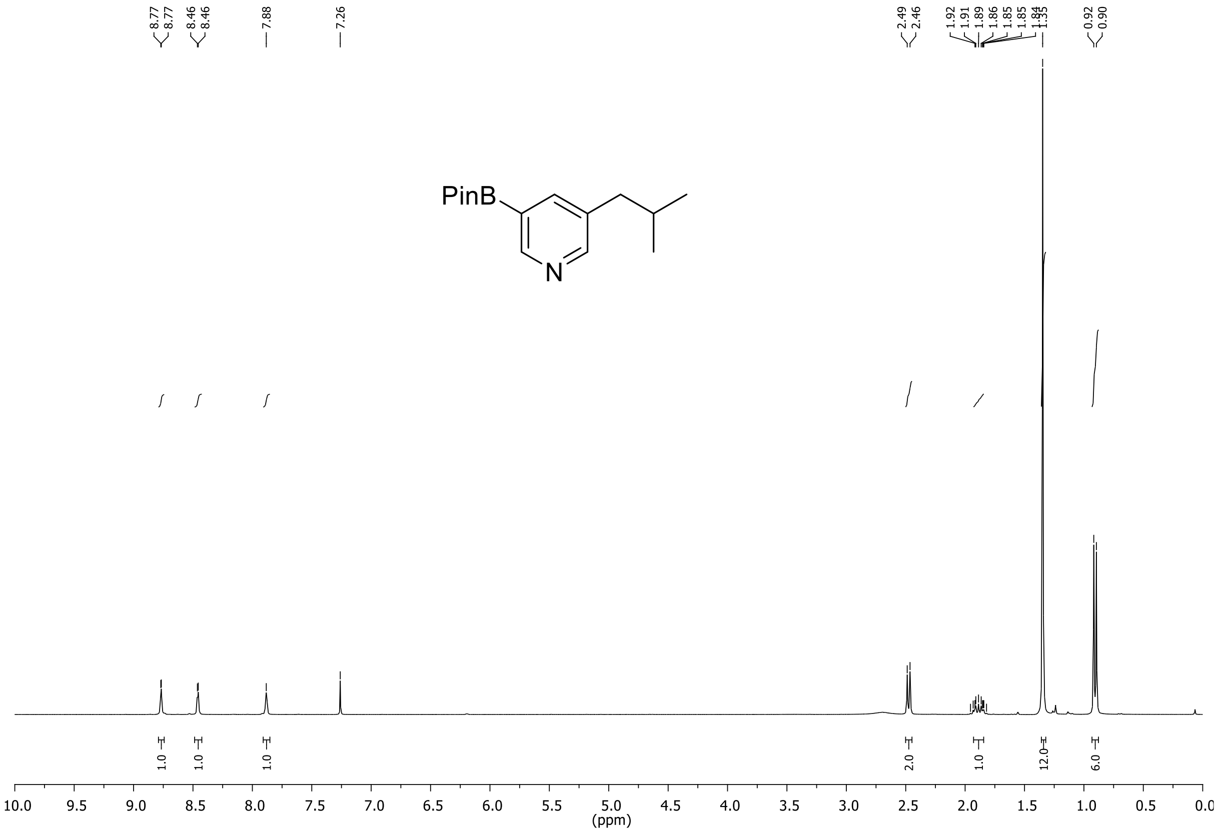

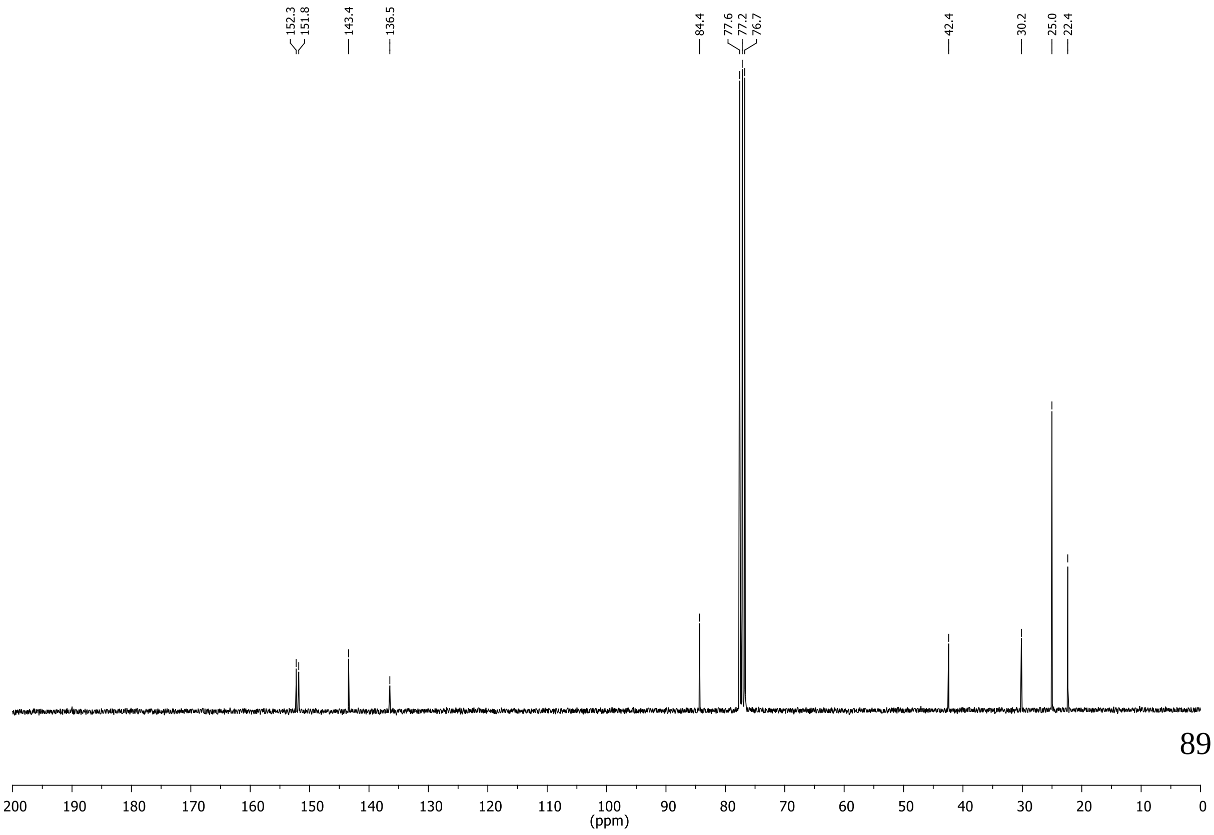

5-Iodonicotinaldehyde **(44a)**

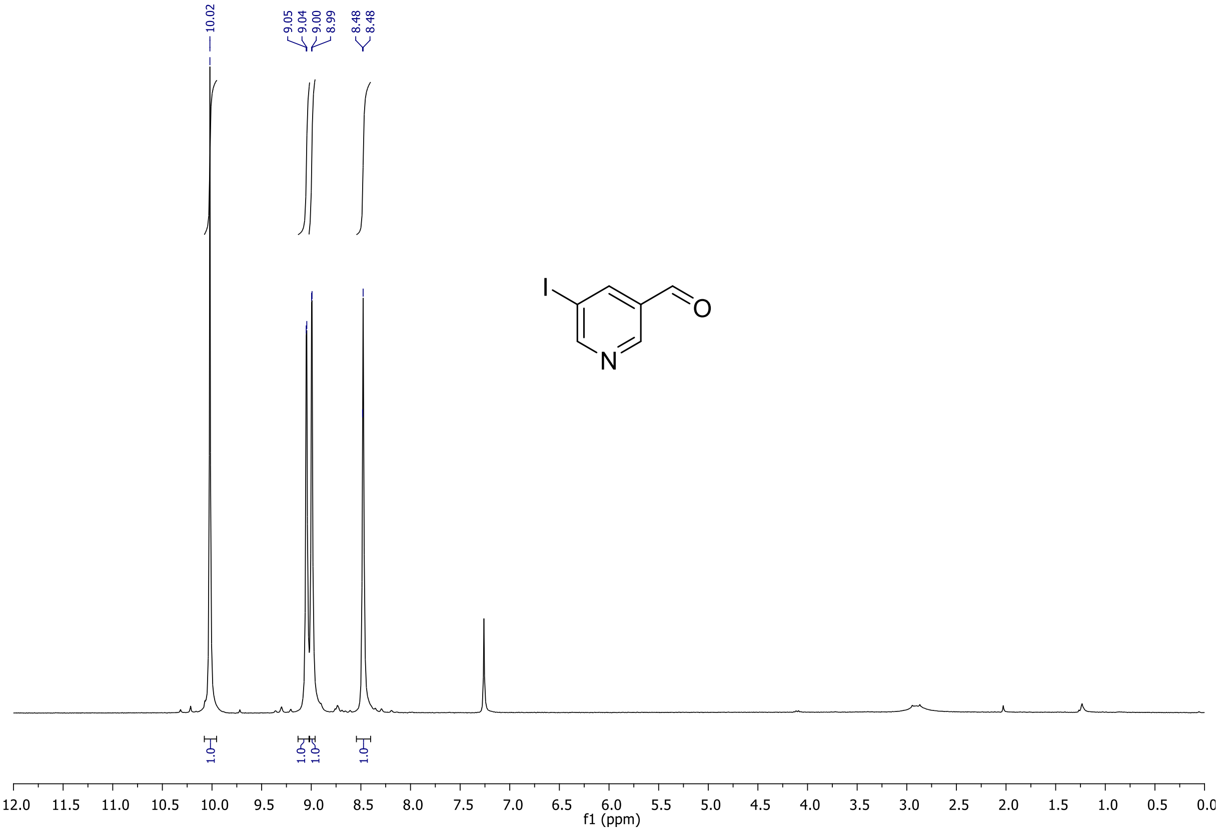

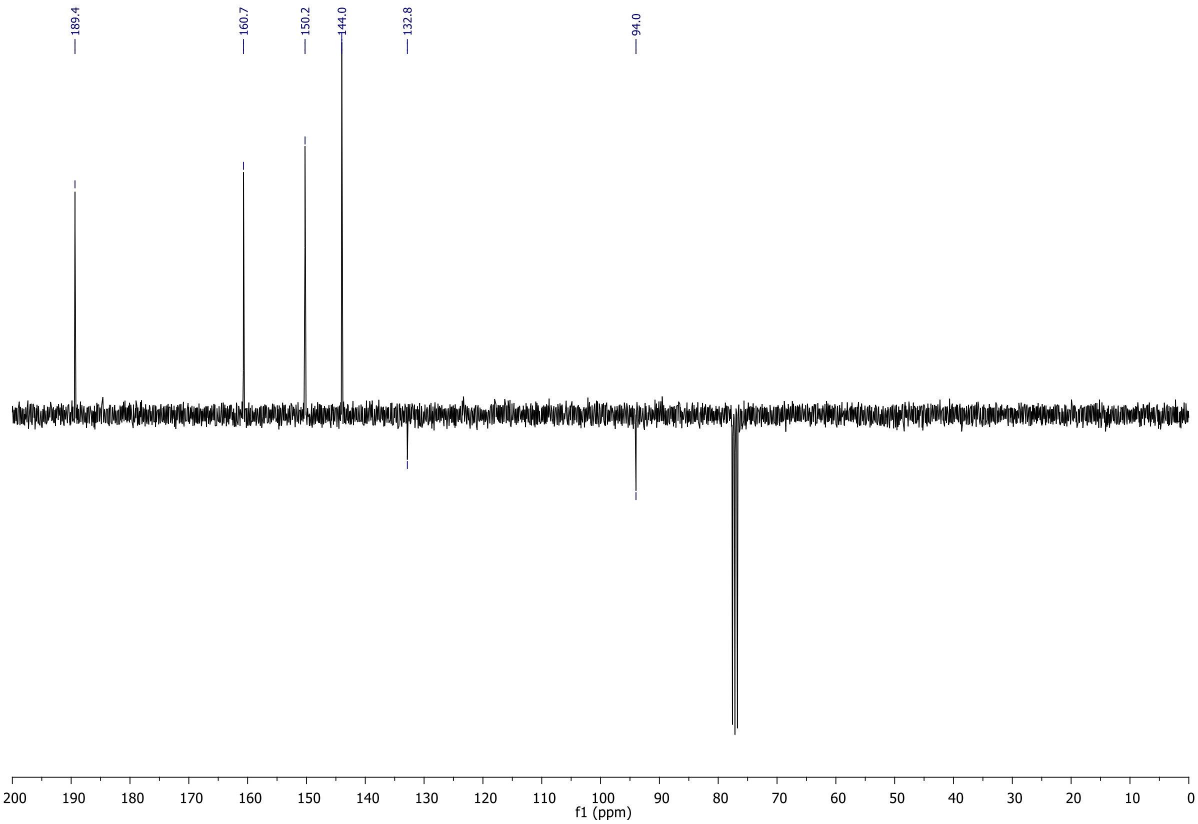

(E,Z)-3-Iodo-5-(2-(methylthio)vinyl)pyridine (**44b**)

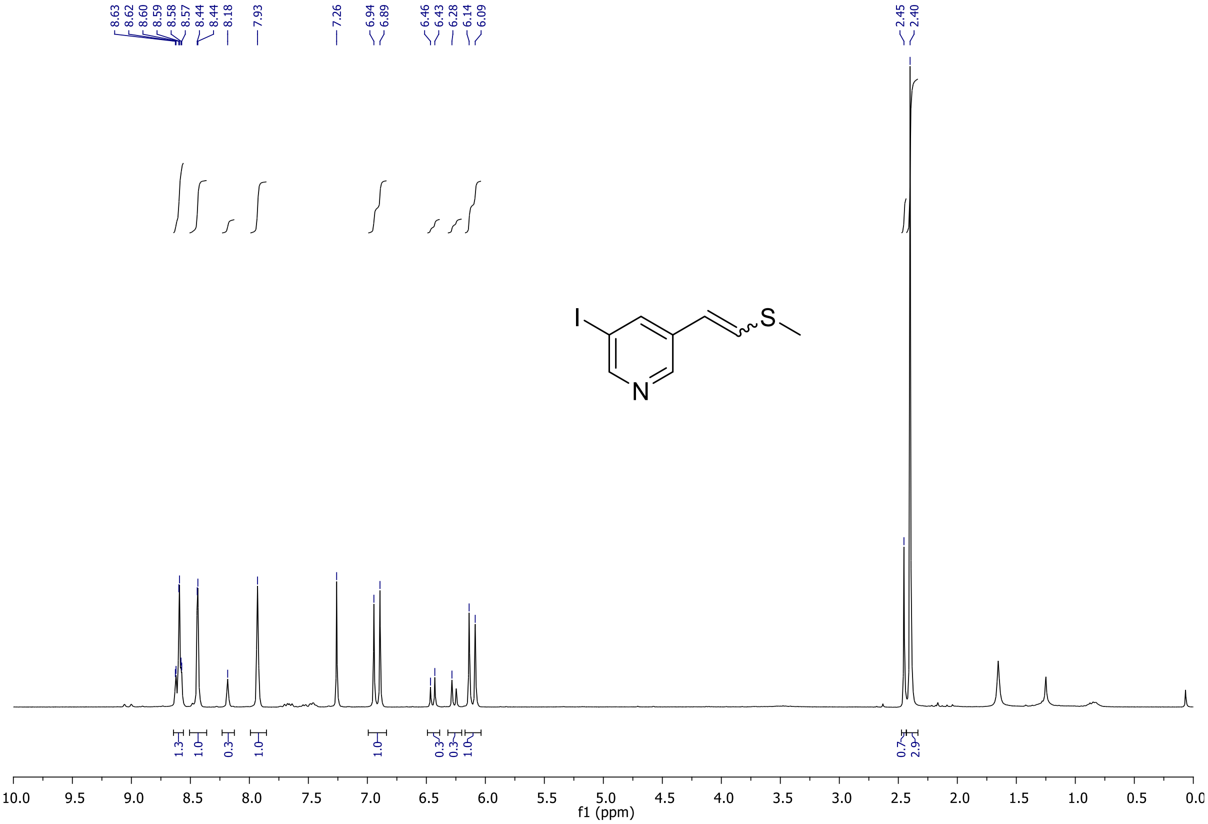

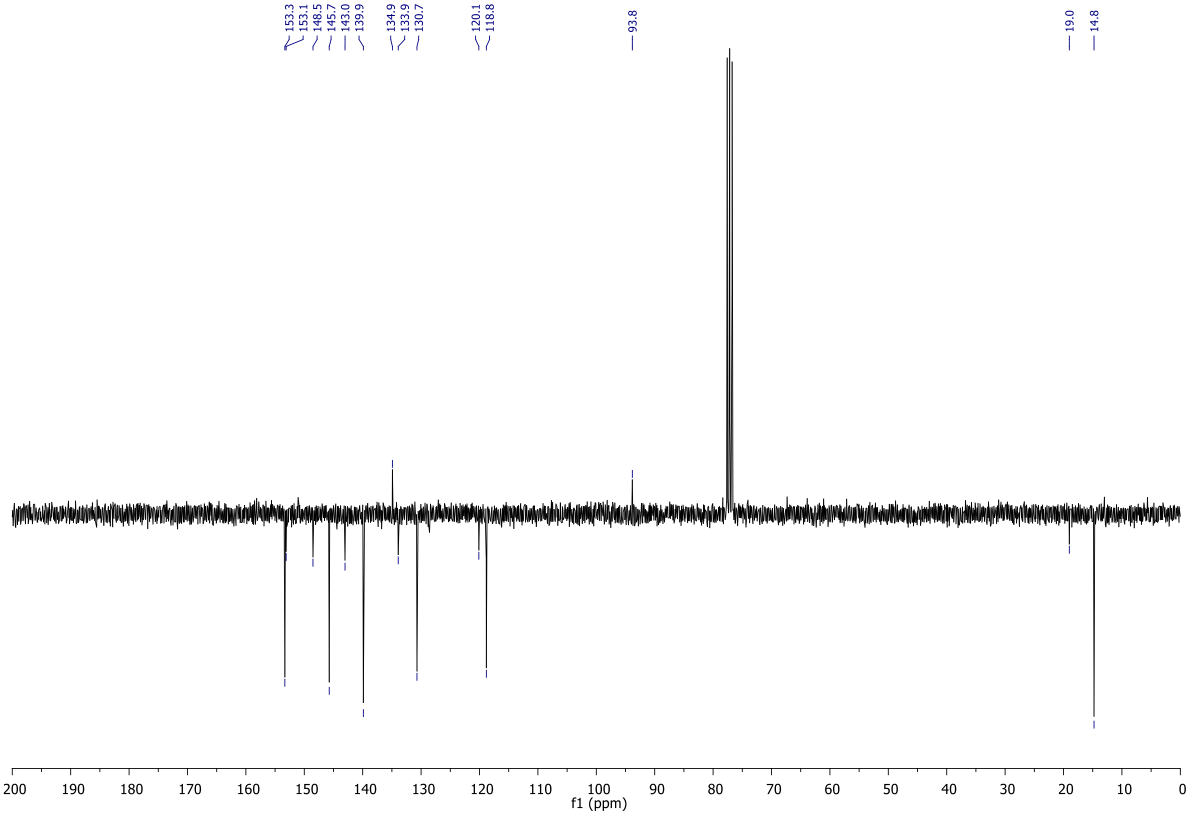

3-Iodo-5-(2-(methylthio)ethyl)pyridine (**44c**)

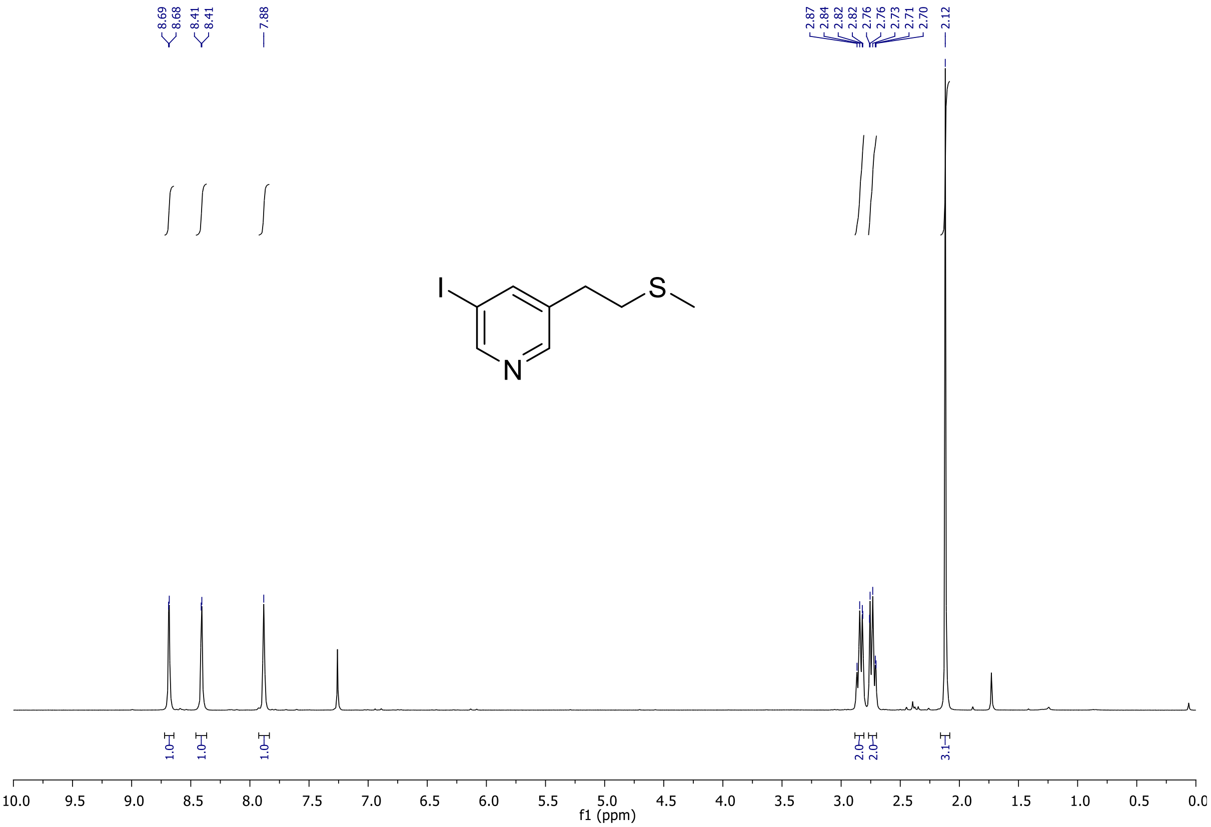

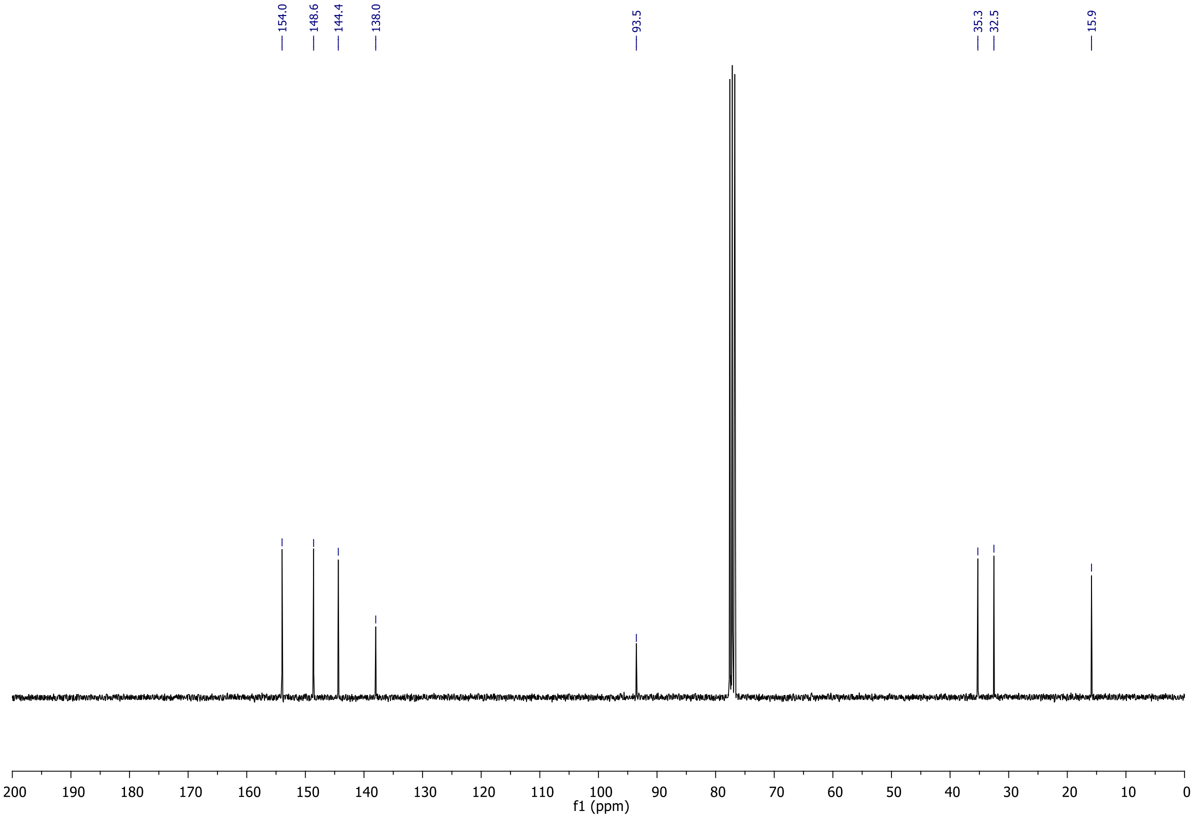

3-(2-(Methylthio)ethyl)-5-(4,4,5,5-tetramethyl-1,3,2-dioxaborolan-2-yl)pyridine (**44**)

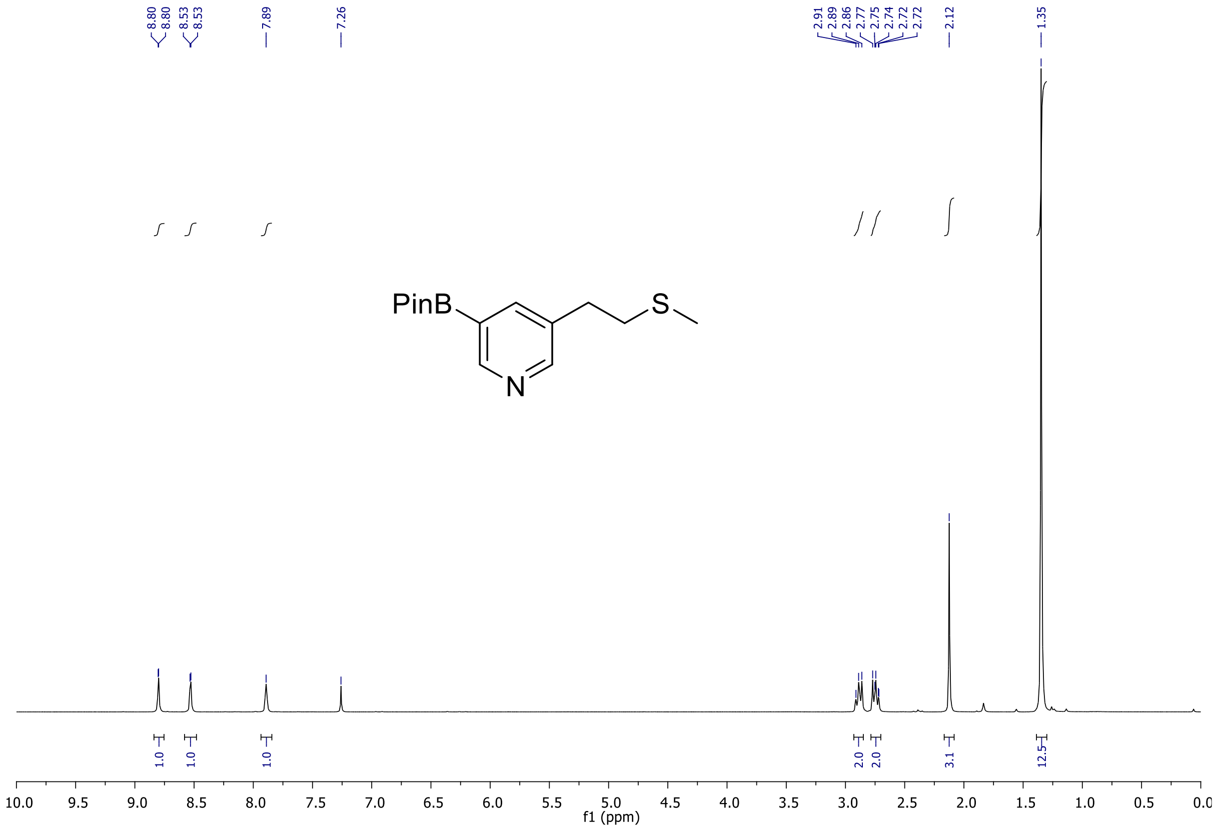

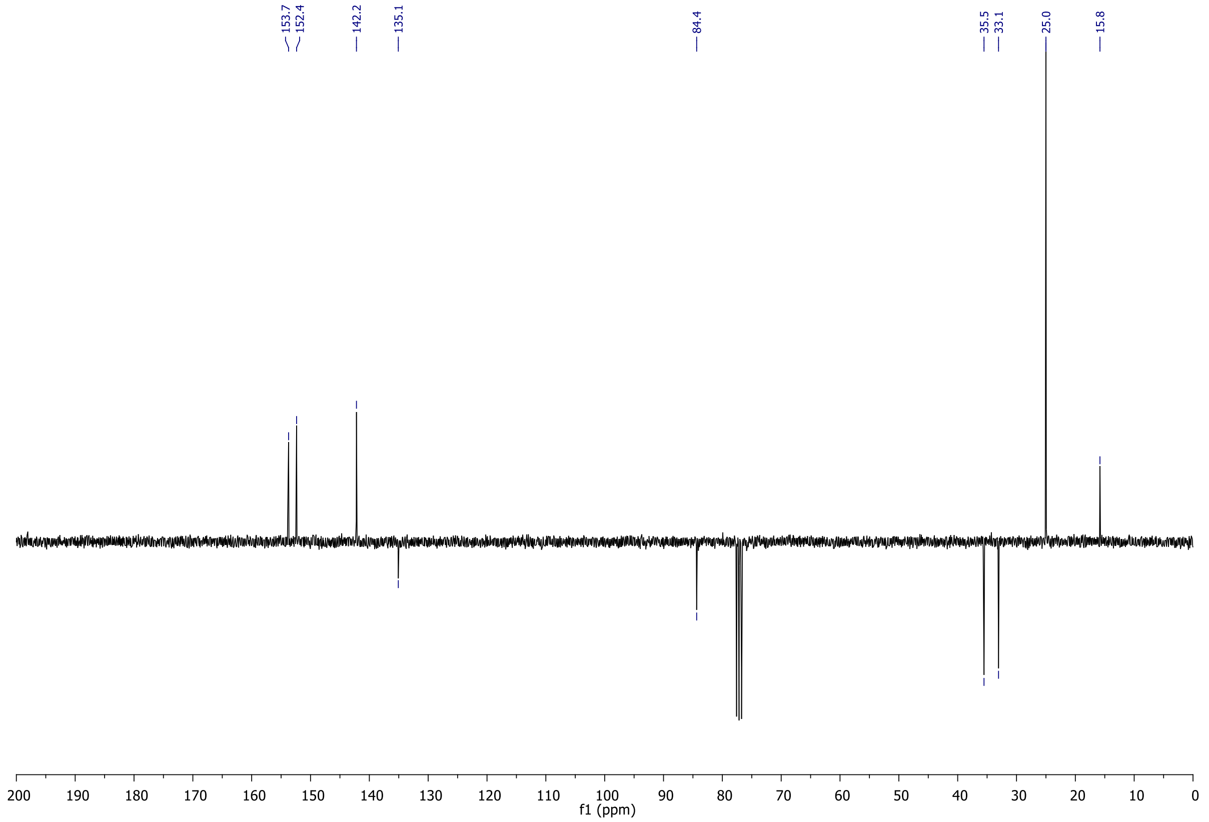

3-(2-(1,3-Dioxolan-2-yl)ethyl)-5-bromopyridine (**45a**)

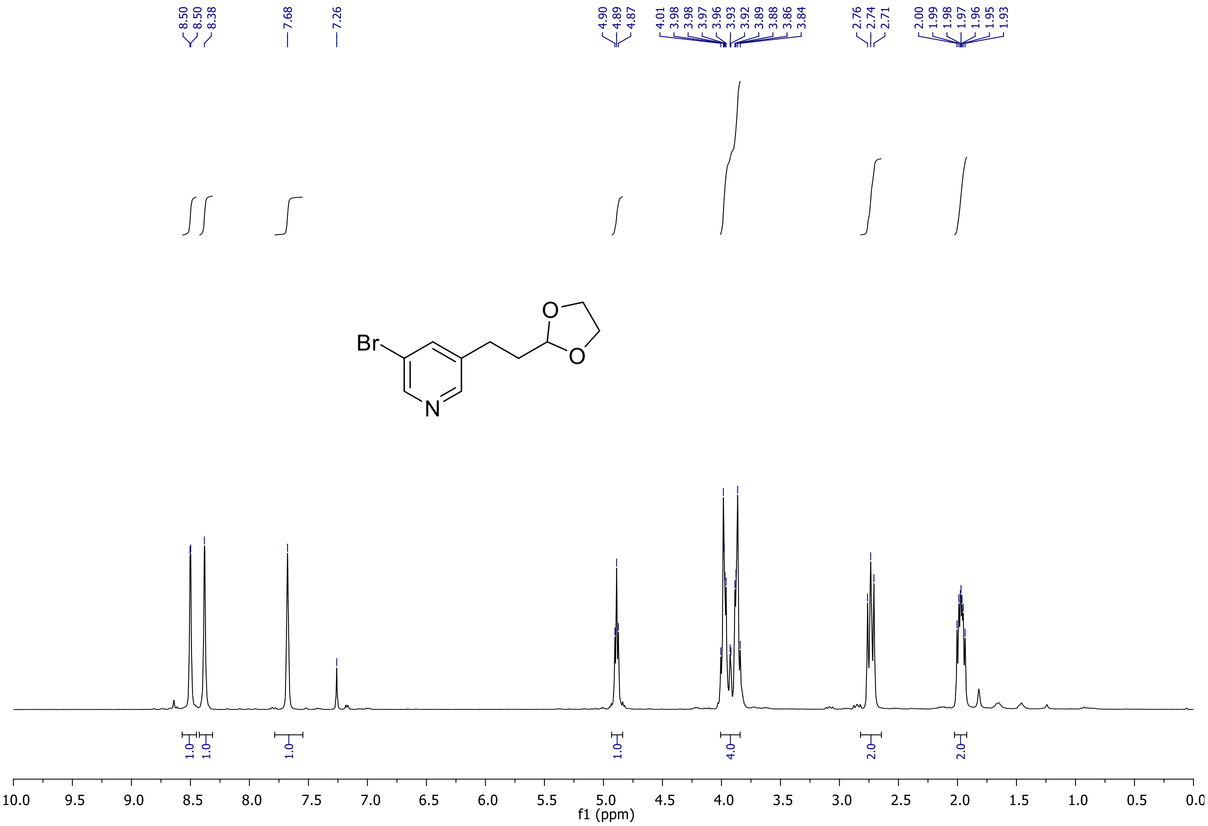

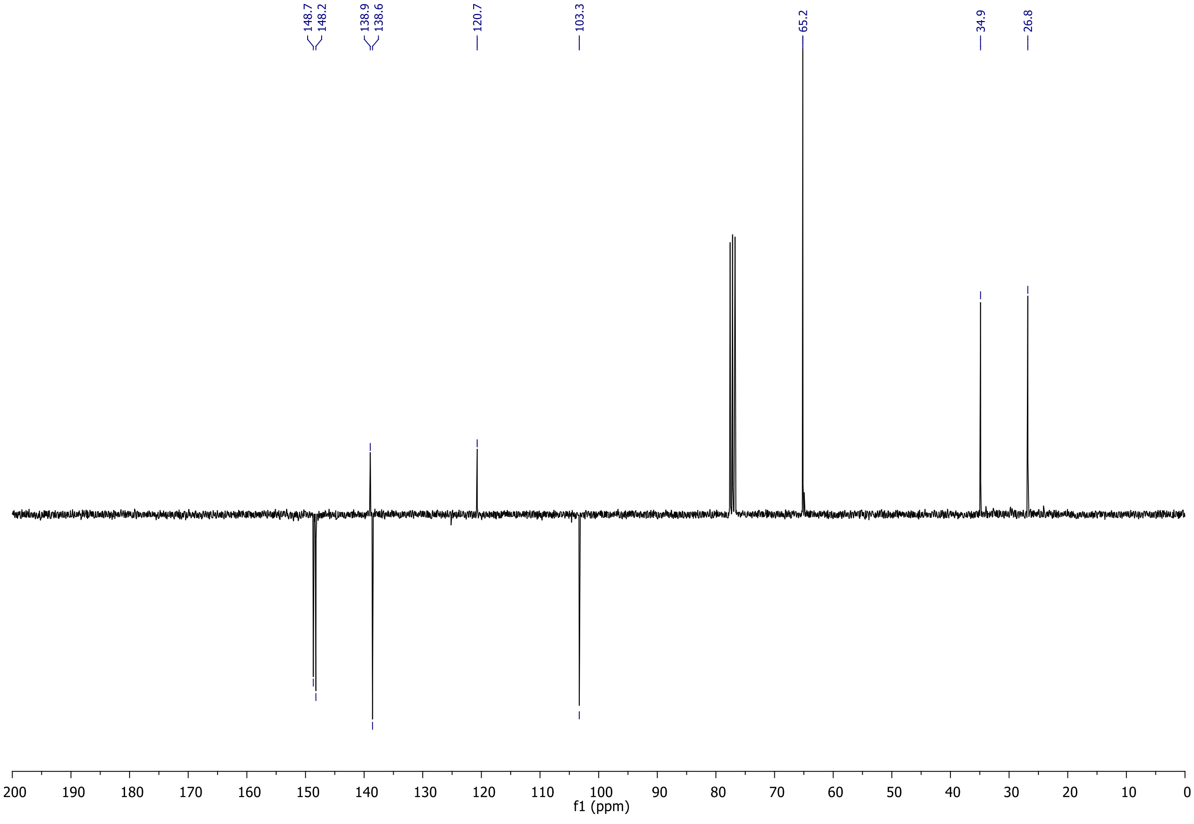

3-((5-Bromopyridin-3-yl)methyl)-1*H*-indole (**45b**)

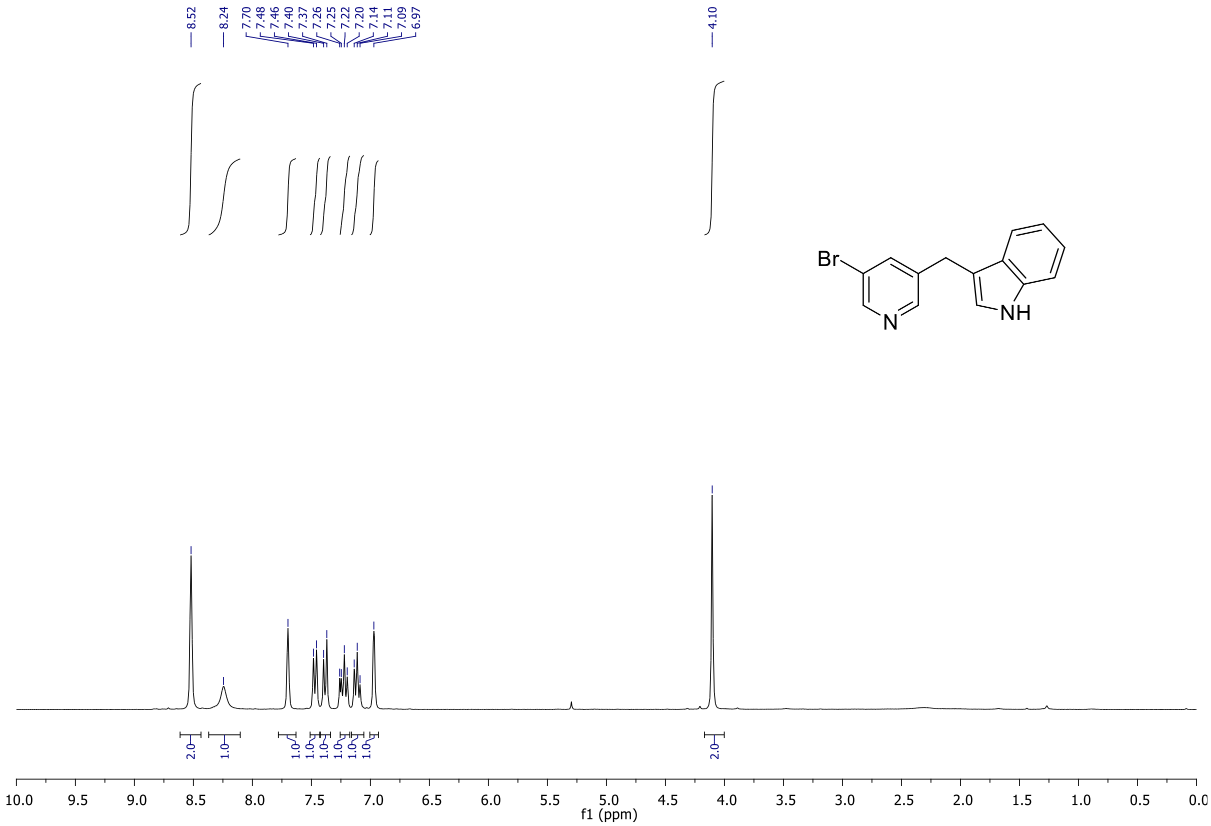

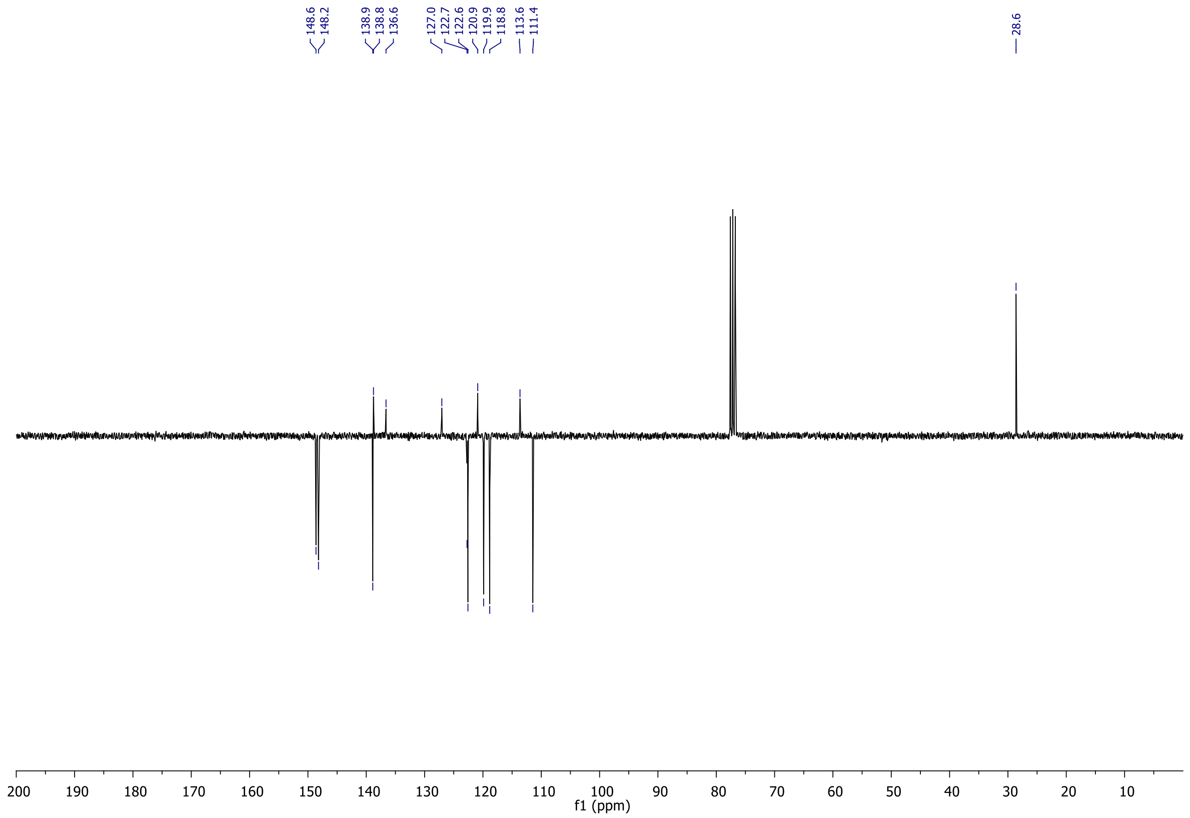

3-((5-(4,4,5,5-Tetramethyl-1,3,2-dioxaborolan-2-yl)pyridin-3-yl)methyl)-1*H*-indole (**45**)

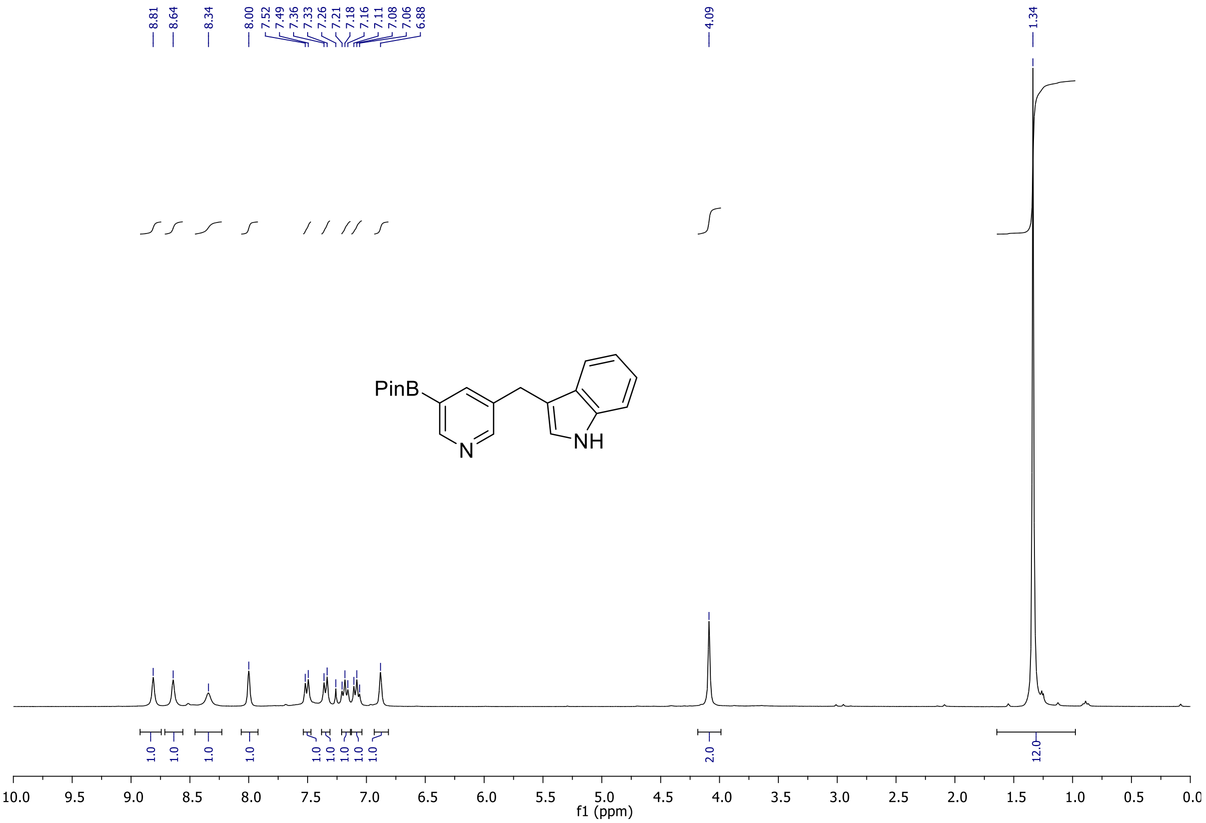

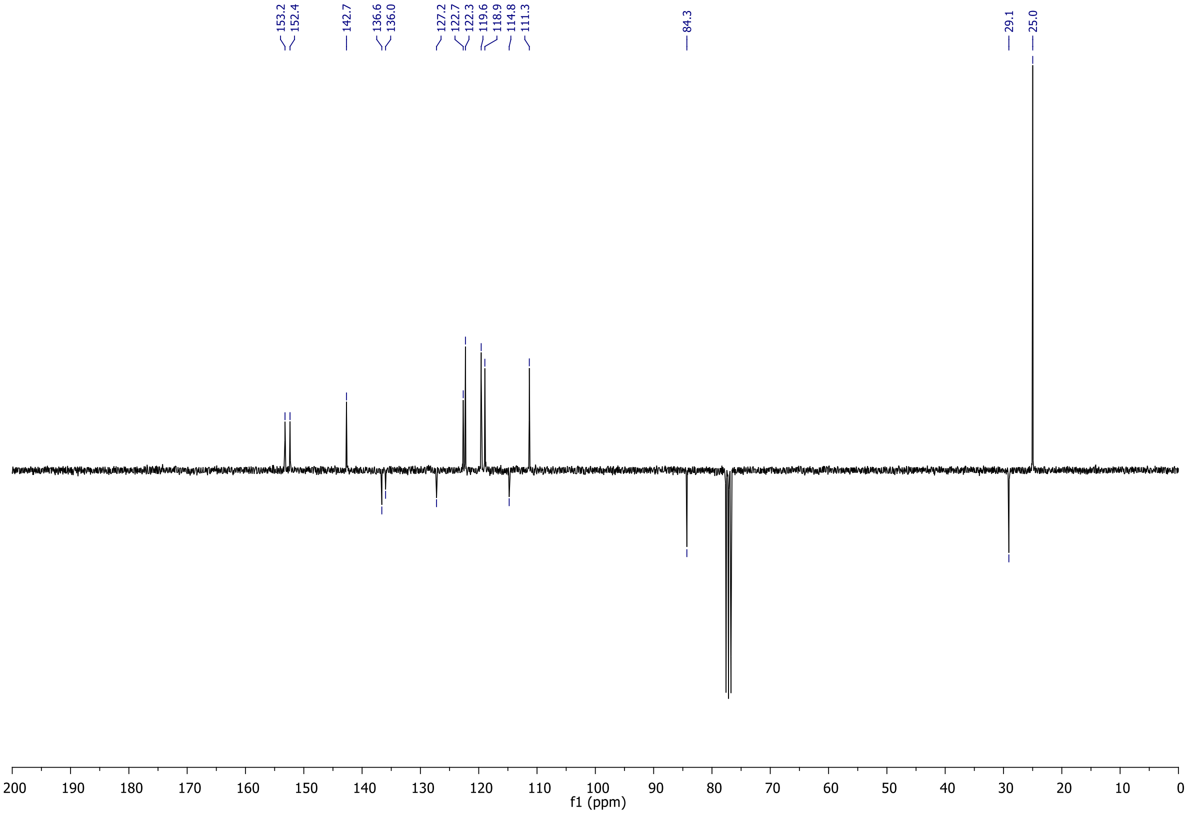

(5-Bromopyridin-3-yl)methanol (**46a**)

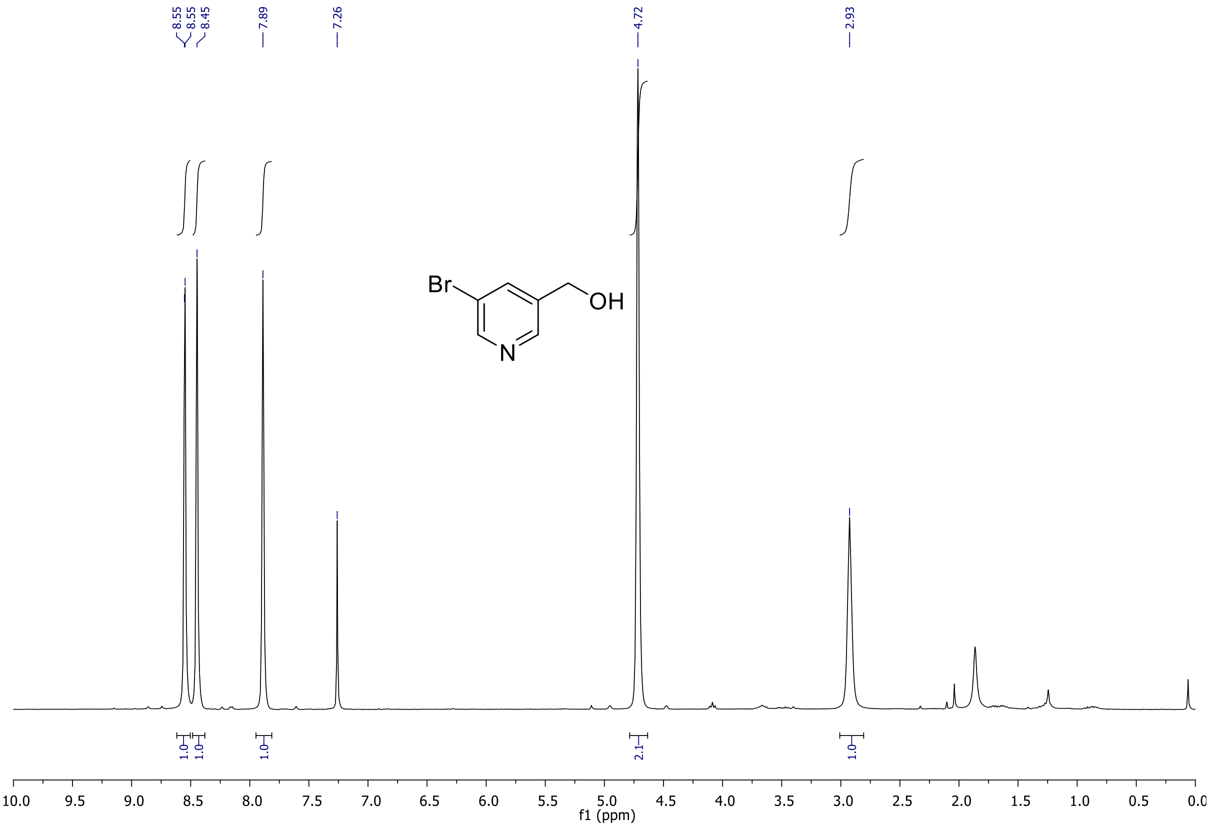

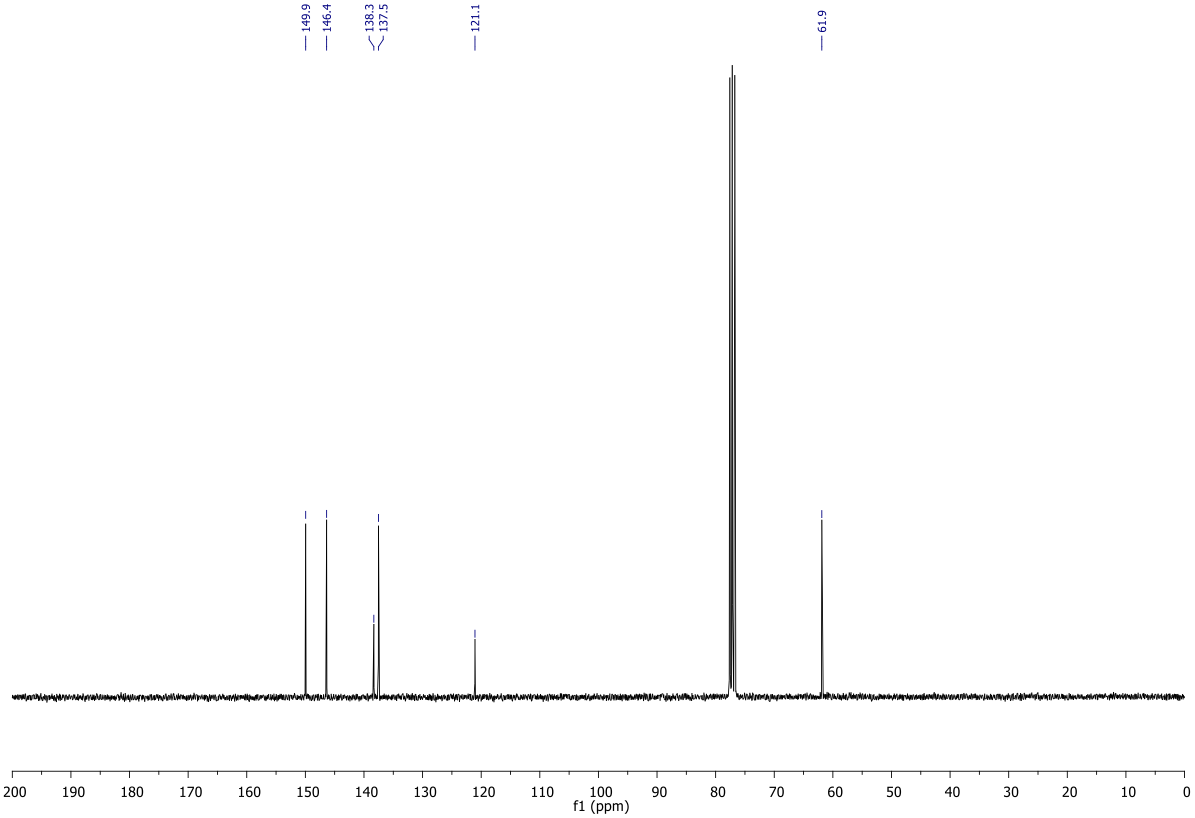

(5-Iodopyridin-3-yl)methanol (**46b**)

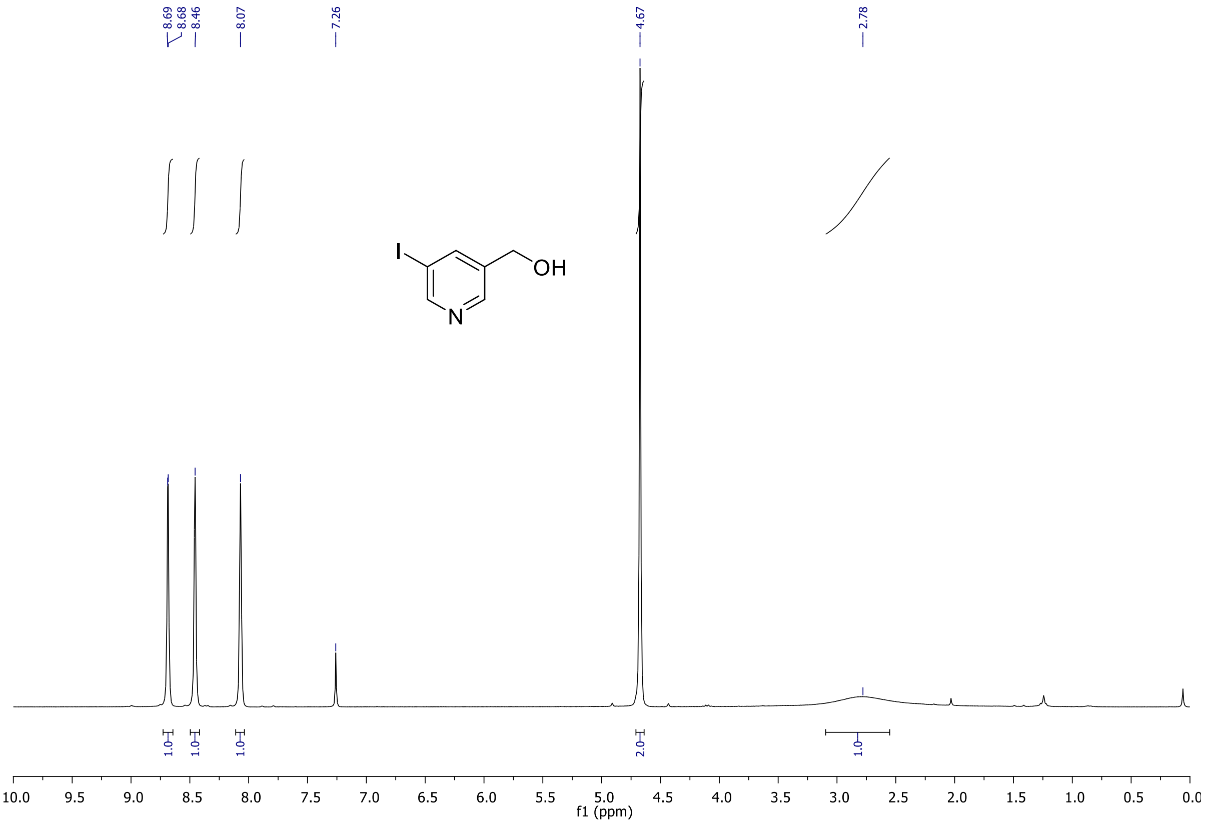

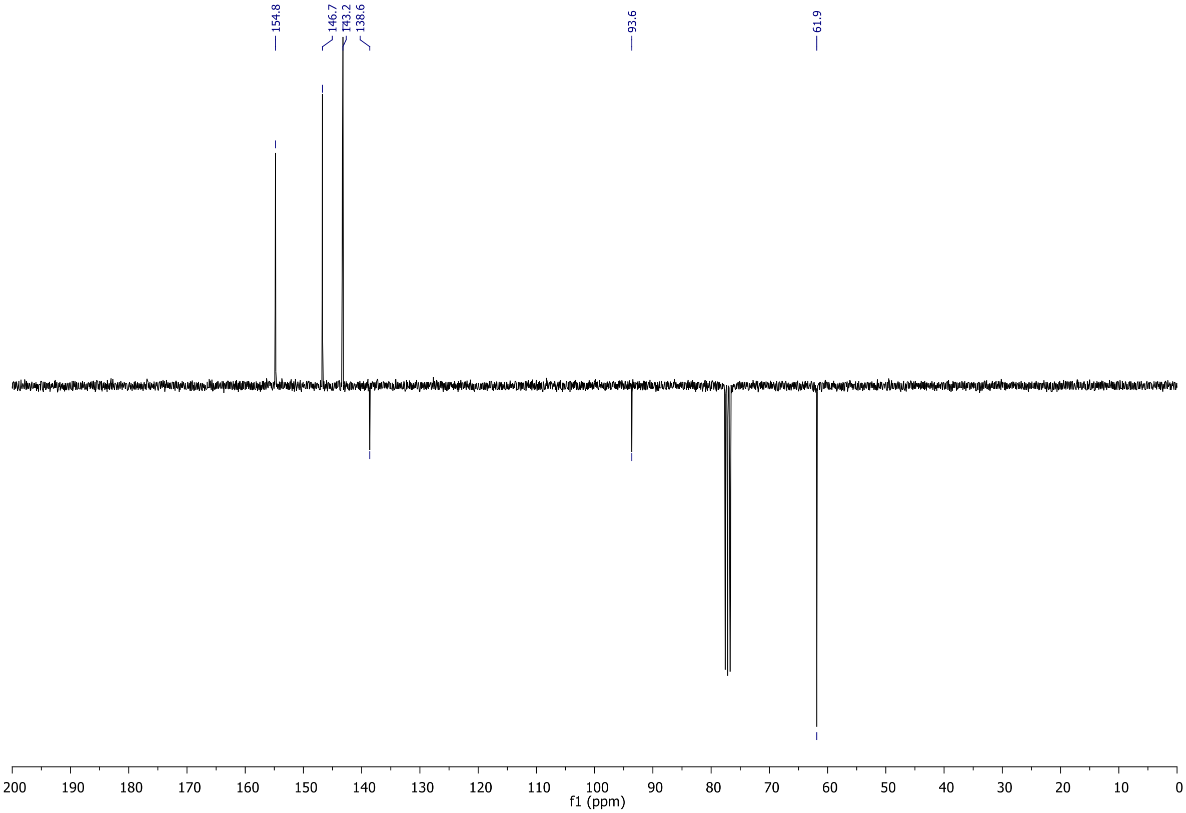

3-(((*tert*-Butyldiphenylsilyl)oxy)methyl)-5-iodopyridine (**46c**)

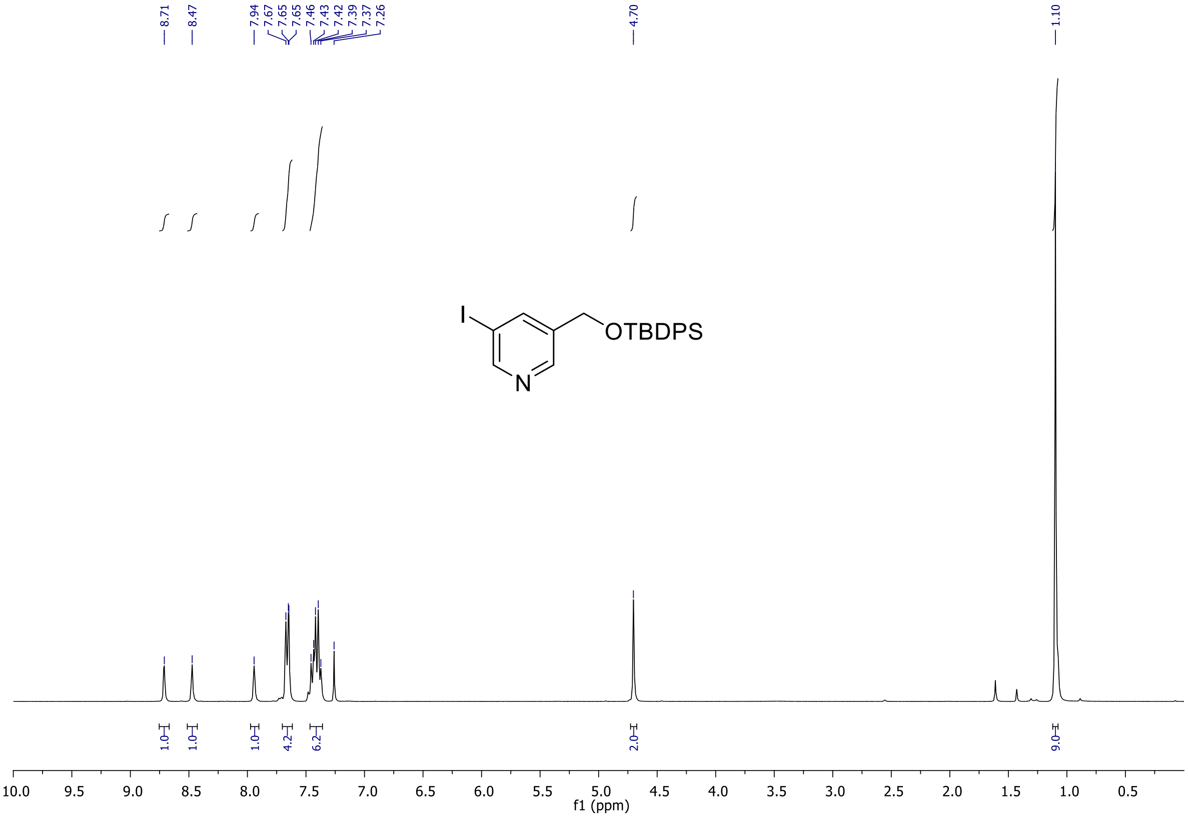

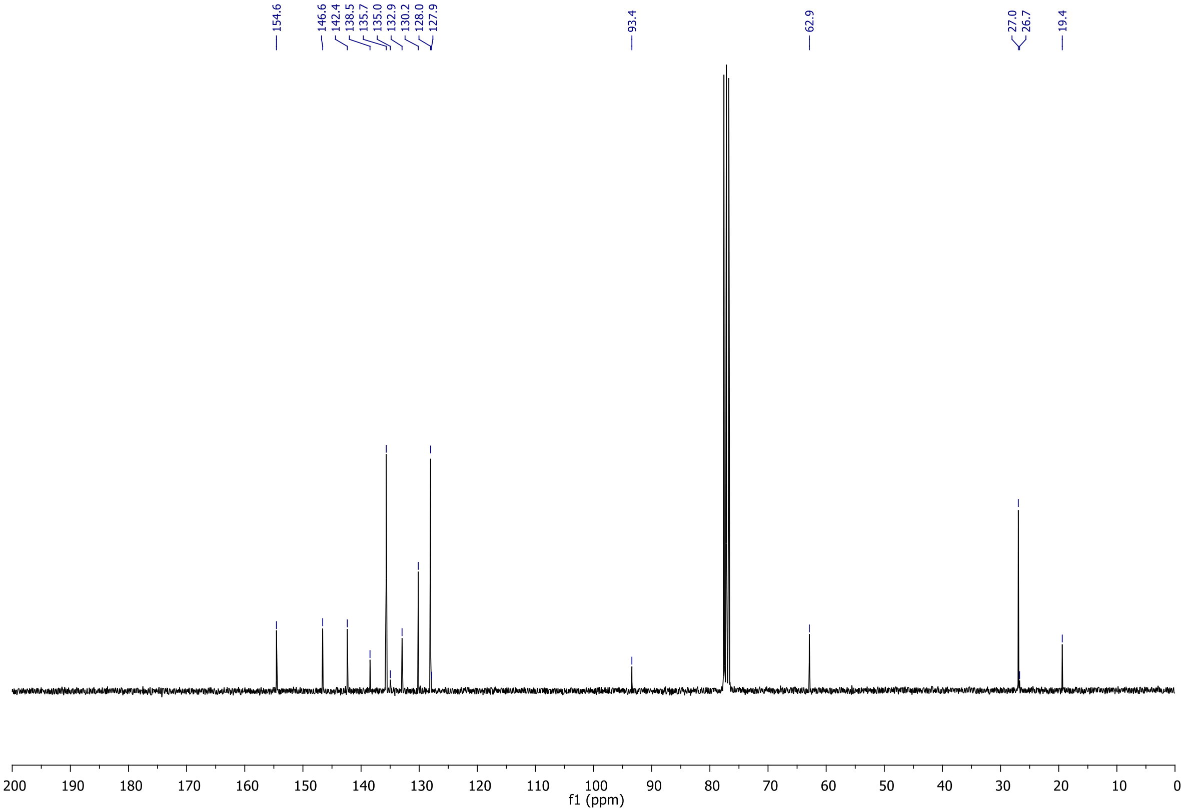

3-(((*tert*-Butyldiphenylsilyl)oxy)methyl)-5-(4,4,5,5-tetramethyl-1,3,2-dioxaborolan-2-yl)pyridine (**46d**)

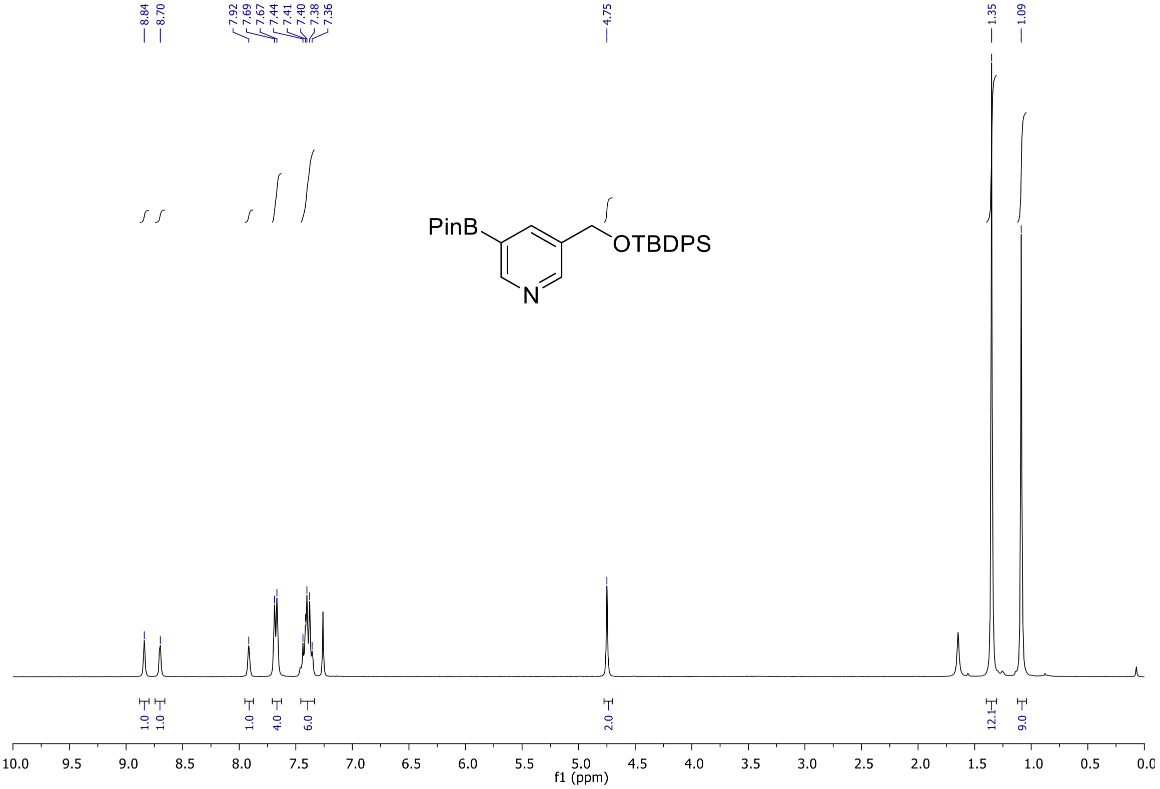

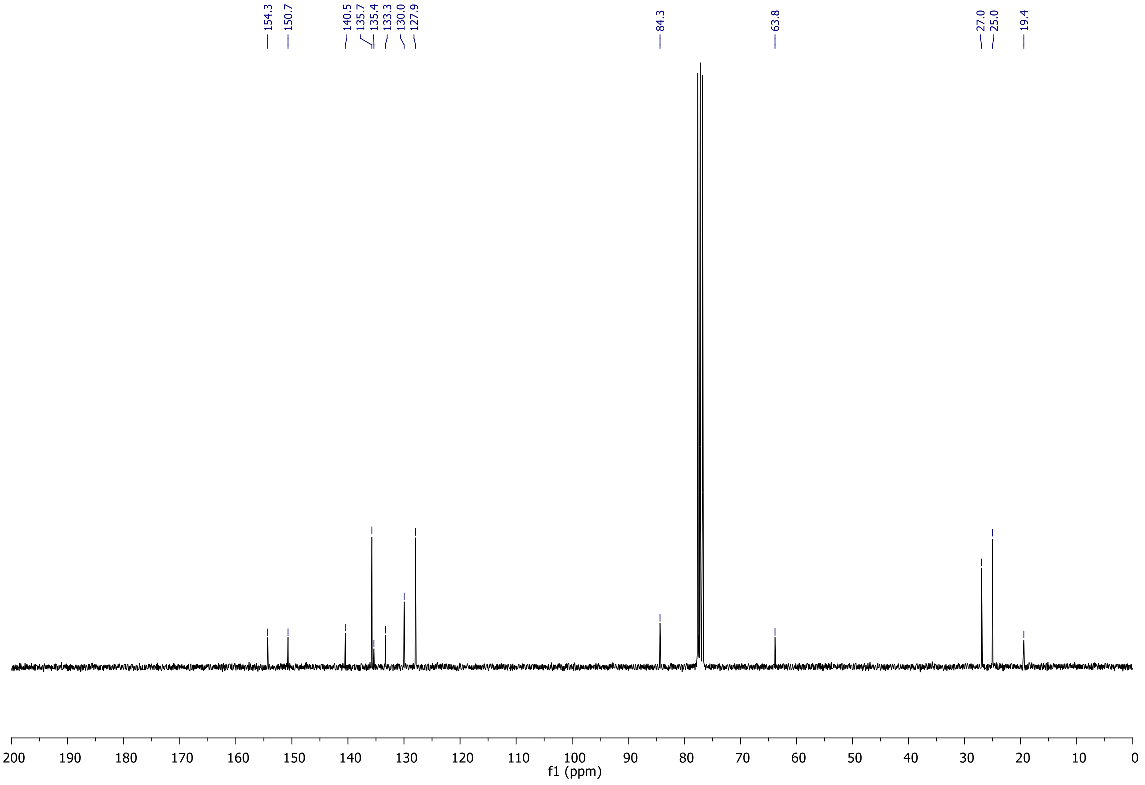

3-Benzyl-5-bromopyridine (**47a**)

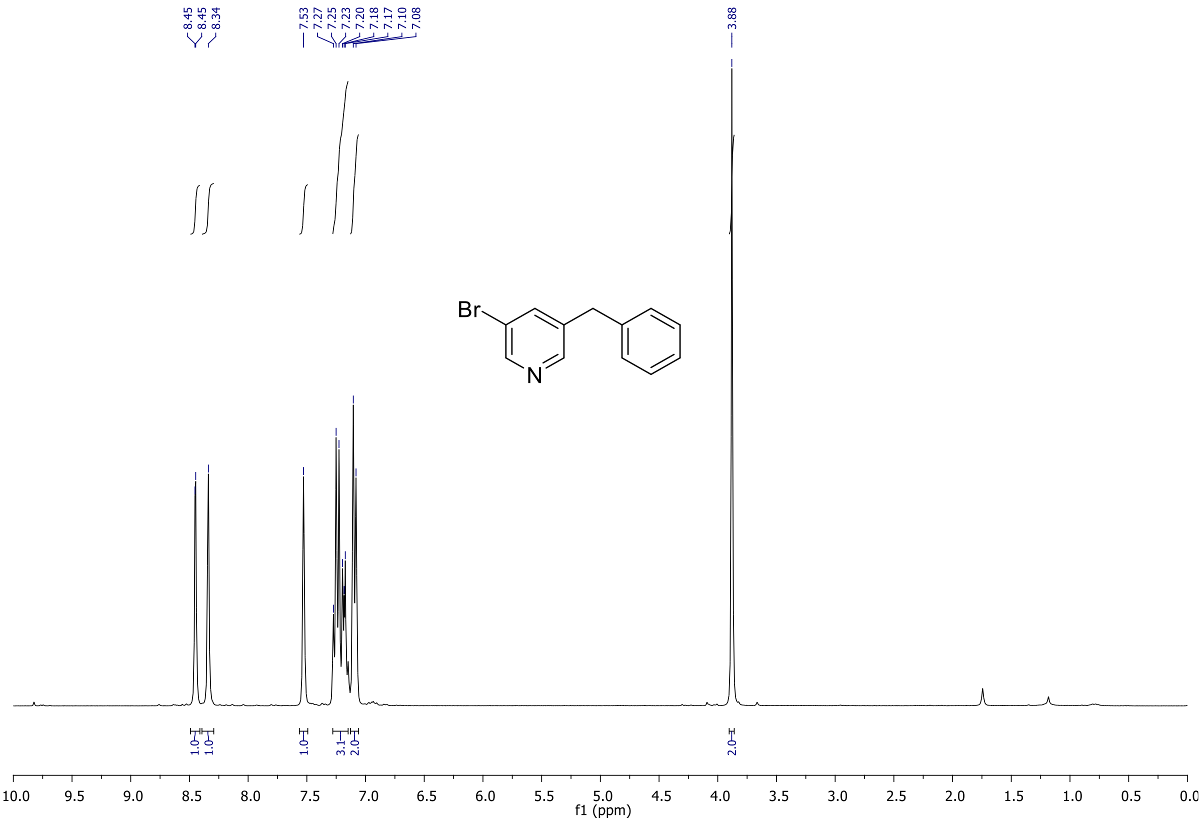

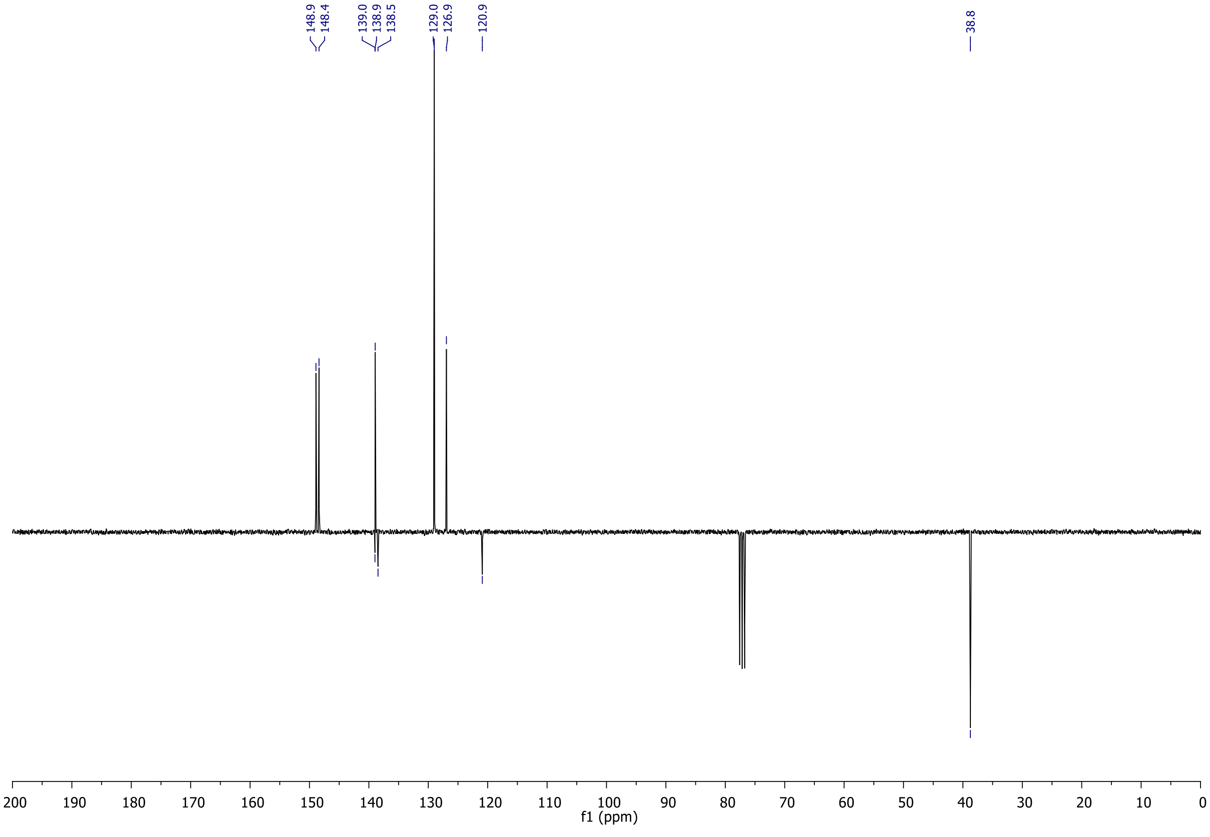

3-Benzyl-5-(4,4,5,5-tetramethyl-1,3,2-dioxaborolan-2-yl)pyridine (**47**)

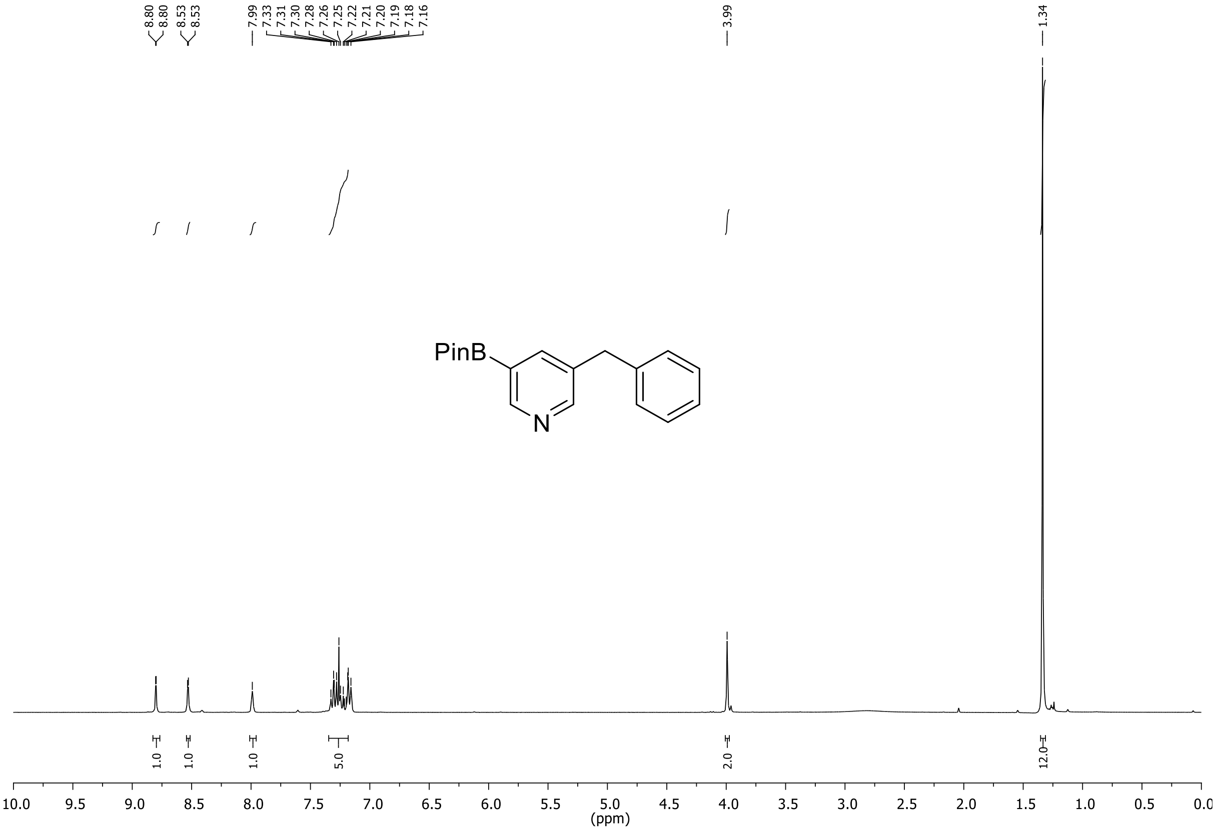

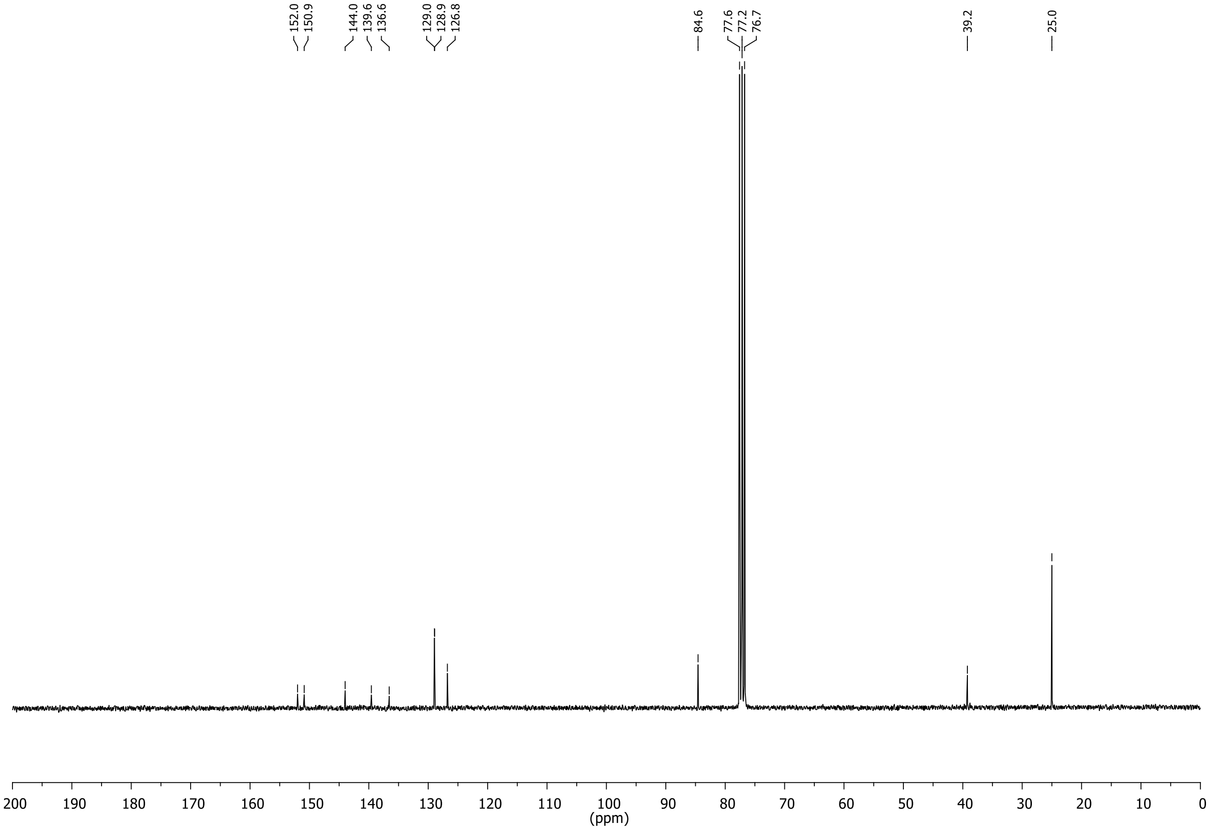

Leu-Trp-Met **(48)**

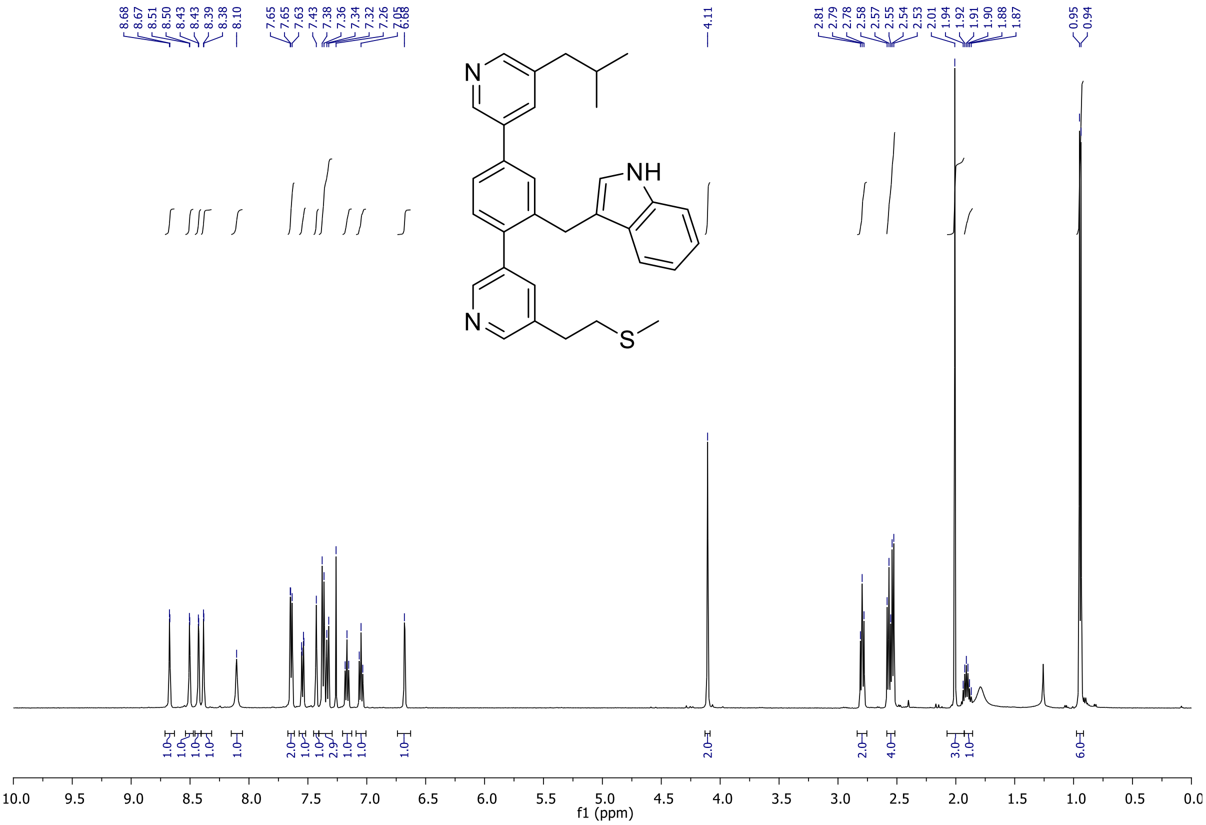

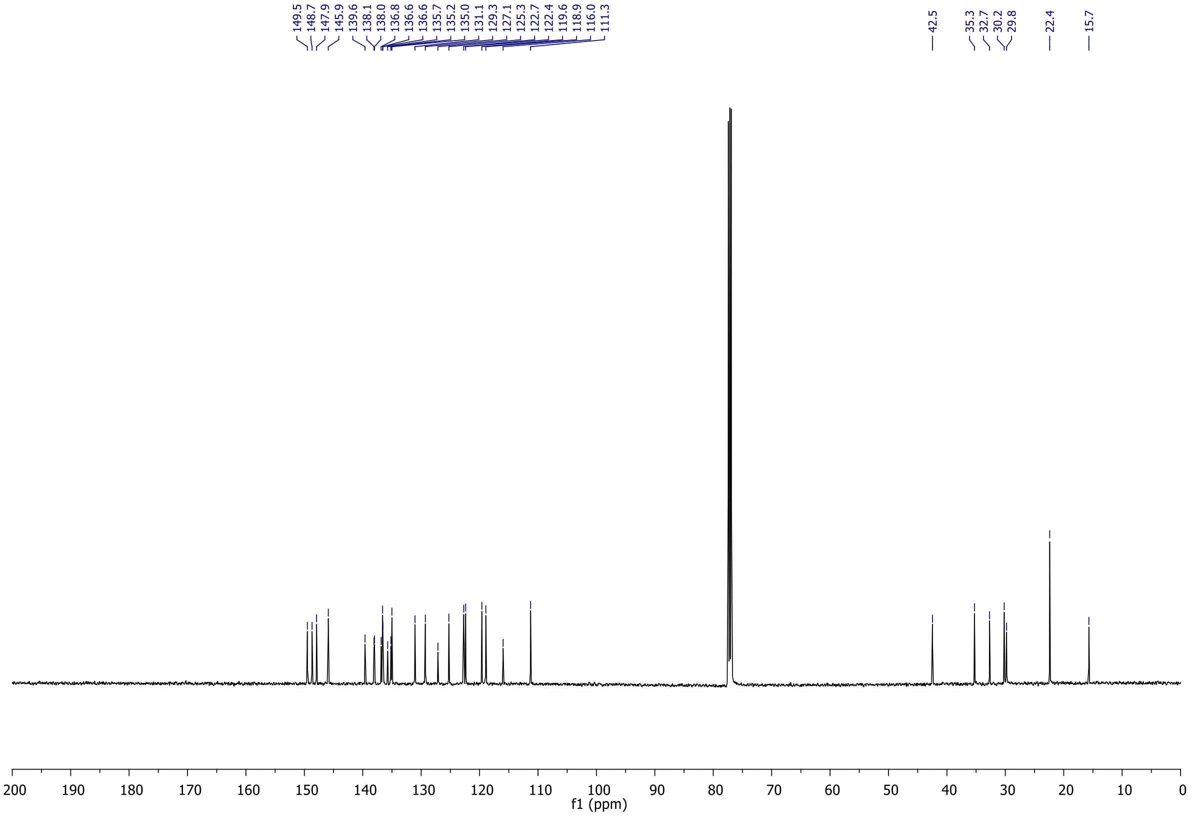

Leu-Val-Trp (**49**)

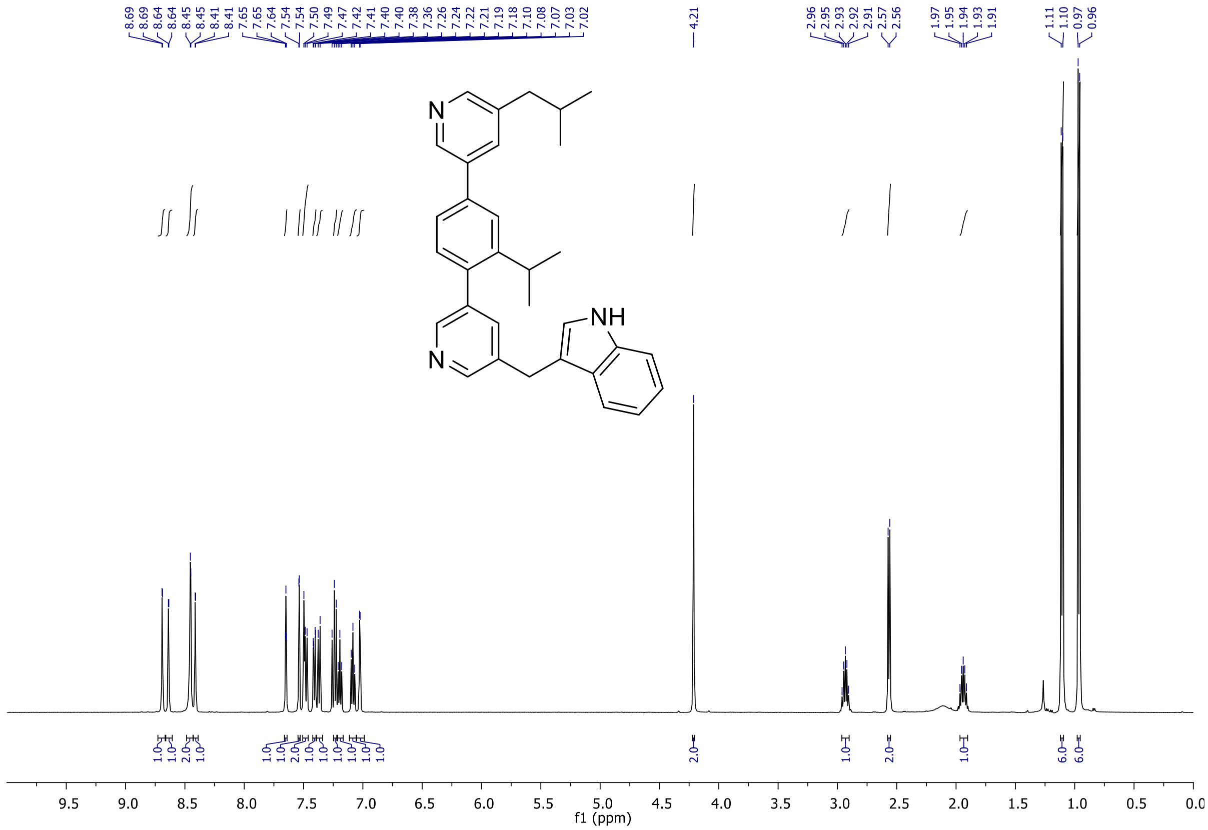

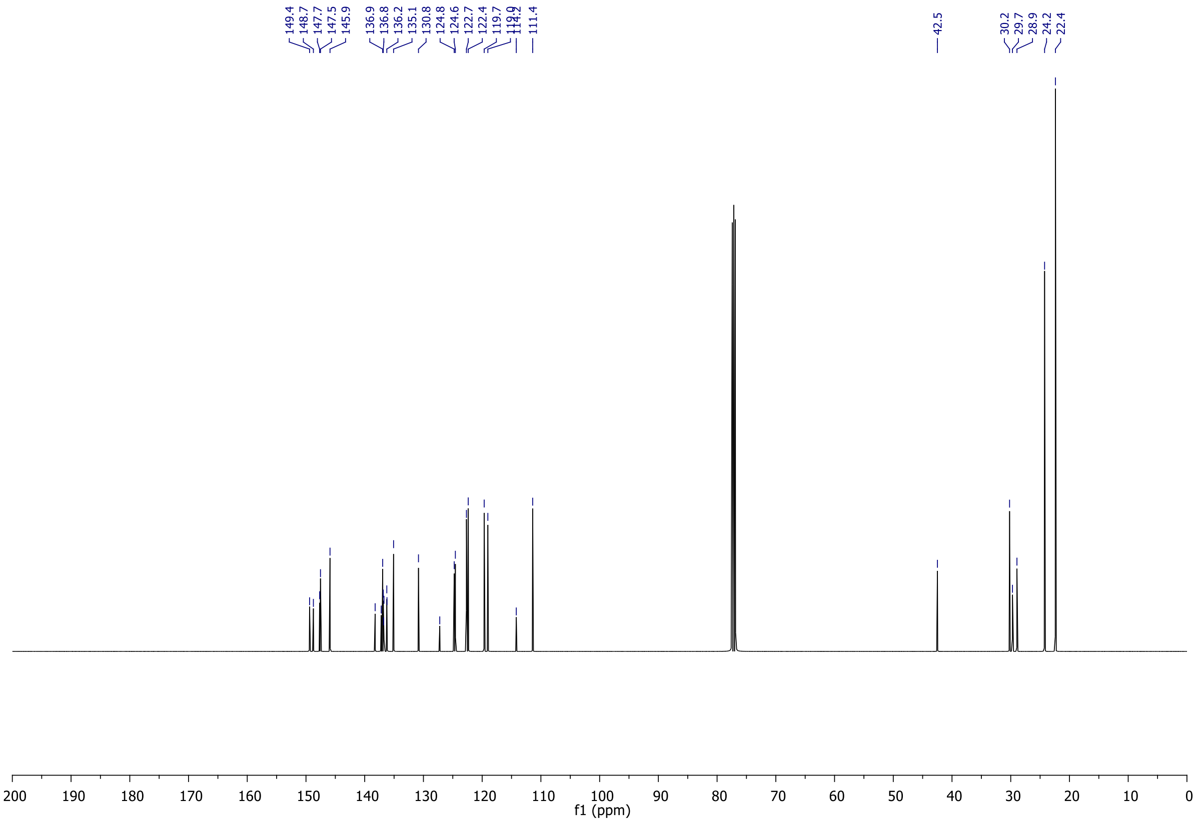

Leu-Asn-Ser **(50)**

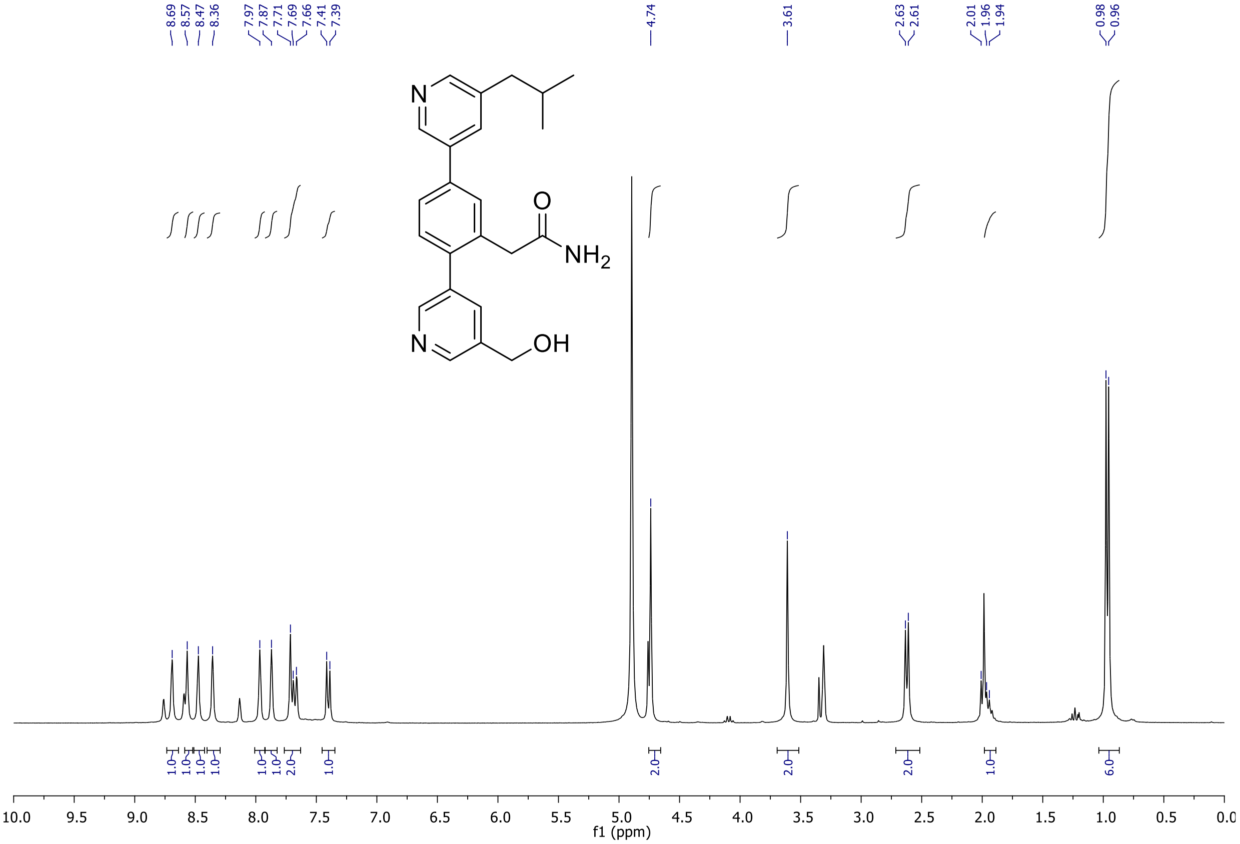

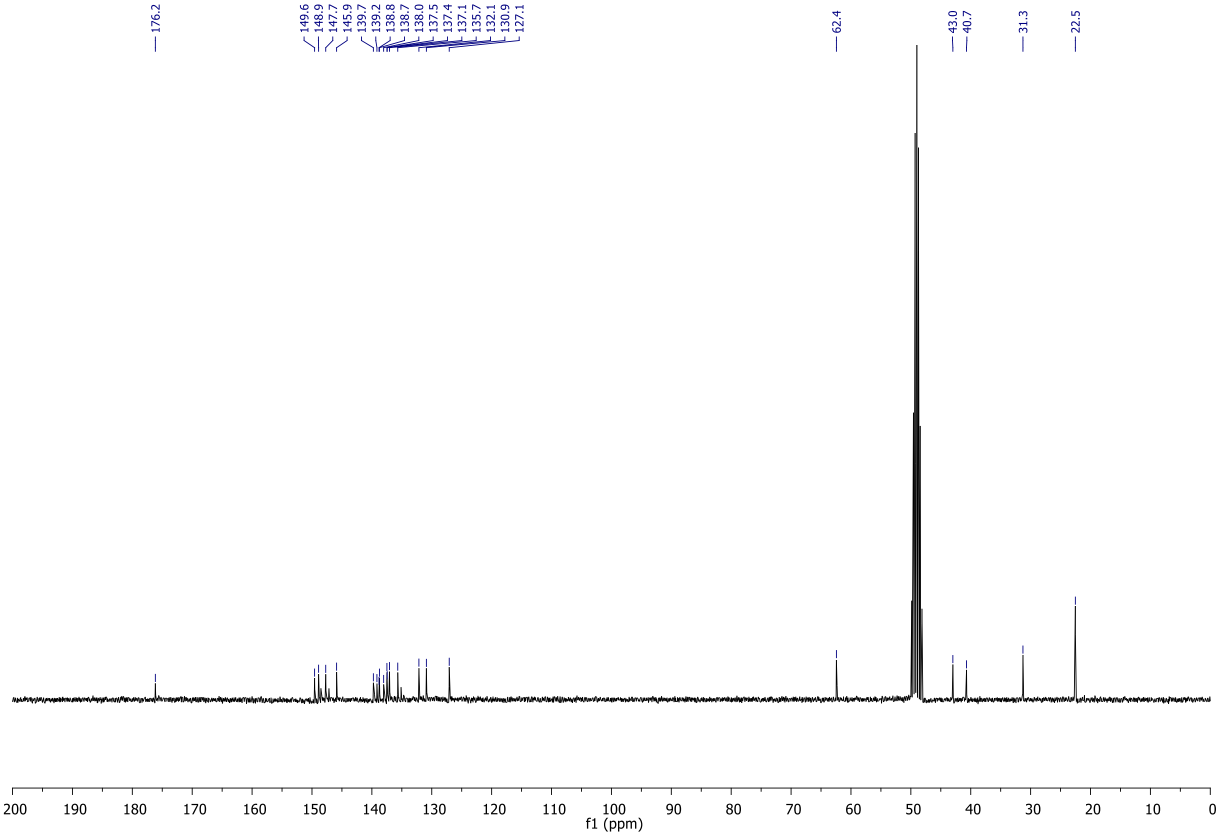

Ethyl 3-(2-(5-benzylpyridin-3-yl)-5-(5-isobutylpyridin-3-yl)phenyl)propanoate (**51a**)

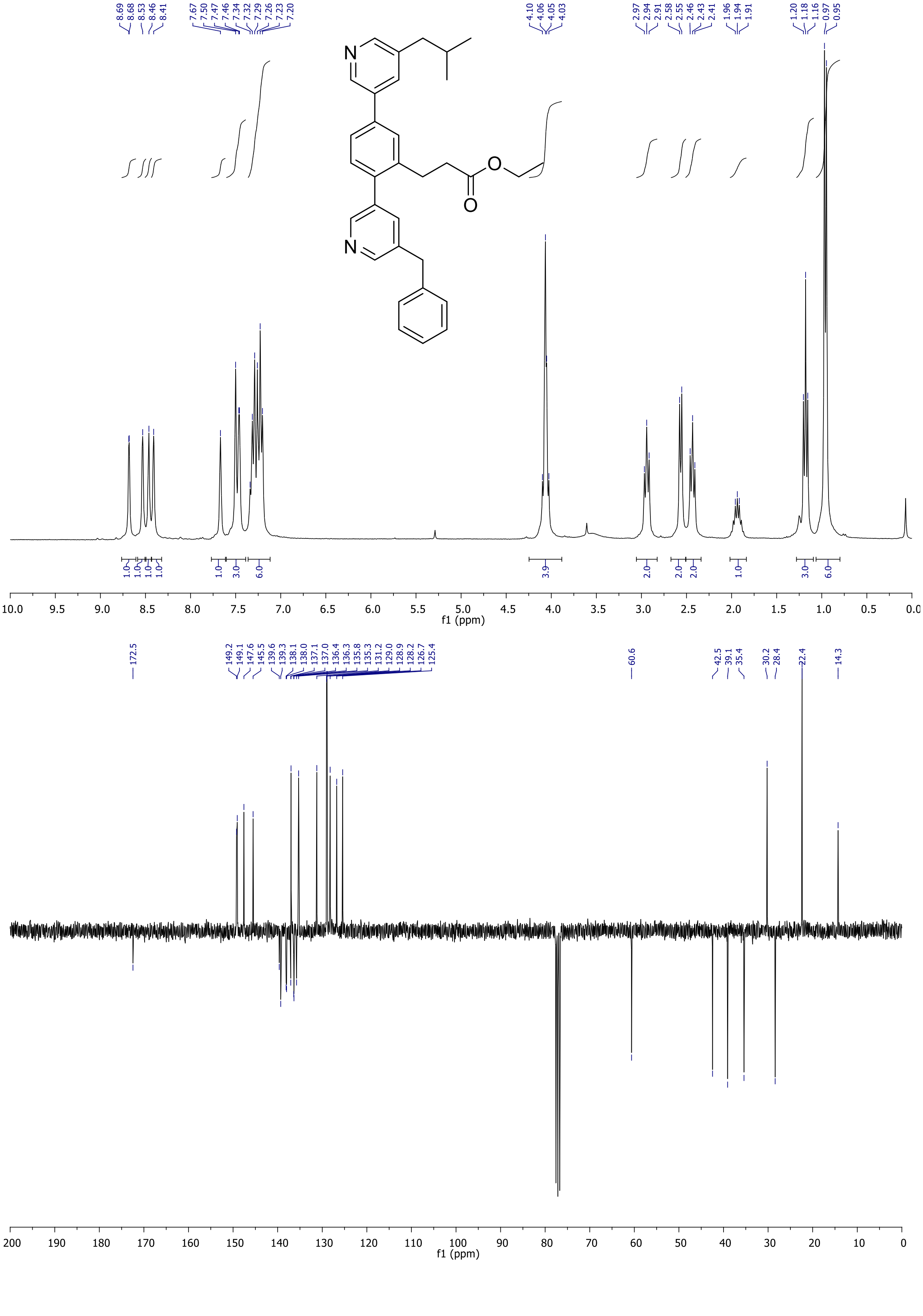

Leu-Glu-Phe (**51**)

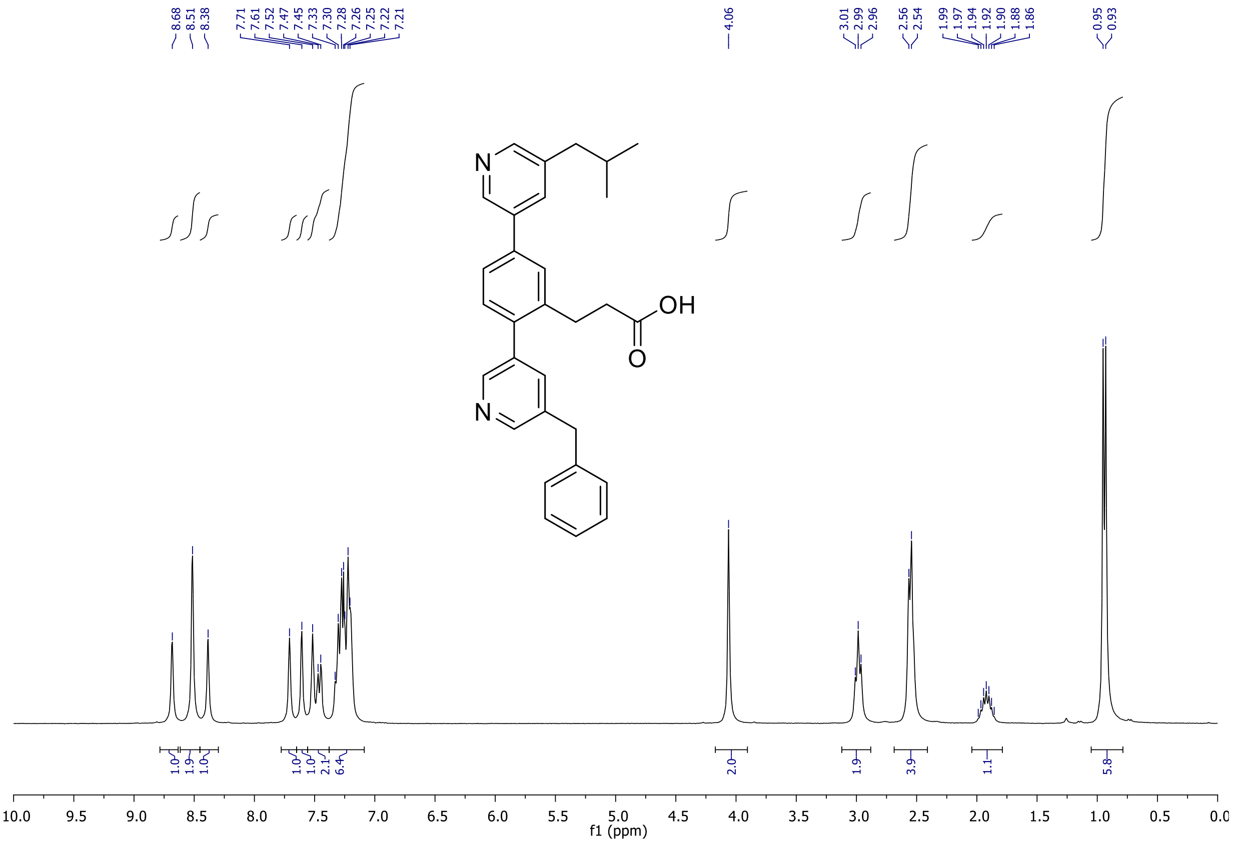

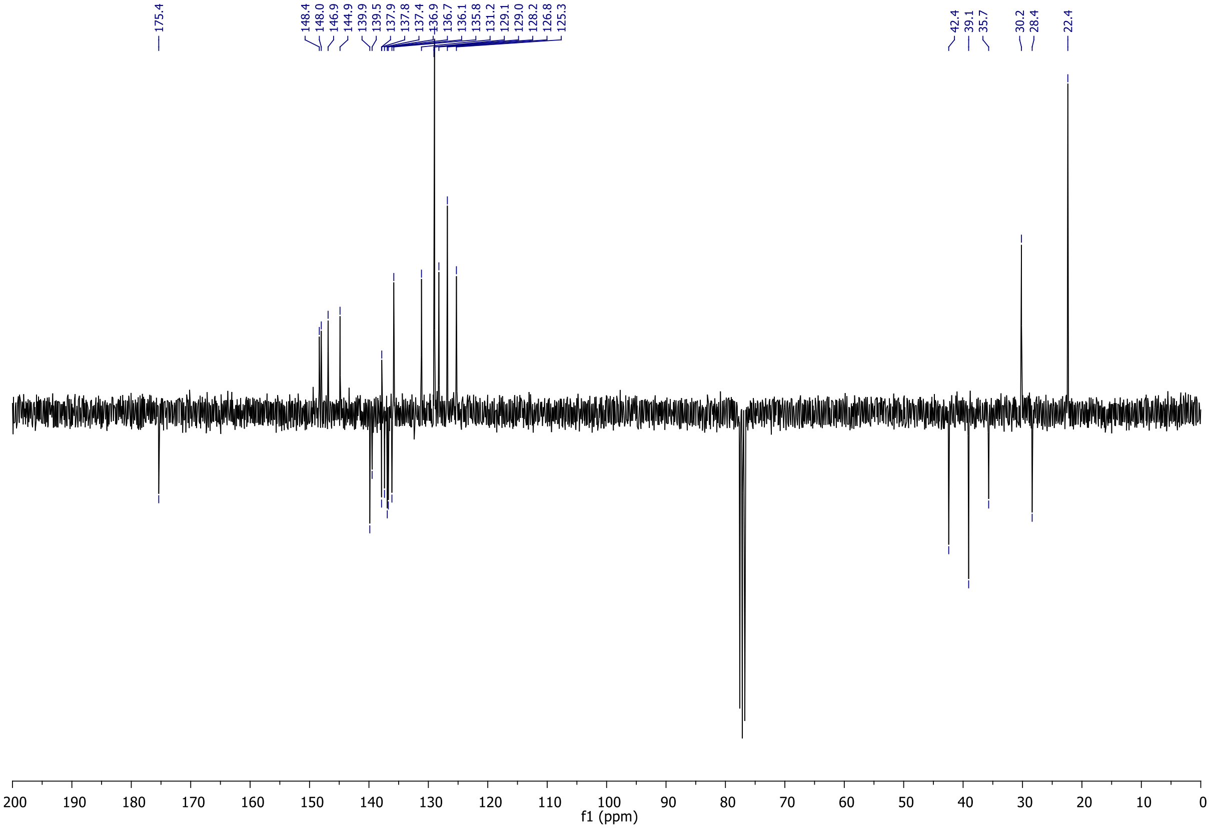

Supplement: Supplementary file 1 — Supporting Information [file EJOC-2022-0-s001.pdf]
